# Dynamic kinetic resolution of γ,γ-disubstituted indole 2-carboxaldehydes *via* NHC-Lewis acid cooperative catalysis for the synthesis of tetracyclic ε-lactones†

**DOI:** 10.1039/d2sc03745a

**Published:** 2022-08-29

**Authors:** Kuruva Balanna, Soumen Barik, Sayan Shee, Rajesh G. Gonnade, Akkattu T. Biju

**Affiliations:** Department of Organic Chemistry, Indian Institute of Science Bangalore-560012 India atbiju@iisc.ac.in https://orgchem.iisc.ac.in/atbiju/; Centre for Materials Characterization, CSIR-National Chemical Laboratory Dr Homi Bhabha Road Pune-411008 India

## Abstract

The ubiquity of ε-lactones in various biologically active compounds inspired the development of efficient and enantioselective routes to these target compounds. Described herein is the enantioselective synthesis of indole-fused ε-lactones by the N-heterocyclic carbene (NHC)-Lewis acid cooperative catalyzed dynamic kinetic resolution (DKR) of *in situ* generated γ,γ-disubstituted indole 2-carboxaldehydes. The Bi(OTf)_3_-catalyzed Friedel–Crafts reaction of indole-2-carboxaldehyde with 2-hydroxy phenyl *p*-quinone methides generates γ,γ-disubstituted indole 2-carboxaldehydes, which in the presence of NHC and Bi(OTf)_3_ afforded the desired tetracyclic ε-lactones in up to 93% yield and >99 : 1 er. Moreover, preliminary studies on the mechanism of this formal [4 + 3] annulation are also provided.

## Abstract 2

NHC-Lewis acid cooperative catalyzed dynamic kinetic resolution (DKR) of transiently generated γ,γ-disubstituted indole 2-carboxaldehydes leading to the enantioselective synthesis of tetracyclic ε-lactones is reported.

## Graphical abstract

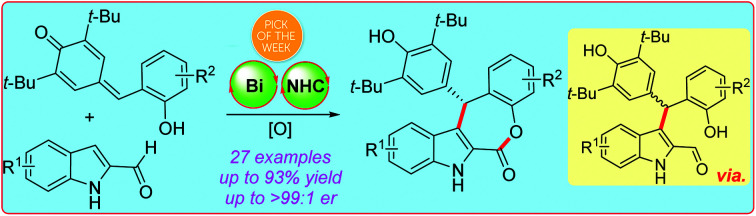

## Introduction

Functionalized ε-lactones are important structural motifs present in various biologically active compounds and this core is responsible for the flavor and aroma in many natural products.^1^ For instance, the natural products rubellins A and B have the benzo-fused ε-lactone moiety connected to the anthraquinone unit, and they exhibit photodynamic activity.^2^ Moreover, 9-dehydroxyeurotinone and 2-*O*-methyl-9-dehydoxyeurotinone have a dibenzo-fused ε-lactone core, and they are useful due to their antimicrobial and cytotoxic activity.^3^ Given the potential applications of ε-lactone-containing compounds, the development of rapid and facile routes for the enantioselective synthesis of ε-lactone derivatives have received remarkable attention. The Baeyer-Villiger oxidation of cyclohexanones constitutes one of the traditional approaches to access ε-lactones.^4^ Moreover, transition metal-catalyzed ring-expansion reactions and carbonylation processes could also provide straightforward access to ε-lactones.^5^ Herein, we report the enantioselective synthesis of tetracyclic indole-fused ε-lactones by the N-heterocyclic carbene (NHC)-Lewis acid catalyzed dynamic kinetic resolution (DKR) of *in situ* generated γ,γ-disubstituted indole 2-carboxaldehydes.^6,7^

**Scheme 1:**
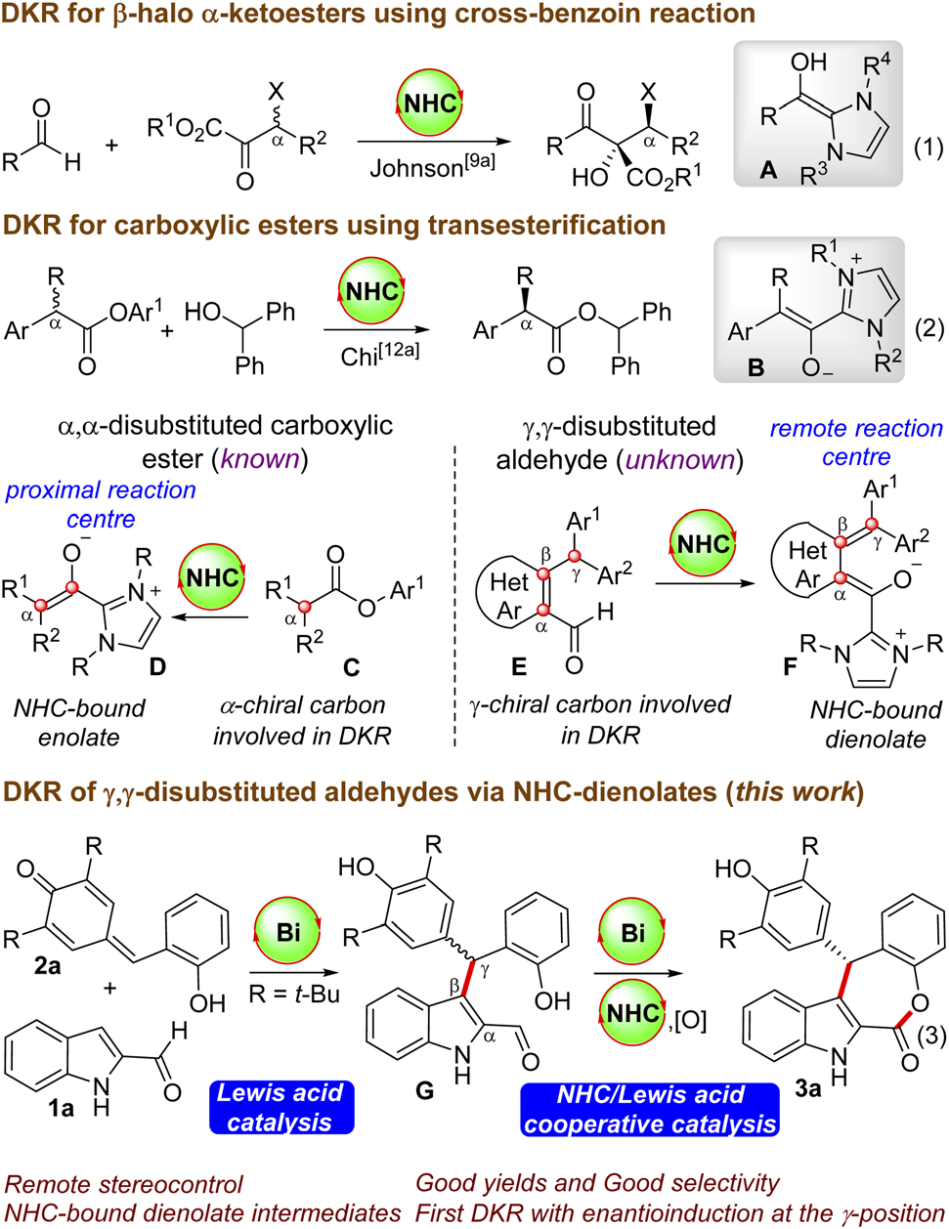
NHC-catalyzed DKR strategies.

NHC-catalyzed DKR strategies are employed for the conversion of racemic substrates to enantiomerically pure products.^8^ Generally, carbene-catalyzed DKR approaches are applicable to racemic carbonyl compounds, where the enantioinduction takes place at the α-carbon centre. For instance, Goodman and Johnson reported the DKR of β-halo α-ketoesters by utilizing the NHC-catalyzed cross-benzoin reaction, where the reaction proceeds *via* the generation of the nucleophilic Breslow intermediate A (Scheme 1, eqn (1)).^9–11^ Moreover, Chi and co-workers demonstrated the NHC-catalyzed DKR of α-alkyl α-aryl carboxylic esters *via* the transesterification strategy, and the NHC- enolate B is the key intermediate (eqn (2)).^12^ In all these cases, the α-carbon center is involved in the DKR process, where the generated chiral center is proximal to the reacting center (generation of D from C), and intriguingly, the synthesis of enantioenriched γ-substituted carboxylic esters from racemic starting materials *via* the DKR process is not known.^13^ This will be interesting as the γ-carbon center will be remote from the reacting carbonyl center and enantioinduction will be challenging (conversion of E to F). In this context, we envisioned the NHC-catalyzed DKR of the γ,γ-disubstituted aldehyde G derived from the unprotected indole-2-carboxaldehyde,^14^ which can be generated *in situ* by the Lewis acid-catalyzed Friedel–Crafts reaction of indole 2-aldehyde 1a with the *o*-hydroxyphenyl-substituted *p*-quinone methide 2a. This formal [4 + 3] annulation reaction afforded indole-fused ε-lactone 3a in good yields and selectivities. The optimal Lewis acid was Bi(OTf)_3_, which plays dual roles: (a) in catalyzing the initial Friedel–Crafts reaction generating G, and (b) then the involvement in the DKR process for the esterification reaction in cooperation with NHCs.^15^ Intriguingly, although NHC-catalyzed DKR strategies are known for the enantioselective synthesis of β-lactones, γ-lactones and δ-lactones, the related DKR strategies for ε-lactones are unknown. It may be noted in this context that NHC-catalyzed synthesis of fused ε-lactones by the [4 + 3] annulation of *o*-quinone methides with enal-derived homoenolates was uncovered independently by Ye’s^16^ and Scheidt’s groups.^17^ Moreover, a related NHC-homoenolate route for the synthesis of spirooxindole ε-lactones (without involving the DKR process) is demonstrated by Li’s^18*a*^ and Enders’ groups.^18*b*^

## Results and discussion

Driven by the idea of inducing stereocontrol at a remote position using the DKR strategy, the present study was initiated by treating indole 2-carboxaldehyde 1a with the *p*-quinone methide 2a in the presence of NHC generated from the chiral triazolium salt 4 using Cs_2_CO_3_ as the base under oxidative conditions using the bisquinone 8. Interestingly, under these conditions, the desired indole-fused ε-lactone 3a was formed in 68% yield and a 95 : 5 enantiomeric ratio (er) (Table 1, entry 1). The product 3a was formed by the initial Friedel–Crafts reaction of 1a with 2a catalyzed by Bi(OTf)_3_ (generating *in situ*3a''), followed by the NHC/Lewis acid-catalyzed DKR *via* a stereoselective esterification reaction. Notably, the ester 3a′ (formed by the esterification of 1a with the phenol moiety of 2a),^19^ and the Friedel–Crafts product 3a′′ were not isolated under these conditions. Moreover, compared to the carbene formed from 4, other chiral triazolium salts 5–7 provided less yield and selectivity of 3a (entries 2–4). The screening of other bases and solvents revealed that Cs_2_CO_3_ is the optimal base and toluene is the best solvent for this transformation (entries 5–10). The use of Sc(OTf)_3_ as the Lewis acid and CF_3_SO_3_H as the Brønsted acid for initiating the Friedel–Crafts reaction was also not efficient (entries 11 and 12). In addition, performing the reaction with 10 mol% of 4 or using 1.0 equiv. of 8 resulted in an incomplete reaction with the isolation of the Friedel–Crafts adduct 3a′′ maintaining high selectivity (entries 13,14). Hence, entry 1 was selected as the best condition for the substrate scope analysis.^20^

**Table 1:** Optimization of the reaction conditions^a^

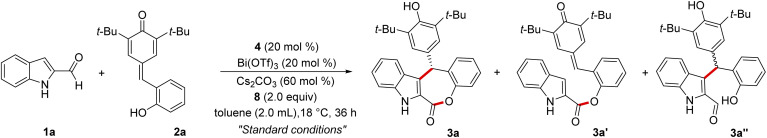					
entry	Variation of the standard conditions^a^	Yield of 3a^b^ (%)	er of 3a^c^	Yield of 3a′^b^ (%)	Yield of 3a′′^b^ (%)
1	None	68	95 : 5	<5	<5
2	5 Instead of 4	62	86 : 14	<5	<5
3	6 Instead of 4	11	86 : 14	<5	66
4	7 Instead of 4	19	81 : 19	<5	<5
5	K_2_CO_3_ instead of Cs_2_CO_3_	36	91 : 9	<5	25
6	KO*t*-Bu instead of Cs_2_CO_3_	<5	-Nd-	<5	<5
7	DABCO instead of Cs_2_CO_3_	<5	-Nd-	<5	<5
8	THF instead of toluene	<5	-Nd-	71	<5
9	DME instead of toluene	<5	-Nd-	67	<5
10	Mesitylene instead of toluene	22	91 : 9	<5	18
11	Sc(OTf)_3_ instead of Bi(OTf)_3_	52	92 : 8	<5	<5
12	CF_3_SO_3_H instead of Bi(OTf)_3_	60	91 : 9	<5	<5
13	10 mol% of 4 instead of 20 mol%	31	95 : 5	<5	48
14	1.0 equiv. of 8 instead of 2.0 equiv.	39	94 : 6	<5	28
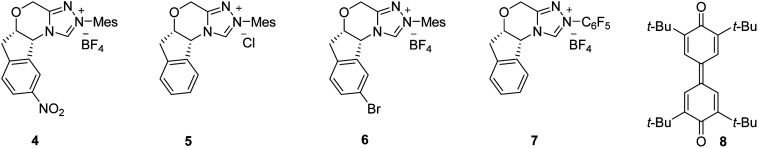					

a Standard conditions: 1a (0.12 mmol), 2a (0.168 mmol), 4 (20 mol%), Bi(OTf)_3_ (20 mol%), Cs_2_CO_3_ (60 mol%), 8 (2.0 equiv.), toluene (2.0 mL), 18 °C and 36 h.

b Yields of the column chromatography purified products are provided.

c The er was established by HPLC analysis on a chiral stationary phase.

**Scheme 2:**
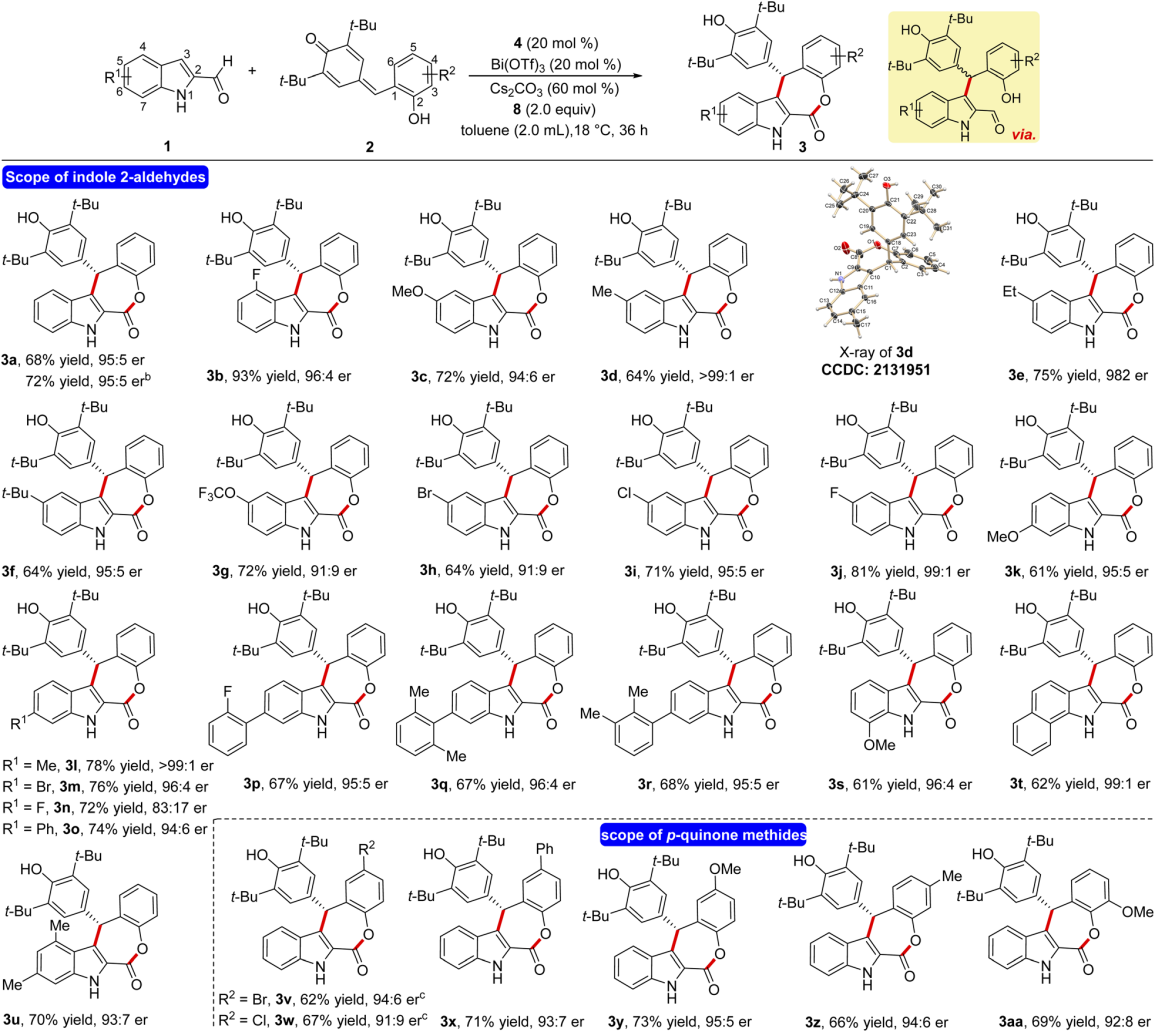
^a^ Reaction conditions: 1 (0.25 mmol), 2 (1.4 equiv.), 4 (20 mol%), Bi(OTf)_3_ (20 mol%), Cs_2_CO_3_ (60 mol%), 8 (2.0 equiv.), toluene (4.0 mL), 18 °C and 36 h. Given are isolated yields of the column chromatography purified products. The er was established by HPLC analysis on a chiral stationary phase. ^b^ The yield and er for a 1.0 mmol scale reaction. ^c^ The reaction performed at 10 °C for 48 h.

Having the optimized reaction conditions in hand, the scope and limitations of the present NHC-catalyzed DKR has been examined. First, the variation of the indole 2-carboxaldehyde has been studied. The unsubstituted parent aldehyde worked well and 4-fluoro substituted aldehyde furnished the tetracyclic ε-lactone 3b in 93% yield and 96 : 4 er (Scheme 2). The formation of 3a in 72% yield and 95 : 5 er on a 1.0 mmol scale indicates that the present DKR process is scalable and practical. A variety of electronically different substituents at the 5-position of indole 2-carboxaldehyde was well tolerated under the optimized conditions and the corresponding ε-lactones were formed in good yields and selectivities (3c–3j). In the case of the methyl derivative 3d, the structure and the absolute stereochemistry of the chiral center were confirmed using X-ray analysis of the crystals.^21^ Moreover, substrates bearing different groups at the 6-position of indole 2-carboxaldehye underwent a smooth NHC-catalyzed annulation reaction to afford the desired products in good yields and er values (3k–3r). In addition, the reaction using 7-methoxy indole 2-carboxaldehyde furnished the product 3s in 61% yield and 96 : 4 er. Furthermore, disubstituted indole -aldehydes also provided good yield of the target product thus expanding the scope of this annulation (3t and 3u).

Next, the variation in the *o*-hydroxyphenyl-substituted *p*-quinone methide moiety was studied. The *p*-quinone methides having –Br, –Cl, Ph and –OMe groups at the 5-position are well tolerated under the present conditions and the desired annulated products are formed in reasonable yields and selectivities (3v–3y). Moreover, –Me and –OMe groups at the 4- and 3-position of 2 did not affect the reaction outcome and the target ε-lactones are formed in good yields and er values (3z and 3aa).

**Scheme 3:**
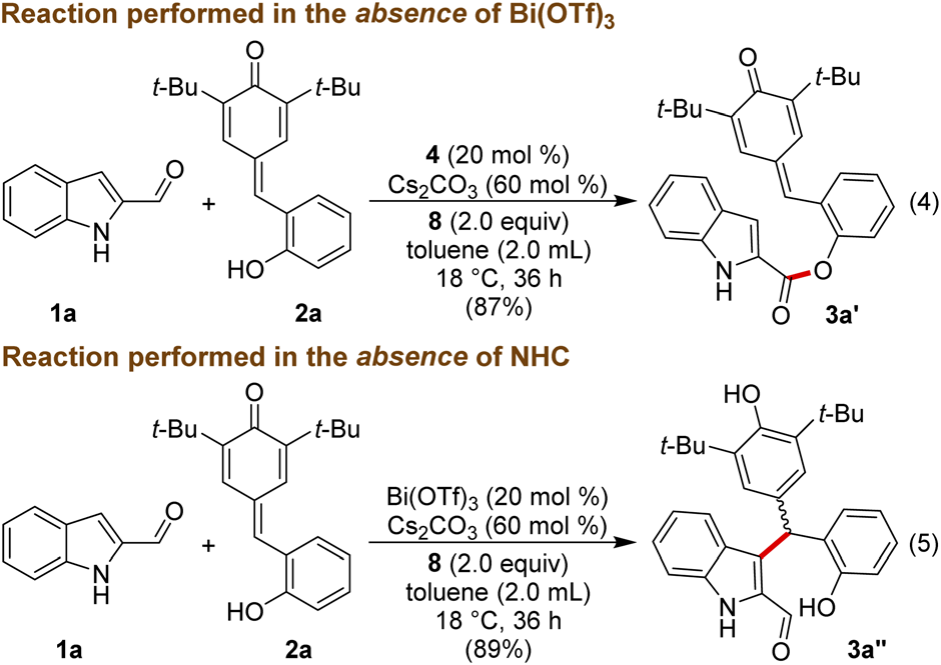
Control experiments.

To get insight into the mechanism of the reaction, a few mechanistic experiments were performed. When the reaction of 1a was performed with 2a in the absence of Bi(OTf)_3_, the reaction furnished the ester product 3a′ in 87% yield, and 3a was not formed under these conditions (Scheme 3, eqn (4)). Notably, related esterification reactions catalyzed by NHCs are reported by Studer and co-workers.^19^ Moreover, treatment of 1a with 2a in the absence of NHC resulted in the formation of the Friedel–Crafts adduct 3a′′ in 89% yield (eqn (5)).

**Scheme 4:**
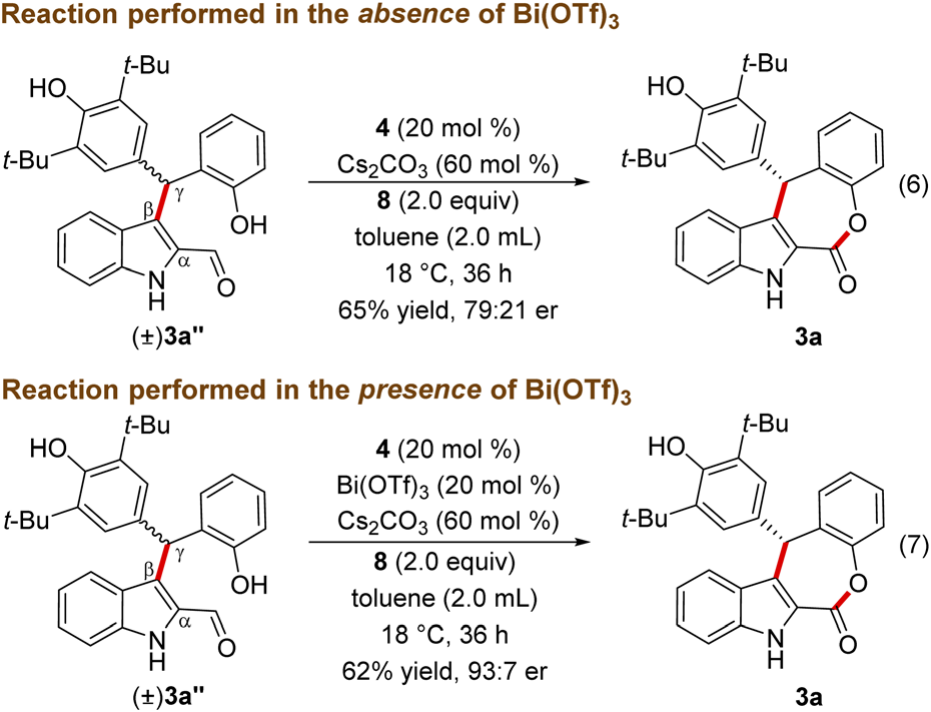
Role of a Lewis acid in the DKR process.

The lack of the desired product 3a formation in the absence of either Bi(OTf)_3_ or NHC indicates the role of these two catalysts for the direct and enantioselective synthesis of the ε-lactone 3a. To get further insight into the role of Bi(OTf)_3_ in the DKR process, the Friedel–Crafts alkylation product 3a′′ was treated with NHC generated from 4 under oxidative conditions in the absence of Bi(OTf)_3_. This reaction afforded 3a in 65% yield and 79 : 21 er (Scheme 4, eqn (6)). Interestingly, when the same reaction was conducted in the presence of Bi(OTf)_3_ the product 3a was formed in 62% yield and an improved er of 93 : 7 shedding light on the role of a Lewis acid in the DKR process (eqn (7)).^22^ It is reasonable to assume that the Bi(iii) Lewis acid is involved in coordination with the NHC-bound dienolate and the phenolic –OH moiety for the facile dienolate protonation and intramolecular acylation.^23,24^

**Scheme 5:**
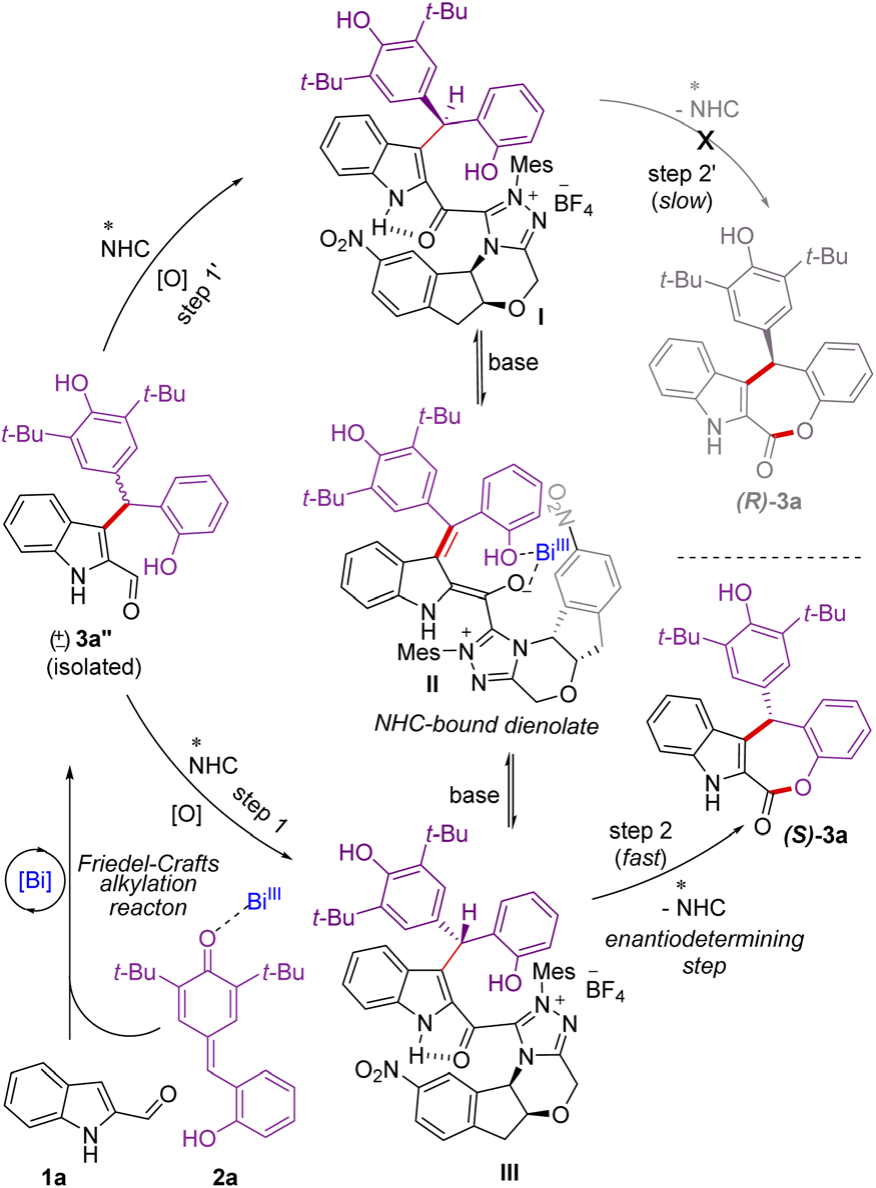
Proposed mechanism of the reaction.

Mechanistically, in the presence of Lewis acidic Bi(OTf)_3_, indole 2-carboxaldehyde 1a^25^ adds to the *p*-quinone methide 2a generating *in situ* the racemic γ,γ-disubstituted indole 2-carboxaldehyde 3a′′ through an intermolecular Friedel–Crafts alkylation reaction (Scheme 5). Under oxidative conditions, the addition of NHC to the aldehyde 3a′′ could generate the diastereomeric NHC-bound acylazoliums I and III.^26^ It is reasonable to assume that the NHC acylazolium I could not undergo intramolecular acylation due to the presence of a bulky chiral indanone core of the catalyst. Hence, the formation of (*R*)-3a is not feasible. On the other hand, the NHC acylazolium III undergoes facile intramolecular acylation to afford the desired product (*S*)-3a as the aminoindanol and the bulky 2,6-di-*tert*-butyl phenolic moieties are on the opposite side. The acylazolium I under basic conditions could form the NHC-dienolate intermediate II,^6*j*,27^ which could undergo enantioselective protonation to generate the intermediate III, which can further undergo acylation to form the product (*S*)-3a. During the re-protonation of NHC-bound dienolate intermediate II, Bi(OTf)_3_ is likely involved in the coordination with the dienolate and –OH moieties to facilitate protonation and then esterification.

In conclusion, we have presented the NHC-Lewis acid cooperative catalyzed DKR for the enantioselective synthesis of tetracyclic indole-fused ε-lactones, a formal [4 + 3] annulation. The transiently generated γ,γ-disubstituted indole 2-carboxaldehydes from indole-2-carboxaldehyde and 2-hydroxy phenyl *p*-quinone methides using Bi(OTf)_3_ catalysis underwent an efficient DKR process, where the NHC-bound dienolates are the key intermediates. In the presence of NHC and Bi(OTf)_3_, facile ε-lactonization takes place with enantioinduction at the γ-position. The tetracyclic ε-lactones are formed in up to 93% yield and >99 : 1 er. The stereoinduction at the remote γ-carbon, mild reaction conditions, and *in situ* generation of the racemic substrate for DKR are the notable features of the present annulation reaction.

## Data availability

Details of the experimental procedures, mechanistic experiments, characterization data of all the tetracyclic indole-fused ε-lactones, and X-ray data of 3d.

## Author contributions

K. B. and A. T. B. conceived and designed the project. K. B. performed the optimization studies, substrate scope analysis and mechanistic studies. S. B. and S. S. helped in the substrate scope studies. R. G. G. performed the X-ray crystallographic analysis. K. B. and A. T. B. wrote the manuscript. All authors have given approval to the final version of the manuscript.

## Conflicts of interest

There are no conflicts to declare.

## Supplementary Material

SC-013-D2SC03745A-s001

SC-013-D2SC03745A-s002
