# Supplementary material for: Dynamic kinetic resolution of γ,γ-disubstituted indole 2-carboxaldehydes via NHC-Lewis acid cooperative catalysis for the synthesis of tetracyclic ε-lactones
Source: Chem Sci. 2022 Aug 29;13(39):11513–8. doi: 10.1039/d2sc03745a (PMC9555563; doi:10.1039/d2sc03745a)
Supplement: SC-013-D2SC03745A-s001 [file SC-013-D2SC03745A-s001.pdf]

Electronic Supplementary Material (ESI) for Chemical Science.
This journal is © The Royal Society of Chemistry 2022

Dynamic kinetic resolution of γ,γ-disubstituted indole 2-carboxaldehydes via NHC-Lewis acid cooperative catalysis for the synthesis of tetracyclic *ε*-lactones

Kuruva Balanna,[†] Soumen Barik,[†] Sayan Shee,[†] Rajesh G. Gonnade,[‡] and Akkattu T. Biju*[,†]

[†] Department of Organic Chemistry, Indian Institute of Science, Bangalore-560012, India

[‡] Centre for Materials Characterization, CSIR-National Chemical Laboratory, Dr. Homi Bhabha Road, Pune-411008, India

E-mail: atbiju@iisc.ac.in

Supporting Information

1. General Information S2
2. General Procedure for the Optimization of the Reaction Conditions S3
3. General Procedure for the Enantioselective Synthesis of Tetracyclic *ε*-Lactones S5
4. General Procedure for the Racemic Synthesis of Tetracyclic *ε*-Lactones S6
5. X-ray Data of **3d** S6
6. Mechanistic Experiments S10
7. Synthesis and Characterization of Tetracyclic *ε*-Lactones S25
8. ^{1}H and ^{13}C NMR Spectra of Tetracyclic *ε*-Lactones S46
9. HPLC Data of Tetracyclic *ε*-Lactones S81

1. General Information

Unless otherwise specified, all reactions were carried out under an atmosphere of nitrogen in oven-dried reaction vessels with Teflon screw caps. 25 °C Corresponds to the room temperature (rt) of the lab when the experiments were performed. Dry toluene was purchased from commercial sources and stored under nitrogen over sodium wire, and dry $CHCl_3$ was purchased from commercial sources and stored under nitrogen over 4 Å molecular sieves. The 1*H*-indole-2-carbaldehyde **1a**, **1b-1u** were synthesised following the literature procedure.[1] The *p*-quinone methide **2a-2g** were synthesized by following the literature procedure.[2] The triazolium salt **4** was synthesized following the literature procedure.[3] The Kharasch oxidant **8** was synthesized following the literature procedure.[4] Cs_2CO_3 was purchased from Spectrochem and was activated before use. $Bi(OTf)_3$ and $Sc(OTf)_3$ were purchased from Sigma-Aldrich.

Analytical thin layer chromatography was performed on TLC Silica gel 60 F_{254}. Visualization was accomplished with short wave UV light or $KMnO_4$ staining solutions followed by heating. Flash chromatography was performed on silica gel (230-400 mesh) by standard techniques eluting with Pet. Ether-EtOAc solvent system.

All compounds were fully characterized. ^{1}H and ^{13}C NMR spectra were recorded on Bruker AV 400 and Bruker Ultrashield spectrometer in solvents as indicated. Chemical shifts (δ) are given in ppm. The residual solvent signals were used as references and the chemical shifts converted to the TMS scale ($CDCl_3$: δH = 7.26 ppm, δC = 77.16 ppm and Acetone-D_6: δH = 2.05 ppm, δC = 29.84, 206.26 ppm). Infrared (FT-IR) spectra were recorded on a Bruker Alfa FT-IR, ν-max in cm^{-1}. Optical rotations were measured on JASCO P-2000 Polarimeter at room temperature using 50 mm cell of 1.0 mL capacity. HRMS (ESI) data were recorded on a Waters Xevo G2-XS Q-TOF instrument. HPLC analysis was performed on Agilent Technologies 1260 Infinity II with a Variable Wavelength Detector.

---

[1] Y. Liu, G. Luo, X. Yang, S. Jiang, W. Xue, Y. R. Chi and Z. Jin, *Angew. Chem., Int. Ed.,* 2020, **59**, 442.
[2] J.-P. Tan, P. Yu, J.-H. Wu, Y. Chen, J. Pan, C. Jiang, X. Ren, H.-S. Zhang and T. Wang, *Org. Lett.*, 2019, **21**, 7298.
[3] C.-G. Zhao, F.-Y. Li and J. Wang, *Angew. Chem., Int. Ed.*, 2016, **55**, 1820.
[4] M. S. Kharasch and B. S. Joshi, *J. Org. Chem.*, 1957, **22**, 1439.

2. General Procedure for the Optimization of the Reaction Conditions

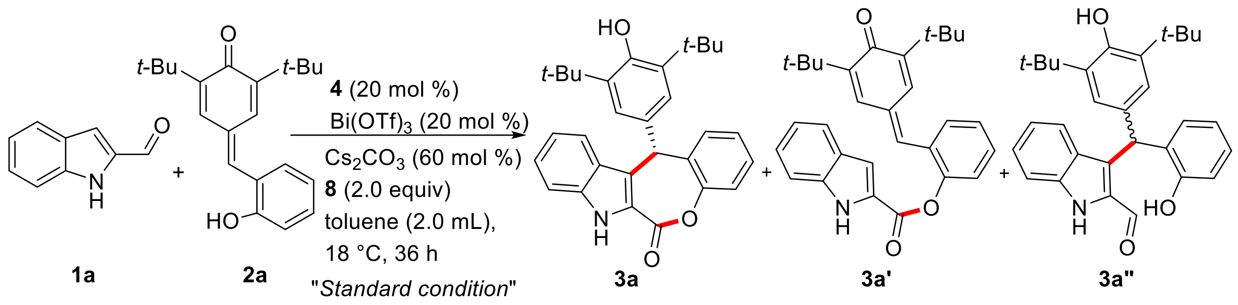

To an oven-dried Schlenk tube with a teflon screw cap was taken the triazolium salt **4** (5.6 mg, 0.012 mmol), Cs_2CO_3 (19.5 mg, 0.06 mmol) and $Bi(OTf)_3$ (15.6 mg, 0.024 mmol) inside a glove-box. Then 1*H*-indole-2-carbaldehyde **1a** (17.4 mg, 0.12 mmol), 2,6-di-*tert*-butyl-4-(2-hydroxybenzylidene)cyclohexa-2,5-dien-1-one **2a** (52.1 mg, 0.168 mmol) and oxidant **8** (49.0 mg, 0.12 mmol) were added outside the glove-box at 18 °C under nitrogen atmosphere followed by addition of toluene (1.0 mL). The reaction mixture was stirred at 18 °C for 12 h and then to the stirring solution, triazolium salt **4** (5.6 mg, 0.012 mmol), Cs_2CO_3 (4.0 mg, 0.012 mmol), and oxidant **8** (49.0 mg, 0.12 mmol) were added at 18 °C under nitrogen atmosphere followed by addition of toluene (1.0 mL). The reaction mixture was stirred at 18 °C for 24 h under nitrogen atmosphere. After the completion of the reaction, the solvent was evaporated, and the crude residue was purified by flash column chromatography on silica gel to afford the corresponding (*S*)-12-(3,5-di-*tert*-butyl-4-hydroxyphenyl)-7,12-dihydro-6*H*-benzo [6,7] oxepino [3,4-*b*] indol-6-one **3a.** The enantiomeric ratio was determined by HPLC analysis on a chiral stationary phase.

Optimization Studies

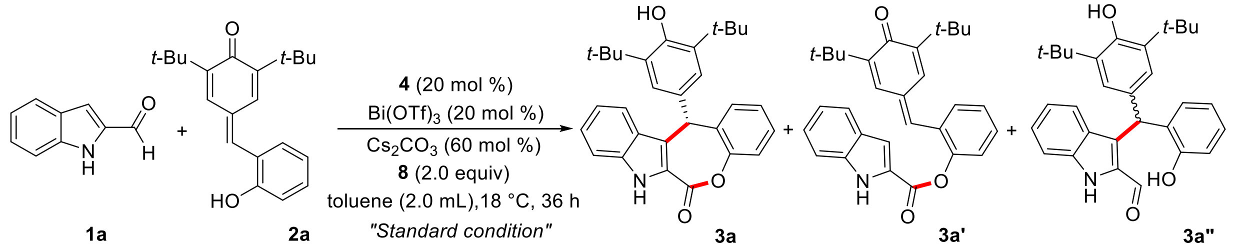

entry	Variation of the standard conditions[a]	Yield of **3a** (%)[b]	er of **3a**[c]	Yield of **3a'** (%)[b]	Yield of **3a"** (%)[b]
	Carbene Precursor Screening				
1	***none***	***69***	***95:5***	***<5***	***<5***
2	**5** instead of **4**	62	86:14	<5	<5
3	**6** instead of **4**	11	86:14	<5	66

4	**7** instead of **4**	19	81:19	<5	<5
Base Screening					
5	K_2CO_3 instead of Cs_2CO_3	36	91:9	<5	25
6	KO*t*-Bu instead of Cs_2CO_3	<5	-nd-	<5	<5
7	DBU instead of Cs_2CO_3	<5	-nd-	<5	<5
8	DABCO instead of Cs_2CO_3	<5	-nd-	<5	<5
9	DMAP instead of Cs_2CO_3	<5	-nd-	<5	<5
Acid Screening					
10	$Sc(OTf)_3$ instead of $Bi(OTf)_3$	52	92:8	<5	<5
11	$Sn(OTf)_3$ instead of $Bi(OTf)_3$	<5	-nd-	64	<5
12	$Mg(OTf)_2$ instead of $Bi(OTf)_3$	<5	-nd-	76	<5
13	$Eu(OTf)_3$ instead of $Bi(OTf)_3$	<5	-nd-	64	<5
14	Tl(OTf) instead of $Bi(OTf)_3$	<5	-nd-	72	<5
15	$AgNO_3$ instead of $Bi(OTf)_3$	<5	-nd-	88	<5
16	CF_3SO_3H instead of $Bi(OTf)_3$	60	91:9	<5	<5
Solvent Screening					
17	THF instead of toluene	<5	-nd-	71	<5
18	DME instead of toluene	<5	-nd-	67	<5
19	DMSO instead of toluene	<5	-nd-	<5	<5
20	DCM instead of toluene	31	89:11	<5	52
21	DCE instead of toluene	14	90:10	<5	75
22	Mesitylene instead of toluene	22	91:9	<5	18
23	Chlorobenzene instead of toluene	16	86:14	<5	68
24	Trifluorotoluene instead of toluene	15	86:14	<5	70
Temperature Screening					
25[d]	50 °C instead of 18 °C	79	86:14	<5	<5
26[d]	45 °C instead of 18 °C	63	88:12	<5	<5
27[d]	40 °C instead of 18 °C	52	90:10	<5	07
28[d]	30°C instead of 18 °C	37	91:9	<5	57
29[d]	0 °C instead of 18 °C	<5	-nd-	<5	<5
Stoichiometry variation					
30	10 mol % of **4** instead of 20 mol %	31	95:5	<5	48
31	15 mol % of **4** instead of 20 mol %	49	95:5	<5	22
32	25 mol % of **4** instead of 20 mol %	69	95:5	<5	<5
33	1.0 equiv of **8** instead of 2.0 equiv	39	94:6	<5	28
34	1.5 equiv of **8** instead of 2.0 equiv	58	94:6	<5	17
35	1.0 equiv of **2a** instead of 1.4 equiv	53	94:6	<5	<5
36	1.2 equiv of **2a** instead of 1.4 equiv	60	94:6	<5	<5
37	1.6 equiv of **2a** instead of 1.4 equiv	68	94:6	<5	<5
38	Reaction without **4**	<5	-nd-	<5	89
39	Reaction without $Bi(OTf)_3$	<5	-nd-	87	<5

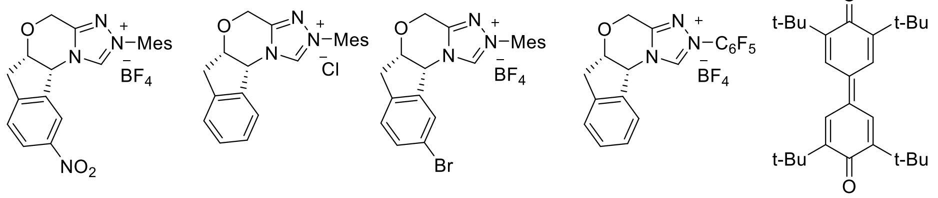

4 **5** **6** **7** **8**

[a] Standard conditions: **1a** (0.12 mmol), **2a** (0.168 mmol), **4** (20 mol %), $Bi(OTf)_3$ (20 mol %), Cs_2CO_3 (60 mol %), **8** (2.0 equiv), toluene (2.0 mL), 18 °C and 36 h. [b] Yield of the column chromatography purified products are provided. [c] The er was established by HPLC analysis on a chiral stationary column. [d] Reaction performed upto12 h.

3. General Procedure for the Enantioselective Synthesis of Tetracyclic *ε*-Lactones

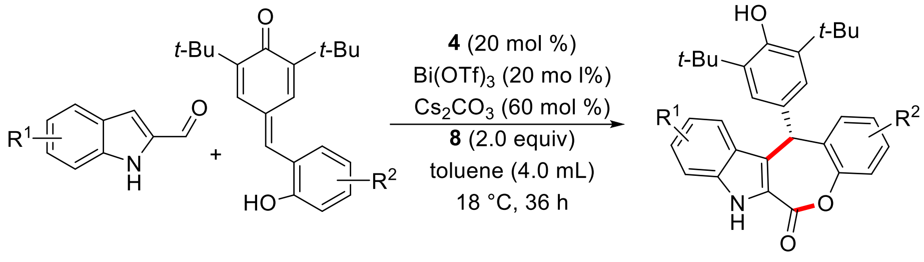

1 **2** **3**

To an oven-dried Schlenk tube with a teflon screw cap was taken the triazolium salt **4** (11.6 mg, 0.025 mmol), Cs_2CO_3 (41.0 mg, 0.125 mmol) and $Bi(OTf)_3$ (33.0 mg, 0.05 mmol) in a glove-box. Then 1*H*-indole-2-carbaldehyde derivative **1** (36.3 mg, 0.25 mmol), 2,6-di-*tert*-butyl-4-(2-hydroxybenzylidene)cyclohexa-2,5-dien-1-one derivative **2** (108.6 mg, 0.35 mmol) and oxidant **8** (102.0 mg, 0.25 mmol) were added outside the glove-box at 18 °C under nitrogen atmosphere followed by addition of toluene (2.0 mL). The reaction mixture was stirred at 18 °C for 12 h and then to the stirring solution, triazolium salt **4** (11.6 mg, 0.025 mmol), Cs_2CO_3 (8.0 mg, 0.025 mmol), and oxidant **8** (102.0 mg, 0.25 mmol) were added at 18 °C under nitrogen atmosphere followed by addition of toluene (2.0 mL). The reaction mixture was stirred at 18 °C for 24 h under nitrogen atmosphere, after the completion of the reaction solvent was evaporated, and the crude residue was purified by flash column chromatography on silica gel to afford the corresponding tetracyclic *ε*-lactones **3.**

Procedure for the 1 mmol scale experiment

To an oven-dried Schlenk tube with a teflon screw cap was taken the triazolium salt **4** (46.4 mg, 0.1 mmol), Cs_2CO_3 (163.0 mg, 0.5 mmol) and $Bi(OTf)_3$ (130.0 mg, 0.2 mmol) in a glove-box. Then 1*H*-indole-2-carbaldehyde **1a** (145.0 mg, 1.0 mmol), 2,6-di-*tert*-butyl-4-(2-hydroxybenzylidene)cyclohexa-2,5-dien-1-one **2a** (434.0 mg, 1.4 mmol) and oxidant **8** (408.0 mg, 1.0 mmol) were added outside the glove-box at 18 °C under nitrogen atmosphere followed by addition of toluene (4.0 mL). The reaction mixture was stirred at 18 °C for 12 h and then to the

stirring solution, triazolium salt **4** (46.4 mg, 0.1 mmol), Cs_2CO_3 (33.0 mg, 0.1 mmol), and oxidant **8** (408.0 mg, 1.0 mmol) were added at 18 °C under nitrogen atmosphere followed by addition of toluene (4.0 mL). The reaction mixture was stirred at 18 °C for 24 h under nitrogen atmosphere, after the completion of the reaction solvent was evaporated, and the crude residue was purified by flash column chromatography on silica gel to afford the corresponding (*S*)-12-(3,5-di-*tert*-butyl-4-hydroxyphenyl)-7,12-dihydro-6*H*-benzo [6,7]oxepino[3,4-*b*]indol-6-one **3a** as a white solid (328.0 mg, 72 % yield with 95:5 er).

4. General Procedure for the Racemic Synthesis of Tetracyclic ε-Lactones

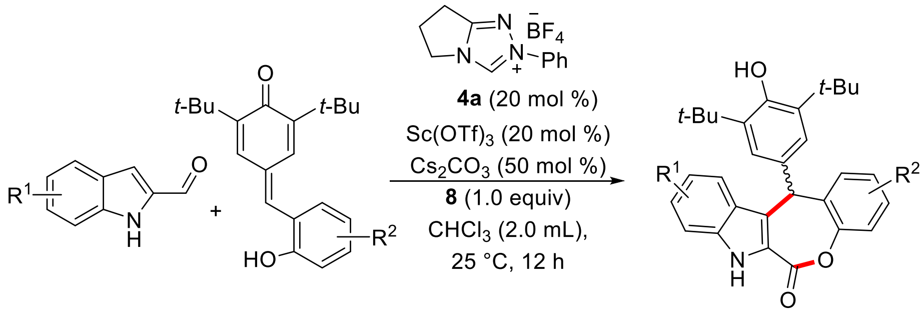

1 **2** **3**

To an oven-dried Schlenk tube with a teflon screw cap was taken the triazolium salt **4a** (6.5 mg, 0.024 mmol), Cs_2CO_3 (19.5 mg, 0.06 mmol) and $Sc(OTf)_3$ (11.8 mg, 0.024 mmol) in a glove-box. Then 1*H*-indole-2-carbaldehyde derivative **1** (17.4 mg, 0.12 mmol), 2,6-di-*tert*-butyl-4-(2-hydroxybenzylidene)cyclohexa-2,5-dien-1-one derivative **2** (52.1 mg, 0.168 mmol) and oxidant **8** (49.0 mg, 0.12 mmol) were added outside the glove-box at 25 °C under nitrogen atmosphere followed by addition of $CHCl_3$ (1.0 mL). The reaction mixture was stirred at 25 °C for 12 h under nitrogen atmosphere, after the completion of the reaction solvent was evaporated, and the crude residue was purified by flash column chromatography on silica gel to afford the corresponding tetracyclic ε-lactones **3.**

5. X-ray Data of 3d

X-ray intensity data measurements of compound **3d** was carried out on a Bruker D8 VENTURE Kappa Duo PHOTON II CPAD diffractometer equipped with Incoatec multilayer mirrors optics. The intensity measurements were carried out with Cu micro-focus sealed tube diffraction source (CuK_α= 1.54178 Å) at 100(2) K temperature. The X-ray generator was

operated at 50 kV and 1.1 mA. A preliminary set of cell constants and an orientation matrix were calculated from two sets of 40 frames. Data were collected with ω scan width of 0.5° at different settings of φ and 2θ with a frame time of 15 secs keeping the sample-to-detector distance fixed at 5.00 cm. The X-ray data collection was monitored by APEX3 program (Bruker, 2016).[5] All the data were corrected for Lorentzian, polarization and absorption effects using SAINT[6] and SADABS programs (Bruker, 2016). Using APEX3 (Bruker) program suite, the structure was solved with the ShelXS-97[7] (Sheldrick, 2008) structure solution program, using direct methods. The model was refined with version of ShelXL-2014[8] (Sheldrick, 2014) using Least Squares minimisation. All the hydrogen atoms were placed in a geomerically idalized positions and constrained to ride on its parent atoms except H-atom attached to the hydroxy group of ethanol solvent molecule. The H-atom bound to the –OH group of ethanl solvent molecule has been located in the difference Fourier and refined isotropically. An *ORTEP* III[9] view of compound was drawn with 50% probability displacement ellipsoids and H atoms are shown as small spheres of arbitrary radii. The absolute configuration was established by anomalous dispersion effect (Flack parameter, 0.1(3)) in X-ray diffraction measurements carried out with Cu radiation. The single crystal X-ray diffraction data analysis clearly established that our synthesize compound has *S* configurations at C1 position.

Compound **3d** having molecular formula $C_{33}H_{39}NO_4$ with approximate crystal dimensions 0.050 mm $\times$ 0.120 mm $\times$ 0.140 mm, was used for the X-ray crystallographic analysis. The X-ray intensity data were measured (λ = 1.54178 Å). The integration of the data using a monoclinic unit cell yielded a total of 28158 reflections to a maximum θ angle of 72.24° (0.81 Å resolution), of which 5575 were independent (average redundancy 5.051, completeness = 99.3%, R_{int} = 4.82%, R_{sig} = 4.22%) and 5497 (98.60%) were greater than $2\sigma(F^2)$. The final cell constants of $\underline{a}$ = 9.2800(10) Å, $\underline{b}$ = 17.0143(19) Å, $\underline{c}$ =10.0461(10) Å, β = 115.351(5)°, volume = 1433.5(3) Å^3, are based upon the refinement of the XYZ-centroids of reflections above 20 $\sigma(I)$. The calculated minimum and maximum transmission coefficients (based on crystal size)

[5] APEX3 suite for crystallographic software, Bruker axs, Madison, WI (Bruker (2016).
[6] SAINT - Software for the Integration of CCD Detector System Bruker Analytical X-ray Systems, Bruker axs, Madison, WI (Bruker, 2006 and Bruker, 2016)
[7] G. M. Sheldrick, A short history of ShelX, Acta Cryst., 2008, **A64**, 339.
[8] T. Gruene, H. W. Hahn, A. V. Luebben, F. Meilleur and G. M. Sheldrick, *J. Appl. Cryst.* 2014, **47**, 462.
[9] L. J. Farrugia, *J. Appl. Cryst.* 1997, **30**, 565.

are 0.6366 and 0.7536. The structure was solved and refined using the Bruker SHELXTL Software Package, using the Sohnke (chiral) space group *P*2(1), with *Z* = 2 for the formula unit, $C_{33}H_{39}NO_4$. The final anisotropic full-matrix least-squares refinement on F^2 with 357 variables converged at *R*1 = 4.33%, for the observed data and *wR*2 = 10.73% for all data. The goodness-of-fit was 1.066. The largest peak in the final difference electron density synthesis was 0.478 $e^-/Å^3$ and the largest hole was -0.460 $e^-/Å^3$ with an RMS deviation of 0.047 $e^-/Å^3$. On the basis of the final model, the calculated density was 1.190 g/cm^3 and *F*(000), 552 e^-.

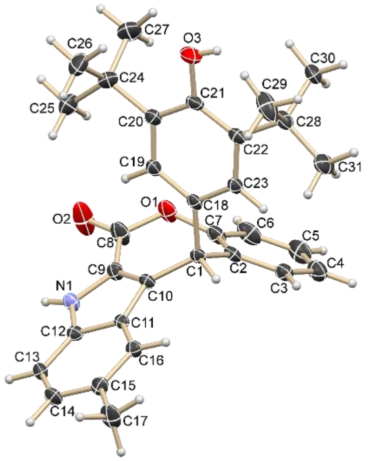

Figure 1. ORTEP view of compound **3d** showing the atom-numbering scheme. The displacement ellipsoids are drawn at the 50% probability level and H atoms are shown as small spheres with arbitrary radii. The solvent ethanol molecule is omitted for clarity.

Sample and crystal data for 3d.

Identification code	**3d**
CCDC	2131951
Chemical formula	$C_{33}H_{39}NO_4$
Formula weight	513.65 g/mol
Temperature	100(2) K
Wavelength	1.54178 Å
Crystal size	0.050 × 0.120 × 0.140 mm
Crystal system	monoclinic
Space group	P2(1)

Unit cell dimensions	*a* = 9.2800(10) Å	*α* = 90°
	b = 17.0143(19) Å	*β* = 115.351(5)°
	c = 10.0461(10) Å	*γ* = 90°
Volume	1433.5(3) $Å^3$	
Z	2	
Density (calculated)	1.190 g/cm^3	
Absorption coefficient	0.611 mm^{-1}	
F(000)	552	

Data collection and structure refinement for 3d.

Theta range for data collection	4.87 to 72.24°	
Index ranges	-11<=h<=11, -20<=k<=21, -12<=l<=12	
Reflections collected	28158	
Independent reflections	5575 [R(int) = 0.0482]	
Max. and min. transmission	0.7536 and 0.6366	
Structure solution technique	direct methods	
Structure solution program	SHELXS-97 (Sheldrick 2008)	
Refinement method	Full-matrix least-squares on F^2	
Refinement program	SHELXL-2014/7 (Sheldrick, 2014)	
Function minimized	$\Sigma\ w(F_o^2 - F_c^2)^2$	
Data / restraints / parameters	5575 / 1 / 357	
Goodness-of-fit on F^2	1.066	
Final R indices	5497 data; $I>2\sigma(I)$	R1 = 0.0433, wR2 = 0.1068
	all data	R1 = 0.0438, wR2 = 0.1073
Weighting scheme	$w=1/[\sigma^2(F_o^2)+(0.0408P)^2+0.6926P]$ where $P=(F_o^2+2F_c^2)/3$	
Absolute structure parameter	0.1(3)	
Extinction coefficient	0.0121(15)	
Largest diff. peak and hole	0.478 and -0.460 $eÅ^{-3}$	
R.M.S. deviation from mean	0.047 $eÅ^{-3}$	

6. Mechanistic Experiments

(a) Reaction performed in the absence of Bi(OTf)$_3$

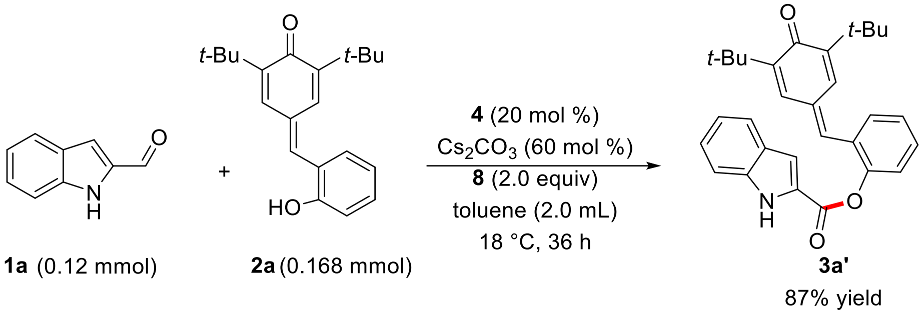

To an oven-dried Schlenk tube with a teflon screw cap was taken the triazolium salt **4** (5.6 mg, 0.012 mmol), and Cs_2CO_3 (19.5 mg, 0.06 mmol)) in a glove-box. Then 1*H*-indole-2-carbaldehyde **1a** (17.4 mg, 0.12 mmol), 2,6-di-*tert*-butyl-4-(2-hydroxybenzylidene)cyclohexa-2,5-dien-1-one **2a** (52.1 mg, 0.168 mmol) and oxidant **8** (49.0 mg, 0.12 mmol) were added outside the glove-box under nitrogen atmosphere followed by addition of toluene (1.0 mL). The reaction mixture was stirred at 18 °C for 12 h and then to the stirring solution, Cs_2CO_3 (3.9 mg, 0.012 mmol), triazolium salt **4** (5.6 mg, 0.012 mmol) and oxidant **8** (49.0 mg, 0.12 mmol) were added again under nitrogen atmosphere followed by addition of toluene (2.0 mL). The reaction mixture was stirred at 18 °C for 24 h under nitrogen atmosphere. After the completion of the reaction, the solvent was evaporated and the crude residue preadsorbed on silica gel and purified by flash column chromatography on silica gel to afford 2-((3,5-di-*tert*-butyl-4-oxocyclohexa-2,5-dien-1-ylidene)methyl)phenyl 1*H*-indole-2-carboxylate **3a’** as a orange solid (47.5 mg, 87% yield).

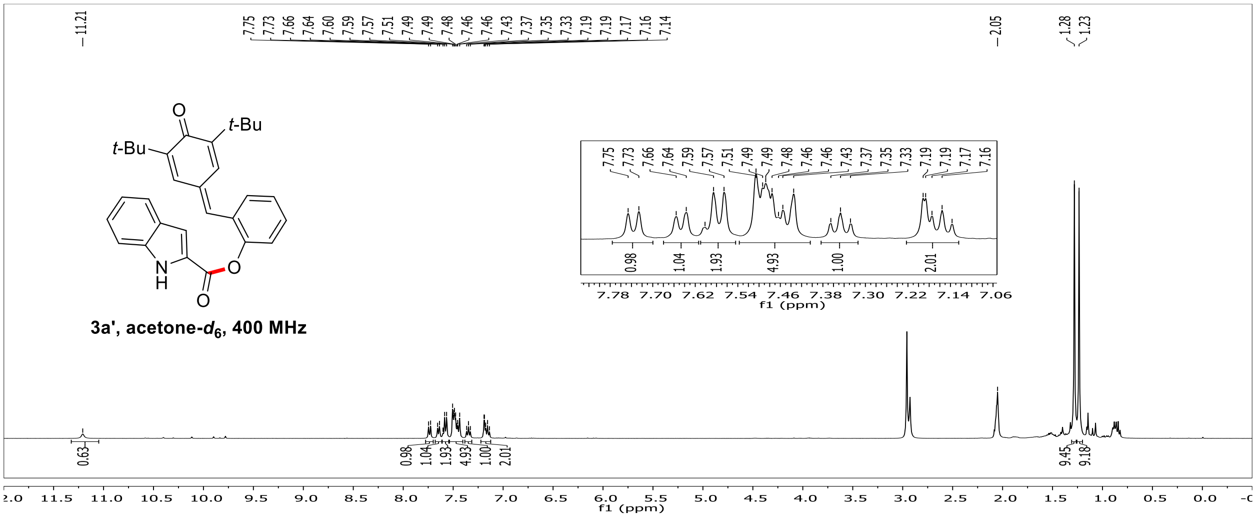

This experiment indicates the role of Bi(OTf)$_3$ in the Friedel-Crafts alkylation step.

(b) Reaction performed in the absence of NHC

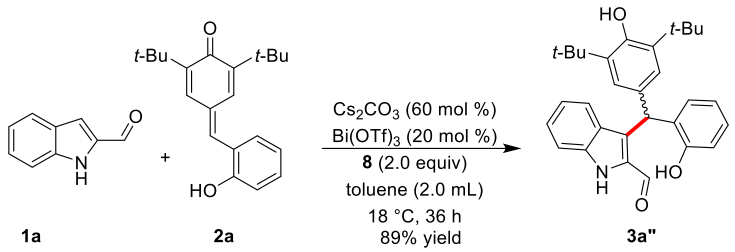

To an oven-dried Schlenk tube with a teflon screw cap was taken the Cs_2CO_3 (19.5 mg, 0.06 mmol) and $Bi(OTf)_3$ (15.6 mg, 0.024 mmol) in a glove-box. Then 1*H*-indole-2-carbaldehyde **1a** (17.4 mg, 0.12 mmol), 2,6-di-*tert*-butyl-4-(2-hydroxybenzylidene)cyclohexa-2,5-dien-1-one **2a** (52.1 mg, 0.168 mmol) and oxidant **8** (49.0 mg, 0.12 mmol) were added outside the glove-box under nitrogen atmosphere followed by addition of toluene (1.0 mL). The reaction mixture was stirred at 18 °C for 12 h and then to the stirring solution, Cs_2CO_3 (3.9 mg, 0.012 mmol), and oxidant **8** (49.0 mg, 0.12 mmol) were added again under nitrogen atmosphere followed by addition of toluene (2.0 mL). The reaction mixture was stirred at 18 °C for 24 h under nitrogen atmosphere. After the completion of the reaction, the solvent was evaporated to obtain the crude product, which was analyzed using ^{1}H NMR using CH_2Br_2 (8.5 µL, 0.12 mmol) as the internal standard. ^{1}H NMR analysis shows that the crude mixture contains the expected product **3a”** in 90% yield as well as the crude residue was further purified by flash column chromatography on silica gel to afford the corresponding 3-((3,5-di-*tert*-butyl-4-hydroxyphenyl)(2-hydroxyphenyl)methyl)-1*H*-indole-2-carbaldehyde **3a”** as a white solid (48.5 mg, 89% yield).

^{1}H-NMR Spectrum of 3-((3,5-di-*tert*-butyl-4-hydroxyphenyl)(2-hydroxyphenyl)methyl)-1*H*-indole-2-carbaldehyde 3a”

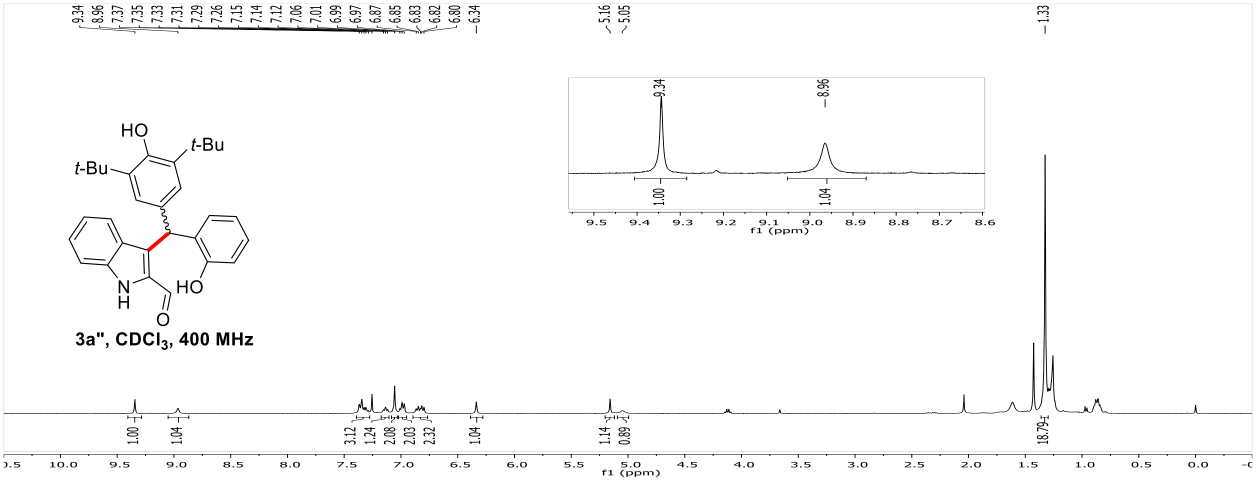

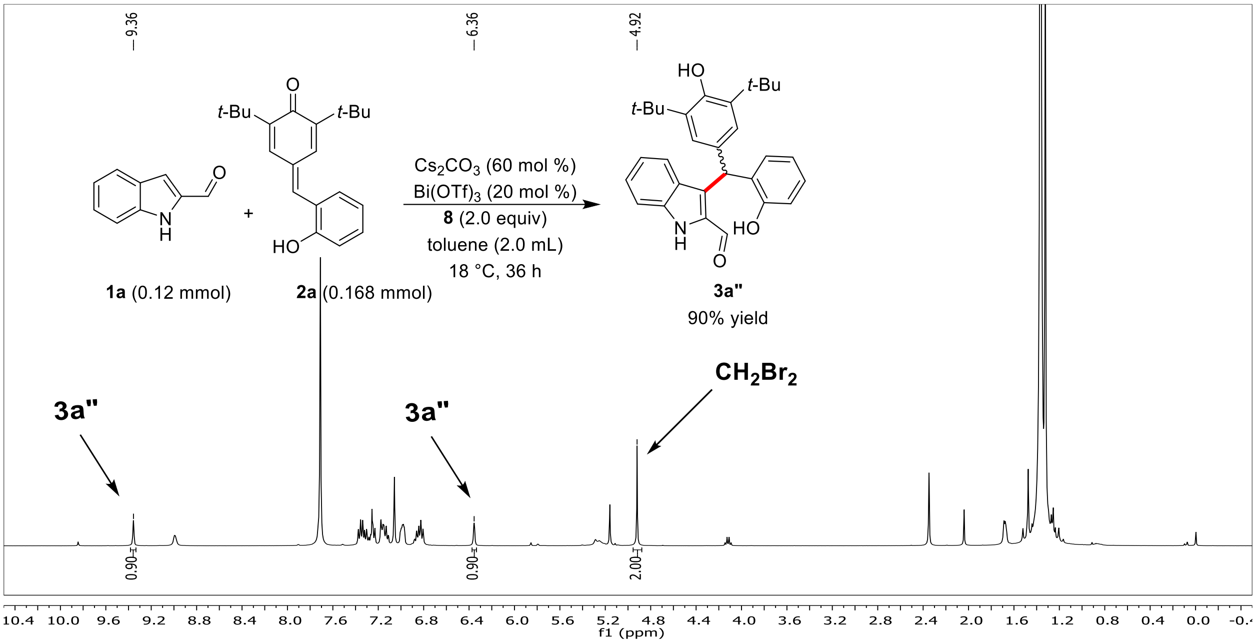

3-((3,5-di-*tert*-butyl-4-hydroxyphenyl)(2-hydroxyphenyl)methyl)-1*H*-indole-2-carbaldehyde (3a”)

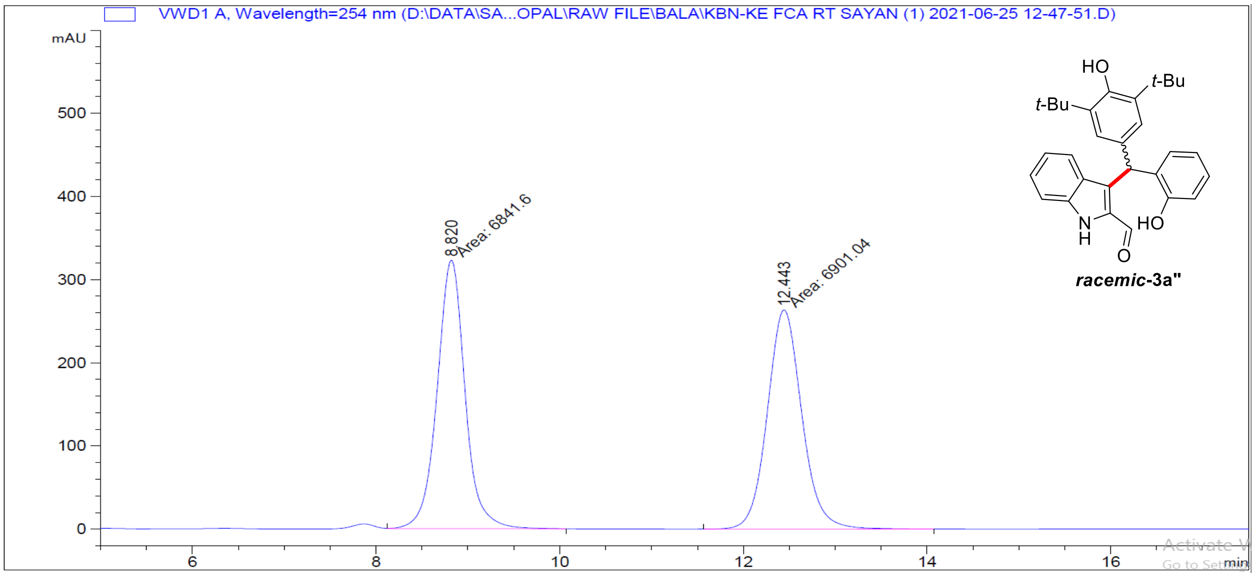

Peak #	RetTime [min]	Type	Width [min]	Area [mAU*s]	Height [mAU]	Area %
1	8.820	MM	0.3533	6841.59912	322.76346	49.7837
2	12.443	MM	0.4365	6901.03857	263.52148	50.2163

Sample Info : CHIRALPAK IA, 15 % IPA-HEXANE, 1.0 mL/min, 254 nM.M

The above result indicates the role of NHC for the formation of the desired ε-lactone product.

(c) Reaction performed in the absence of **Bi(OTf)₃**

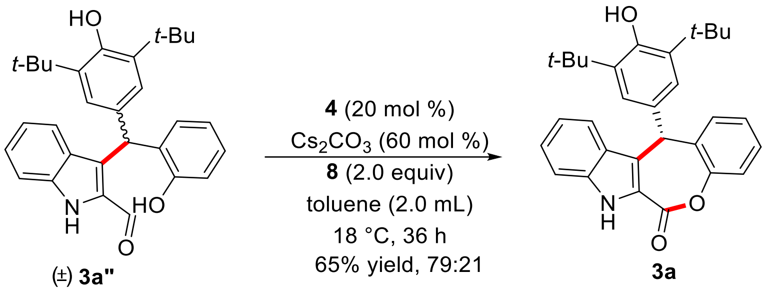

To an oven-dried Schlenk tube with a teflon screw cap was taken the triazolium salt **4** (5.6 mg, 0.012 mmol) and Cs_2CO_3 (19.5 mg, 0.06 mmol) in a glove-box. Then 3-((3,5-di-*tert*-butyl-4-hydroxyphenyl)(2-hydroxyphenyl)methyl)-1*H*-indole-2-carbaldehyde **3a"** (54.6 mg, 0.12 mmol), and oxidant **8** (49.0 mg, 0.12 mmol) were added outside the glove-box under nitrogen atmosphere followed by addition of toluene (1.0 mL). The reaction mixture was stirred at 18 °C for 12 h and then to the stirring solution, triazolium salt **4** (5.6 mg, 0.012 mmol), Cs_2CO_3 (3.9 mg, 0.012 mmol), and oxidant **8** (49.0 mg, 0.12 mmol) were added again under nitrogen atmosphere followed by addition of toluene (1.0 mL). The reaction mixture was stirred at 18 °C for 24 h under nitrogen atmosphere. After the completion of the reaction, the solvent was evaporated to obtain the crude product, which was analyzed using ^{1}H NMR using CH_2Br_2 (17.0 µL, 0.24 mmol) as the internal standard. ^{1}H NMR analysis shows that the crude mixture contains the expected product **3a** in 65% yield as well as the crude residue was further purified by flash column chromatography on silica gel to afford the corresponding (*S*)-12-(3,5-di-*tert*-butyl-4-hydroxyphenyl)-7,12-dihydro-6*H*-benzo [6,7] oxepino [3,4-*b*] indol-6-one **3a** as a white solid (35.0 mg, 65 % yield with 79:21 er).

^{1}H-NMR Spectrum of (*S*)-12-(3,5-di-*tert*-butyl-4-hydroxyphenyl)-7,12-dihydro-6*H*-benzo [6,7] oxepino [3,4-*b*] indol-6-one (3a)

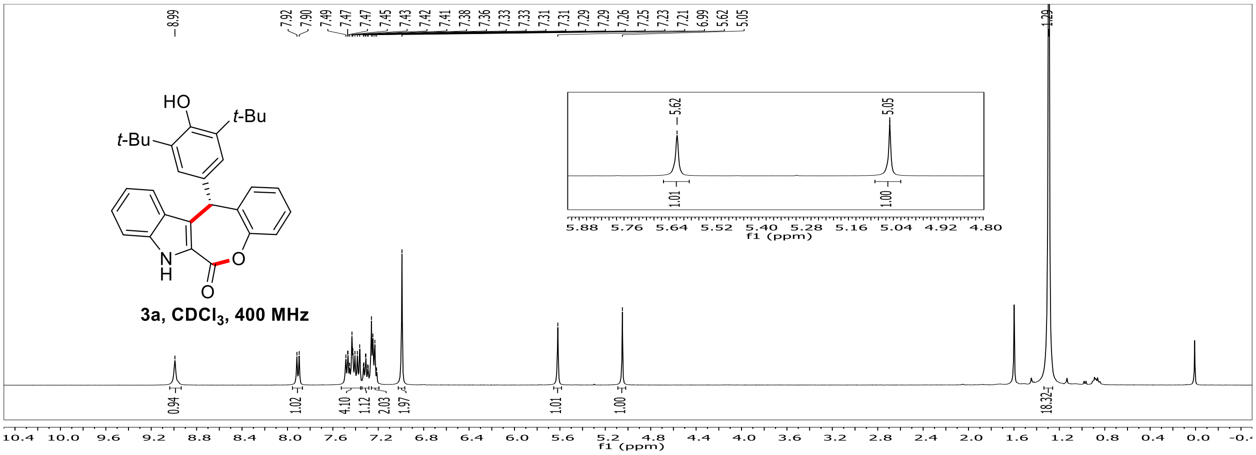

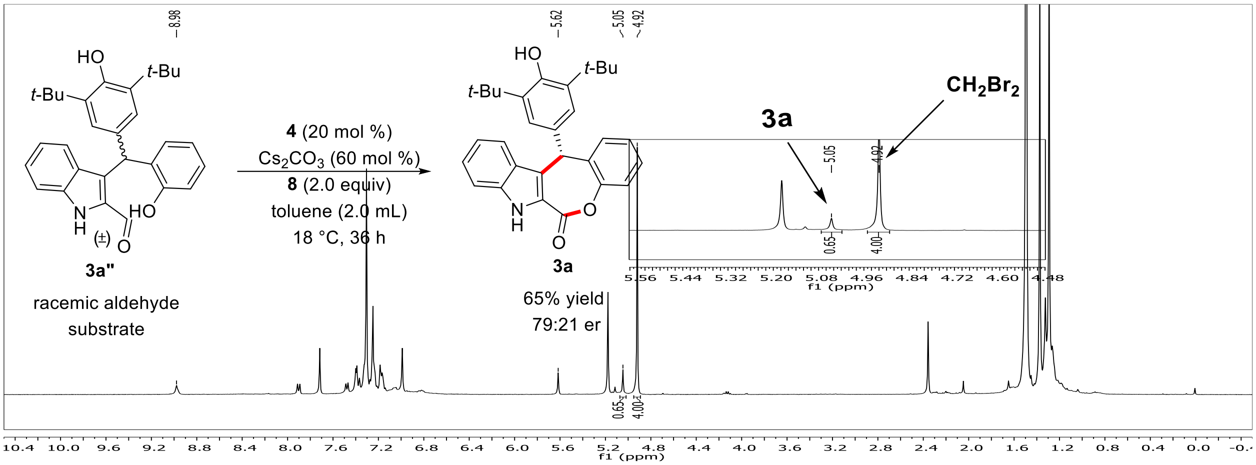

(*S*)-12-(3, 5-Di-*tert*-butyl-4-hydroxyphenyl)-7, 12-dihydro-6*H*-benzo [6, 7] oxepino [3, 4-*b*] indol-6-one (3a)

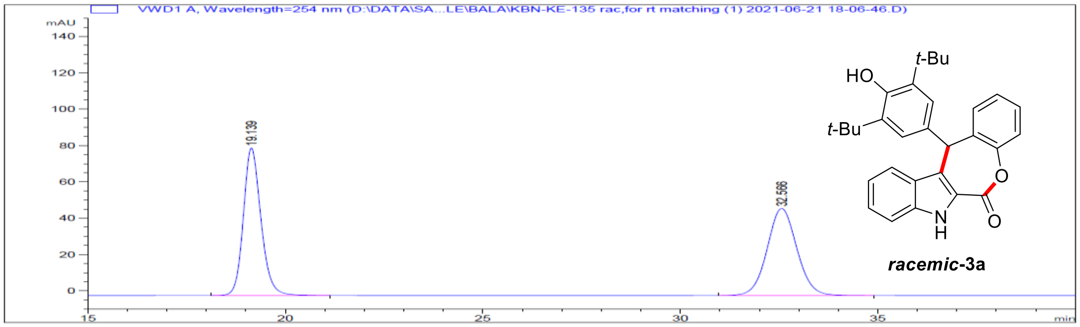

Peak #	RetTime [min]	Type	Width [min]	Area [mAU*s]	Height [mAU]	Area %
1	19.139	BB	0.4718	2500.86304	81.08739	50.0304
2	32.566	BB	0.8066	2497.81885	47.80070	49.9696

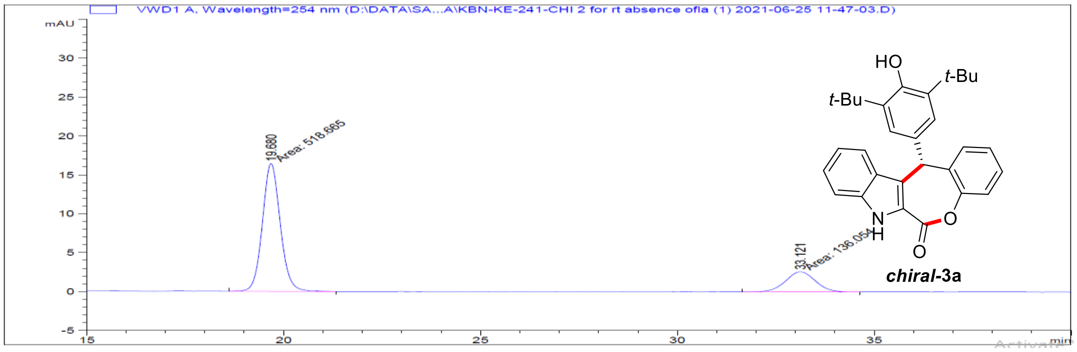

Peak #	RetTime [min]	Type	Width [min]	Area [mAU*s]	Height [mAU]	Area %
1	19.680	MM	0.5263	518.66522	16.42613	79.2195
2	33.121	MM	0.8778	136.05414	2.58312	20.7805

Sample Info : CHIRALPAK IA, 10 % IPA-HEXANE, 1.0 mL/min, 254 nM.M

The above result indicates the role of Lewis acid in the present DKR process.

(d) Reaction performed in the presence of Bi(OTf)$_3$

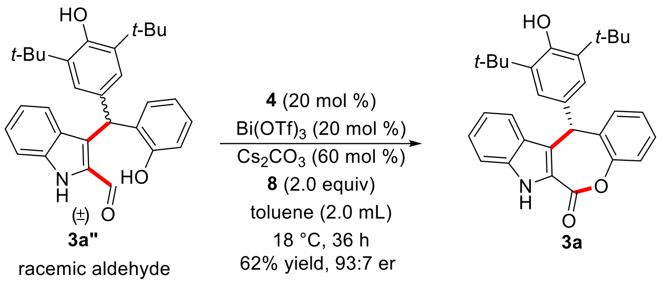

To an oven-dried Schlenk tube with a teflon screw cap was taken the triazolium salt **4** (5.6 mg, 0.012 mmol), Cs_2CO_3 (19.5 mg, 0.06 mmol) and Bi(OTf)$_3$ (15.6 mg, 0.024 mmol) in a glove-box. Then 3-((3,5-di-*tert*-butyl-4-hydroxyphenyl)(2-hydroxyphenyl)methyl)-1*H*-indole-2-carbaldehyde **3a"** (54.6 mg, 0.12 mmol), and oxidant **8** (49.0 mg, 0.12 mmol) were added outside the glove-box under nitrogen atmosphere followed by addition of toluene (1.0 mL). The reaction mixture was stirred at 18 °C for 12 h and then to the stirring solution, triazolium salt **4** (5.6 mg, 0.012 mmol), Cs_2CO_3 (3.9 mg, 0.012 mmol), and oxidant **8**(49.0 mg, 0.12 mmol) were added again under nitrogen atmosphere followed by addition of toluene (1.0 mL). The reaction mixture was stirred at 18 °C for 24 h under nitrogen atmosphere. After the completion of the reaction, the solvent was evaporated to obtain the crude product, which was analyzed using ^{1}H NMR using CH_2Br_2 (8.5 µL, 0.12 mmol) as the internal standard. ^{1}H NMR analysis shows that the crude mixture contains the expected product **3a** in 62% yield as well as the crude residue was further purified by flash column chromatography on silica gel to afford the corresponding (*S*)-12-(3,5-di-*tert*-butyl-4-hydroxyphenyl)-7,12-dihydro-6*H*-benzo [6,7] oxepino [3,4-*b*] indol-6-one **3a** as a white solid (33.5 mg, 62 % yield with 93:7 er).

^{1}H-NMR Spectrum of (*S*)-12-(3,5-di-*tert*-butyl-4-hydroxyphenyl)-7,12-dihydro-6*H*-benzo[6,7]oxepino[3,4-*b*]indol-6-one (3a)

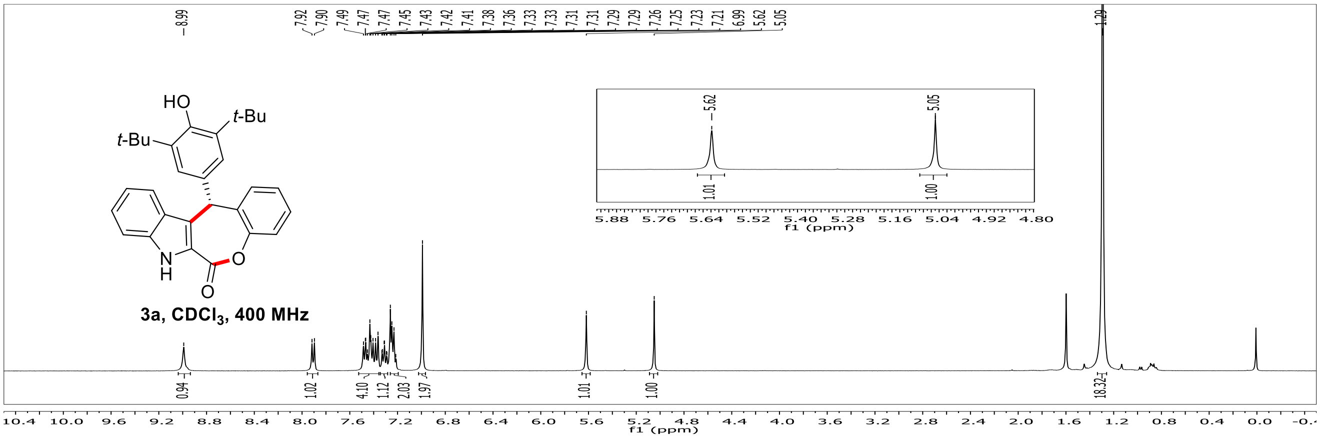

^{1}H-NMR Spectrum of the Reaction Mixture

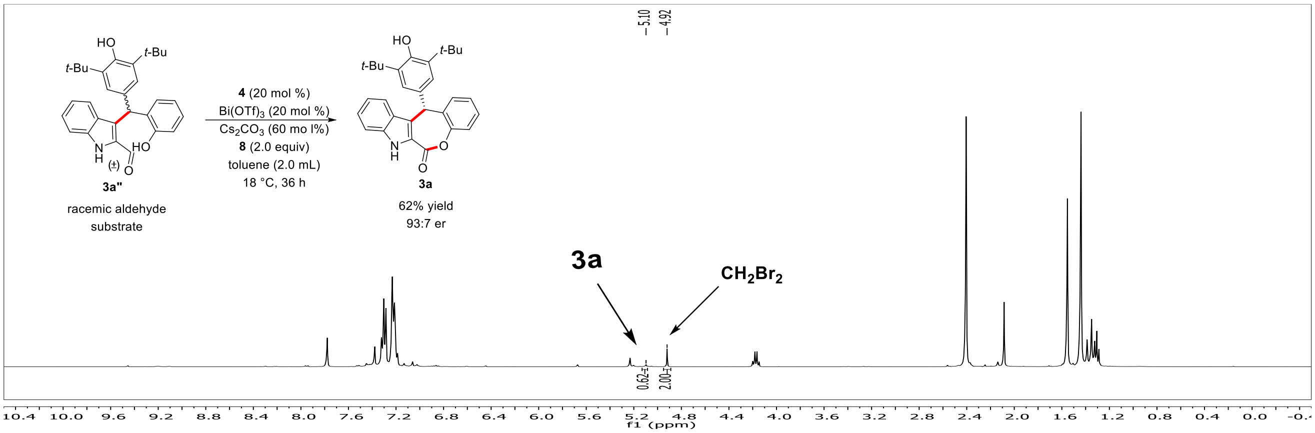

(*S*)-12-(3,5-Di-*tert*-butyl-4-hydroxyphenyl)-7,12-dihydro-6*H*-benzo[6,7]oxepino[3,4-*b*] indol-6-one (3a)

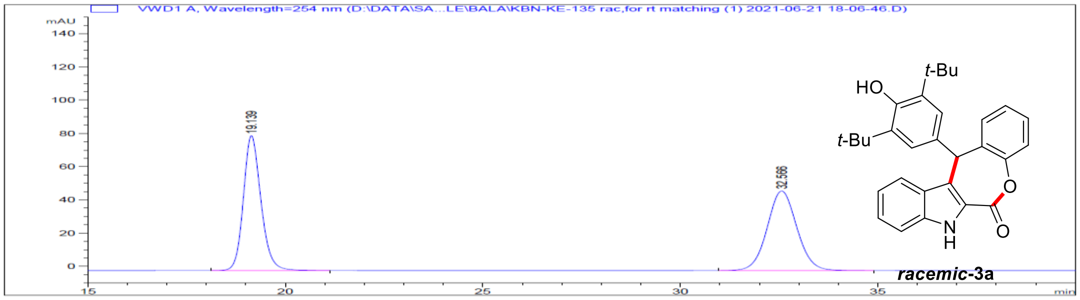

Peak #	RetTime [min]	Type	Width [min]	Area [mAU*s]	Height [mAU]	Area %
1	19.139	BB	0.4718	2500.86304	81.08739	50.0304
2	32.566	BB	0.8066	2497.81885	47.80070	49.9696

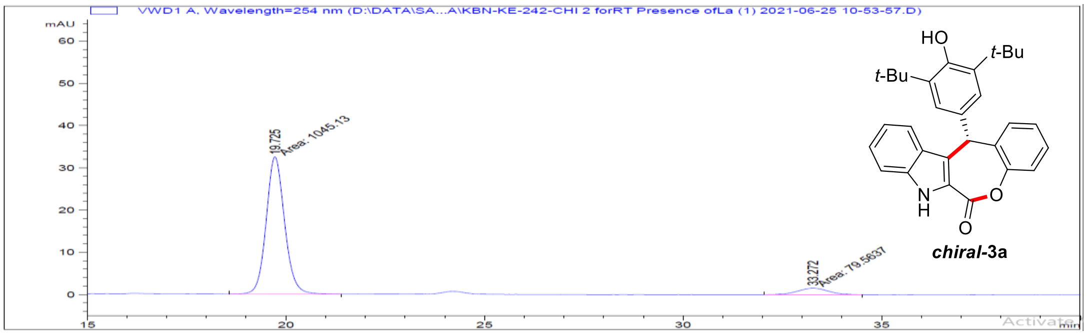

Peak #	RetTime [min]	Type	Width [min]	Area [mAU*s]	Height [mAU]	Area %
1	19.725	MM	0.5369	1045.13416	32.44448	92.9258
2	33.272	MM	0.8763	79.56369	1.51319	7.0742

Sample Info : CHIRALPAK IA, 10 % IPA-HEXANE, 1.0 mL/min, 254 nM.M

The above result indicates that the present reaction is NHC-Lewis acid cooperatively catalyzed dynamic kinetic resolution.

(e) Role of -OH group on PQM (one-pot reaction)

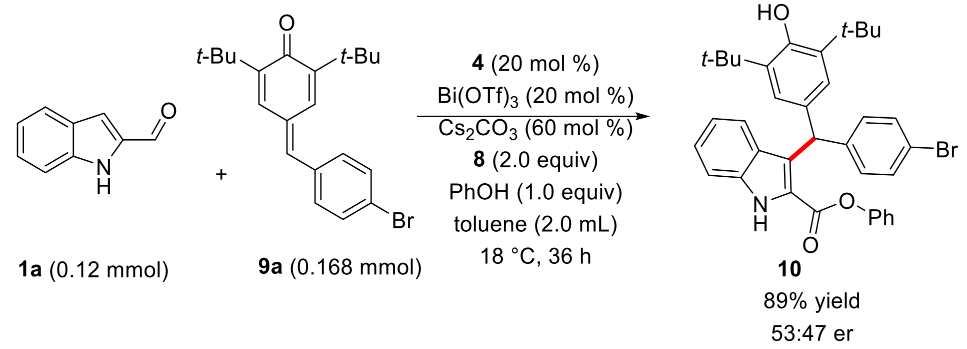

To an oven-dried Schlenk tube with a teflon screw cap was taken the triazolium salt **4** (5.6 mg, 0.012 mmol), Cs_2CO_3 (19.5 mg, 0.06 mmol) and $Bi(OTf)_3$ (15.6 mg, 0.024 mmol) in a glove-box. Then 1*H*-indole-2-carbaldehyde **1a** (17.4 mg, 0.12 mmol), 4-(4-bromobenzylidene)-2,6-di-*tert*-butylcyclohexa-2,5-dien-1-one **9a** (62.5 mg, 0.168 mmol), PhOH (11.3 mg, 0.12 mmol), and oxidant **8** (49.0 mg, 0.12 mmol) were added outside the glove-box under nitrogen atmosphere followed by addition of toluene (1.0 mL). The reaction mixture was stirred at 18 °C for 12 h and then to the stirring solution, Cs_2CO_3 (3.9 mg, 0.012 mmol), and oxidant **8** (49.0 mg, 0.12 mmol) were added again under nitrogen atmosphere followed by addition of toluene (1.0 mL). The reaction mixture was stirred at 18 °C for 24 h under nitrogen atmosphere. After the completion of the reaction, the solvent was evaporated to obtain the crude product, which was analyzed using ^1H NMR using CH_2Br_2 (8.5 µL, 0.12 mmol) as the internal standard. ^1H NMR analysis shows that the crude mixture contains the expected product **10** in 89% yield as well as the crude residue was further purified by flash column chromatography on silica gel to afford the corresponding phenyl 3-((4-bromophenyl)(3,5-di-*tert*-butyl-4-hydroxyphenyl)methyl)-1*H*-indole-2-carboxylate **10** as a white solid (66.0 mg, 89% yield with 53:47 er).

^{1}H-NMR Spectrum of phenyl 3-((4-bromophenyl)(3,5-di-*tert*-butyl-4-hydroxyphenyl)methyl)-1*H*-indole-2-carboxylate (10)

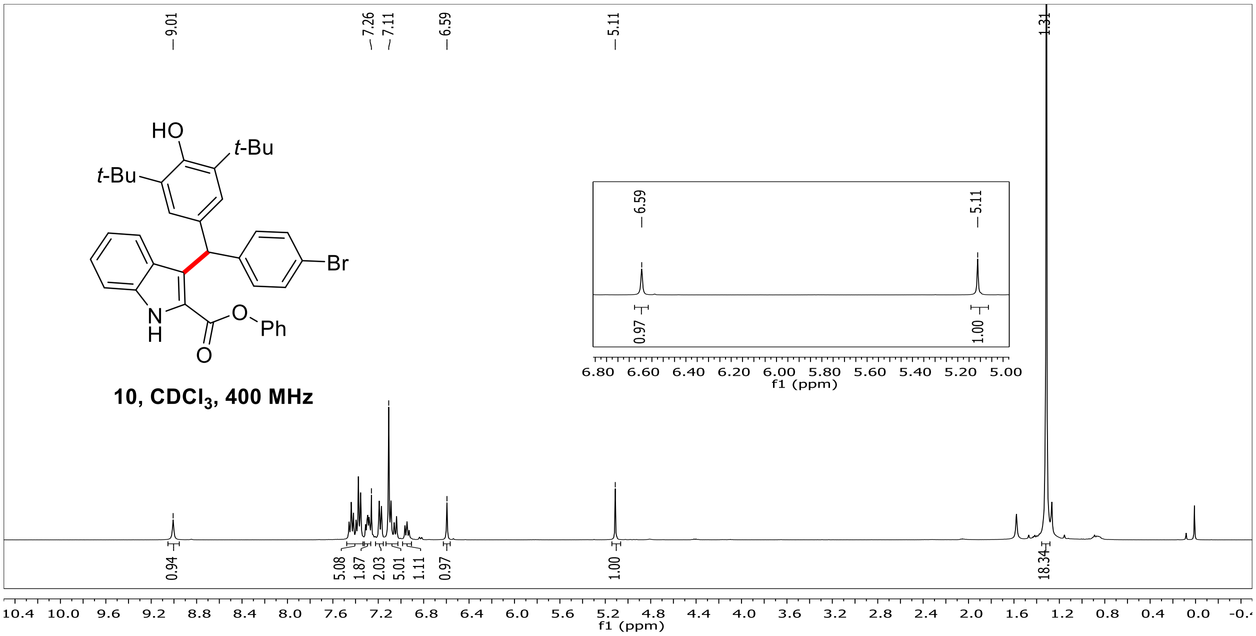

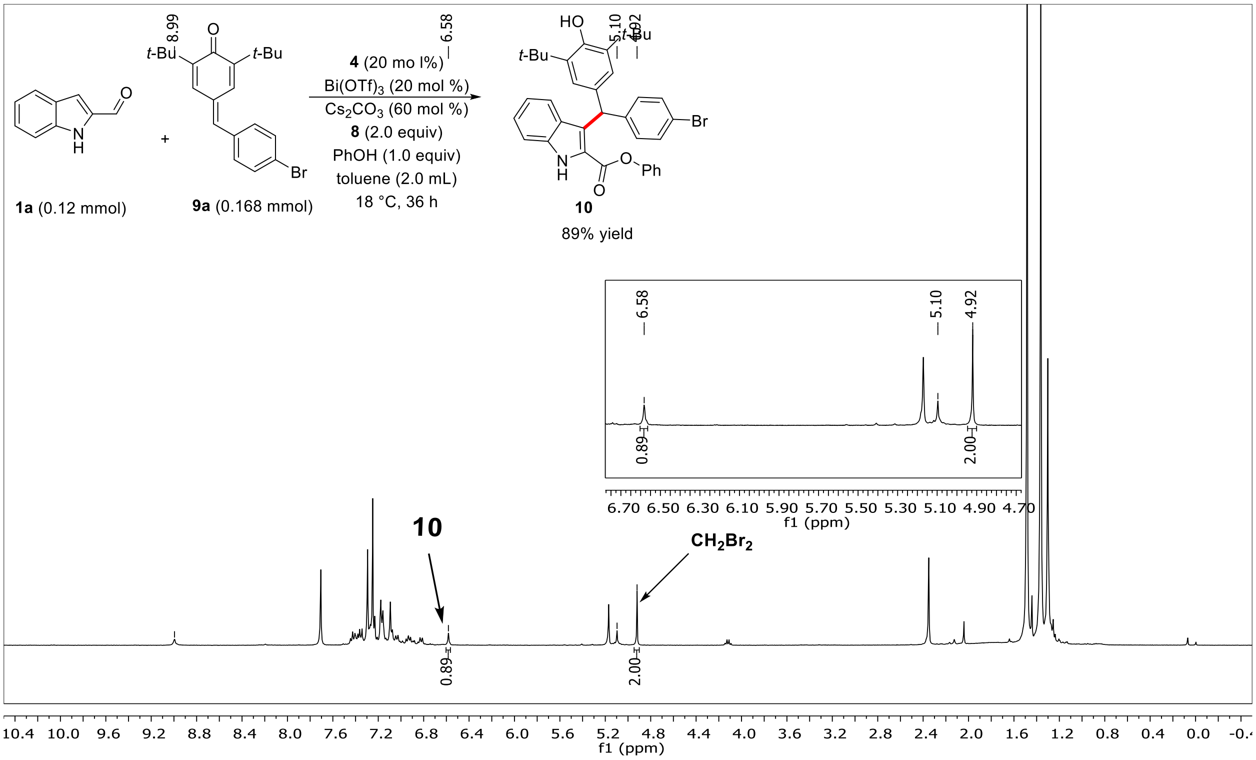

Reaction performed using phenol and paraquinone methide ***9a,*** *which does not contain -OH group afforded the acyclic ester product* ***10*** *in 89% yield with only 6% ee, which indicated that the presence of phenol group on PQM is crucial for getting enantioselectivity.*

(f) To test the feasibility of intramolecular Friedel-Crafts Reaction as the second step

To an oven-dried Schlenk tube with a Teflon screw cap was taken the triazolium salt **4** (11.6 mg, 0.025 mmol), Cs_2CO_3 (41.0 mg, 0.125 mmol) and $Bi(OTf)_3$ (33.0 mg, 0.05 mmol) in a glove-box. Then 2-((3,5-di-*tert*-butyl-4-oxocyclohexa-2,5-dien-1-ylidene)methyl)phenyl 1*H*-indole-2-carboxylate **3a'** (113.4 mg, 0.25 mmol), oxidant **8** (102.0 mg, 0.25 mmol) were added outside the glove-box at 18 °C under nitrogen atmosphere followed by addition of toluene (2.0 mL). The reaction mixture was stirred at 18 °C for 12 h and then to the stirring solution, triazolium salt **4** (11.6 mg, 0.025 mmol), Cs_2CO_3 (8.0 mg, 0.025 mmol), and oxidant **8** (102.0 mg, 0.25 mmol) were added at 18 °C under nitrogen atmosphere followed by addition of toluene (2.0 mL). The reaction mixture was stirred at 18 °C for 24 h under nitrogen atmosphere. The present reaction did not afford the corresponding tetracyclic ε-lactone **3a.**

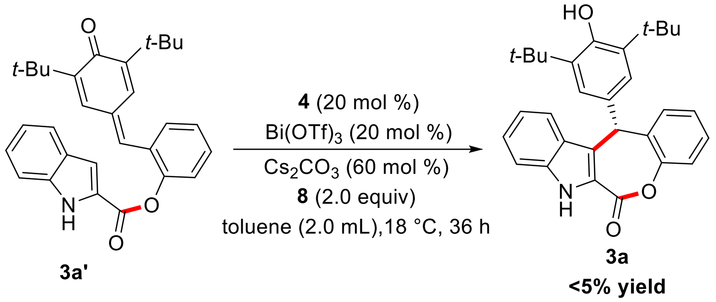

*This reaction did not work and the product **3a** was not formed under these conditions. This indicates that the Friedel-Crafts alkylation proceeds prior to the annulation, which is catalyzed by NHCs.*

(g) Attempted Reaction with other Electron-rich Aldehydes

To an oven-dried Schlenk tube with a teflon screw cap was taken the triazolium salt **4** (11.6 mg, 0.025 mmol), Cs_2CO_3 (41.0 mg, 0.125 mmol) and $Bi(OTf)_3$ (33.0 mg, 0.05 mmol) in a glove-box. Then aromatic/heteroaromatic aldehyde derivatives (0.25 mmol), 2,6-di-*tert*-butyl-4-(2-hydroxybenzylidene)cyclohexa-2,5-dien-1-one **2** (108.6 mg, 0.35 mmol) and oxidant **8** (102.0 mg, 0.25 mmol) were added outside the glove-box at 18 °C under nitrogen atmosphere followed by addition of toluene (2.0 mL). The reaction mixture was stirred at 18 °C for 12 h and then to the stirring solution, triazolium salt **4** (11.6 mg, 0.025 mmol), Cs_2CO_3 (8.0 mg, 0.025 mmol), and oxidant **8** (102.0 mg, 0.25 mmol) were added at 18 °C under nitrogen atmosphere followed by addition of toluene (2.0 mL). The reaction mixture was stirred at 18 °C for 24 h under nitrogen atmosphere.

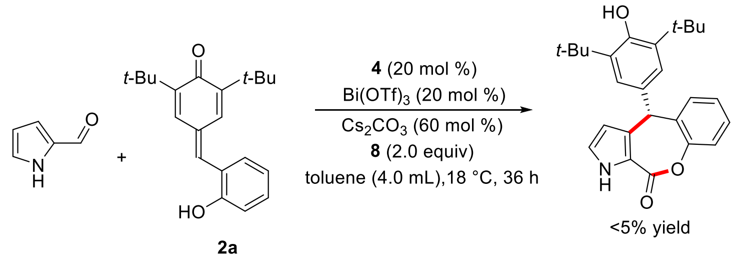

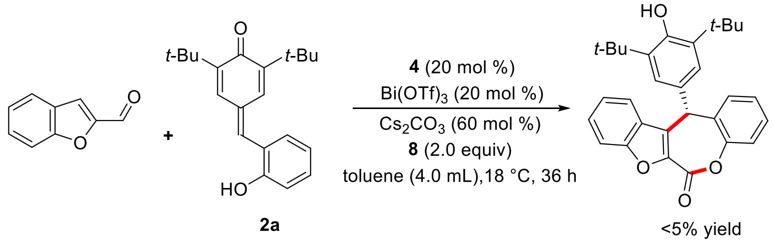

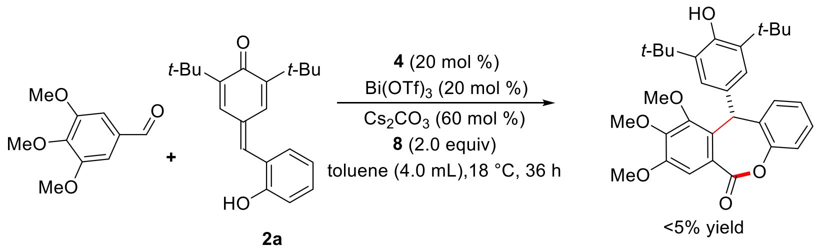

We used the indole 2-carboxaldehydes as nucleophiles because they are sufficiently nucleophilic at the 3-position despite the presence of -CHO moiety at the 2-position. The performed reactions with pyrrole 2-carboxaldehyde, benzofuran 2-carboxaldehyde and 3,4,5-trimethoxy benzaldehyde as nucleophiles did not afford the desired annulated products under the optimized conditions.

(h) Test for the Reversibility of Product Formation

To an oven-dried Schlenk tube with a teflon screw cap was taken the triazolium salt **4** (11.6 mg, 0.025 mmol), Cs_2CO_3 (41.0 mg, 0.125 mmol) and $Bi(OTf)_3$ (33.0 mg, 0.05 mmol) in a glove-box. Then 12-(3,5-di-*tert*-butyl-4-hydroxyphenyl)-7,12-dihydro-6*H*-benzo[6,7]oxepino[3,4-*b*]indol-6-one (+/-)**3a** (113.4 mg, 0.25 mmol), oxidant **8** (102.0 mg, 0.25 mmol) were added outside the glove-box at 18 °C under nitrogen atmosphere followed by addition of toluene (2.0 mL). The reaction mixture was stirred at 18 °C for 12 h and then to the stirring solution, triazolium salt **4** (11.6 mg, 0.025 mmol), Cs_2CO_3 (8.0 mg, 0.025 mmol), and oxidant **8** (102.0 mg, 0.25 mmol) were added at 18 °C under nitrogen atmosphere followed by addition of toluene (2.0 mL). The reaction mixture was stirred at 18 °C for 24 h under nitrogen atmosphere, after the completion of the reaction solvent was evaporated, and the crude residue was purified by flash column chromatography on silica gel to afford the corresponding tetracyclic ε-lactones (+/-)**3a** as a white

solid (112.0 mg, 99% yield with 50:50 er). The enantiomeric ratio was determined by HPLC analysis on a chiral column.

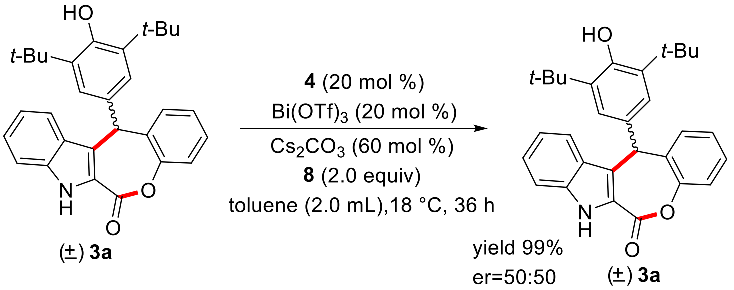

*The performed a reaction of racemic (+/-)**3a** under the optimized reaction conditions did not afford the enantioenriched lactone **3a**. This indicates the irreversible nature of the present annulation.*

(i) Test the possibility of Indole N-H Functionalization

To an oven-dried Schlenk tube with a Teflon screw cap was taken the triazolium salt **4** (11.6 mg, 0.025 mmol), Cs_2CO_3 (41.0 mg, 0.125 mmol) and $Bi(OTf)_3$ (33.0 mg, 0.05 mmol) in a glove-box. Then 1*H*-indole-2-carbaldehyde **1a** (36.3 mg, 0.25 mmol), 2,6-di-*tert*-butyl-4-(2-hydroxybenzylidene)cyclohexa-2,5-dien-1-one **2a** (108.6 mg, 0.35 mmol), oxidant **8** (102.0 mg, 0.25 mmol) were added outside the glove-box at 18 °C under nitrogen atmosphere followed by addition of toluene (2.0 mL) and 2,2,2-trifluoro-1-phenylethan-1-one (70.0 µL, 0.5 mmol). The reaction mixture was stirred at 18 °C for 12 h and then to the stirring solution, triazolium salt **4** (11.6 mg, 0.025 mmol), Cs_2CO_3 (8.0 mg, 0.025 mmol), and oxidant **8** (102.0 mg, 0.25 mmol) were added at 18 °C under nitrogen atmosphere followed by addition of toluene (2.0 mL). The reaction mixture was stirred at 18 °C for 24 h under nitrogen atmosphere, after the completion of the reaction solvent was evaporated, and the crude mixture was passed through a pad of silica gel and eluted with EtOAc (3x10 mL). The reaction mixture was concentrated under reduced pressure and then the yield of **3a** was determined by the ^{1}H NMR analysis of the crude reaction products using CH_2Br_2 as the internal standard.

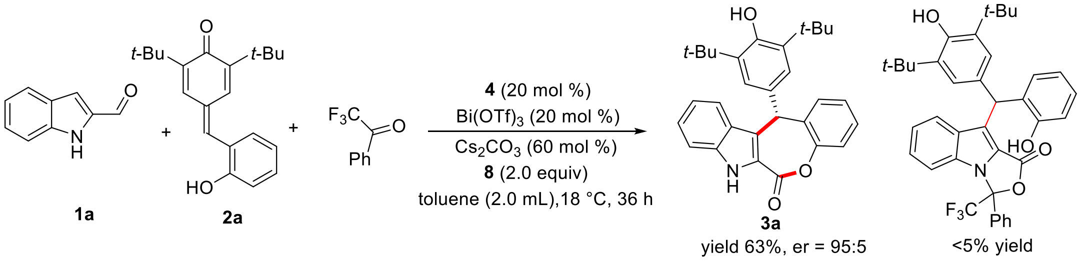

*The desired ε-lactone **3a** was formed in 63% yield and the product derived from NHC-aza-fulvene intermediate was not observed in this case. These results likely indicate that the initial Friedel-Crafts alkylation is faster than the formation of acylazolium intermediate from indole2-aldehyde.*

(j) Reaction Using N-Methyl Indole 2-Carboxaldehyde

Following the general procedure, treatment of 1-methyl-1*H*-indole-2-carbaldehyde (39.8 mg, 0.25 mmol) and 2,6-di-*tert*-butyl-4-(2-hydroxybenzylidene)cyclohexa-2,5-dien-1-one **2** (108.6 mg, 0.35 mmol) with triazolium salt **4** (23.2 mg, 0.05 mmol), Cs_2CO_3 (49.0 mg, 0.15 mmol), oxidant **5** (204.0 mg, 0.5 mmol), $Bi(OTf)_3$ (33.0 mg, 0.05mmol) in toluene (4.0 mL) and stirring the reaction mixture at 18 °C for 36 h followed by flash column chromatography (Pet. ether - EtOAc: 98:2 to 90:10) to afford 12-(3,5-Di-*tert*-butyl-4-hydroxyphenyl)-7-methyl-7,12-dihydro-6*H*-benzo[6,7]oxepino[3,4-*b*] indol-6-one as a white solid (117.0 mg, 93 % yield with 50:50 er). ***R_f*** (Pet. ether /EtOAc = 90/10): 0.42; er = 50:50, **HPLC** (Chiralpak IA, *n*-hexane/IPA = 98:2, flow rate = 1.0 mL/min, λ = 254 nm) t_R = 5.1 min, 10.4 min.

^{1}H NMR (400 MHz, $CDCl_3$) δ 7.89 (d, *J* = 8.1 Hz, 1H), 7.46-7.39 (m, 3H), 7.34-7.29 (m, 2H), 7.24-7.19 (m, 2H), 6.93 (s, 2H), 5.61 (s, 1H), 5.04 (s, 1H), 4.03 (s, 3H), 1.28 (s, 18H). **^{13}C NMR (100 MHz, $CDCl_3$)** δ 159.7, 152.6, 151.6, 139.6, 136.1, 135.8, 131.6, 130.3, 129.6, 128.3, 126.7, 126.0, 125.0, 123.8, 122.6, 122.6, 122.0, 121.0, 120.5, 110.6, 45.3, 34.4, 31.8, 30.2. **HRMS (ESI) m/z:** $[M+H]^+$ calcd for $C_{31}H_{34}NO_3$ 468.2533; found 468.2538. **FTIR (cm^{-1})** 3635, 2960, 2870,1713, 1488, 1434, 1236, 1177, 1070, 882.

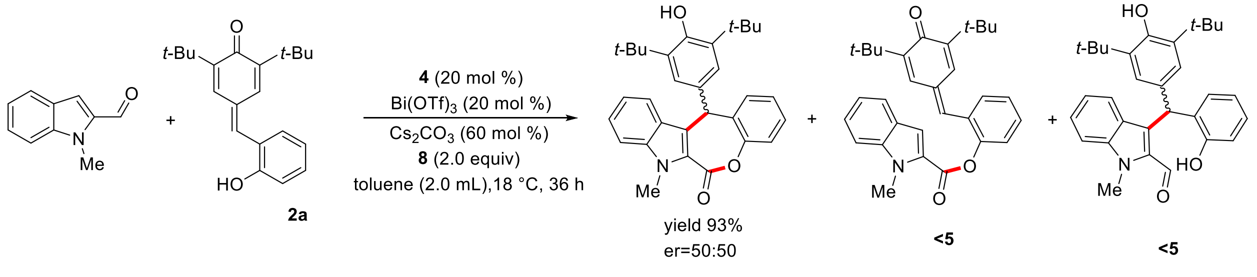

*The reaction afforded the N-methyl ε-lactones in 93% yield and er 50:50; This result indicates that the free N-H is required for getting selectivity. It could be acylazolium **III** stabilized by intramolecular hydrogen bonding with the N-H moiety.*

12-(3,5-di-*tert*-butyl-4-hydroxyphenyl)-7-methyl-7,12-dihydro-6*H*-benzo[6,7]oxepino[3,4-*b*]indol-6-one

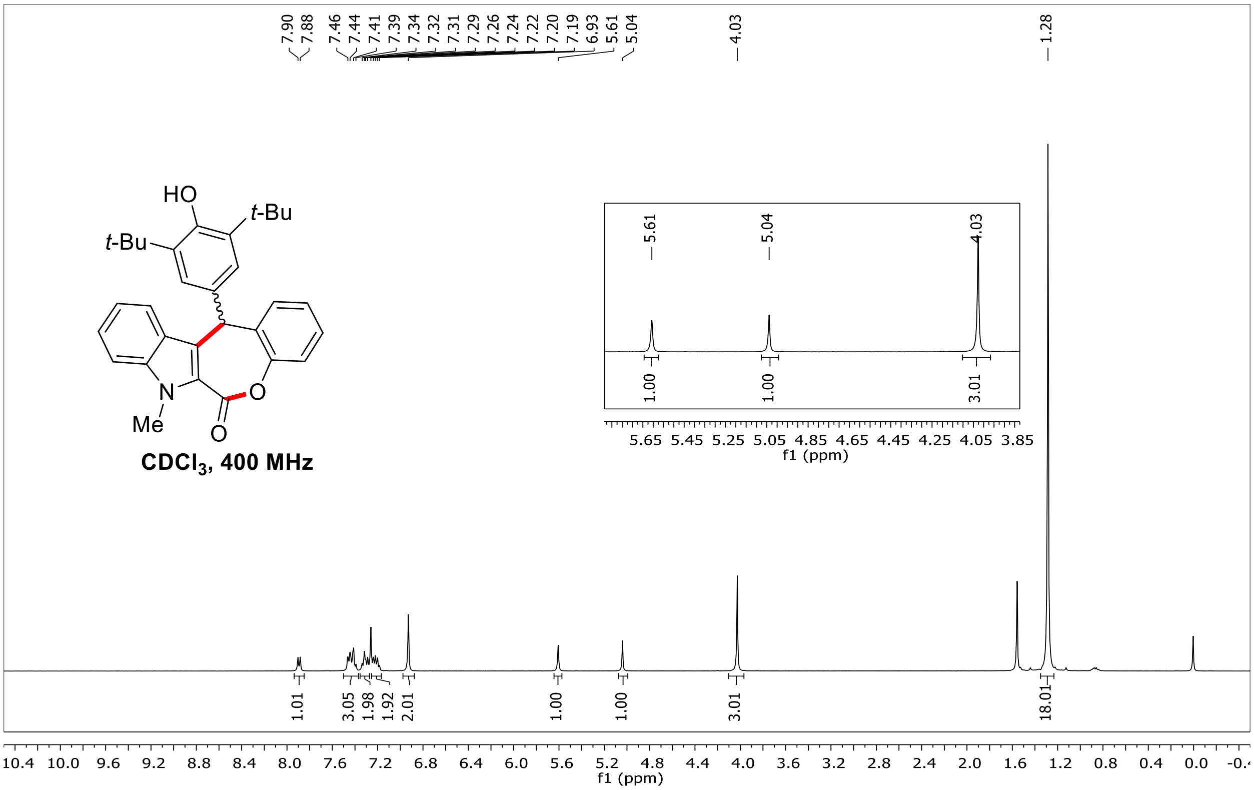

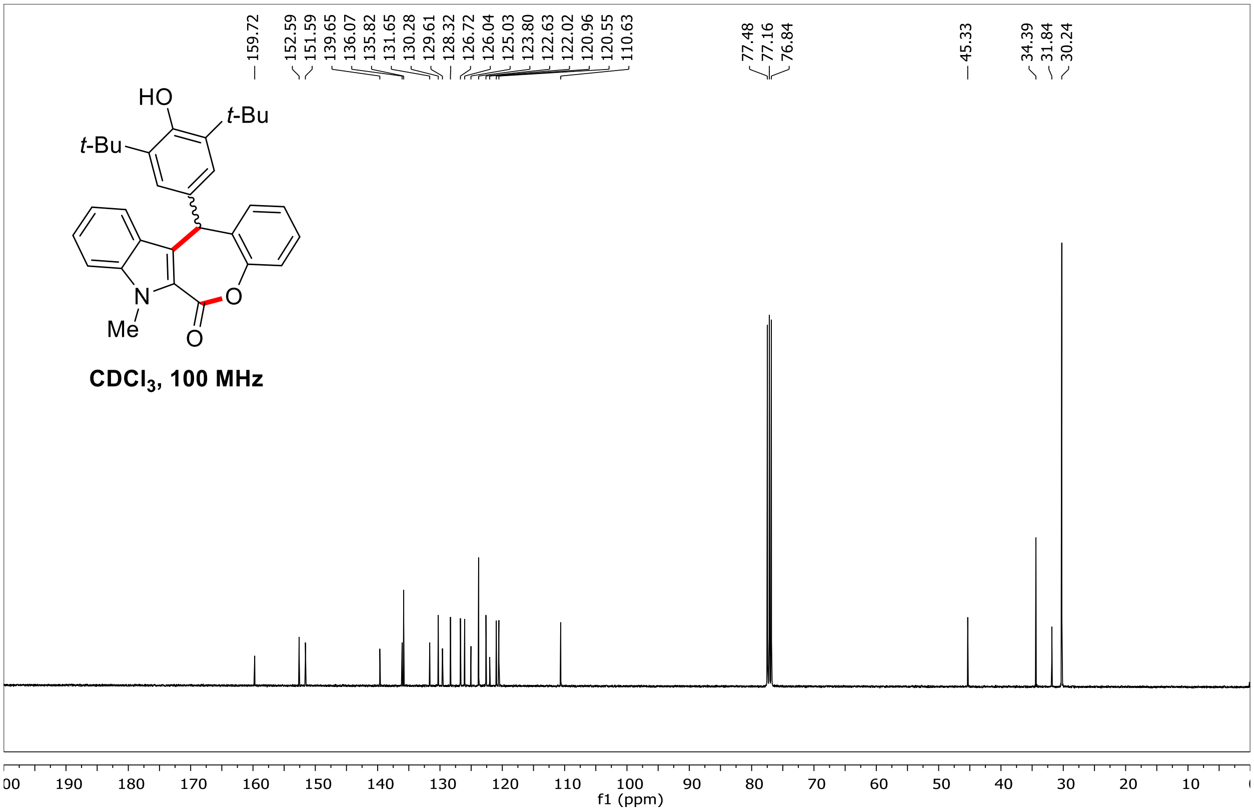

(k) Tolerance of the Reaction with Common Functional Groups

To an oven-dried Schlenk tube with a teflon screw cap was taken the triazolium salt **4** (11.6 mg, 0.025 mmol), Cs_2CO_3 (41.0 mg, 0.125 mmol) and $Bi(OTf)_3$ (33.0 mg, 0.05 mmol) in a glove-box. Then 1*H*-indole-2-carbaldehyde derivative **1** (36.3 mg, 0.25 mmol), 2,6-di-*tert*-butyl-4-(2-hydroxybenzylidene)cyclohexa-2,5-dien-1-one derivative **2** (108.6 mg, 0.35 mmol), oxidant **8** (102.0 mg, 0.25 mmol) and **electrophile** (0.25 mmol) were added outside the glove-box at 18 °C under nitrogen atmosphere followed by addition of toluene (2.0 mL). The reaction mixture was stirred at 18 °C for 12 h and then to the stirring solution, triazolium salt **4** (11.6 mg, 0.025 mmol), Cs_2CO_3 (8.0 mg, 0.025 mmol), and oxidant **8** (102.0 mg, 0.25 mmol) were added at 18 °C under nitrogen atmosphere followed by addition of toluene (2.0 mL). The reaction mixture was stirred at 18 °C for 24 h under nitrogen atmosphere, after the completion of the reaction solvent was evaporated, and the crude mixture was passed through a pad of silica gel and eluted with EtOAc (3x10 mL). The reaction mixture was concentrated under reduced pressure and then the yield of recovered electrophile and **3a** was determined by the ^{1}H NMR analysis of the crude reaction products using CH_2Br_2 as the internal standard.

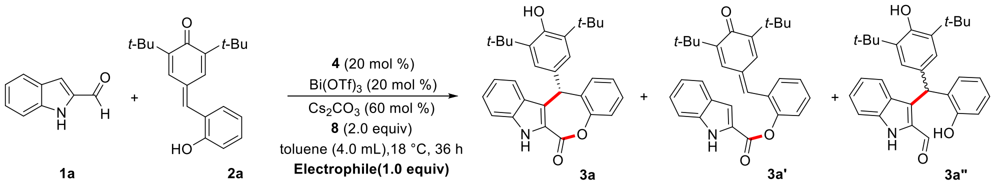

Entry	Electrophile	Electrophile remaining (%)[b]	Yield of **3a** (%)[b]	er of **3a** (%)[c]	Yield of **3a'**(%)[b]	Yield of **3a''**(%)[b]
1	none	–	68	95:5	<5	<5
2	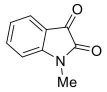	100	40	95:5	<5	49
3	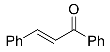	100	68	95:5	<5	<5
4	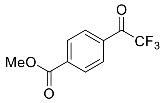	92	67	95:5	<5	18
5	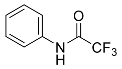	92	60	95:5	<5	<5
6	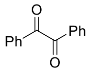	93	33	95:5	<5	55
7	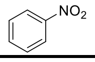	94	66	95:5	<5	<5

[a] Reaction conditions: **1** (0.25 mmol), **2** (1.4 equiv), Electrophile (0.25 mmol), **4** (20 mol %), $Bi(OTf)_3$ (20 mol %), Cs_2CO_3 (60 mol %), **8** (2.0 equiv), toluene (4.0 mL), 18 °C and 36 h. [b] Yield was determined on the basis of the ^{1}H NMR of the crude products using CH_2Br_2 as the internal standard. [c] The er was established by HPLC analysis on a chiral stationary phase.

7. Synthesis and Characterization of Tetracyclic ε-Lactones

(*S*)-12-(3,5-Di-*tert*-butyl-4-hydroxyphenyl)-7,12-dihydro-6*H*-benzo[6,7]oxepino[3,4-*b*]indol-6-one (3a)

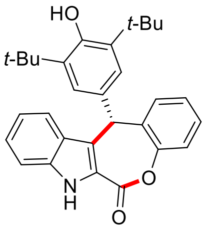

3a

Following the general procedure, treatment of 1*H*-indole-2-carbaldehyde **1a** (36.3 mg, 0.25 mmol) and 2,6-di-*tert*-butyl-4-(2-hydroxybenzylidene) cyclohexa-2,5-dien-1-one **2a** (108.6 mg, 0.35 mmol) with triazolium salt **4** (23.2 mg, 0.05 mmol), Cs_2CO_3 (49.0 mg, 0.15 mmol), oxidant **8** (204.0 mg, 0.5 mmol), $Bi(OTf)_3$ (33.0 mg, 0.05mmol) in toluene (4.0 mL) and stirring the reaction mixture at 18 °C for 36 h followed by flash column chromatography (Pet. ether -EtOAc: 98:2 to 90:10) to afford (*S*)-12-(3,5-di-*tert*-butyl-4-hydroxyphenyl)-7,12-dihydro-6*H*-benzo[6,7]oxepino [3,4-*b*]indol-6-one (**3a**) as a white solid (78.0 mg, 69 % yield with 95:5 er).

R_f (Pet. ether /EtOAc = 90/10): 0.40; er = 95:5, $[\alpha]_D^{25}$ = +83.98 (c 1.0, $CHCl_3$). **HPLC** (Chiralpak IA, *n*-hexane/IPA = 90:10, flow rate = 1.0 mL/min, λ = 254 nm) t_R = 19.0 min (major), 32.5 min (minor).

^{1}H NMR (400 MHz, $CDCl_3$) δ 8.99 (s, 1H), 7.91 (d, *J* = 8.15 Hz, 1H), 7.49-7.36 (m, 4H), 7.33-7.29 (m, 1H), 7.25-7.21 (m, 2H), 6.99 (s, 2H), 5.62 (s, 1H), 5.05 (s, 1H), 1.29 (s, 18H). **^{13}C NMR (100 MHz, $CDCl_3$)** δ 160.7, 152.6, 151.4, 137.3, 135.9, 134.8, 132.0, 130.9, 129.0, 128.5, 127.2, 126.3, 126.2, 123.8, 123.0, 121.5, 121.2, 120.8, 112.4, 46.0, 34.4, 30.2. **HRMS (ESI) m/z:** $[M+Na]^+$ calcd for $C_{30}H_{31}NO_3Na$ 476.2196; found 476.2192. **FTIR (cm^{-1})** 3630, 3334, 2960, 2869, 2335, 1690, 1433, 1233, 1094, 895.

(*S*)-12-(3,5-Di-*tert*-butyl-4-hydroxyphenyl)-11-fluoro-7,12-dihydro-6*H*-benzo[6,7]oxepino[3,4-*b*]indol-6-one (3b)

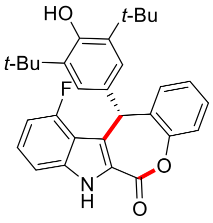

3b

Following the general procedure, treatment of 4-fluoro-1*H*-indole-2-carbaldehyde **1b** (41.0 mg, 0.25 mmol) and 2,6-di-*tert*-butyl-4-(2-hydroxybenzylidene)cyclohexa-2,5-dien-1-one **2a** (108.6 mg, 0.35 mmol) with triazolium salt **4** (23.2 mg, 0.05 mmol), Cs_2CO_3 (49.0 mg, 0.15 mmol), oxidant **8** (204.0 mg, 0.5 mmol), $Bi(OTf)_3$ (33.0 mg, 0.05mmol) in toluene (4.0 mL) and stirring the reaction mixture at 18 °C for 36 h followed by flash column chromatography (Pet. ether -EtOAc: 98:2 to 90:10) to afford (*S*)-12-(3,5-di-*tert*-butyl-4-hydroxyphenyl)-11-fluoro-7,12-dihydro-6*H*-benzo[6,7]oxepino[3,4-*b*]indol-6-one (**3b**) as a white solid (111.0 mg, 93 % yield with

96:4 er). ***Rf*** (Pet. ether /EtOAc = 90/10): 0.49; er = 96:4, $[\alpha]_D^{25}$ = +58.91 (c 1.0, $CHCl_3$). **HPLC** (Chiralpak IA, *n*-hexane/IPA = 90:10, flow rate = 1.0 mL/min, λ = 254 nm) t_R = 26.6 min (major), 33.2 min (minor).

^{1}H NMR (400 MHz, $CDCl_3$) δ 9.09 (s, 1H), 7.84 (s, 1H), 7.47-7.46 (m, 1H), 7.36-7.30 (m, 2H), 7.24-7.24 (m, 1H), 7.13-7.11 (m, 1H), 7.04-6.96 (m, 3H), 5.56 (s, 1H), 5.07 (s, 1H), 1.29 (s, 18H). **^{13}C NMR (100 MHz, $CDCl_3$)** δ 162.9 (d, *J* = 244.9 Hz), 160.5, 152.7, 151.4, 137.5 (d, *J* = 12.9 Hz), 136.0, 134.6, 131.7, 130.9, 129.3, 128.7, 126.4, 123.8, 123.0, 122.2 (d, *J* = 10.7 Hz), 122.1 (d, *J* = 3.5 Hz), 111.08 (d, *J* = 24.9 Hz), 98.4 (d, *J* = 26.3 Hz), 46.1, 34.4, 30.2. **^{19}F-NMR (376 MHz, $CDCl_3$)** δ = -113.1. **HRMS (ESI) m/z:** $[M+H]^+$ calcd for $C_{30}H_{31}FNO_3$: 472.2282, found: 472.2288. **FTIR (cm^{-1})** 3614, 3322, 2959, 2333, 1691, 1628, 1433, 1317, 1235, 1082, 1024, 840.

(*S*)-12-(3,5-Di-*tert*-butyl-4-hydroxyphenyl)-10-methoxy-7,12-dihydro-6*H*-benzo[6,7] oxepino[3, 4-b]indol-6-one (3c)

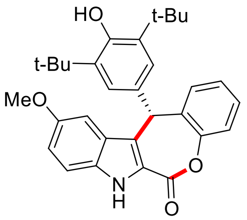

3c

Following the general procedure, treatment of 5-methoxy-1*H*-indole-2-carbaldehyde **1c** (43.8 mg, 0.25mmol) and 2,6-di-*tert*-butyl-4-(2-hydroxybenzylidene)cyclohexa-2,5-dien-1-one **2a** (108.6 mg, 0.35 mmol) with triazolium salt **4** (23.2 mg, 0.05 mmol), Cs_2CO_3 (49 mg, 0.15 mmol), oxidant **8** (204 mg, 0.5 mmol), $Bi(OTf)_3$ (33.0 mg, 0.05mmol) in toluene (4.0 mL) and stirring the reaction mixture at 18 °C for 36 h followed by flash column chromatography (Pet. ether -EtOAc: 98:2 to 90:10) to afford (*S*)-12-(3,5-di-*tert* butyl-4-hydroxyphenyl)-10-methoxy-7, 12-dihydro-6*H*-benzo[6,7]oxepino[3, 4-b]indol-6-one **(3c)** as a white solid (87 mg, 72 % yield with 94:6 er). ***Rf*** (Pet. ether /EtOAc = 85/15): 0.41; er =94:6, $[\alpha]_D^{25}$ = +21.38 (c 1.0, $CHCl_3$). **HPLC** (Chiralpak IA, *n*-hexane/IPA = 90:10, flow rate = 1.0 mL/min, λ = 254 nm) t_R = 12.3 min (major), 24.1 min (minor).

^{1}H NMR (400 MHz, $CDCl_3$) δ 9.04 (s, 1H), 7.49 (d, *J* = 6.41 Hz, 1H), 7.38-7.29 (m, 3H), 7.24-7.22 (m, 2H), 7.10-7.07 (m, 1H), 7.00 (s, 2H), 5.56 (s, 1H), 5.06 (s, 1H), 3.91 (s, 3H), 1.29 (s, 18H). **^{13}C NMR (100 MHz, $CDCl_3$)** δ 160.7, 155.1, 152.5, 151.5, 135.9, 134.9, 132.8, 132.1, 130.9, 128.5, 128.3, 126.5, 126.2, 123.8, 123.0, 121.9, 119.2, 113.5, 100.3, 55.9, 46.0, 34.4, 30.2. **HRMS (ESI) m/z:** $[M+H]^+$ calcd for $C_{31}H_{34}NO_4$ 484.2482; found 484.2485. **FTIR (cm^{-1})** 3104, 3064, 2924, 2854, 2377, 2305, 1694, 1629, 1586, 1433, 1117, 1021, 900, 851.

(*S*)-12-(3,5-Di-*tert*-butyl-4-hydroxyphenyl)-10-methyl-7,12-dihydro-6*H*benzo [6,7]oxepino[3,4-b]indol-6-one (3d)

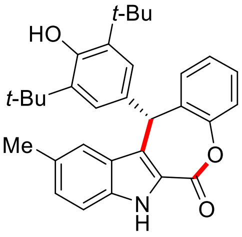

3d

Following the general procedure, treatment of 5-methyl-1*H*-indole-2-carbaldehyde **1d** (39.8 mg, 0.25 mmol) and 2,6-di-*tert*-butyl-4-(2-hydroxybenzylidene)cyclohexa-2,5-dien-1-one **2a** (108.6 mg, 0.35 mmol) with triazolium salt **4** (23.2 mg, 0.05 mmol), Cs_2CO_3 (49.0 mg, 0.15 mmol), oxidant **8** (204.0 mg, 0.5 mmol), $Bi(OTf)_3$ (33.0 mg, 0.05mmol) in toluene (4.0 mL) and stirring the reaction mixture at 18 °C for 36 h followed by flash column chromatography (Pet. ether -EtOAc: 98:2 to 90:10) to afford (*S*)-12-(3,5-di-*tert*-butyl-4-hydroxyphenyl)-10-methyl-7,12-dihydro-6*H* benzo [6,7]oxepino[3,4-b]indol-6-one (**3d**) as a yellow solid (84.1 mg, 72 % yield with >99:1 er).

$\boldsymbol{R_f}$ (Pet. ether /EtOAc = 90/10): 0.24; er = >99:1, $[\alpha]_D^{25}$ = +32.65 (c 1.0, $CHCl_3$). **HPLC** (Chiralpak IA, *n*-hexane/IPA = 90:10, flow rate = 1.0 mL/min, λ = 254 nm) t_R = 11.2 min (major).

^{1}H NMR (400 MHz, $CDCl_3$) δ 8.97 (s, 1H), 7.65 (s, 1H), 7.46 (d, *J* = 7.4 Hz, 1H), 7.36-7.19 (m, 5H), 6.99 (s, 2H), 5.58 (s, 1H), 5.04 (s, 1H), 2.48 (s, 3H), 1.29 (s, 18H). **^{13}C NMR (100 MHz, $CDCl_3$)** δ 160.8, 152.6, 151.4, 135.8, 135.0, 132.0, 130.8, 130.6, 129.2, 128.4, 128.3, 126.4, 126.2, 123.8, 123.0, 121.6, 119.9, 112.1, 46.0, 34.4, 30.2, 21.7. **HRMS (ESI)** calculated $[M+Na]^+$ for $C_{31}H_{33}NNaO_3$: 490.2353, found: 490.2355. **FTIR (cm^{-1})** 3635, 3325, 2959, 2867, 1690, 1433, 1232, 1076, 863.

(*S*)-12-(3,5-di-*Tert*-butyl-4-hydroxyphenyl)-10-ethyl-7,12-dihydro-6*H*-benzo[6,7]oxepino[3,4-*b*]indol-6-one (3e)

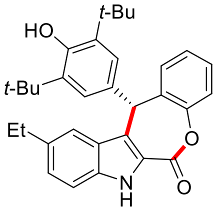

3e

Following the general procedure, treatment of 5-ethyl-1*H*-indole-2-carbaldehyde **1e** (43.3 mg, 0.25 mmol) and 2,6-di-*tert*-butyl-4-(2-hydroxybenzylidene)cyclohexa-2,5-dien-1-one **2a** (108.6 mg, 0.35 mmol) with triazolium salt **4** (23.2 mg, 0.05 mmol), Cs_2CO_3 (49.0 mg, 0.15 mmol), oxidant **8** (204.0 mg, 0.5 mmol), $Bi(OTf)_3$ (33.0 mg, 0.05mmol) in toluene (4.0 mL) and stirring the reaction mixture at 18 °C for 36 h followed by flash column chromatography (Pet. ether -EtOAc: 98:2 to 90:10) to afford (*S*)-12-(3,5-di-*Tert*-butyl-4-hydroxyphenyl)-10-ethyl-7,12-dihydro-6*H*-benzo[6,7]oxepino[3,4-*b*]indol-6-one (3e) as a white solid (89.7 mg, 75 % yield with 98:2 er).

R_f (Pet. ether /EtOAc = 90/10): 0.29; er = 98:2, $[\alpha]_D^{25}$ = +28.4 (c 0.1, $CHCl_3$). **HPLC** (Chiralpak IA, *n*-hexane/IPA = 90:10, flow rate = 1.0 mL/min, λ = 254 nm) t_R = 11.4 min (major), 27.3 min (minor).

^{1}H NMR (400 MHz, $CDCl_3$) δ 8.99-8.98 (m, 1H), 7.70 (s, 1H), 7.50-7.48 (m, 1H), 7.38-7.35 (m, 2H), 7.33-7.26 (m, 2H), 7.25-7.21 (m, 1H), 7.02 (s, 2H), 5.62 (s, 1H), 5.06 (s, 1H), 2.80 (q, *J* = 7.4 Hz, 2H), 1.34-1.30 (m, 21H). **^{13}C NMR (100 MHz, $CDCl_3$)** δ 160.8, 152.6, 151.6, 137.3, 136.0, 135.9, 134.9, 132.2, 130.9, 128.7, 128.5, 128.3, 126.5, 126.3, 123.9, 123.0, 121.6, 118.7, 112.2, 46.0, 34.4, 30.3, 29.2, 16.3. **HRMS (ESI)** calculated $[M+H]^+$ for $C_{32}H_{36}NO_3$: 482.2690, found: 482.2692. **FTIR (cm^{-1})** 3451, 3417, 2918, 2867, 1691, 1629, 1433, 1335, 1174.

(*S*)-10-(*Tert*-butyl)-12-(3,5-di-*tert*-butyl-4-hydroxyphenyl)-7,12-dihydro-6*H* benzo[6,7]oxepino[3,4-b]indol-6-one (3f)

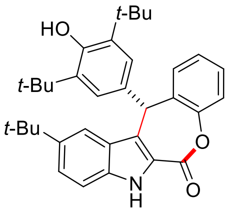

3f

Following the general procedure, treatment of 5-(*tert*-butyl)-1*H*-indole-2-carbaldehyde **1f** (52.6 mg, 0.25 mmol) and 2,6-di-*tert*-butyl-4-(2 hydroxybenzylidene)cyclohexa-2,5-dien-1-one **2a** (108.6 mg, 0.35 mmol) with triazolium salt **4** (23.2 mg, 0.05 mmol), Cs_2CO_3 (49.0 mg, 0.15 mmol), oxidant **8** (204.0 mg, 0.5 mmol), $Bi(OTf)_3$ (33.0 mg, 0.05mmol) in toluene (4.0 mL) and stirring the reaction mixture at 18 °C for 36 h followed by flash column chromatography (Pet. ether -EtOAc: 98:2 to 90:10) to afford (*S*)-10-(*tert*-butyl)-12-(3,5-di-*tert*-butyl-4-hydroxyphenyl)-7,12-dihydro-6*H* benzo[6,7]oxepino[3,4-b]indol-6-one (**3f**) as a white solid (81.4 mg, 64 % yield with 95:5 er).

R_f (Pet. ether /EtOAc = 90/10): 0.28; er = 95:5, $[\alpha]_D^{25}$ = +19.5 (c 1.0, $CHCl_3$). **HPLC** (Chiralpak IA, *n*-hexane/IPA = 98:2, flow rate = 1.0 mL/min, λ = 254 nm) t_R = 9.6 min (minor), 11.6 min (major).

^{1}H NMR (400 MHz, $CDCl_3$) δ 8.82 (s, 1H), 7.86 (s, 1H), 7.52-7.48 (m, 2H), 7.40-7.35 (m, 2H), 7.34-7.29 (m, 1H), 7.25-7.21 (m, 1H), 7.03 (s, 2H), 5.63 (s, 1H), 5.06 (s, 1H), 1.42 (s, 9H),1.29 (s, 18H). **^{13}C NMR (100 MHz, $CDCl_3$)** δ 160.8, 152.6, 151.6, 144.2, 135.9, 135.6, 134.6, 132.3, 131.1, 129.3, 128.5, 126.3, 126.1, 126.0, 123.9, 123.0, 121.3, 115.9, 112.0, 45.9, 35.0, 34.4, 31.8. **HRMS (ESI)** calculated $[M+H]^+$ for $C_{34}H_{40}NO_3$: 510.3003, found: 510.3008. **FTIR (cm^{-1})** 3189, 3069, 2960, 2868, 2362, 1690, 1433, 1232, 1067, 868.

(*S*)-12-(3,5-Di-*tert*-butyl-4-hydroxyphenyl)-10-(trifluoromethoxy)-7,12-dihydro-6*H*-benzo[6,7]oxepino[3,4-b]indol-6-one (3g)

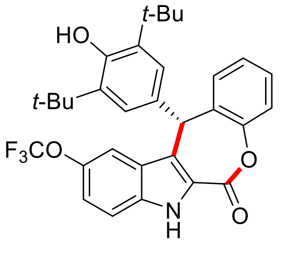

3g

Following the general procedure, treatment of 5-(trifluoromethoxy)-1*H*-indole-2-carbaldehyde **1g** (57.2 mg, 0.25 mmol) and 2,6-di-*tert*-butyl-4-(2-hydroxybenzylidene)cyclohexa-2,5-dien-1-one **2a** (108.6 mg, 0.35 mmol) with triazolium salt **4** (23.2 mg, 0.05 mmol), Cs_2CO_3 (49.0 mg, 0.15 mmol), oxidant **8** (204.0 mg, 0.5 mmol), $Bi(OTf)_3$ (33.0 mg, 0.05mmol) in toluene (4.0 mL) and stirring the reaction mixture at 18 °C for 36 h followed by flash column chromatography (Pet. ether -EtOAc: 98:2 to 90:10) to afford (*S*)-12-(3,5-Di-*tert*-butyl-4-hydroxyphenyl)-10-(trifluoromethoxy)-7,12-dihydro-6*H*-benzo[6,7]oxepino[3,4-*b*]indol-6-one (**3g**) as a white solid (78.0 mg, 72 % yield with 91:9 er).

R_f (Pet. ether /EtOAc = 90/10): 0.25; er = 91:9, $[\alpha]_D^{25}$ = +4.30 (c 1.0, $CHCl_3$). **HPLC** (Chiralpak IA, *n*-hexane/IPA = 90:10, flow rate = 1.0 mL/min, λ = 254 nm) t_R = 6.4 min (major), 11.7 min (minor).

^{1}H NMR (400 MHz, $CDCl_3$) δ 9.14 (s, 1H), 7.76 (s, 1H), 7.49-7.44 (m, 2H), 7.39-7.24 (m, 4H), 6.97 (s, 2H), 5.54 (s, 1H), 5.08 (s, 1H), 1.29 (s, 18H). **^{13}C NMR (100 MHz, $CDCl_3$)** δ 160.5, 152.8, 151.3, 143.9, 136.1, 135.4, 134.4, 131.7, 131.1, 129.2, 128.8, 126.6, 126.2, 123.7, 123.2, 123.0, 121.5, 120.8 (q, *J* = 256.2 Hz), 113.6, 112.9, 46.0, 34.4, 30.1. **^{19}F-NMR (376 MHz, $CDCl_3$)** δ = -58.1. **HRMS (ESI)** calculated $[M+Na]^+$ for $C_{31}H_{30}F_3NO_4Na$: 560.2019, found: 560.2025. **FTIR (cm^{-1})** 3640, 3402, 2963, 2854, 1692, 1434, 1265, 1078, 865.

(*S*)-10-Bromo-12-(3,5-di-*tert*-butyl-4-hydroxyphenyl)-7,12-dihydro-6*H*-benzo [6,7]oxepino[3,4-*b*]indol-6-one (3h)

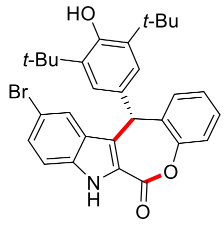

3h

Following the general procedure, treatment of 5-bromo-1*H*-indole-2-carbaldehyde **1h** (56.0 mg, 0.25mmol) and 2,6-di-*tert*-butyl-4-(2-hydroxybenzylidene)cyclohexa-2,5-dien-1-one **2a** (108.6 mg, 0.35 mmol) with triazolium salt **4** (23.2 mg, 0.05 mmol), Cs_2CO_3 (49 mg, 0.15 mmol), oxidant **8** (204 mg, 0.5 mmol), $Bi(OTf)_3$ (33.0 mg, 0.05mmol) in toluene (4.0 mL) and stirring the reaction mixture at 18 °C for 36 h followed by flash column chromatography (Pet. ether -EtOAc: 98:2 to 90:10) to afford (*S*)-10-Bromo-12-(3,5-di-*tert*-butyl-4-hydroxyphenyl)-7,12-dihydro-6*H*-benzo [6,7]oxepino[3,4-*b*]indol-6-one (**3h**) as a

white solid (85 mg, 64 % yield with 91:9 er). ***R**$_f$* (Pet. ether /EtOAc = 90/10): 0.40; er = 91:9, $[\alpha]_D^{25}$ = +29.61 (c 1.0, $CHCl_3$). **HPLC** (Chiralpak IA, *n*-hexane/IPA = 90:10, flow rate = 1.0 mL/min, λ = 254 nm) t_R = 7.3 min (major), 14.0 min (minor).

^{1}H NMR (400 MHz, $CDCl_3$) δ 9.11 (s, 1H), 8.03 (s, 1H), 7.49-7.47 (m, 2H), 7.36-7.29 (m, 3H), 7.24-7.23 (m, 1H), 6.96 (s, 2H), 5.52 (s, 1H), 5.07 (s, 1H), 1.30 (s, 18H). **^{13}C NMR (100 MHz, $CDCl_3$)** δ 160.4, 152.8, 151.2, 136.0, 135.7, 134.7, 131.5, 130.8, 130.1, 128.7, 127.9, 127.7, 126.5, 123.7, 123.4, 123.0, 122.7, 114.4, 114.0, 46.0, 34.4, 30.2. **HRMS (ESI) m/z:** $[M+H]^+$ calcd for $C_{30}H_{31}BrNO_3$ 532.1482; found 532.1487. **FTIR (cm^{-1})** 3633, 3298, 2959, 2868, 1691, 1432, 1368, 1213, 1159, 1083, 943, 860.

(*S*)-10-Chloro-12-(3,5-di-*tert*-butyl-4-hydroxyphenyl)-7,12-dihydro-6*H*-benzo [6,7]oxepino[3,4-*b*]indol-6-one (3i)

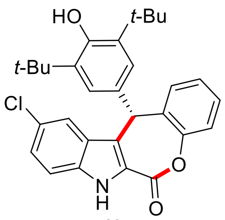

3i

Following the general procedure, treatment of 5-chloro-1*H*-indole-2-carbaldehyde **1i** (45.0 mg, 0.25mmol) and 2,6-di-*tert*-butyl-4-(2-hydroxybenzylidene)cyclohexa-2,5-dien-1-one **2a** (108.6 mg, 0.35 mmol) with triazolium salt **4** (23.2 mg, 0.05 mmol), Cs_2CO_3 (49 mg, 0.15 mmol), oxidant **8** (204 mg, 0.5 mmol), $Bi(OTf)_3$ (33.0 mg, 0.05mmol) in toluene (4.0 mL) and stirring the reaction mixture at 18 °C for 36 h followed by flash column chromatography (Pet. ether -EtOAc: 98:2 to 90:10) to afford (*S*)-10-chloro-12-(3,5-di-*tert*-butyl-4-hydroxyphenyl)-7,12-dihydro-6*H*-benzo[6,7]oxepino[3,4-*b*]indol-6-one (**3i**) as a white solid (87 mg, 71 % yield with 95:5 er).

***R**$_f$* (Pet. ether /EtOAc = 90/10): 0.41; er = 95:5, $[\alpha]_D^{25}$ = +7.92 (c 1.0, $CHCl_3$). **HPLC** (Chiralpak IA, *n*-hexane/IPA = 90:10, flow rate = 1.0 mL/min, λ = 254 nm) t_R = 8.4 min (major), 17.6 min (minor).

^{1}H NMR (400 MHz, $CDCl_3$) δ 9.06 (s, 1H), 7.86 (s, 1H), 7.48-7.30 (m, 6H), 6.96 (s, 2H), 5.52 (s, 1H), 5.07 (s, 1H), 1.30 (s, 18H). **^{13}C NMR (100 MHz, $CDCl_3$)** δ 160.4, 152.8, 151.2, 136.0, 135.5, 134.7, 131.5, 130.8, 128.7, 128.0, 127.7, 127.1, 127.0, 126.5, 123.7, 123.0, 122.9, 120.2, 113.6, 46.0, 34.4, 30.2. **HRMS (ESI) m/z:** $[M+H]^+$ calcd for $C_{30}H_{31}ClNO_3$ 488.1987; found 488.1992. **FTIR (cm^{-1})** 3629, 3292, 2954, 2913, 2858, 1692, 1458, 1430, 1369, 1295, 1233, 1176, 1019, 949, 859.

(*S*)-12-(3,5-Di-*tert*-butyl-4-hydroxyphenyl)-10-fluoro-7,12-dihydro-6*H*-benzo[6,7]oxepino[3,4-b]indol-6-one (3j)

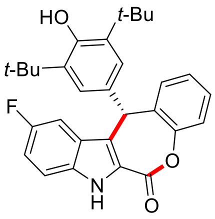

3j

Following the general procedure, treatment of 5-fluoro-1*H*-indole-2-carbaldehyde **1j** (41.0 mg, 0.25mmol) and 2,6-di-*tert*-butyl-4-(2-hydroxybenzylidene)cyclohexa-2,5-dien-1-one **2a** (108.6 mg, 0.35 mmol) with triazolium salt **4** (23.2 mg, 0.05 mmol), Cs_2CO_3 (49 mg, 0.15 mmol), oxidant **8** (204 mg, 0.5 mmol), $Bi(OTf)_3$ (33.0 mg, 0.05mmol) in toluene (4.0 mL) and stirring the reaction mixture at 18 °C for 36 h followed by flash column chromatography (Pet. ether -EtOAc: 98:2 to 90:10) to afford (*S*)-12-(3,5-Di-*tert*-butyl-4-hydroxyphenyl)-10-fluoro-7,12-dihydro-6*H*-benzo[6,7]oxepino[3,4-b]indol-6-one (**3j**) as a white solid (96 mg, 81 % yield with 99:1 er). ***R_f*** (Pet. ether /EtOAc = 90/10): 0.36; er = 99:1, $[\alpha]_D^{25}$ = +45.58 (c 1.0, $CHCl_3$). **HPLC** (Chiralpak IA, *n*-hexane/IPA = 90:10, flow rate = 1.0 mL/min, λ = 254 nm) t_R = 8.2 min (major), 24.1 min (minor).

^{1}H NMR (400 MHz, acetone-D6) δ 11.14 (s, 1H), 7.74-7.71 (m, 3H), 7.30-7.10 (m, 6H), 5.87 (s, 2H), 1.29 (s, 18H). **^{13}C NMR (100 MHz, acetone-D6)** δ 160.1, 159.1 (d, *J* = 235.7 Hz), 153.5, 152.4, 138.2, 138.1, 136.9, 135.4, 133.6, 131.7, 129.3, 128.7 (d, *J* = 5.7 Hz), 127.1, 127.0, 124.4, 123.4, 116.4 (d, *J* = 27.2 Hz), 115.1 (d, *J* = 27.2 Hz), 105.6 (d, *J* = 23.7 Hz), 46.0, 35.2, 30.6. **^{19}F-NMR (376 MHz, $CDCl_3$)** δ = -121.6. **HRMS (ESI) m/z:** $[M+H]^+$ calcd for $C_{30}H_{31}FNO_3$ 472.2282; found 472.2286. **FTIR (cm^{-1})** 3632, 3294, 2959, 2870, 2332, 1693, 1628, 1547, 1433, 1359, 1231, 1149, 1075.

(*S*)-12-(3,5-di-*Tert*-butyl-4-hydroxyphenyl)-9-methoxy-7,12-dihydro-6*H*-benzo[6,7]oxepino[3,4-b]indol-6-one (3k)

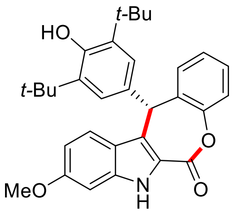

3k

Following the general procedure, treatment of 6-methoxy-1*H*-indole-2-carbaldehyde **1k** (43.8 mg, 0.25 mmol) and 2,6-di-*tert*-butyl-4-(2-hydroxybenzylidene)cyclohexa-2,5-dien-1-one **2a** (108.6 mg, 0.35 mmol) with triazolium salt **4** (23.2 mg, 0.05 mmol), Cs_2CO_3 (49.0 mg, 0.15 mmol), oxidant **8** (204.0 mg, 0.5 mmol), $Bi(OTf)_3$ (33.0 mg, 0.05mmol) in toluene (4.0 mL) and stirring the reaction mixture at 18 °C for 36 h followed by flash column chromatography (Pet. ether -EtOAc: 98:2 to 90:10) to afford (*S*)-12-(3,5-di-*tert*-butyl-4-hydroxyphenyl)-9-

methoxy-7,12-dihydro-6*H*-benzo[6,7]oxepino[3,4-b]indol-6-one (**3k**) as a white solid (73.5 mg, 61 % yield with 95:5 er).

***R**$_f$* (Pet. ether /EtOAc = 90/10): 0.20; er = 95:5, $[\alpha]_D^{25}$ = +36.8 (c 1.0, $CHCl_3$). **HPLC** (Chiralpak IA, *n*-hexane/IPA = 90:10, flow rate = 1.0 mL/min, λ = 254 nm) t_R = 22.2 min (major), 48.4 min (minor).

^{1}H NMR (400 MHz, $CDCl_3$) δ 9.21 (s, 1H), 7.79 (d, *J* = 8.9 Hz, 1H), 7.50 (d, *J* = 7.6 Hz, 1H), 7.40 (d, *J* = 8.0 Hz, 1H), 7.35-7.32 (m, 1H), 7.29-7.24 (m, 1H), 7.03 (s, 2H), 6.95-6.92 (m, 1H), 6.87 (s, 1H), 5.60 (s, 1H), 5.10 (s, 1H), 3.90 (s, 3H),1.33 (s, 18H). **^{13}C NMR (100 MHz, $CDCl_3$)** δ 160.8, 160.3, 152.6, 151.6, 138.8, 135.8, 134.7, 132.0, 130.9, 129.7, 128.5, 126.2, 123.8, 123.0, 121.7, 120.7, 120.2, 113.3, 93.7, 55.7, 46.1, 34.4, 30.2. **HRMS (ESI)** calculated $[M+H]^+$ for $C_{31}H_{34}NO_4$: 484.2482, found: 484.2483. **FTIR (cm^{-1})** 3635, 3318, 3071, 2961, 2870, 1683, 1627, 1433, 108, 759.

(*S*)-12-(3,5-Di-*tert*-butyl-4-hydroxyphenyl)-9-methyl-7,12-dihydro-6*H*-benzo[6,7]oxepino[3,4-*b*]indol-6-one (3l)

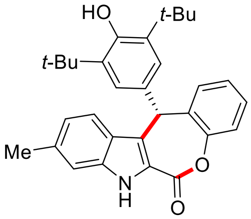

3l

Following the general procedure, treatment of 6-methyl-1*H*-indole-2-carbaldehyde **1l** (39.8 mg, 0.25 mmol) and 2,6-di-*tert*-butyl-4-(2-hydroxybenzylidene)cyclohexa-2,5-dien-1-one **2a** (108.6 mg, 0.35 mmol) with triazolium salt **4** (23.2 mg, 0.05 mmol), Cs_2CO_3 (49.0 mg, 0.15 mmol), oxidant **8** (204.0 mg, 0.5 mmol), $Bi(OTf)_3$ (33.0 mg, 0.05mmol) in toluene (4.0 mL) and stirring the reaction mixture at 18 °C for 36 h followed by flash column chromatography (Pet. ether -EtOAc: 98:2 to 90:10) to afford (*S*)-12-(3,5-di-*tert*-butyl-4-hydroxyphenyl)-9-methyl-7,12-dihydro-6*H*-benzo[6,7]oxepino[3,4-*b*]indol-6-one (**3l**) as a white solid (89.0 mg, 78% yield with >99:1 er). ***R**$_f$* (Pet. ether /EtOAc = 90/10): 0.41; er = >99:1, $[\alpha]_D^{25}$ = +46.42 (c 1.0, $CHCl_3$). **HPLC** (Chiralpak IA, *n*-hexane/IPA = 90:10, flow rate = 1.0 mL/min, λ = 254 nm) t_R = 24.6 min (major), 42.9 min (minor).

^{1}H NMR (400 MHz, $CDCl_3$) δ 8.82 (s, 1H), 7.77-7.75 (m, 1H), 7.47-7.45 (m, 1H), 7.30-7.30 (m, 1H), 7.21-7.21 (m, 3H), 7.08-7.06 (m, 1H), 6.98 (s, 2H), 5.57 (s, 1H), 5.04 (s, 1H), 2.49 (s, 3H), 1.29 (s, 18H). **^{13}C NMR (100 MHz, $CDCl_3$)** δ 160.8, 152.6, 151.5, 137.8, 137.7, 135.8, 134.8, 132.1, 130.9, 129.1, 128.5, 126.2, 124.3, 123.8, 123.4, 123.0, 120.9, 120.4, 111.9, 46.1, 34.4, 30.2,

22.2. **HRMS (ESI)** $[M+H]^+$ calcd for $C_{31}H_{34}NO_3$: 468.2533, found: 468.2535. **FTIR (cm^{-1})** 3583, 3322, 3077, 2923, 2852, 1716, 1544, 1433, 1326, 1233, 1081, 1022.

(*S*)-9-Bromo-12-(3,5-di-*tert*-butyl-4-hydroxyphenyl)-7,12-dihydro-6*H*-benzo[6,7]oxepino[3,4-*b*]indol-6-one (3m)

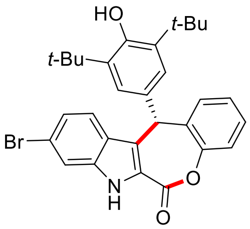

3m

Following the general procedure, treatment of 6-bromo-1*H*-indole-2-carbaldehyde **1m** (56.0 mg, 0.25 mmol) and 2,6-di-*tert*-butyl-4-(2-hydroxybenzylidene)cyclohexa-2,5-dien-1-one **2a** (108.6 mg, 0.35 mmol) with triazolium salt **4** (23.2 mg, 0.05 mmol), Cs_2CO_3 (49.0 mg, 0.15 mmol), oxidant **8** (204.0 mg, 0.5 mmol), $Bi(OTf)_3$ (33.0 mg, 0.05mmol) in toluene (4.0 mL) and stirring the reaction mixture at 18 °C for 36 h followed by flash column chromatography (Pet. ether -EtOAc: 98:2 to 90:10) to afford (*S*)-9-Bromo-12-(3,5-di-*tert*-butyl-4-hydroxyphenyl)-7,12-dihydro-6*H*-benzo[6,7]oxepino[3,4-*b*]indol-6-one (**3m**) as a white solid (101.0 mg, 76% yield with 96:4 er). R_f (Pet. ether /EtOAc = 90/10): 0.46; er = 96:4, $[\alpha]_D^{25}$ = +45.84 (c 1.0, $CHCl_3$). **HPLC** (Chiralpak IA, *n*-hexane/IPA = 90:10, flow rate = 1.0 mL/min, λ = 254 nm) t_R = 27.1 min (major), 33.8 min (minor).

^{1}H NMR (400 MHz, $CDCl_3$) δ 8.95 (s, 1H), 7.76 (d, *J* = 8.77 Hz, 1H), 7.62 (s, 1H), 7.46 (d, *J* = 7.51 Hz, 1H), 7.36-7.30 (m, 3H), 7.25-7.21 (m, 1H), 6.94 (s, 2H), 5.5 (s, 1H), 5.07 (s, 1H), 1.29 (s, 18H). **^{13}C NMR (100 MHz, $CDCl_3$)** δ 160.4, 152.8, 151.3, 137.7, 136.0, 134.6, 131.7, 130.9, 129.0, 128.7, 126.4, 125.1, 124.9, 123.7, 123.0, 122.2, 122.1, 121.2, 115.3, 45.9, 34.4, 30.2. **HRMS (ESI) m/z:** $[M+H]^+$ calcd for $C_{30}H_{31}BrNO_3$ 532.1482; found 532.1487. **FTIR (cm^{-1})** 3745, 3617, 3586, 3290, 2920, 2866, 2364, 1836, 1742, 1695, 1539, 1429, 1218, 1044.

(*S*)-12-(3,5-Di-*tert*-butyl-4-hydroxyphenyl)-9-fluoro-7,12-dihydro-6*H*-benzo[6,7]oxepino[3,4-*b*]indol-6-one (3n)

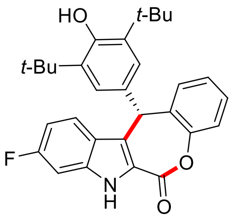

3n

Following the general procedure, treatment of 6-fluoro-1*H*-indole-2-carbaldehyde **1n** (40.8 mg, 0.25 mmol) and 2,6-di-*tert*-butyl-4-(2-hydroxybenzylidene)cyclohexa-2,5-dien-1-one **2a** (108.6 mg, 0.35 mmol) with triazolium salt **4** (23.2 mg, 0.05 mmol), Cs_2CO_3 (49.0 mg, 0.15 mmol), oxidant **8** (204.0 mg, 0.5 mmol), $Bi(OTf)_3$ (33.0 mg, 0.05mmol) in toluene (4.0 mL) and stirring the reaction mixture at 18 °C for 36 h followed by flash

column chromatography (Pet.ether-EtOAc: 98:2 to 90:10) to afford (*S*)-12-(3,5-di-*tert*-butyl-4-hydroxyphenyl)-9-fluoro-7,12-dihydro-6*H*-benzo[6,7]oxepino[3,4-*b*]indol-6-one (**3n**) as a white solid (86.0 mg, 72% yield with 83:17 er). ***Rf*** (Pet. ether /EtOAc = 90/10): 0.35; er = 83:17, $[\alpha]_D^{25}$ = +181.28 (c 1.0, $CHCl_3$). **HPLC** (Chiralpak IA, *n*-hexane/IPA = 90:10, flow rate = 1.0 mL/min, λ = 254 nm) t_R = 8.2 min (minor), 13.5 min (major).

^{1}H NMR (400 MHz, $CDCl_3$) δ 9.09 (s, 1H), 7.48 (d, *J* = 7.31 Hz, 1H), 7.38-7.27 (m, 3H), 7.24-7.18 (m, 2H), 7.10 (s, 2H), 6.88-6.84 (m, 1H), 5.92 (s, 1H), 5.06 (s, 1H), 1.31 (s, 18H). **^{13}C NMR (100 MHz, $CDCl_3$)** δ 160.5, 158.5 (d, *J* = 250.7 Hz), 152.7, 151.3, 139.2 (d, *J* =9.2 Hz), 135.9, 134.8, 132.4, 131.3, 128.6, 127.7 (d, *J* =4.4 Hz), 127.5 (d, *J* =8.3 Hz), 126.5, 124.0, 122.8, 121.7, 115.7 (d, *J* = 18.1 Hz), 108.6 (d, *J* = 4.1 Hz), 106.0 (d, *J* = 19.2 Hz), 47.2, 34.4, 30.2. **^{19}F-NMR (376 MHz, $CDCl_3$)** δ = -121.0. **HRMS (ESI) m/z:** $[M+H]^+$ calcd for $C_{30}H_{31}FNO_3$: 472.2282, found: 472.2289. **FTIR (cm^{-1})** 3631, 3295, 2912, 2009, 1692, 1608, 1428, 1239, 1110, 1051, 902, 733.

(*S*)-12-(3,5-Di-*tert*-butyl-4-hydroxyphenyl)-9-phenyl-7,12-dihydro-6*H* benzo[6,7]oxepino[3,4-*b*]indol-6-one (3o)

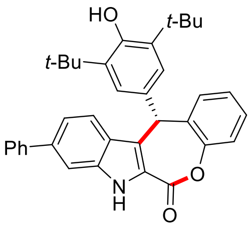

3o

Following the general procedure, treatment of 6-phenyl-1*H*-indole-2-carbaldehyde **1o** (55.3 mg, 0.25 mmol) and 2,6-di-*tert*-butyl-4-(2-hydroxybenzylidene)cyclohexa-2,5-dien-1-one **2a** (108.6 mg, 0.35 mmol) with triazolium salt **4** (23.2 mg, 0.05 mmol), Cs_2CO_3 (49.0 mg, 0.15 mmol), oxidant **8** (204.0 mg, 0.5 mmol), $Bi(OTf)_3$ (33.0 mg, 0.05mmol) in toluene (4.0 mL) and stirring the reaction mixture at 18 °C for 36 h followed by flash column chromatography (Pet. ether -EtOAc: 98:2 to 90:10) to afford (*S*)-12-(3,5-di-*tert*-butyl-4-hydroxyphenyl)-9-phenyl-7,12-dihydro-6*H*-benzo[6,7]oxepino[3,4-*b*]indol-6-one (**3o**) as a white solid (98 mg, 74 % yield with 94:6 er). ***R_f*** (Pet. ether /EtOAc = 90/10): 0.41; er = 94:6, $[\alpha]_D^{25}$ = +25.48 (c 1.0, $CHCl_3$). **HPLC** (Chiralpak IA, *n*-hexane/IPA = 90:10, flow rate = 1.0 mL/min, λ = 254 nm) t_R = 24.8 min (major), 59.3 min (minor).

^{1}H NMR (400 MHz, $CDCl_3$) δ 9.09 (s, 1H), 7.95 (d, *J* = 8.56 Hz, 1H), 7.68-7.63 (m, 3H), 7.53-7.45 (m, 4H), 7.39-7.30 (m, 3H), 7.24-7.22 (m, 1H), 7.03 (s, 2H), 5.64 (s, 1H), 5.06 (s, 1H), 1.31 (s, 18H). **^{13}C NMR (100 MHz, $CDCl_3$)** δ 160.6, 152.7, 151.5, 141.4, 140.6, 137.9, 136.0, 134.9, 132.0, 130.9, 129.0, 129.0, 128.6, 127.7, 127.6, 126.3, 125.6, 123.8, 123.0, 122.0, 121.4, 121.1,

110.5, 46.2, 34.4, 30.3. **HRMS (ESI) m/z:** $[M+H]^+$ calcd for $C_{36}H_{36}NO_3$ 530.2690; found 530.2691. **FTIR (cm^{-1})** 3618, 3337, 2918, 2861, 2237, 1684, 1510, 1429, 1358, 1239, 1142, 904, 867.

(*S*)-12-(3,5-Di-*tert*-butyl-4-hydroxyphenyl)-9-(2-fluorophenyl)-7,12-dihydro-6*H*-benzo [6,7] oxepino[3,4-*b*] indol-6-one (3p)

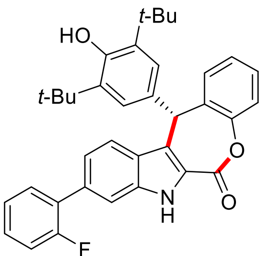

3p

Following the general procedure, treatment of 6-(2-fluorophenyl)-1*H*-indole-2-carbaldehyde **1p** (59.8 mg, 0.25 mmol) and 2,6-di-*tert*-butyl-4-(2-hydroxybenzylidene)cyclohexa-2,5-dien-1-one **2a** (108.6 mg, 0.35 mmol) with triazolium salt **4** (23.2 mg, 0.05 mmol), Cs_2CO_3 (49.0 mg, 0.15 mmol), oxidant **8** (204.0 mg, 0.5 mmol), $Bi(OTf)_3$ (33.0 mg, 0.05mmol) in toluene (4.0 mL) and stirring the reaction mixture at 18 °C for 36 h followed by flash column chromatography (Pet. ether -EtOAc: 98:2 to 90:10) to afford (*S*)-12-(3,5-di-*tert*-butyl-4-hydroxyphenyl)-9-(2-fluorophenyl)-7,12-dihydro-6*H*-benzo [6,7] oxepino[3,4-*b*] indol-6-one (**3p**) as a yellow solid (91.7 mg, 67 % yield with 95:5 er).

Rf (Pet. ether /EtOAc = 90/10): 0.23; er = 95:5, $[\alpha]_D^{25}$ = +51.08 (c 1.0, $CHCl_3$). **HPLC** (Chiralpak IA, *n*-hexane/IPA = 90:10, flow rate = 1.0 mL/min, λ = 254 nm) t_R = 23.2 min (major), 43.4 (minor).

^{1}H NMR (400 MHz, $CDCl_3$) δ 9.13 (s, 1H), 7.95 (d, *J* = 8.6 Hz, 1H), 7.63 (s, 1H), 7.53-7.44 (m, 3H), 7.39-7.30 (m, 3H), 7.26-7.16 (m, 3H), 7.04 (s, 2H), 5.64 (s, 1H), 5.07 (s, 1H), 1.31 (s, 18H). **^{13}C NMR (100 MHz, $CDCl_3$)** δ 160.6, 159.9 (d, *J* = 248.0 Hz), 152.7, 151.4, 137.4, 135.9, 134.8, 131.9, 131.1(d, *J* = 3.6 Hz), 130.9, 129.4, 129.3, 129.2, 128.8, 128.6, 126.4, 125.6, 124.6 (d, *J* = 3.6 Hz), 123.8, 123.0, 122.8 (d, *J* = 2.2 Hz), 122.2, 120.7, 116.4 (d, *J* = 22.8 Hz), 112.8 (d, *J* = 3.0 Hz) 46.2, 34.4, 30.2. **^{19}F-NMR (376 MHz, $CDCl_3$)** δ = -117.5. **HRMS (ESI)** calculated $[M+H]^+$ for $C_{36}H_{35}FNO_3$: 548.2595, found: 548.2601. **FTIR (cm^{-1})** 3635, 3329, 2961, 2870, 1686, 1435, 1236, 1080, 872.

(*S*)-12-(3,5-di-*Tert*-butyl-4-hydroxyphenyl)-9-(2,6-dimethylphenyl)-7,12-dihydro-6*H*-benzo[6,7]oxepino[3,4-*b*]indol-6-one (3q)

Following the general procedure, treatment of 6-(2,6-dimethylphenyl)-1*H*-indole-2-carbaldehyde **1q** (62.3 mg, 0.25 mmol) and 2,6-di-*tert*-butyl-4-(2-hydroxybenzylidene)cyclohexa-2,5-dien-1-

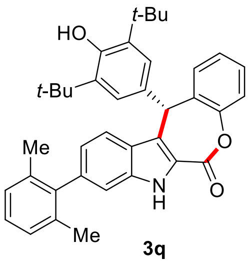

3q

one **2a** (108.6 mg, 0.35 mmol) with triazolium salt **4** (23.2 mg, 0.05 mmol), Cs_2CO_3 (49.0 mg, 0.15 mmol), oxidant **8** (204.0 mg, 0.5 mmol), $Bi(OTf)_3$ (33.0 mg, 0.05mmol) in toluene (4.0 mL) and stirring the reaction mixture at 18 °C for 36 h followed by flash column chromatography (Pet. ether - EtOAc: 98:2 to 90:10) to afford (*S*)-12-(3,5-di-*tert*-butyl-4-hydroxyphenyl)-9-(2,6-dimethylphenyl)-7,12-dihydro-6*H*-benzo[6,7]oxepino[3,4-*b*]indol-6-one (**3q**)as a white solid (93.1 mg, 67 % yield with 96:4 er).

Rf (Pet. ether /EtOAc = 90/10): 0.25; er = 96:4, $[\alpha]_D^{25}$ = +19.8 (c 0.1, $CHCl_3$). **HPLC** (Chiralpak IA, *n*-hexane/IPA = 90:10, flow rate = 1.0 mL/min, λ = 254 nm) t_R = 13.9 min (minor), 24.0 min (major).

^{1}H NMR (400 MHz, $CDCl_3$) δ 9.27 (s, 1H), 7.96 (d, *J* = 8.3 Hz, 1H), 7.48 (d, *J* = 7.3 Hz, 1H), 7.38 (d, *J* = 7.8 Hz, 1H), 7.31 (t, *J* = 7.2 Hz, 1H), 7.23-7.11 (m, 5H), 7.04 (s, 3H), 5.66 (s, 1H), 5.07 (s, 1H), 2.02 (s, 6H), 1.29 (s, 18H). **^{13}C NMR (100 MHz, $CDCl_3$)** δ 160.8, 152.7, 151.5, 141.8, 140.5, 137.7, 136.2, 136.2, 135.9, 134.7, 132.1, 131.0, 129.2, 128.6, 127.5, 127.5, 127.4, 126.4, 125.1, 123.8, 123.0, 121.5, 120.9, 112.5, 46.2, 34.4, 30.2, 20.9, 20.9. **HRMS (ESI)** calculated $[M+H]^+$ for $C_{38}H_{40}NO_3$: 558.3003, found: 558.3007. **FTIR (cm^{-1})** 3452, 3396, 2920, 2868, 1869, 1626, 1434, 1027, 898.

(*S*)-12-(3,5-Di-*tert*-butyl-4-hydroxyphenyl)-9-(2,3-dimethylphenyl)-7,12-dihydro-6*H*-benzo [6,7] oxepino[3,4-*b*] indol-6-one (3r)

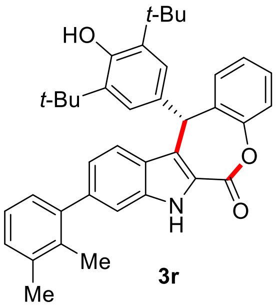

3r

Following the general procedure, treatment of 6-(2,3-dimethylphenyl)-1*H*-indole-2-carbaldehyde **1r** (62.3 mg, 0.25 mmol) and 2,6-di-*tert*-butyl-4-(2-hydroxybenzylidene)cyclohexa-2,5-dien-1-one **2a** (108.6 mg, 0.35 mmol) with triazolium salt **4** (23.2 mg, 0.05 mmol), Cs_2CO_3 (49.0 mg, 0.15 mmol), oxidant **8** (204.0 mg, 0.5 mmol), $Bi(OTf)_3$ (33.0 mg, 0.05mmol) in toluene (4.0 mL) and stirring the reaction mixture at 18 °C for 36 h followed by flash column chromatography (Pet. ether -EtOAc: 98:2 to 90:10) to afford (*S*)-12-(3,5-di-*tert*-butyl-4-hydroxyphenyl)-9-(2,3-dimethylphenyl)-7,12-dihydro-6*H*-benzo [6,7] oxepino[3,4-*b*] indol-6-one (**3r**) as a white solid (94.7 mg, 68 % yield with 95:5 er).

R_f (Pet. ether /EtOAc = 90/10): 0.25; er = 95:5, $[\alpha]_D^{25}$ = +36.46 (c 1.0, $CHCl_3$). **HPLC** (Chiralpak IA, *n*-hexane/IPA = 95:5, flow rate = 1.0 mL/min, λ = 254 nm) t_R = 40.7 min (major), 47.7 min (minor).

^{1}H NMR (400 MHz, $CDCl_3$) δ 8.97 (s, 1H), 7.91 (d, *J* = 8.4 Hz, 1H), 7.48 (d, *J* = 7.3 Hz, 1H), 7.39-7.30 (m, 3H), 7.23-7.12 (m, 5H), 7.05 (s, 2H), 5.64 (s, 1H), 5.06 (s, 1H), 2.36 (s, 3H), 2.15 (s, 3H), 1.31 (s, 18H). **^{13}C NMR (100 MHz, $CDCl_3$)** δ 160.8, 152.7, 151.4, 142.3, 142.0, 137.4, 137.3, 135.9, 134.8, 134.2, 132.1, 130.9, 129.2, 129.0, 128.5, 127.8, 126.3, 125.4, 124.9, 123.9, 123.6, 123.0, 121.7, 120.1, 112.8, 46.3, 34.4, 30.2, 20.8, 17.2. **HRMS (ESI)** calculated $[M+H]^+$ for $C_{38}H_{40}NO_3$: 558.3003, found: 548.3009. **FTIR (cm^{-1})** 3637, 3397, 2962, 2868, 1688, 1433, 1236, 1098, 870.

(*S*)-12-(3,5-Di-*tert*-butyl-4-hydroxyphenyl)-8-methoxy-7,12-dihydro-6*H*-benzo[6,7]oxepino[3,4-*b*]indol-6-one (3s)

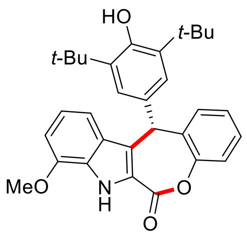

3s

Following the general procedure, treatment of 1*H*-indole-2-carbaldehyde **1s** (43.8 mg, 0.25 mmol) and 2,6-di-*tert*-butyl-4-(2-hydroxybenzylidene) cyclohexa-2,5-dien-1-one **2a** (108.6 mg, 0.35 mmol) with triazolium salt **4** (23.2 mg, 0.05 mmol), Cs_2CO_3 (49.0 mg, 0.15 mmol), oxidant **8** (204.0 mg, 0.5 mmol), $Bi(OTf)_3$ (33.0 mg, 0.05mmol) in toluene (4.0 mL) and stirring the reaction mixture at 18 °C for 36 h followed by flash column chromatography (Pet. ether -EtOAc: 98:2 to 90:10) to afford (*S*)-12-(3,5-di-*tert*-butyl-4-hydroxyphenyl)-8-methoxy-7,12-dihydro-6*H*-benzo[6,7]oxepino[3,4-*b*]indol-6-one (**3s**) as a white solid (74.0 mg, 61% yield with 96:4 er). ***R_f*** (Pet. ether /EtOAc = 85/15): 0.36; er = 96:4, $[\alpha]_D^{25}$ = +51.12 (c 1.0, $CHCl_3$). **HPLC** (Chiralpak IA, *n*-hexane/IPA = 90:10, flow rate = 1.0 mL/min, λ = 254 nm) t_R = 8.8 min (minor), 33.8 min (major).

^{1}H NMR (400 MHz, $CDCl_3$) δ 9.07 (s, 1H), 7.48-7.46 (m, 2H), 7.38-7.36 (m, 1H), 7.32-7.31 (m, 1H), 7.24-7.22 (m, 1H), 7.17-7.15 (m, 1H), 6.99 (s, 2H), 6.88 (d, *J* = 7.56 Hz, 1H) 5.58 (s, 1H), 5.04 (s, 1H), 3.97 (s, 3H), 1.29 (s, 18H). **^{13}C NMR (100 MHz, $CDCl_3$)** δ 160.5, 152.6, 151.5, 146.8, 135.9, 134.9, 132.1, 130.9, 129.1, 128.7, 128.5, 127.5, 126.2, 123.8, 123.0, 121.7, 121.2, 112.9, 105.6, 55.7, 46.3, 34.4, 30.2. **HRMS (ESI) m/z:** $[M+H]^+$ calcd for $C_{31}H_{34}NO_4$ 484.2482, found: 484.2488. **FTIR (cm^{-1})** 3634, 3321, 3007, 2364, 1704, 2861, 1580, 1392, 1259, 1113, 983, 856.

(*S*)-7-(3,5-Di-*tert*-butyl-4-hydroxyphenyl)-7,14-dihydro-13*H*-benzo[*g*]benzo[6,7] oxepino[3,4-*b*]indol-13-one (3t)

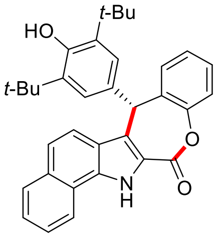

3t

Following the general procedure, treatment of 1H-benzo[*g*]indole-2-carbaldehyde **1t** (48.8 mg, 0.25 mmol) and 2,6-di-*tert*-butyl-4-(2-hydroxybenzylidene)cyclohexa-2,5-dien-1-one **2a** (108.6 mg, 0.35 mmol) with triazolium salt **4** (23.2 mg, 0.05 mmol), Cs_2CO_3 (49.0 mg, 0.15 mmol), oxidant **8** (204.0 mg, 0.5 mmol), $Bi(OTf)_3$ (33.0 mg, 0.05mmol) in toluene (4.0 mL) and stirring the reaction mixture at 18 °C for 36 h followed by flash column chromatography (Pet. ether -EtOAc: 98:2 to 90:10) to afford (*S*)-7-(3,5-Di-*tert*-butyl-4-hydroxyphenyl)-7,14-dihydro-13*H*-benzo[*g*]benzo[6,7]oxepino[3,4-*b*]indol-13-one (**3t**) as a white solid (77.6 mg, 62 % yield with 99:1 er).

Rf (Pet. ether /EtOAc = 90/10): 0.25; er = 99:1, $[\alpha]_D^{25}$ = +36.1 (c 1.0, $CHCl_3$). **HPLC** (Chiralpak IA, *n*-hexane/IPA = 90:10, flow rate = 1.0 mL/min, λ = 254 nm) t_R = 8.8 min (minor), 9.7 min (major).

^{1}H NMR (400 MHz, $CDCl_3$) δ 10.19-10.12 (m, 1H), 8.24-8.20 (m, 1H), 7.93 (d, *J* = 7.9 Hz, 1H), 7.86 (d, *J* = 8.9 Hz, 1H), 7.62-7.51 (m, 4H), 7.40 (d, *J* = 7.9 Hz, 1H), 7.33 (t, *J* = 7.3 Hz, 1H), 7.27-7.24 (m, 1H), 7.02 (s, 2H), 5.70 (s, 1H), 5.06 (s, 1H), 1.29 (s, 18H). **^{13}C NMR (100 MHz, $CDCl_3$)** δ 160.6, 152.7, 151.7, 135.9, 134.8, 133.8, 132.9, 132.0, 131.0, 130.4, 129.2, 128.6, 126.7, 126.7, 126.3, 123.8, 123.1, 122.8, 122.4, 121.7, 121.0, 120.1, 118.9, 46.2, 34.4, 30.2. **HRMS (ESI)** calculated $[M+H]^+$ for $C_{34}H_{34}NO_3$: 504.2533, found: 504.2535. **FTIR (cm^{-1})** 3618, 3442, 2958, 2921, 2886, 1686, 1431, 1035, 1027, 859.

(*S*)-12-(3,5-di-*Tert*-butyl-4-hydroxyphenyl)-9,11-dimethyl-7,12-dihydro-6*H*-benzo[6,7]oxepino[3,4-b]indol-6-one (3u)

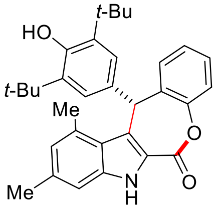

3u

Following the general procedure, treatment of 4,6-dimethyl-1*H*-indole-2-carbaldehyde **1u** (43.3 mg, 0.25 mmol) and 2,6-di-*tert*-butyl-4-(2-hydroxybenzylidene)cyclohexa-2,5-dien-1-one **2a** (108.6 mg, 0.35 mmol) with triazolium salt **4** (23.2 mg, 0.05 mmol), Cs_2CO_3 (49.0 mg, 0.15 mmol), oxidant **8** (204.0 mg, 0.5 mmol), $Bi(OTf)_3$ (33.0 mg, 0.05mmol) in toluene (4.0 mL) and stirring the reaction mixture at 18 °C for 36 h followed by flash column chromatography (Pet. ether -EtOAc: 98:2 to 90:10) to afford **(***S***)**-12-(3,5-di-*Tert*-butyl-4-hydroxyphenyl)-9,11-dimethyl-

7,12-dihydro-6*H*-benzo[6,7]oxepino[3,4-b]indol-6-one (**3u**) as a white solid (84.1 mg, 70 % yield with 94:6 er). ***Rf*** (Pet. ether /EtOAc = 90/10): 0.27; er = 94:6, $[\alpha]_D^{25}$ = +70.5 (c 0.1, $CHCl_3$). **HPLC** (Chiralpak IA, *n*-hexane/IPA = 98:2, flow rate = 1.0 mL/min, λ = 254 nm) t_R = 10.6 min (minor), 23.4 min (major).

^{1}H NMR (400 MHz, $CDCl_3$) δ 8.97 (s, 1H), 7.45 (d, *J* = 7.0 Hz, 1H), 7.33-7.26 (m, 2H), 7.24-7.20 (m, 1H), 7.06 (s, 1H) 6.87 (s, 2H), 6.76 (s, 1H), 5.94 (s, 1H), 5.04 (s, 1H), 2.79 (s, 3H), 2.42 (s, 3H), 1.28 (s, 18H). **^{13}C NMR (100 MHz, $CDCl_3$)** δ 161.1, 152.5, 151.2, 138.3, 137.3, 135.8, 135.7, 132.6, 132.0, 130.7, 129.6, 128.4, 126.2, 125.4, 123.8, 123.1, 122.8, 121.3, 110.0, 47.2, 34.4, 30.2, 21.9, 20.8. **HRMS (ESI)** calculated $[M+H]^+$ for $C_{32}H_{36}NO_3$: 482.2690, found: 482.2692. **FTIR (cm^{-1})** 3452, 3418, 2960, 2866, 1683, 1433, 1230, 1149, 837.

(S)-2-Bromo-12-(3,5-di-*tert*-butyl-4-hydroxyphenyl)-7,12-dihydro-6*H*-benzo[6,7]oxepino[3,4-b]indol-6-one (3v)

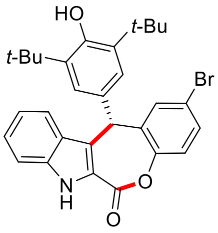

3v

Following the general procedure, treatment of 1*H*-indole-2-carbaldehyde **1a** (36.3 mg, 0.25mmol) and 4-(5-bromo-2-hydroxybenzylidene)-2,6-di-*tert*-butylcyclohexa-2,5-dien-1-one **2b** (136.3 mg, 0.35 mmol) with triazolium salt **4** (23.2 mg, 0.05 mmol), Cs_2CO_3 (49 mg, 0.15 mmol), oxidant **8** (204 mg, 0.5 mmol), $Bi(OTf)_3$ (33.0 mg, 0.05mmol) in toluene (4.0 mL) and stirring the reaction mixture at 10 °C for 48 h followed by flash column chromatography (Pet. ether -EtOAc: 98:2 to 90:10) to afford (*S*)-2-bromo-12-(3,5-di-*tert*-butyl-4-hydroxyphenyl)-7,12-dihydro-6*H*-benzo [6,7]oxepino[3,4-b]indol-6-one **(3v)** as a white solid (84 mg, 64 % yield with 94:6 er).

R_f (Pet. ether /EtOAc = 85/15): 0.37; er = 94:6, $[\alpha]_D^{25}$ = +73.95 (c 1.0, $CHCl_3$). **HPLC** (ChiralCell OD-H, *n*-hexane/IPA = 99:1, flow rate = 1.0 mL/min, λ = 254 nm) t_R = 24.3 min (major), 38.7 min (minor).

^{1}H NMR (400 MHz, $CDCl_3$) δ 8.92 (s, 1H), 7.88 (d, *J* = 8.16 Hz, 1H),7.62 (d, *J* =2.18 Hz,1H), 7.43-7.40 (m, 3H), 7.28-7.23 (m, 2H), 7.00 (s, 2H), 5.54 (s, 1H), 5.08 (s, 1H), 1.31 (s, 18H).**^{13}C NMR (100 MHz, $CDCl_3$)** δ 160.2, 152.9, 150.6, 137.4, 136.9, 136.1, 133.5, 131.4, 131.3, 128.4, 127.4, 126.0, 124.7, 123.7, 121.4, 121.1, 120.7, 119.1, 112.5, 45.9, 34.4, 30.2. **HRMS (ESI) m/z:** $[M+H]^+$ calcd for $C_{30}H_{31}BrNO_3$ 532.1482; found 532.1490. **FTIR (cm^{-1})** 3630, 3336, 2960, 2916, 2868, 2365, 2333, 1696, 1549, 1477, 1433, 1329, 1207, 1160, 1116, 885.

(*S*)-2-Chloro-12-(3,5-di-*tert*-butyl-4-hydroxyphenyl)-7,12-dihydro-6*H*-benzo[6,7]oxepino[3,4-b]indol-6-one (3w)

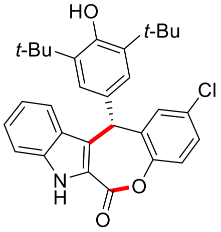

3w

Following the general procedure, treatment of 1*H*-indole-2-carbaldehyde **1** (36.3 mg, 0.25mmol) and 2,6-di-*tert*-butyl-4-(5-chloro-2-hydroxybenzylidene) cyclohexa-2,5-dien-1-one **2** (120.7 mg, 0.35 mmol) with triazolium salt **4** (23.2 mg, 0.05 mmol), Cs_2CO_3 (49 mg, 0.15 mmol), oxidant **8** (204 mg, 0.5 mmol), $Bi(OTf)_3$ (33.0 mg, 0.05mmol) in toluene (4.0 mL) and stirring the reaction mixture at 10 °C for 48 h followed by flash column chromatography (Pet. ether -EtOAc: 98:2 to 90:10) to afford (*S*)-2-chloro-12-(3,5-di-*tert*-butyl-4-hydroxyphenyl)-7,12-dihydro-6*H*-benzo[6,7] oxepino [3,4-b] indol-6-one **(3w)** as a white solid (82 mg, 67 % yield with 91:9 er). R_f (Pet. ether/EtOAc = 90/10): 0.40; er = 91:9, $[\alpha]_D^{25}$ = +68.72 (c 1.0, $CHCl_3$). **HPLC** (ChiralCell OD-H, *n*-hexane/IPA = 99:1, flow rate = 1.0 mL/min, λ = 254 nm) t_R = 23.9 min (major), 37.9 min (minor).

^{1}H NMR (400 MHz, $CDCl_3$) δ 8.97 (s, 1H), 7.88 (d, *J* = 8.12 Hz, 1H), 7.48-7.42 (m, 3H), 7.31-7.24 (m, 3H), 7.01 (s, 2H), 5.55 (s, 1H), 5.08 (s, 1H), 1.31 (s, 18H). **^{13}C NMR (100 MHz, $CDCl_3$)** δ 160.2, 152.9, 150.0, 137.3, 136.5, 136.1, 131.4, 130.5, 128.4, 128.3, 127.4, 126.1, 124.4, 123.7, 121.4, 121.1, 120.7, 112.5, 46.0, 34.4, 30.2. **HRMS (ESI) m/z:** $[M+H]^+$ calcd for $C_{30}H_{31}ClNO_3$ 488.1987; found 488.1990. **FTIR (cm^{-1})** 3632, 3336, 2960, 2367, 1695, 1549, 1480, 1433, 1234, 1160, 1077, 870.

(*S*)-12-(3,5-Di-*tert*-butyl-4-hydroxyphenyl)-2-phenyl-7,12-dihydro-6*H*-benzo [6,7]oxepino[3,4-b]indol-6-one (3x)

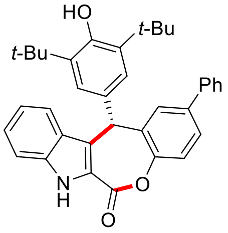

3x

Following the general procedure, treatment of 1*H*-indole-2-carbaldehyde **1a** (36.3 mg, 0.25mmol) and 2,6-di-*tert*-butyl-4-((4-hydroxy-[1,1'-biphenyl]-3-yl)methylene)cyclohexa-2,5-dien-1-one **2d** (135.3 mg, 0.35 mmol) with triazolium salt **4** (23.2 mg, 0.05 mmol), Cs_2CO_3 (49 mg, 0.15 mmol), oxidant **8** (204 mg, 0.5 mmol), $Bi(OTf)_3$ (33.0 mg, 0.05mmol) in toluene (4.0 mL) and stirring the reaction mixture at 18 °C for 36 h followed by flash column chromatography (Pet. ether -EtOAc: 98:2 to 90:10) to afford (*S*)-12-(3,5-di-*tert*-butyl-4-hydroxyphenyl)-2-Phenyl-7,12-dihydro-6*H*-benzo[6,7]oxepino[3,4-b]indol-6-one (**3x**) as a white solid (94 mg, 71 % yield with 93:7 er). R_f (Pet. ether /EtOAc = 85/115): 0.50; er = 93:7, $[\alpha]_D^{25}$ = +95.16 (c 1.0, $CHCl_3$). **HPLC**

(Chiralpak IA, *n*-hexane/IPA = 90:10, flow rate = 1.0 mL/min, λ = 254 nm) t_R = 16.8 min (minor), 25.4 min (major).

^{1}H NMR (400 MHz, $CDCl_3$) δ 8.98 (s, 1H), 7.93 (d, *J* = 7.93 Hz, 1H), 7.69-7.36 (m, 11H), 7.07 (s, 2H), 5.69 (s, 1H), 5.07 (s, 1H), 1.31 (s, 18H). **^{13}C NMR (100 MHz, $CDCl_3$)** δ 160.7, 152.7, 150.8, 140.0, 139.4, 137.3, 135.9, 135.0, 132.0, 129.5, 129.0, 128.9, 127.7, 127.2, 127.1, 126.2, 123.8, 123.4, 121.4, 121.3, 120.8, 112.4, 46.4, 34.4, 30.2. **HRMS (ESI) m/z:** $[M+H]^+$ calcd for $C_{36}H_{36}NO_3$ 530.2690; found 530.2695, **FTIR (cm^{-1})** 3634, 3333, 2959, 2869, 2362, 2330, 1693, 1329, 1207, 1119, 1081, 889.

(*S*)-12-(3,5-Di-*tert*-butyl-4-hydroxyphenyl)-2-methoxy-7,12-dihydro-6*H*-benzo [6,7]oxepino[3,4-b]indol-6-one (3y)

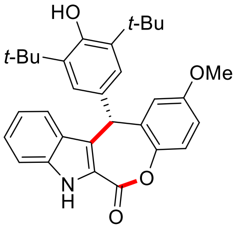

3y

Following the general procedure, treatment of 1*H*-indole-2-carbaldehyde **1a** (36.3 mg, 0.25mmol) and 2,6-di-*tert*-butyl-4-(2-hydroxy-5 methoxybenzylidene)cyclohexa-2,5-dien-1-one **2e** (119.2 mg, 0.35 mmol) with triazolium salt **4** (23.2 mg, 0.05 mmol), Cs_2CO_3 (49 mg, 0.15 mmol), oxidant **8** (204 mg, 0.5 mmol), $Bi(OTf)_3$ (33.0 mg, 0.05mmol) in toluene (4.0 mL) and stirring the reaction mixture at 18 °C for 36 h followed by flash column chromatography (Pet. ether -EtOAc: 98:2 to 95:15) to afford (*S*)-12-(3,5-di-*tert*-butyl-4-hydroxyphenyl)-2-methoxy-7,12-dihydro-6*H*-benzo[6,7]oxepino[3,4-b]indol-6-one (**3y**) as a white solid (88 mg, 73% yield with 95:5 er). ***R_f*** (Pet. ether /EtOAc = 85/15): 0.45; er = 95:5, $[\alpha]_D^{25}$ = +80.66 (c 1.0, $CHCl_3$). **HPLC** (Chiralpak IA, *n*-hexane/IPA = 90:10, flow rate = 1.0 mL/min, λ = 254 nm) t_R = 38.9 min (major), 52.6 min (minor).

^{1}H NMR (400 MHz, $CDCl_3$) δ 8.87 (s, 1H), 7.90 (d, *J* = 7.49 Hz, 1H), 7.42-7.38 (m, 2H), 7.30-7.28 (m, 2H), 7.02-6.97 (m, 3H), 6.82-6.80 (m, 1H), 5.53 (s, 1H), 5.05 (s, 1H), 3.82 (s, 3H), 1.30 (s, 18H). **^{13}C NMR (100 MHz, $CDCl_3$)** δ 161.1, 157.4, 152.7, 145.3, 137.1, 135.9, 135.8, 131.9, 128.7, 127.1, 126.2, 123.8, 121.5, 121.2, 120.7, 115.7, 113.1, 112.4, 55.8, 46.3, 34.4, 30.2. **HRMS (ESI) m/z:** $[M+H]^+$ calcd for $C_{31}H_{34}NO_4$ 484.2482; found 484.2487. **FTIR (cm^{-1})** 2960, 2920, 2362, 1689, 1547, 1494, 1432, 1231, 1183, 1081, 1038, 871.

(*S*)-12-(3,5-Di-*tert*-butyl-4-hydroxyphenyl)-3-methyl-7,12-dihydro-6*H*-benzo[6,7]oxepino[3,4-b]indol-6-one (3z)

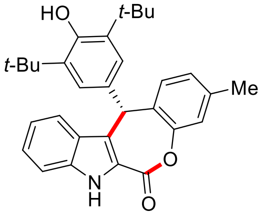

3z

Following the general procedure, treatment of 1*H*-indole-2-carbaldehyde **1a** (36.3 mg, 0.25mmol) and 2,6-di-*tert*-butyl-4-(2-hydroxy-4-methylbenzylidene)cyclohexa-2,5-dien-1-one **2f** (113.6 mg, 0.35 mmol) with triazolium salt **4** (23.2 mg, 0.05 mmol), Cs_2CO_3 (49 mg, 0.15 mmol), oxidant **8** (204 mg, 0.5 mmol), $Bi(OTf)_3$ (33.0 mg, 0.05mmol) in toluene (4.0 mL) and stirring the reaction mixture at 18 °C for 36 h followed by flash column chromatography (Pet. ether -EtOAc: 98:2 to 90:10) to afford (*S*)-12-(3,5-di-*tert*-butyl-4-hydroxyphenyl)-3-methyl-7,12-dihydro-6*H*-benzo[6,7]oxepino[3,4-*b*]indol-6-one (**3z**) as a white solid (77 mg, 66 % yield with 94:6 er).

R_f (Pet. ether /EtOAc = 85/15): 0.50; er = 94:6, $[\alpha]_D^{25}$ = +26.0 (c 1.0, $CHCl_3$). **HPLC** (Chiralpak IA, *n*-hexane/IPA = 90:10, flow rate = 1.0 mL/min, λ = 254 nm) t_R = 15.1 min (major), 44.3 min (minor).

^{1}H NMR (400 MHz, $CDCl_3$) δ 9.07 (s, 1H), 7.90 (d, *J* = 8.09 Hz, 1H), 7.45-7.34 (m, 3H), 7.26-7.19 (m, 2H), 7.04-7.02 (m, 3H), 5.58 (s, 1H), 5.05 (s, 1H), 2.36 (s, 3H) 1.30 (s, 18H).**^{13}C NMR (100 MHz, $CDCl_3$)** δ 160.9, 152.6, 151.2, 138.7, 137.2, 135.8, 132.3, 131.7, 130.6, 129.2, 127.1, 127.0, 126.2, 123.8, 123.4, 121.6, 121.2, 120.8, 112.4, 45.7, 34.4, 30.2, 20.9. **HRMS (ESI) m/z:** $[M+H]^+$ calcd for $C_{31}H_{34}NO_3$ 468.2533; found 468.2542. **FTIR (cm^{-1})** 3635, 3333, 2959, 2913, 2869, 1688, 1547, 1433, 1236, 1210, 1117, 888.

(*S*)-12-(3,5-Di-*tert*-butyl-4-hydroxyphenyl)-4-methoxy-7,12-dihydro-6*H*-benzo[6,7]oxepino[3, 4-b]indol-6-one (3aa)

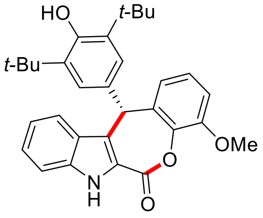

3aa

Following the general procedure, treatment of 1*H*-indole-2-carbaldehyde **1a** (36.3 mg, 0.25mmol) and 2,6-di-*tert*-butyl-4-(2-hydroxy-3 methoxybenzylidene)cyclohexa-2,5-dien-1-one **2g** (119.2 mg, 0.35 mmol) with triazolium salt **4** (23.2 mg, 0.05 mmol), Cs_2CO_3 (49 mg, 0.15 mmol), oxidant **8** (204 mg, 0.5 mmol), $Bi(OTf)_3$ (33.0 mg, 0.05mmol) in toluene (4.0 mL) and stirring the reaction mixture at 18 °C for 36 h followed by flash column chromatography (Pet. ether -EtOAc: 98:2 to 85:15) to afford (*S*)-12-(3,5-di-*tert*-butyl-4-hydroxyphenyl)-4-methoxy-7,12-dihydro-6*H*-benzo[6,7]oxepino[3,4-b]indol-6-one (**3aa**) as a

white solid (83 mg, 69 % yield with 92:8 er). R_f (Pet. ether /EtOAc = 85/15): 0.32; er = 92:8, $[\alpha]_D^{25}$ = +108.74 (c 1.0, $CHCl_3$). **HPLC** (Chiralpak IA, *n*-hexane/IPA = 98:2, flow rate = 1.0 mL/min, λ = 254 nm) t_R = 37.1 min (major), 41.7 min (minor).

^{1}H NMR (400 MHz, $CDCl_3$) δ 8.99 (s, 1H), 7.87 (d, *J* = 7.77 Hz, 1H), 7.43-7.40 (m, 2H), 7.23-7.14 (m, 2H), 7.07-7.05 (m, 1H), 6.99 (s, 2H), 6.92-6.90 (m, 1H), 5.60 (s, 1H), 5.04 (s, 1H), 3.91 (s, 3H), 1.29 (s, 18H). **^{13}C NMR (100 MHz, $CDCl_3$)** δ 160.7, 152.6, 152.3, 140.7, 137.3, 137.2, 135.8, 131.5, 128.9, 127.0, 126.4, 126.2, 123.8, 122.0, 121.8, 121.1, 120.7, 112.5, 111.4, 56.4, 45.9, 34.4, 30.2. **HRMS (ESI) m/z:** $[M+ Na]^+$ calcd for $C_{31}H_{33}NO_4Na$ 506.2302; found 506.2305. **FTIR (cm^{-1})** 3634, 33246, 2960, 2869, 2704, 2331, 1693, 1476, 1434, 1232, 1170, 1081, 886.

2-((3,5-Di-*tert*-butyl-4-oxocyclohexa-2,5-dien-1-ylidene)methyl)phenyl-1*H*-indole-2-carboxylate (3a')

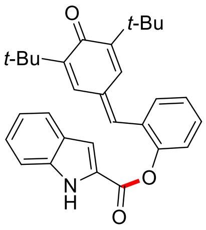

3a'

Following the general procedure, treatment of 1*H*-indole-2-carbaldehyde **1a** (36.3 mg, 0.25 mmol) and 2,6-di-*tert*-butyl-4-(2-hydroxybenzylidene) cyclohexa-2,5-dien-1-one **2a** (108.6 mg, 0.35 mmol) with triazolium salt **4** (23.2 mg, 0.05 mmol), Cs_2CO_3 (49.0 mg, 0.15 mmol), oxidant **8** (204.0 mg, 0.5 mmol), in toluene (4.0 mL) and stirring the reaction mixture at 18 °C for 36 h followed by flash column chromatography (Pet. ether -EtOAc: 98:2 to 90:10) to afford 2-((3,5-Di-*tert*-butyl-4-oxocyclohexa-2,5-dien-1-ylidene)methyl)phenyl-1*H*-indole-2-carboxylate **3a'** as a orange solid (98.0 mg, 87 % yield). R_f (Pet. ether /EtOAc = 90/10): 0.44

^{1}H NMR (400 MHz, Acetone-d6) δ 11.21 (s, 1H), 7.74 (d, *J* = 8.09 Hz, 1H), 7.66-7.64 (m, 1H), 7.60-7.57 (m, 2H), 7.51-7.43 (m, 5H), 7.37-7.33 (m, 1H), 7.19-7.14 (m, 2H), 1.28 (s, 9H), 1.23 (s, 9H). **^{13}C NMR (100 MHz, Acetone-d6)** δ 186.9, 160.2, 150.2, 149.7, 148.2, 139.0, 138.0, 135.7, 133.9, 132.6, 131.3, 130.0, 128.8, 128.1, 127.0, 126.4, 124.1, 123.2, 121.5, 113.3, 110.8, 35.9, 35.5, 29.6. **HRMS (ESI) m/z:** $[M+H]^+$ calcd for $C_{30}H_{32}NO_3$ 454.2377; found 454.2381 **FTIR (cm^{-1})** 3608, 3345, 2956, 2866, 1711, 1614, 1526, 1452, 1361, 1219, 1175, 1144, 1101, 1023, 949.

3-((3,5-Di-*tert*-butyl-4-hydroxyphenyl)(2-hydroxyphenyl)methyl)-1*H*-indole-2-carbaldehyde (3a")

Following the general procedure, treatment of 1*H*-indole-2-carbaldehyde **1a** (36.3 mg, 0.25 mmol) and 2,6-di-*tert*-butyl-4-(2-hydroxybenzylidene) cyclohexa-2,5-dien-1-one **2a** (108.6 mg, 0.35

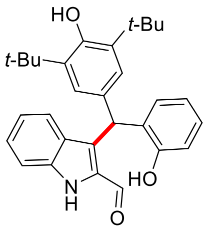

3a"

mmol) with Cs_2CO_3 (49.0 mg, 0.15 mmol), oxidant **8** (204.0 mg, 0.5 mmol), $Bi(OTf)_3$ (33.0 mg, 0.05mmol) in toluene (4.0 mL) and stirring the reaction mixture at 18 °C for 36 h followed by flash column chromatography (Pet. ether-EtOAc: 98:2 to 90:10) to afford 3-((3,5-Di-*tert*-butyl-4-hydroxyphenyl)(2-hydroxyphenyl)methyl)-1*H*-indole-2-carbaldehyde (**3a"**) as a white solid (101.0 mg, 89 % yield with 50:50 er).

Rf (Pet. ether /EtOAc = 85/15): 0.36; er = 50:50, **HPLC** (Chiralpak IA, *n*-hexane/IPA = 85:15, flow rate = 1.0 mL/min, λ = 254 nm) t_R = 8.8 min, 12.4 min.

^{1}H NMR (400 MHz, Acetone-d6) δ 10.62 (s, 1H), 9.56 (s, 1H), 8.43 (s, 1H), 7.50-7.48 (m, 1H), 7.40-7.38 (m, 1H), 7.28-7.24 (m, 1H), 7.15 (s, 2H), 7.08-7.06 (m, 2H), 6.96-6.89 (m, 2H), 6.80-6.77 (m, 1H), 6.59 (s, 1H), 5.96 (s, 1H), 1.33 (s, 18H). **^{13}C NMR (100 MHz, Acetone-d6)** δ 183.1, 155.6, 153.7, 153.3, 138.7, 138.2, 134.5, 134.0, 131.5, 131.0, 128.5, 128.5, 127.1, 126.5, 123.7, 120.6, 120.3, 116.2, 113.5, 41.6, 35.2, 30.7. **HRMS (ESI) m/z:** $[M+H]^+$ calcd for $C_{30}H_{34}NO_3$ 456.2533; found 456.2539. **FTIR (cm^{-1})** 3630, 3419, 3198, 2954, 1619, 1506, 1426, 1318, 1245, 1196, 1147, 877.

Phenyl-3-((4-bromophenyl)(3,5-di-tert-butyl-4-hydroxyphenyl)methyl)-1*H*-indole-2-carboxylate (10)

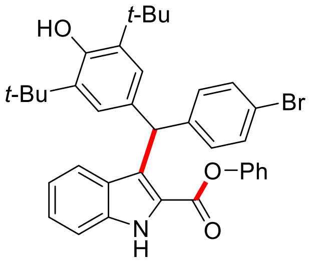

10

Following the general procedure, treatment of 1*H*-indole-2-carbaldehyde **1a** (36.3 mg, 0.25 mmol) and 4-(4-bromobenzylidene)-2,6-di-tert-butylcyclohexa-2,5-dien-1-one **9a** (130.2 mg, 0.35 mmol) with triazolium salt **4** (23.2 mg, 0.05 mmol), Cs_2CO_3 (49.0 mg, 0.15 mmol), PhOH (23.5 mg, 0.25 mmol), oxidant **8** (204.0 mg, 0.5 mmol), $Bi(OTf)_3$ (33.0 mg, 0.05mmol) in toluene (4.0 mL) and stirring the reaction mixture at 18 °C for 36 h followed by flash column chromatography (Pet. ether -EtOAc: 98:2 to 90:10) to afford Phenyl-3-((4-bromophenyl)(3,5-di-tert-butyl-4-hydroxyphenyl)methyl)-1*H*-indole-2-carboxylate (**10**) as a white solid (136.0 mg, 89 % yield with 53:47 er). ***R_f*** (Pet. ether /EtOAc = 90/10): 0.43; er = 53:47, **HPLC** (Chiralpak IA, *n*-hexane/IPA = 90:10, flow rate = 1.0 mL/min, λ = 254 nm) t_R = 6.85 min (major), 7.82 min (minor).

^{1}H NMR (400 MHz, $CDCl_3$) δ 9.01 (s, 1H), 7.46-7.35 (m, 5H), 7.31-7.28 (m, 2H), 7.19-7.17 (m, 2H), 7.11-7.04 (m, 5H), 6.97-6.93 (m, 1H), 6.59 (s, 1H), 5.11 (s, 1H), 1.31 (s, 18H). **^{13}C NMR**

(100 MHz, $CDCl_3$) δ 160.6, 152.5, 150.3, 143.6, 136.8, 135.8, 133.0, 131.2, 131.0, 129.7, 128.3, 127.1, 126.3, 126.2, 126.0, 123.8, 122.8, 121.9 ,120.5, 120.0, 112.1, 46.9, 34.5, 30.4. **HRMS (ESI) m/z:** $[M+K]^+$ calcd for $C_{36}H_{36}BrNO_3K$ 648.1510; found 648.1514. **FTIR (cm^{-1})** 3634, 3582, 3348, 2957, 2924, 2871, 1695, 1593, 1487, 1434, 1233, 1193, 1160, 1070, 1010, 909.

8. ^{1}H and ^{13}C NMR Spectra of Tetracyclic ε-Lactones

(*S*)-12-(3,5-Di-*tert*-butyl-4-hydroxyphenyl)-7,12-dihydro-6*H*-benzo[6,7]oxepino[3,4-*b*] indol-6-one (3a)

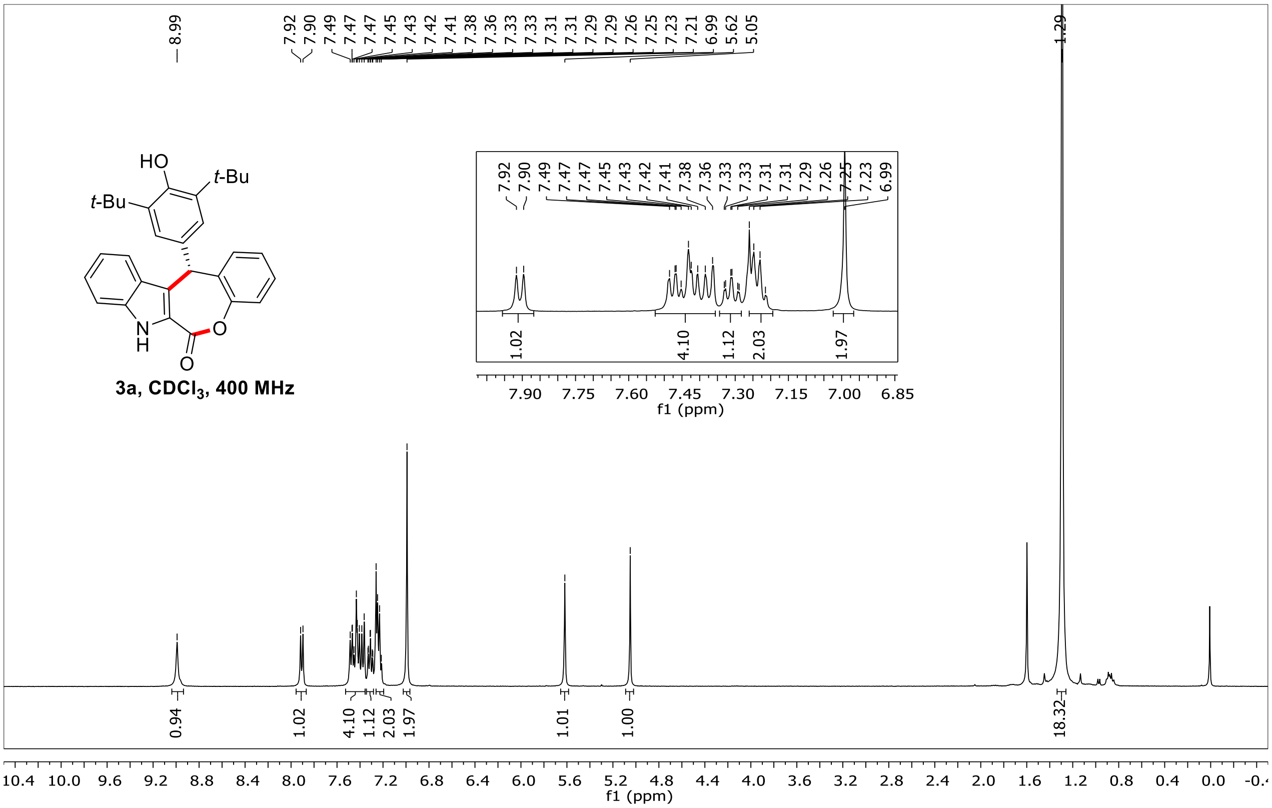

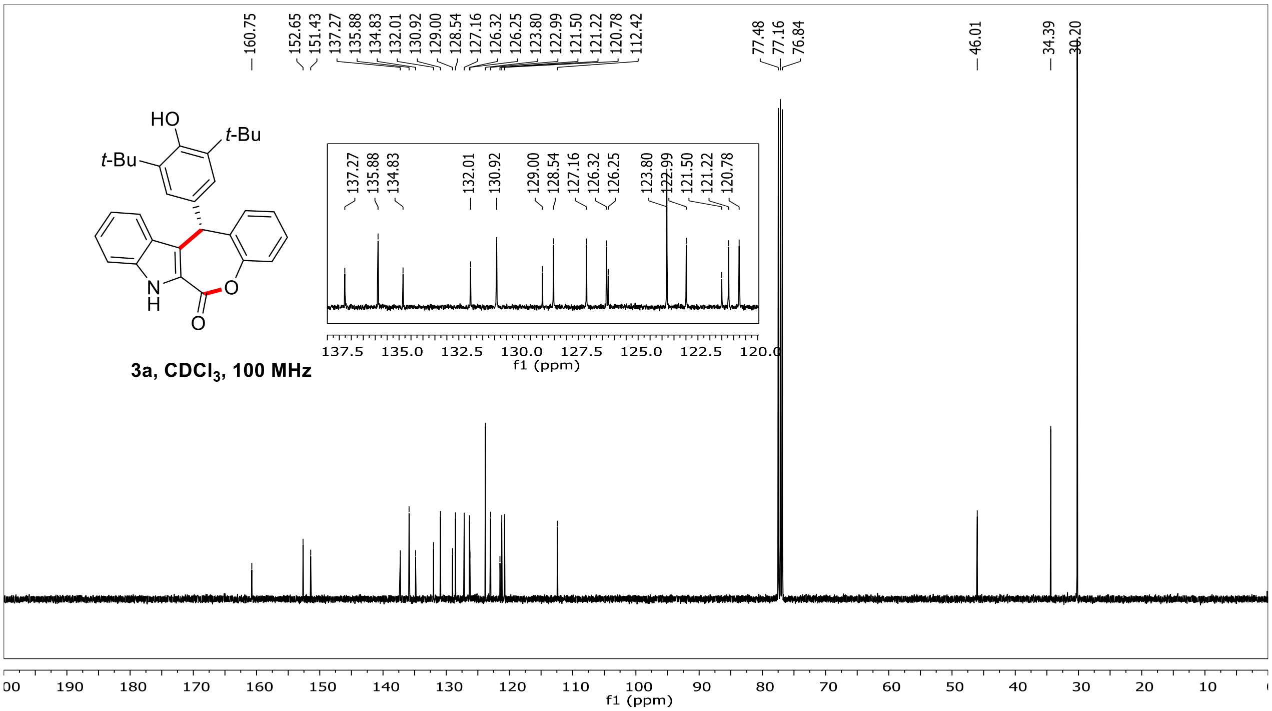

(*S*)-12-(3,5-Di-*tert*-butyl-4-hydroxyphenyl)-11-fluoro-7,12-dihydro-6*H*-benzo[6,7]oxepino[3,4-*b*]indol-6-one (3b)

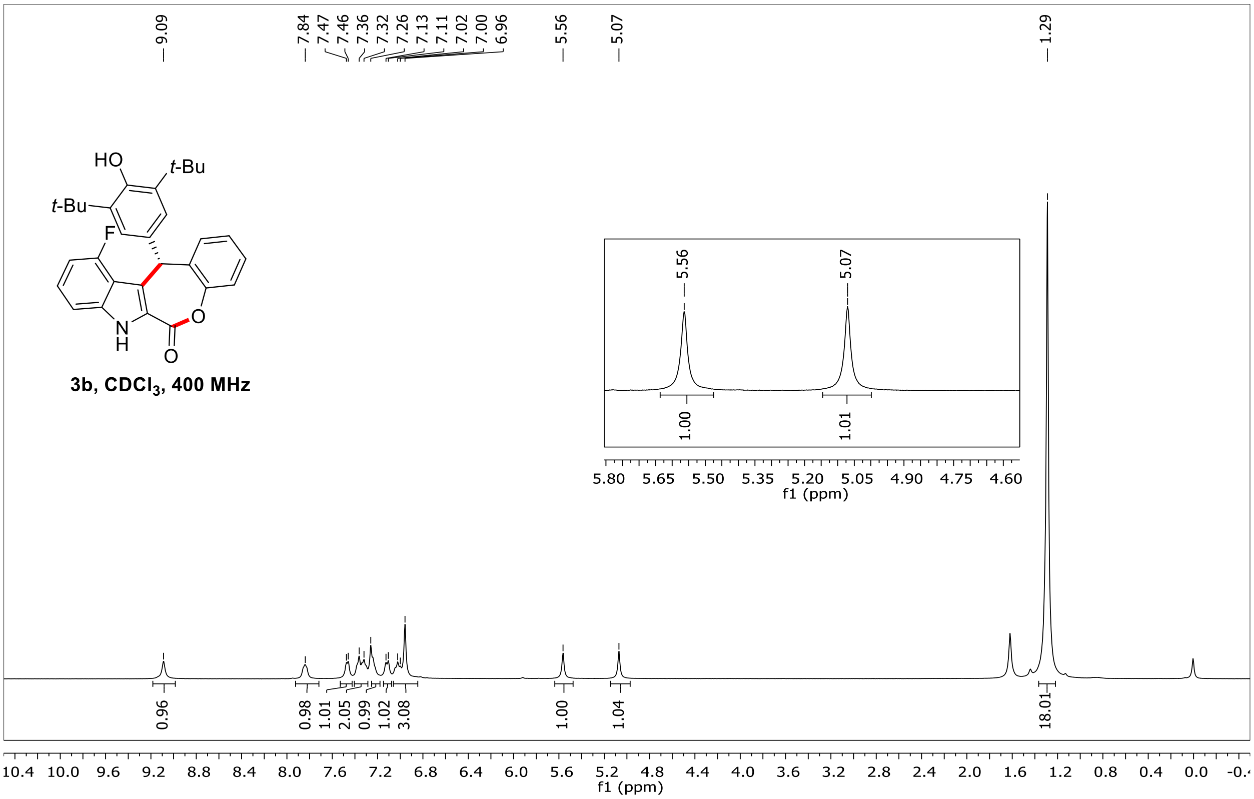

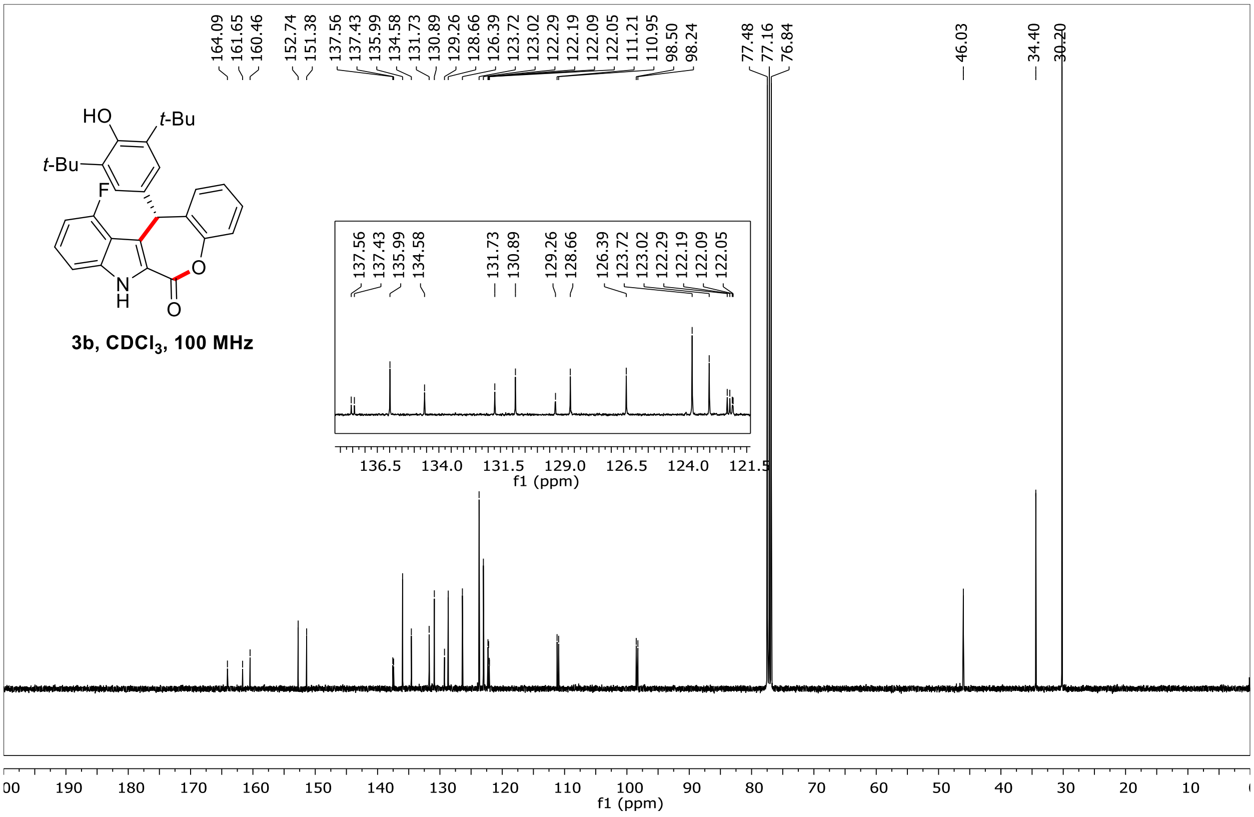

(*S*)-12-(3,5-Di-*tert*-butyl-4-hydroxyphenyl)-11-fluoro-7,12-dihydro-6*H*-benzo[6,7]oxepino[3,4-*b*]indol-6-one (3b)

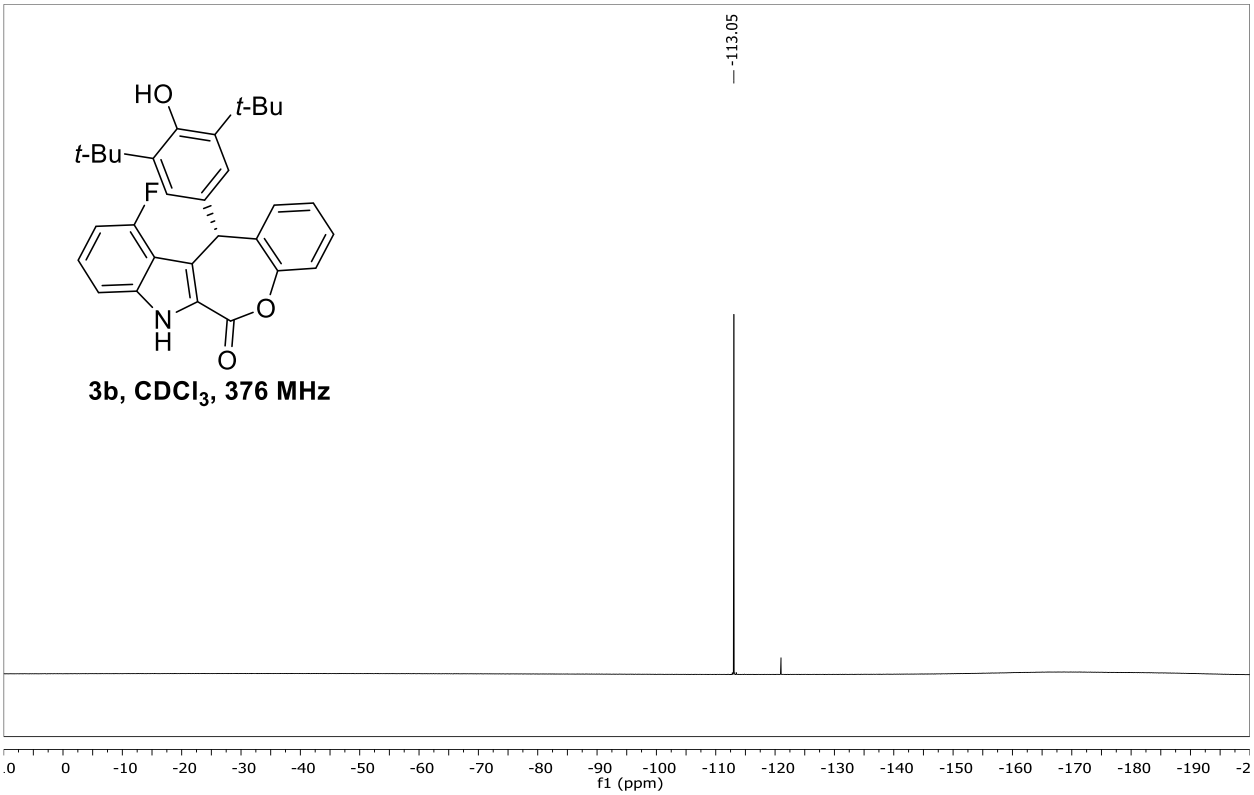

(*S*)-12-(3,5-Di-*tert*-butyl-4-hydroxyphenyl)-10-methoxy-7,12-dihydro-6*H*-benzo[6,7]oxepino[3,4-*b*]indol-6-one (3c)

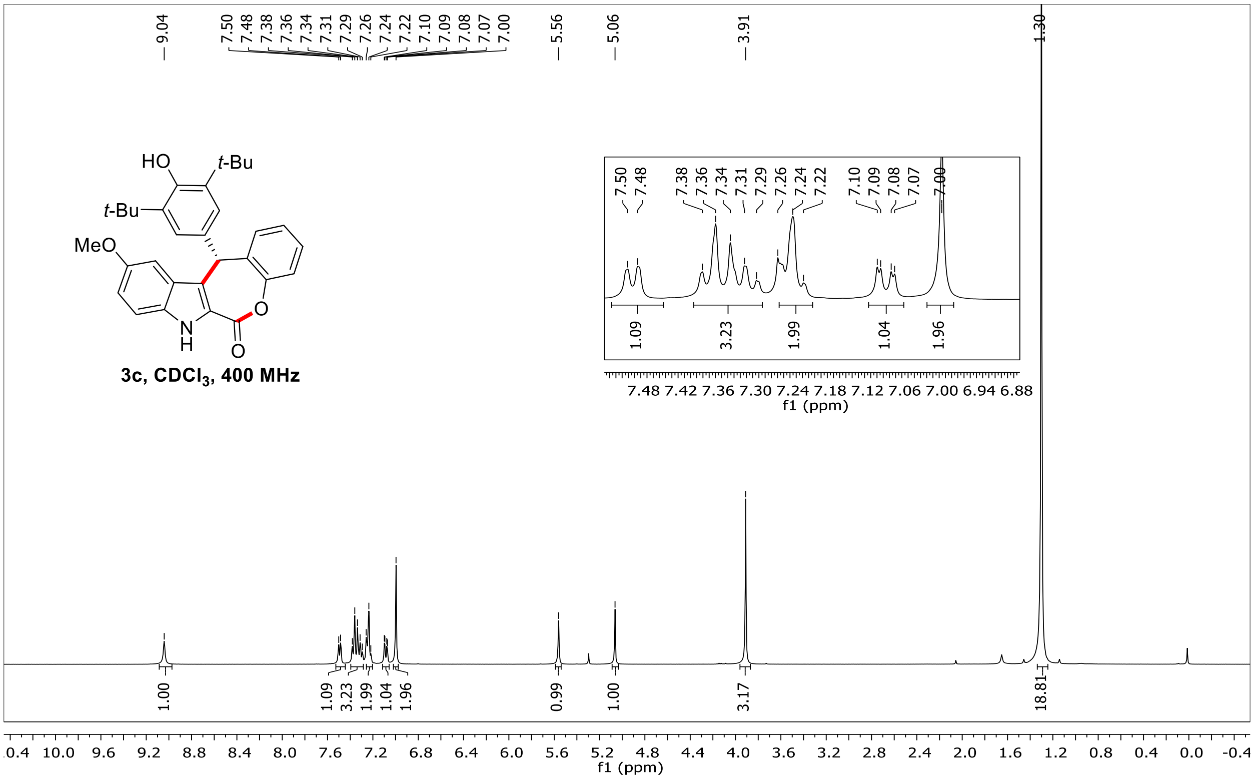

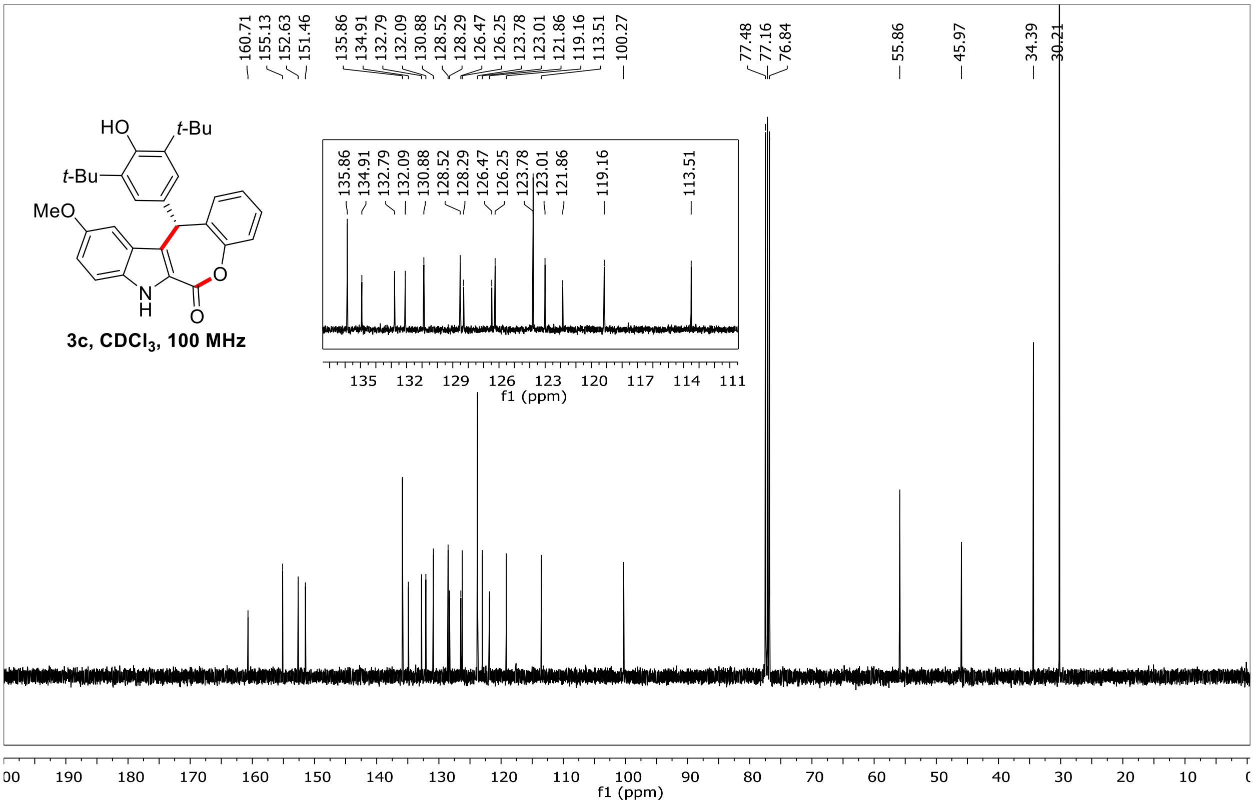

(*S*)-12-(3,5-Di-*tert*-butyl-4-hydroxyphenyl)-10-methyl-7,12-dihydro-6*H* benzo [6,7]oxepino[3,4-b]indol-6-one (3d)

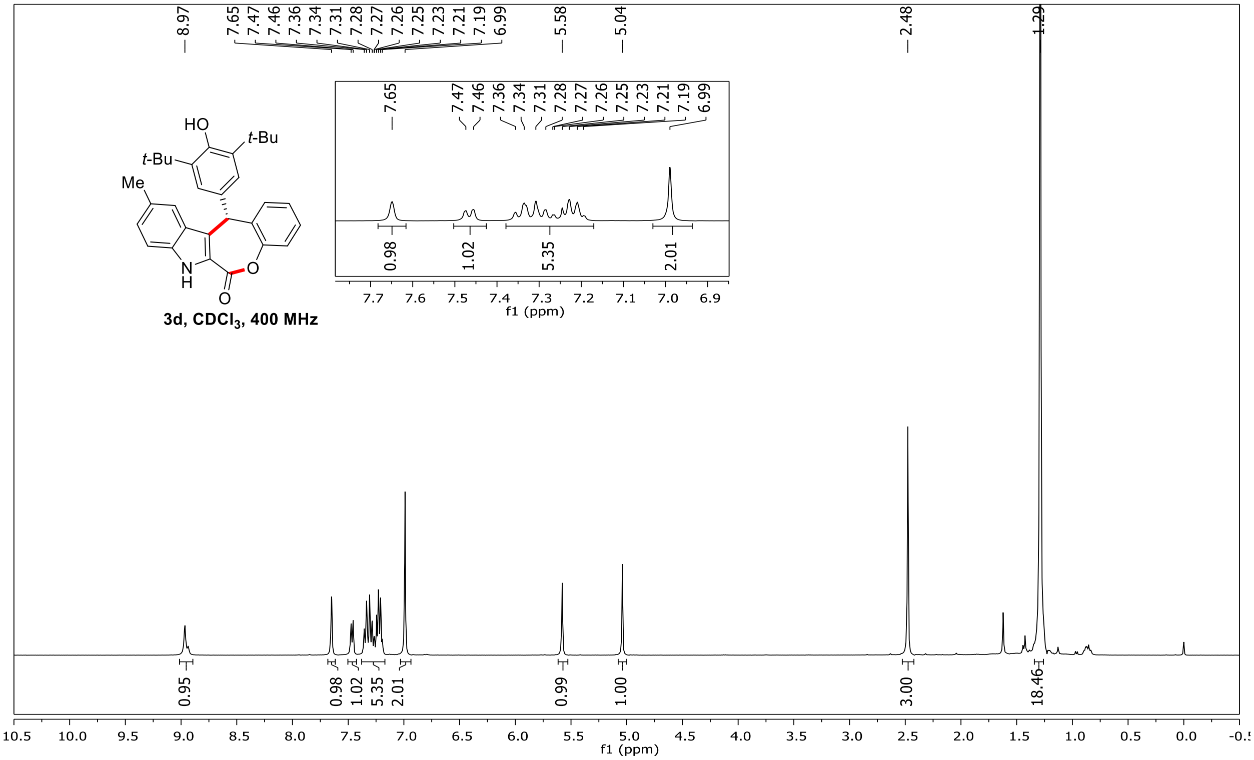

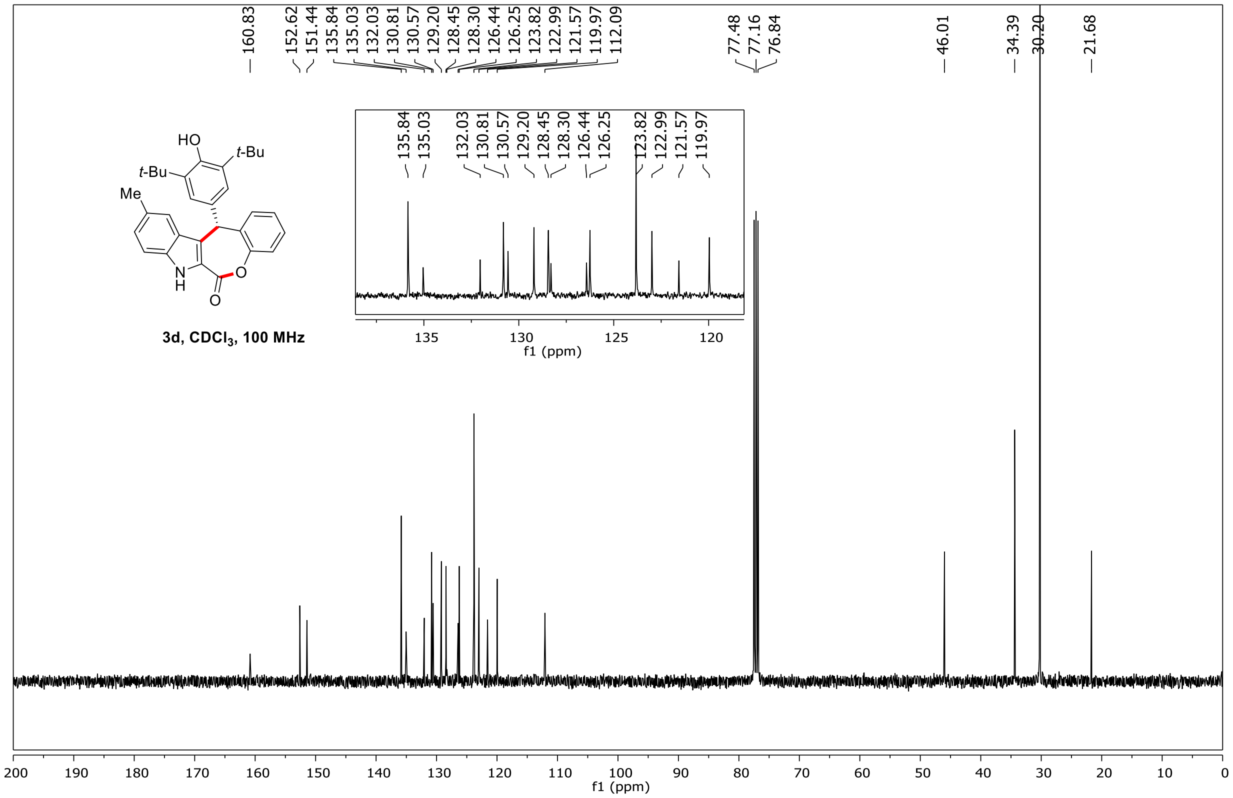

(*S*)-12-(3,5-di-*Tert*-butyl-4-hydroxyphenyl)-10-ethyl-7,12-dihydro-6*H*-benzo[6,7]oxepino[3,4-*b*]indol-6-one (3e)

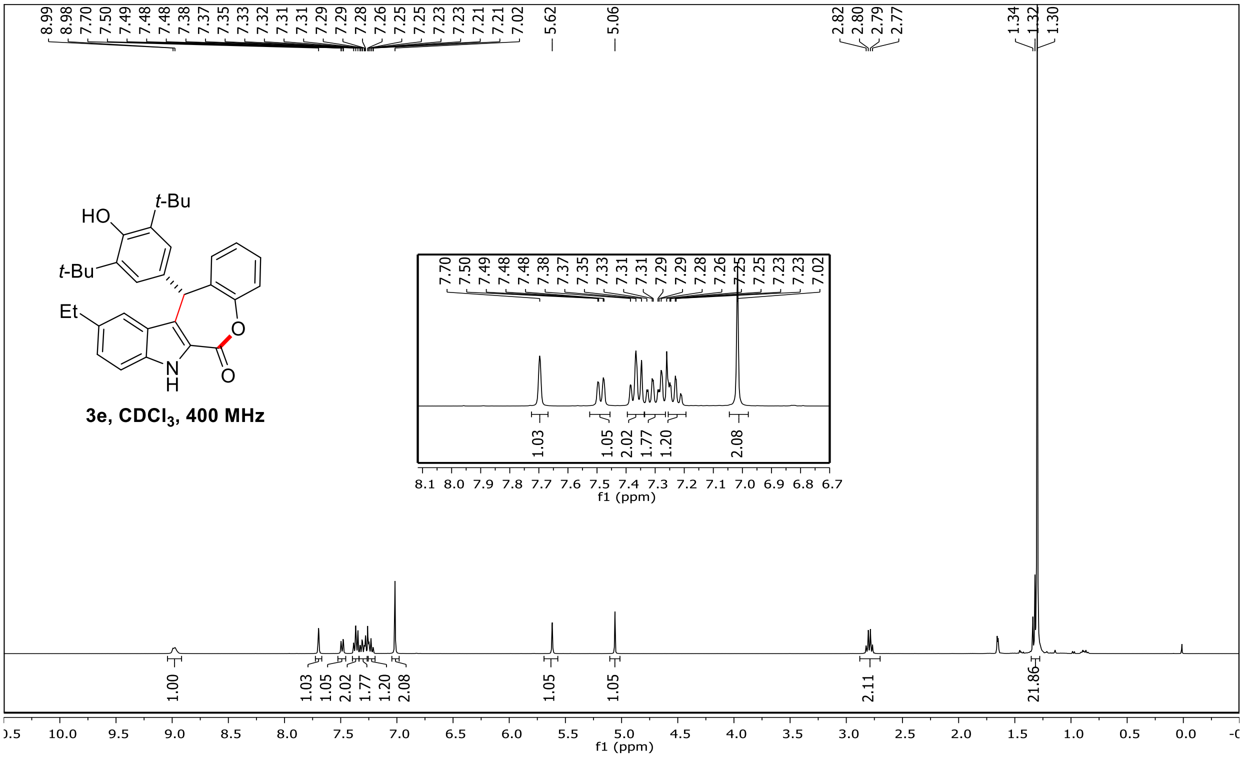

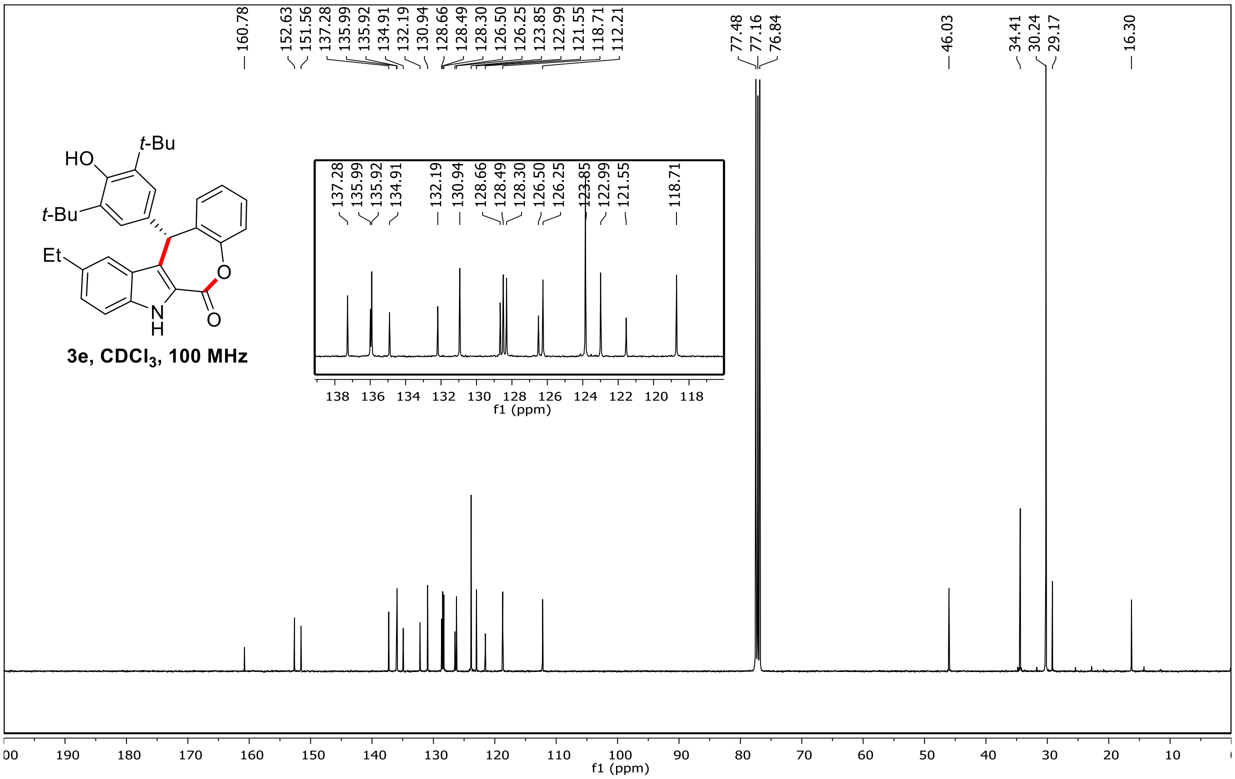

(*S*)-10-(*Tert*-butyl)-12-(3,5-di-*tert*-butyl-4-hydroxyphenyl)-7,12-dihydro-6*H* benzo[6,7]oxepino[3,4-b]indol-6-one (3f)

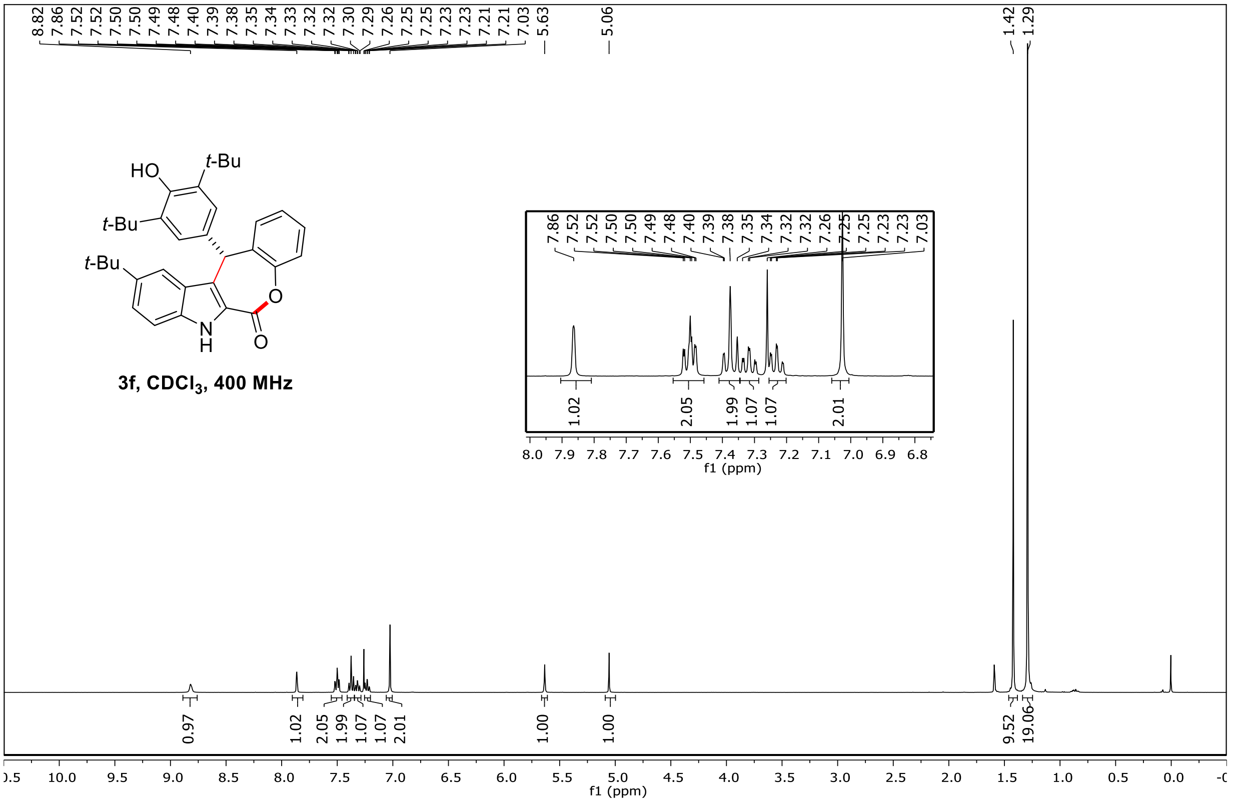

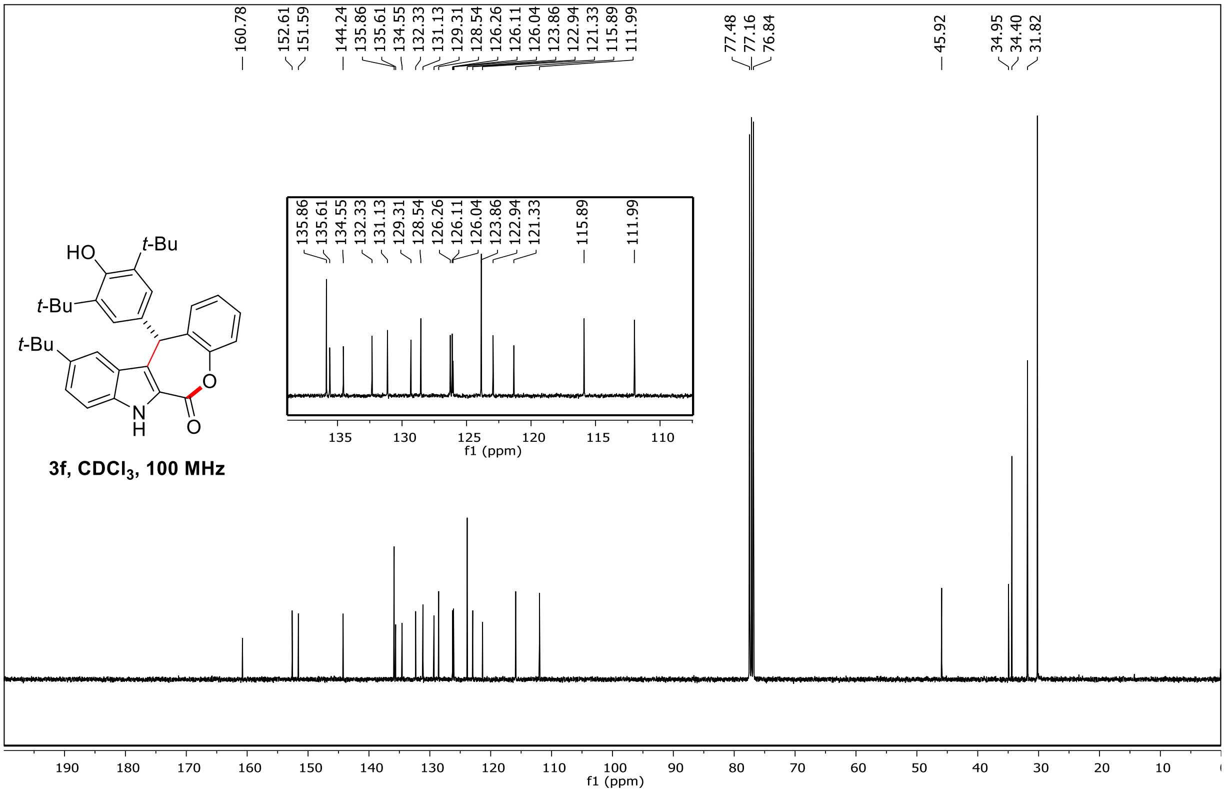

(*S*)-12-(3,5-Di-*tert*-butyl-4-hydroxyphenyl)-10-(trifluoromethoxy)-7,12-dihydro-6*H*-benzo[6,7]oxepino[3,4-b]indol-6-one (3g)

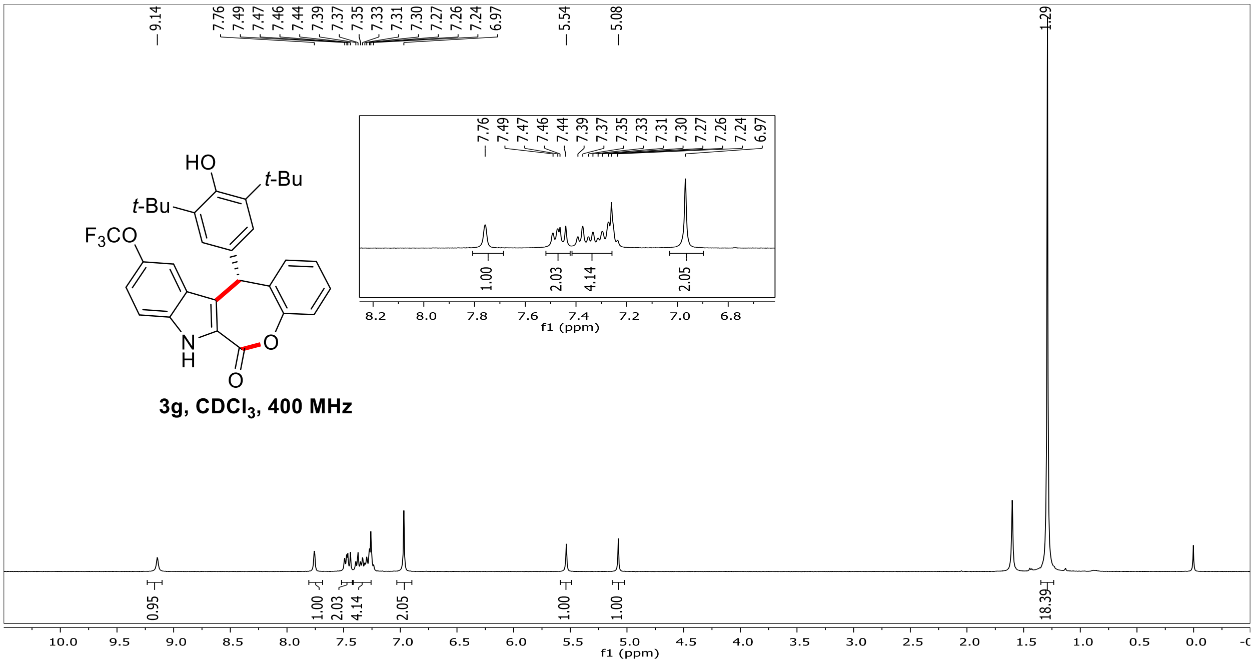

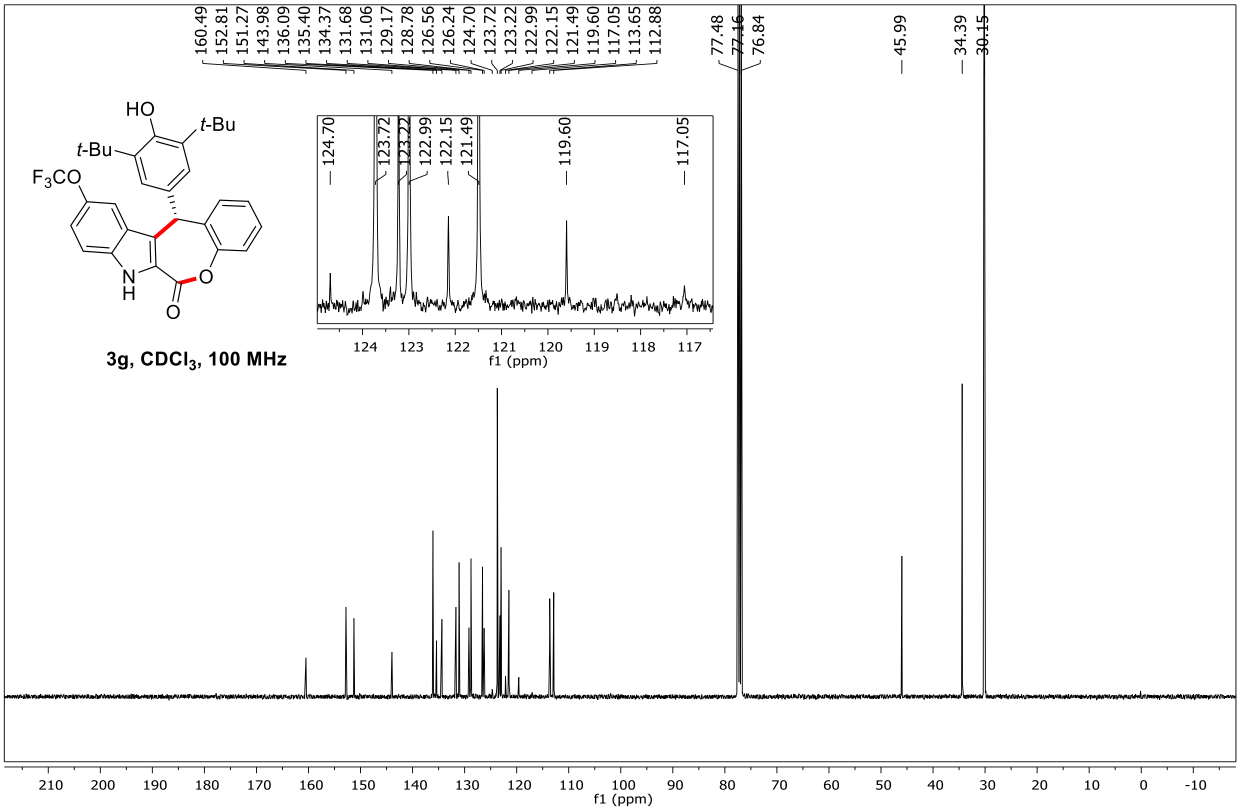

(*S*)-12-(3,5-Di-*tert*-butyl-4-hydroxyphenyl)-10-(trifluoromethoxy)-7,12-dihydro-6*H*-benzo[6,7]oxepino[3,4-b]indol-6-one (3g)

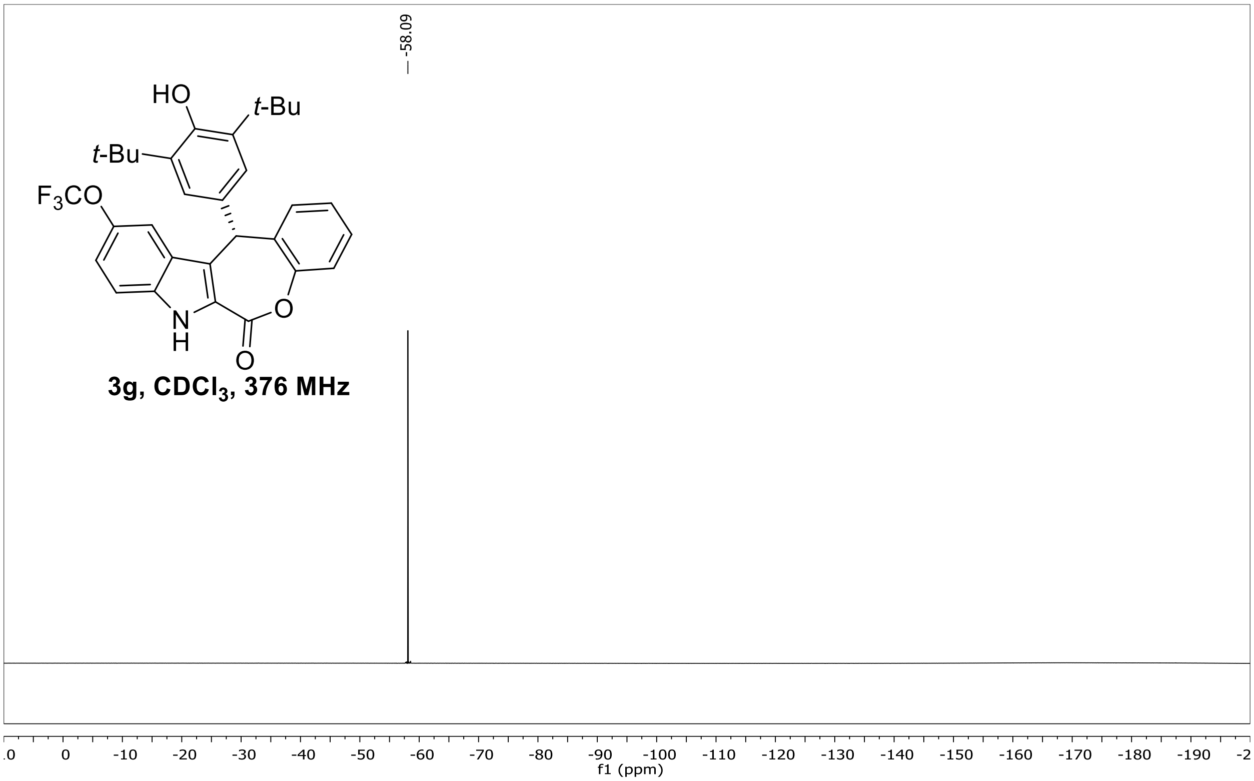

(*S*)-10-Bromo-12-(3,5-di-*tert*-butyl-4-hydroxyphenyl)-7,12-dihydro-6*H*-benzo [6,7]oxepino[3,4-*b*]indol-6-one (3h)

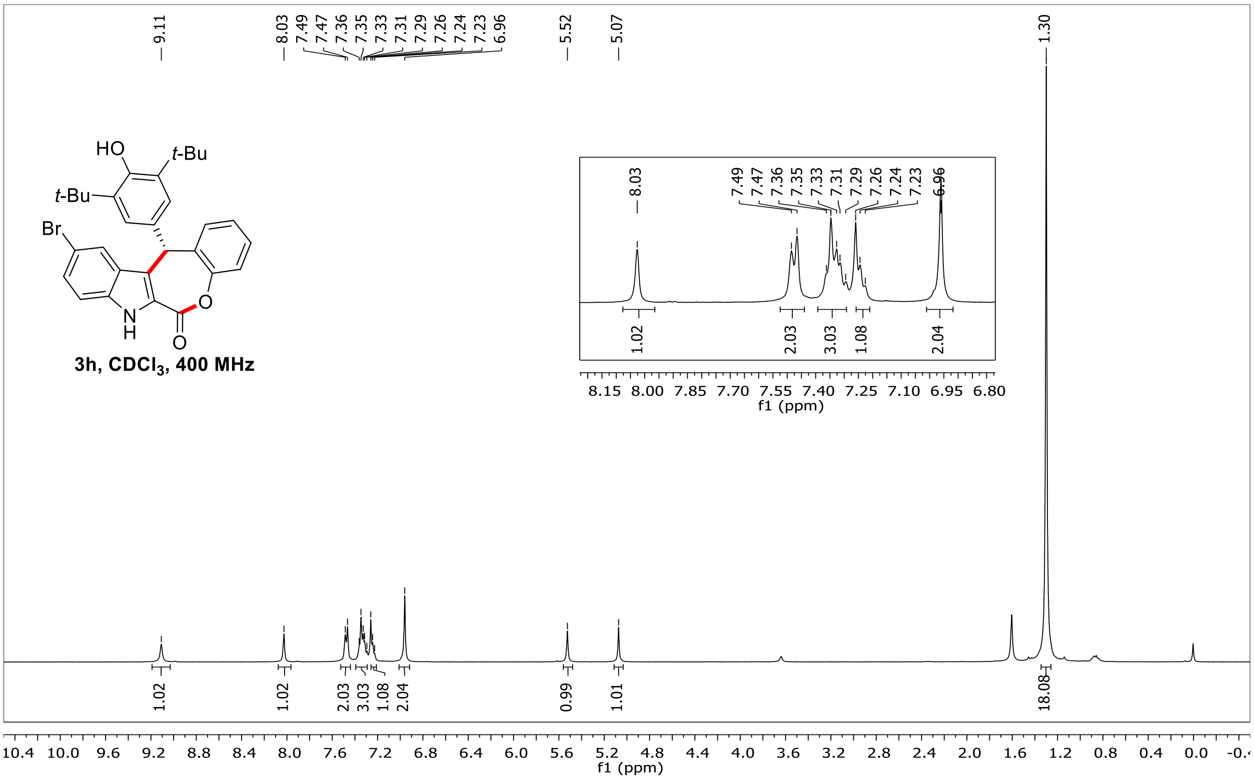

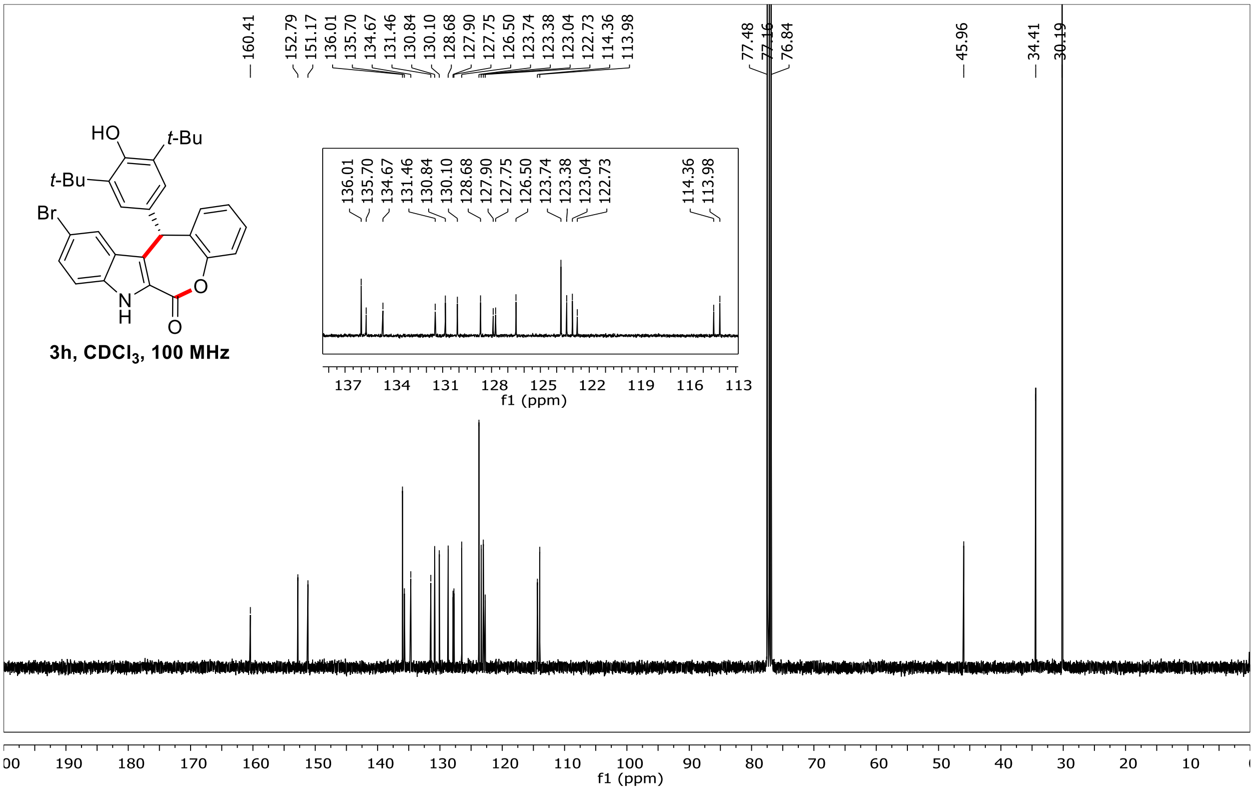

(*S*)-10-Chloro-12-(3,5-di-*tert*-butyl-4-hydroxyphenyl)-7,12-dihydro-6*H*-benzo [6,7]oxepino[3,4-*b*]indol-6-one (3i)

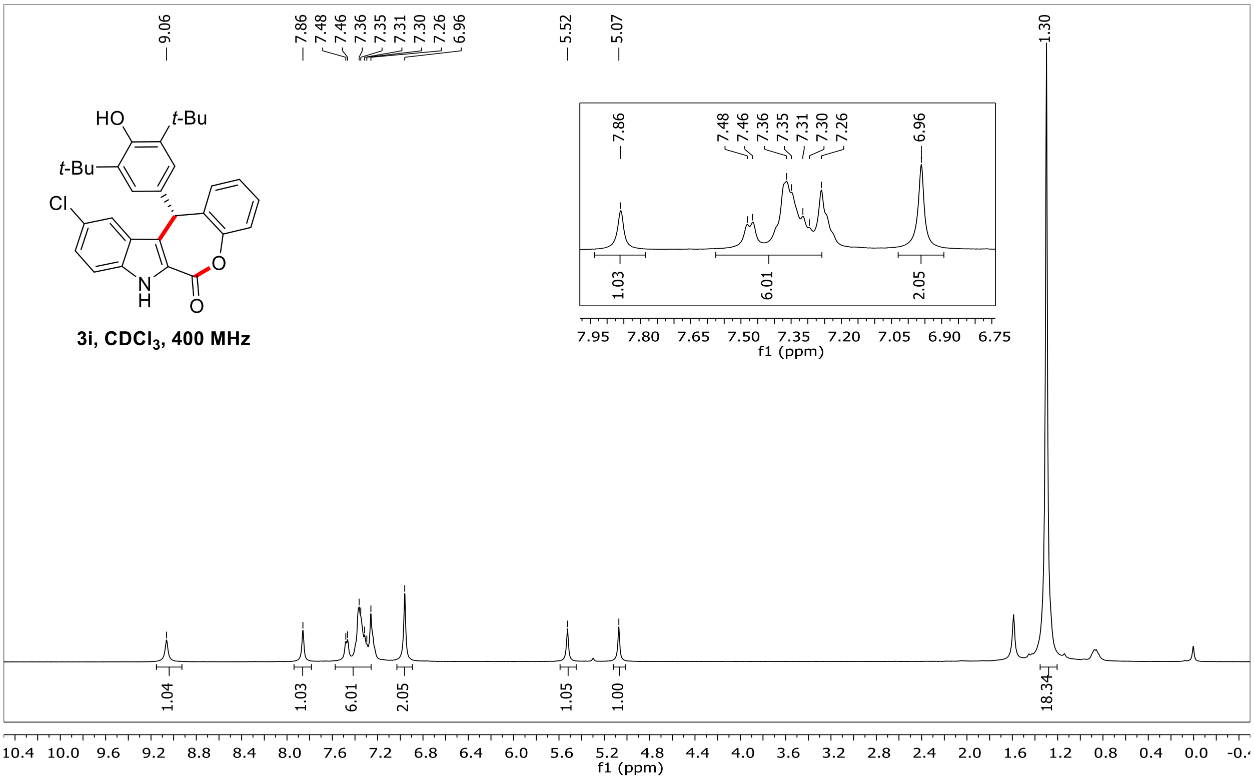

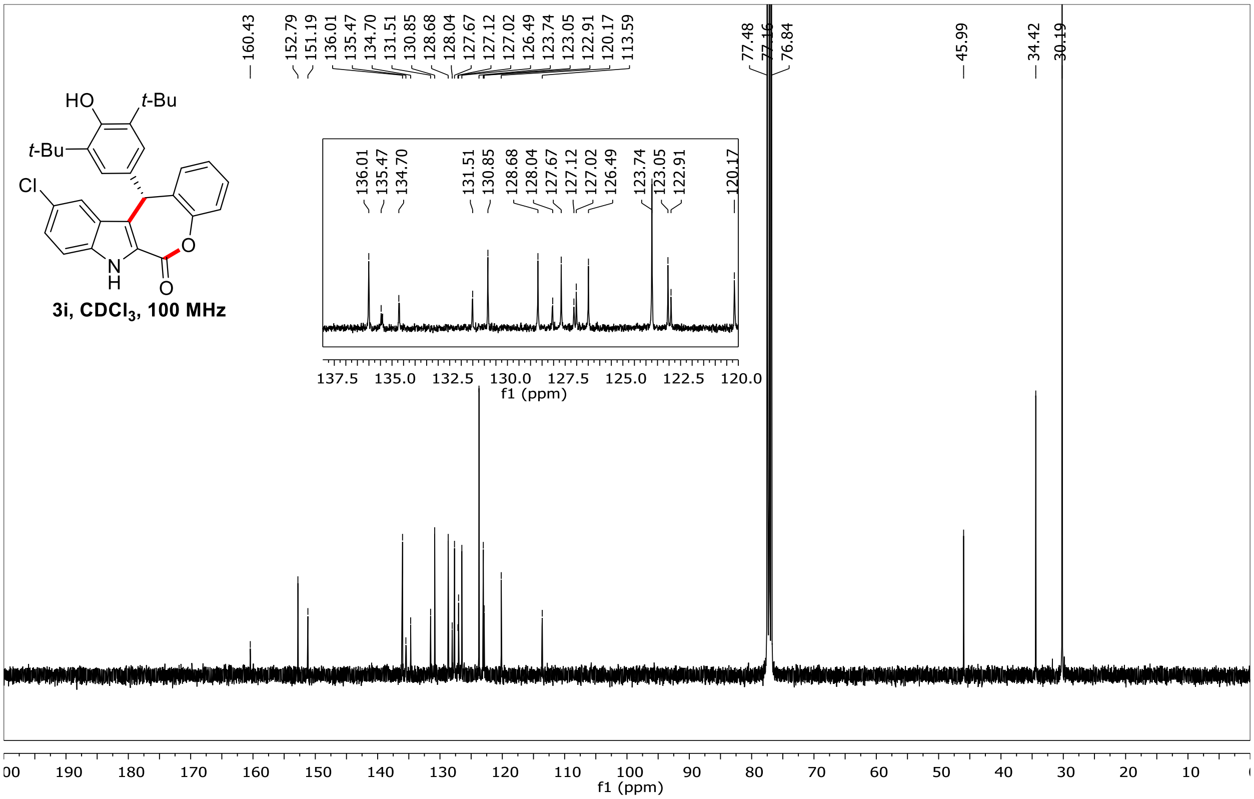

(*S*)-12-(3,5-Di-*tert*-butyl-4-hydroxyphenyl)-10-fluoro-7,12-dihydro-6*H*-benzo[6,7]oxepino[3,4-b]indol-6-one (3j)

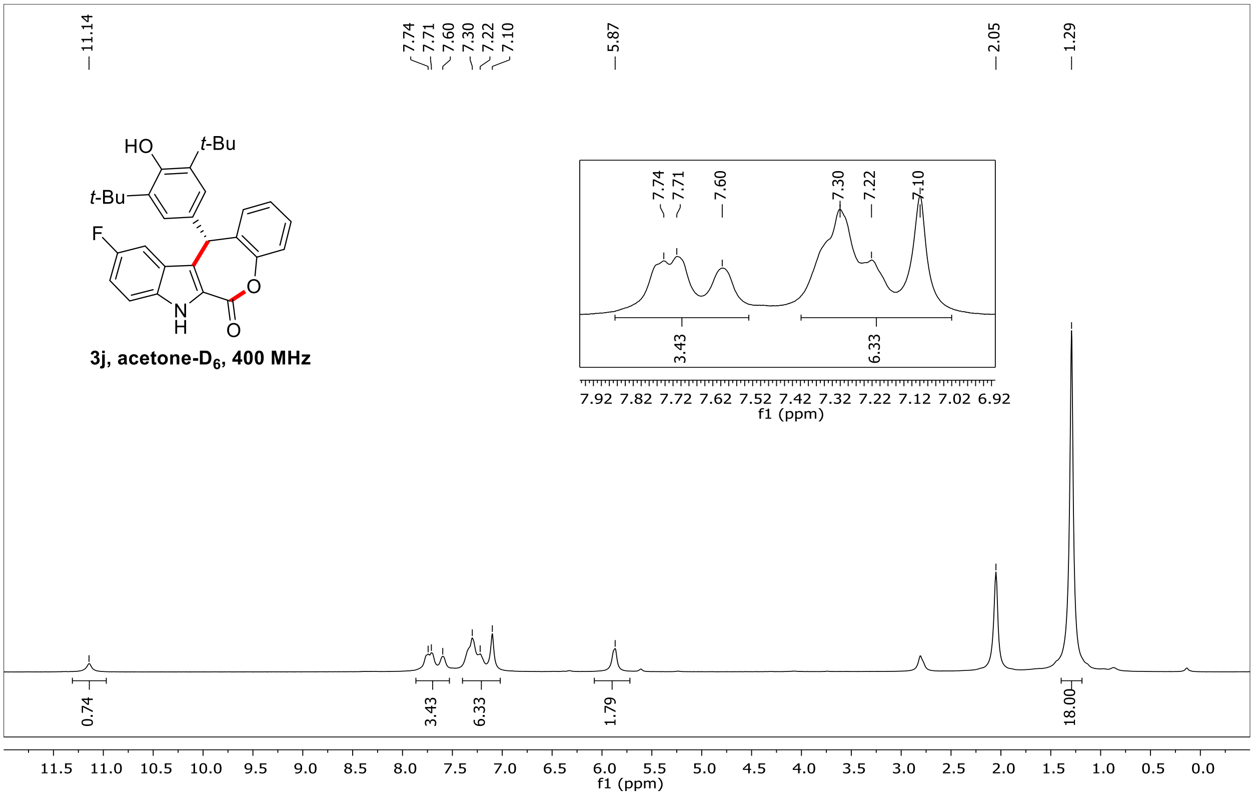

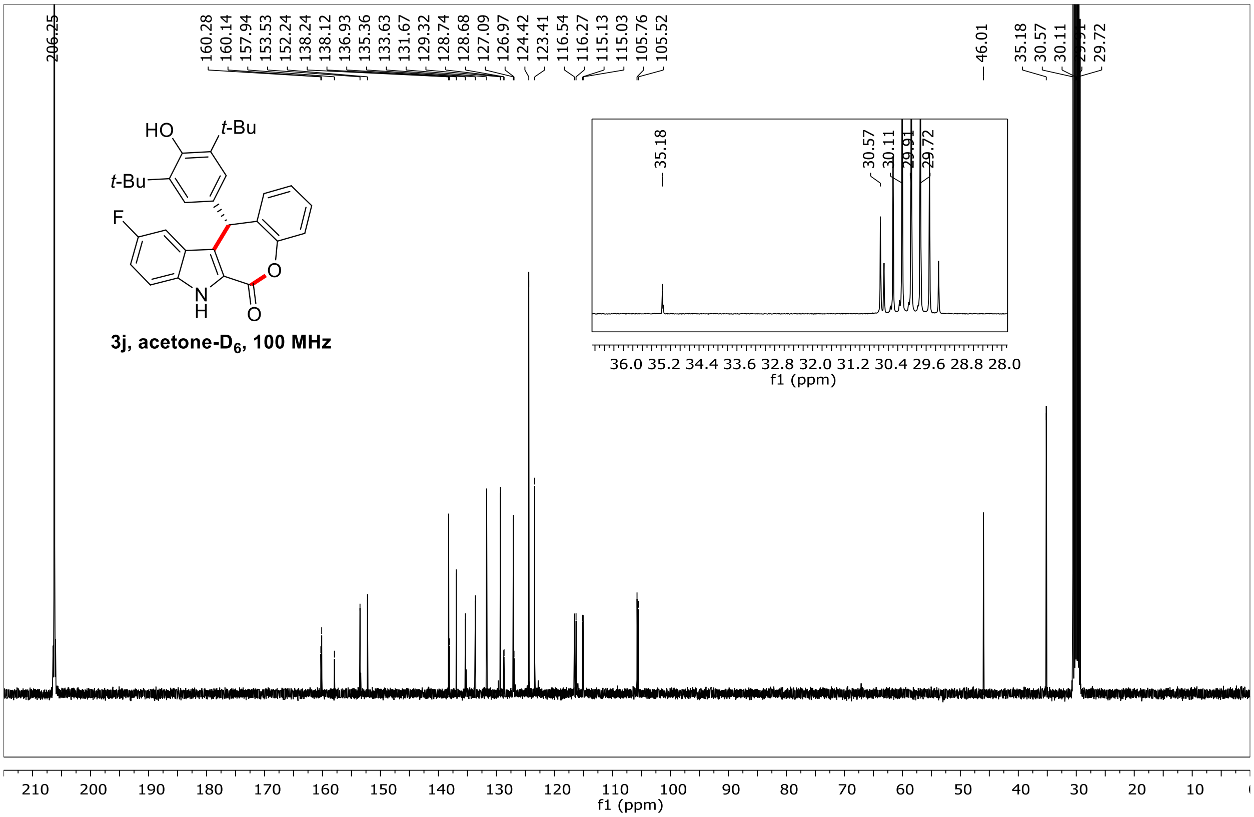

(*S*)-12-(3,5-Di-*tert*-butyl-4-hydroxyphenyl)-10-fluoro-7,12-dihydro-6*H*-benzo[6,7]oxepino[3,4-b]indol-6-one (3j)

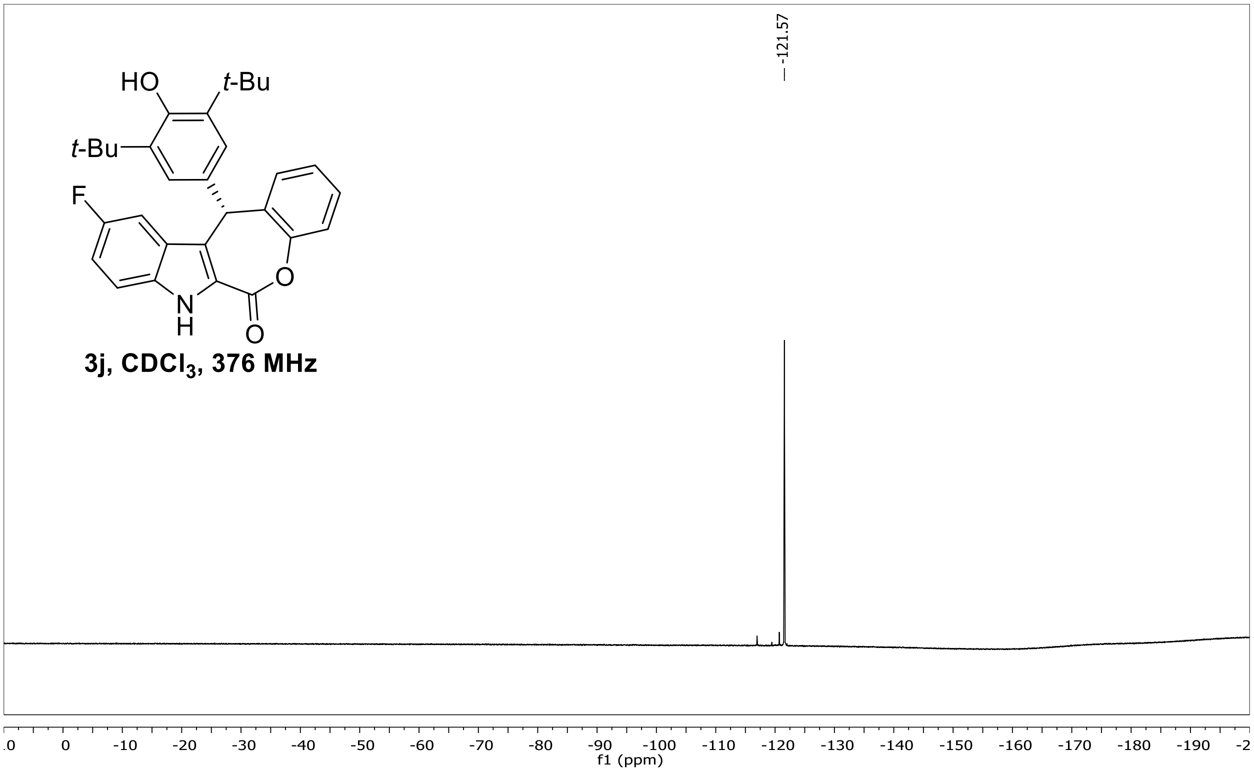

(*S*)-12-(3,5-Di-*tert*-butyl-4-hydroxyphenyl)-9-methoxy-7,12-dihydro-6*H*-benzo[6,7]oxepino[3,4-b]indol-6-one (3k)

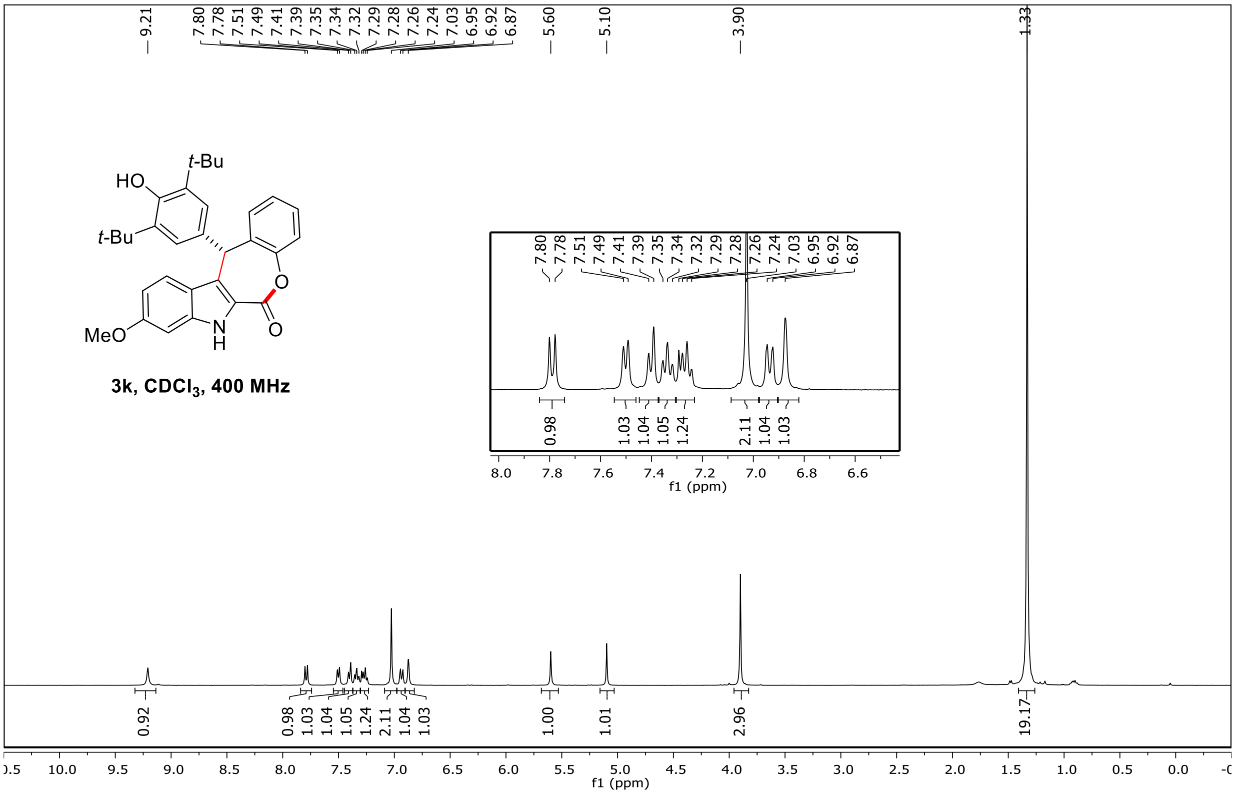

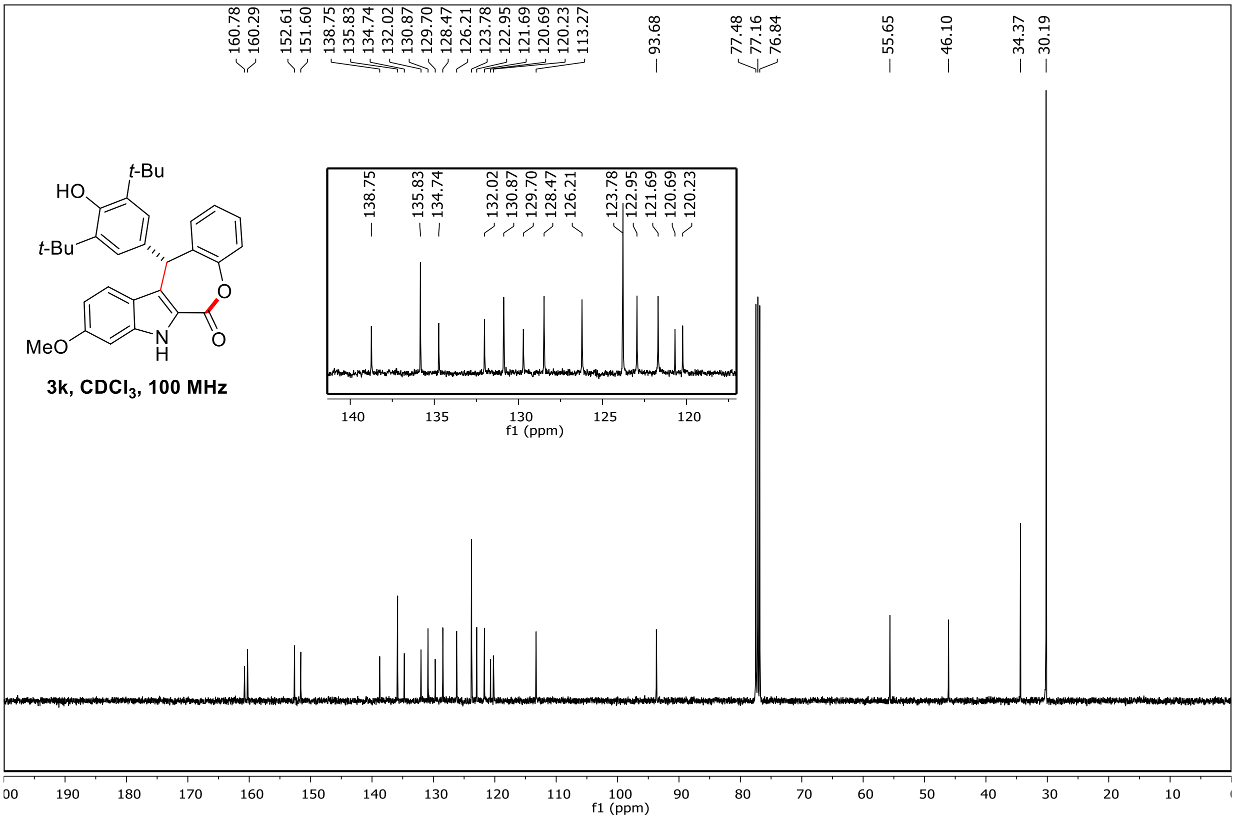

(*S*)-12-(3,5-Di-*tert*-butyl-4-hydroxyphenyl)-9-methyl-7,12-dihydro-6*H*-benzo[6,7]oxepino[3,4-*b*]indol-6-one (3l)

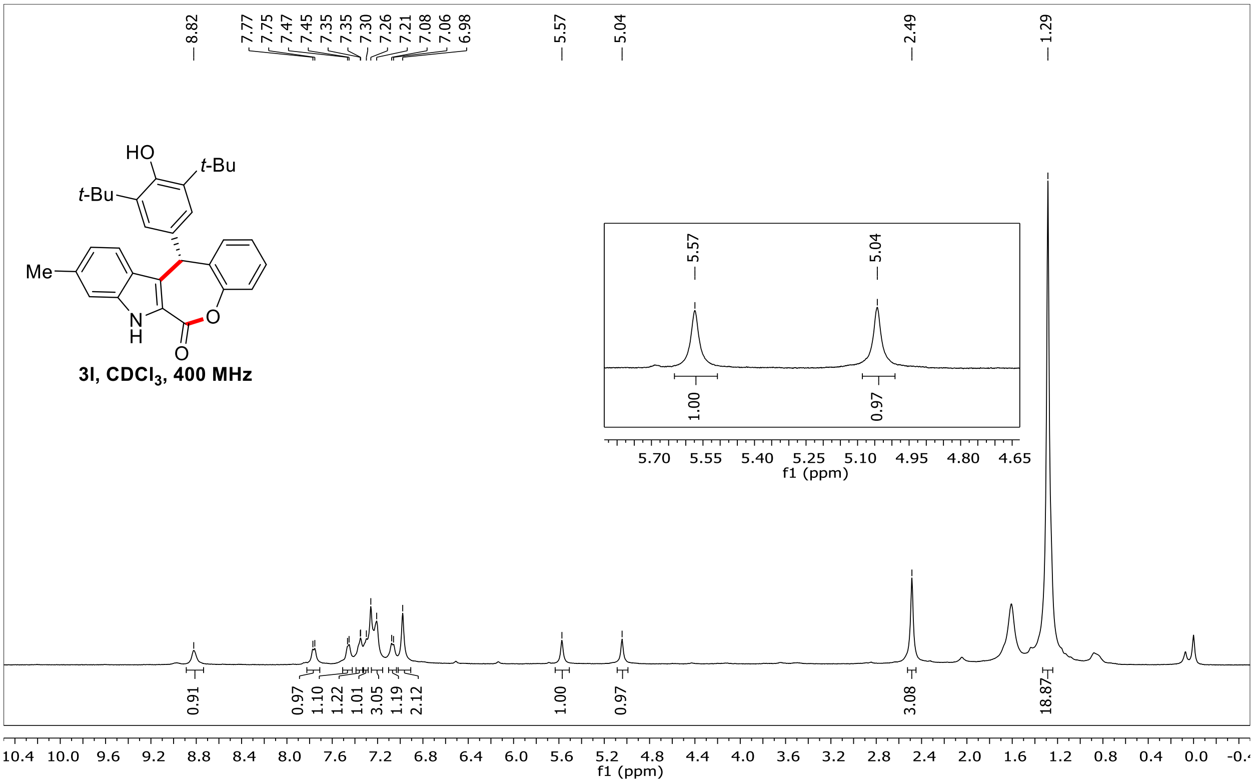

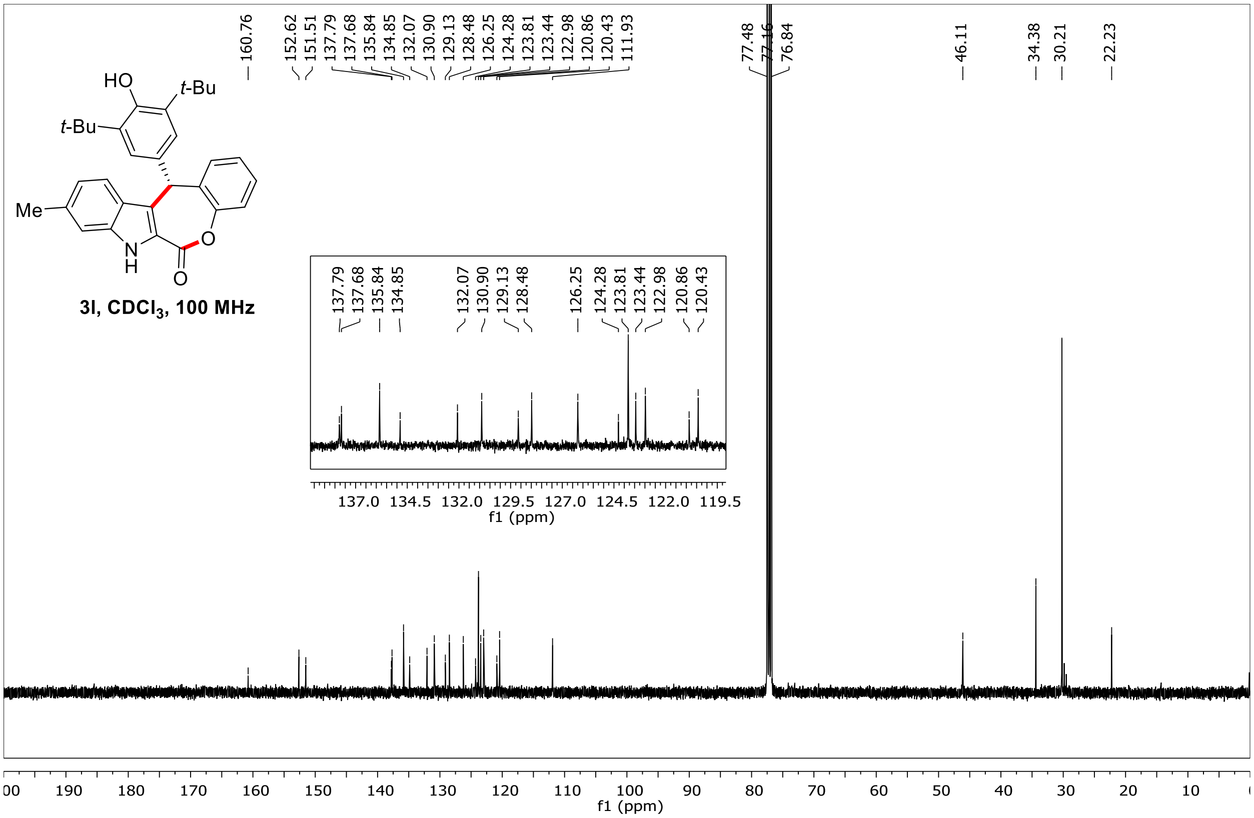

(*S*)-9-Bromo-12-(3,5-di-*tert*-butyl-4-hydroxyphenyl)-7,12-dihydro-6*H*-benzo[6,7]oxepino[3,4-*b*]indol-6-one (3m)

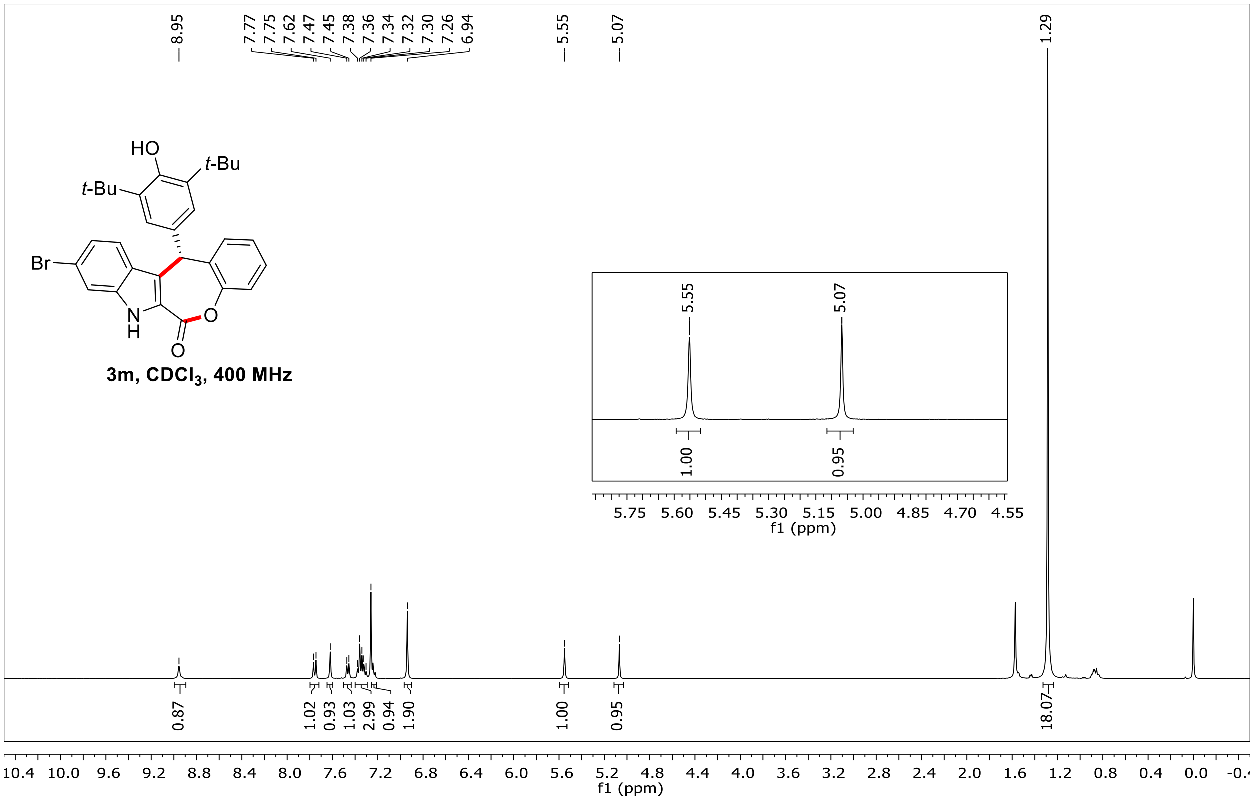

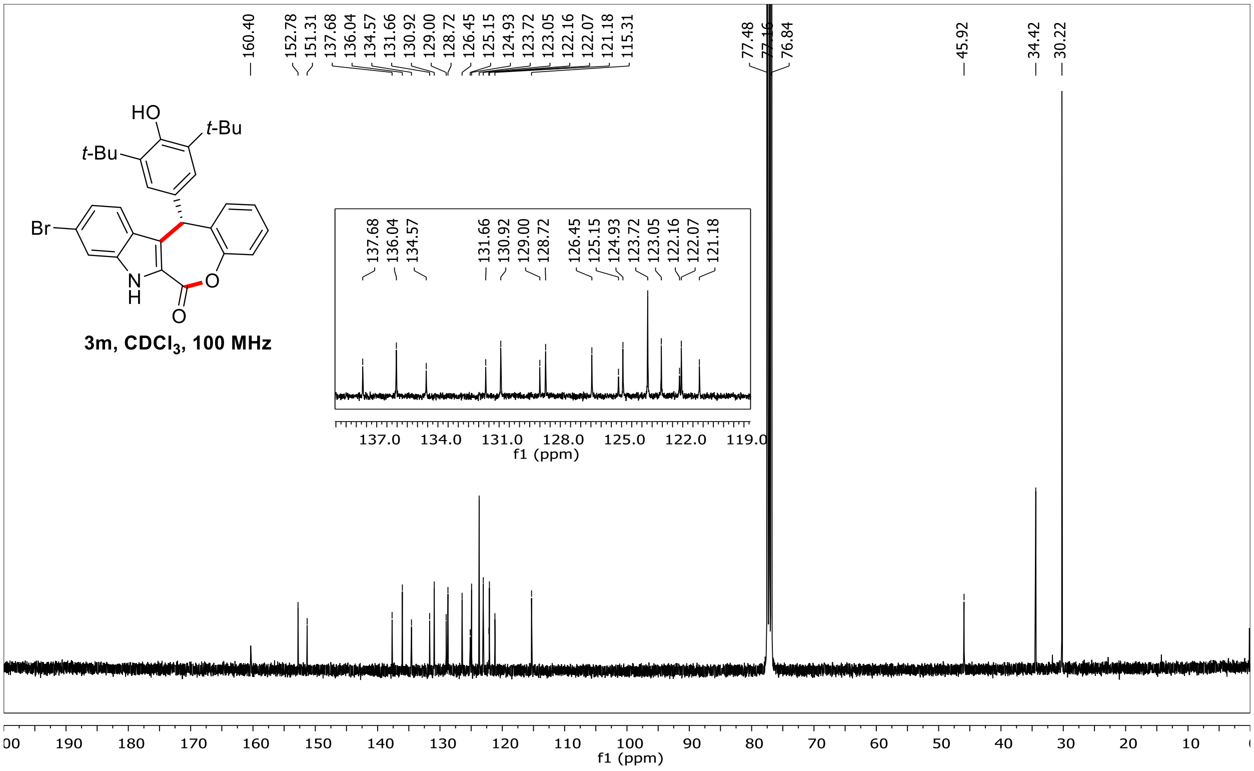

(*S*)-12-(3,5-Di-*tert*-butyl-4-hydroxyphenyl)-9-fluoro-7,12-dihydro-6*H*-benzo[6,7]oxepino[3,4-*b*]indol-6-one (3n)

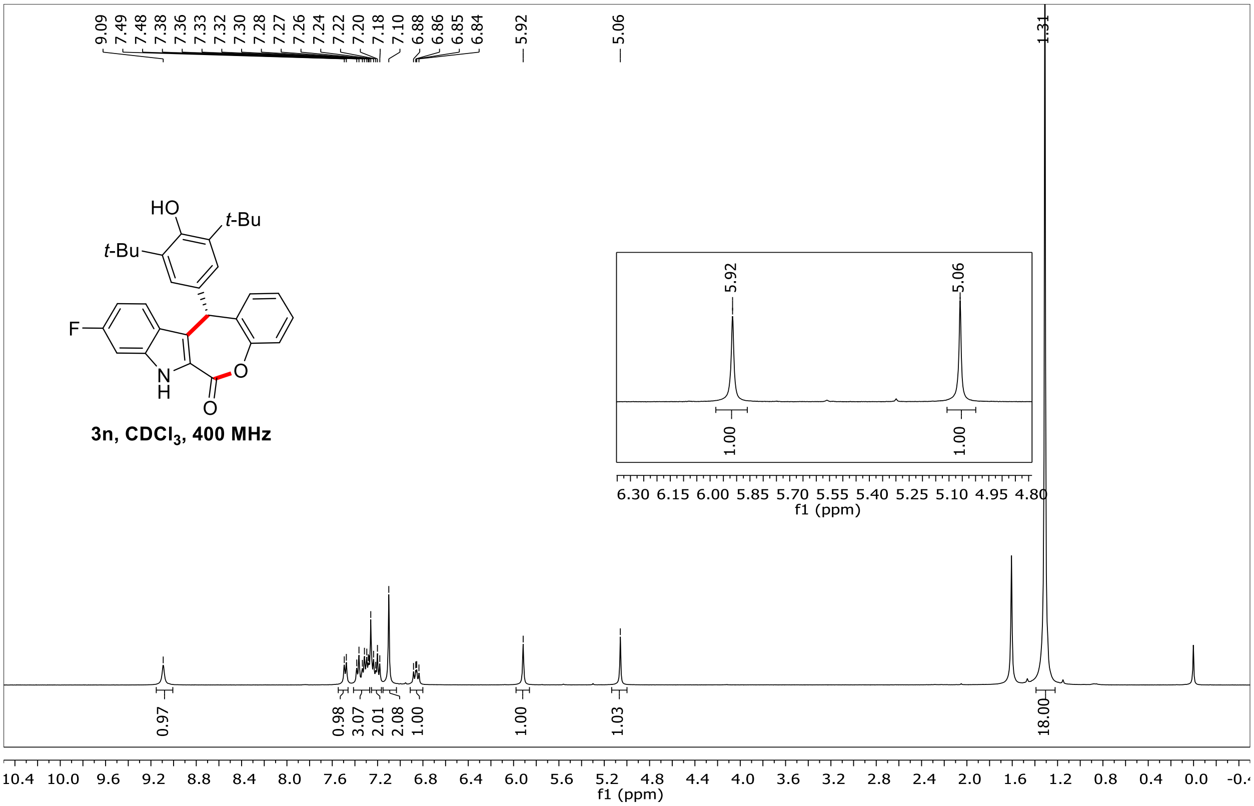

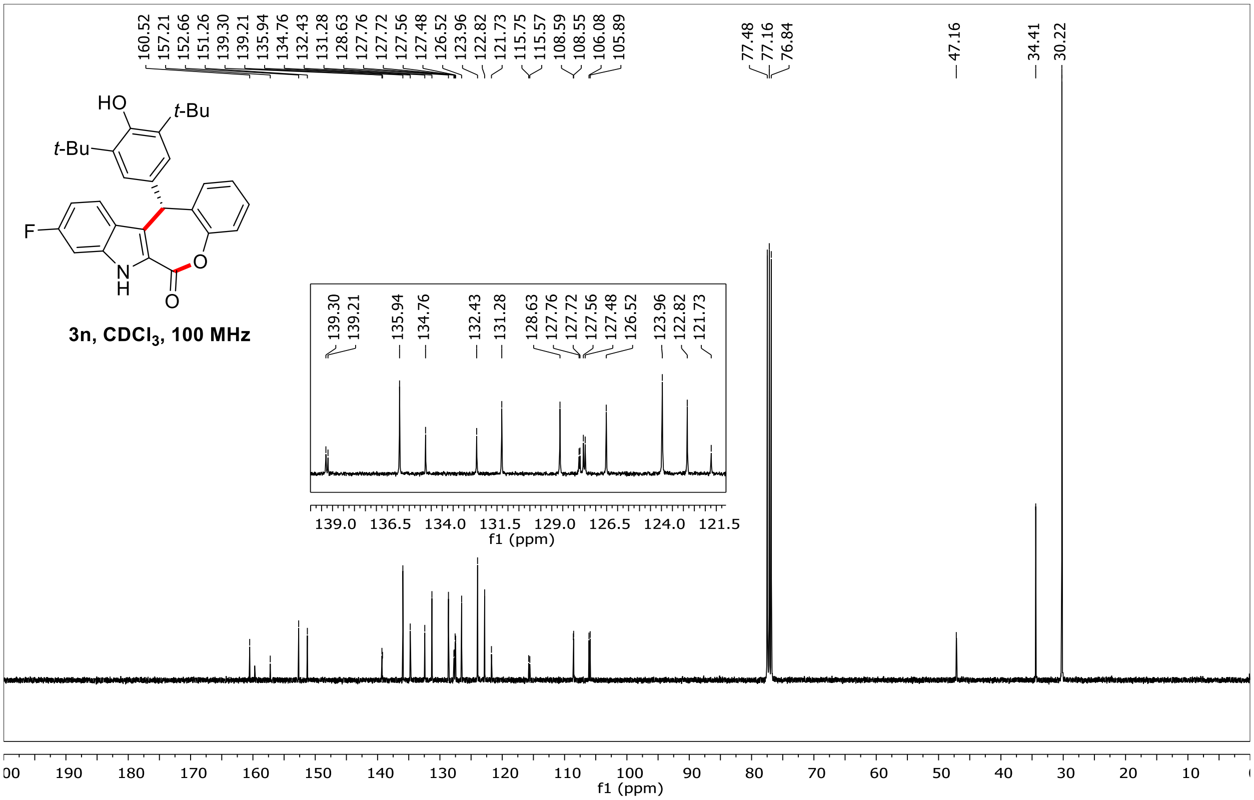

(*S*)-12-(3,5-Di-*tert*-butyl-4-hydroxyphenyl)-9-fluoro-7,12-dihydro-6*H*-benzo[6,7]oxepino[3,4-*b*]indol-6-one (3n)

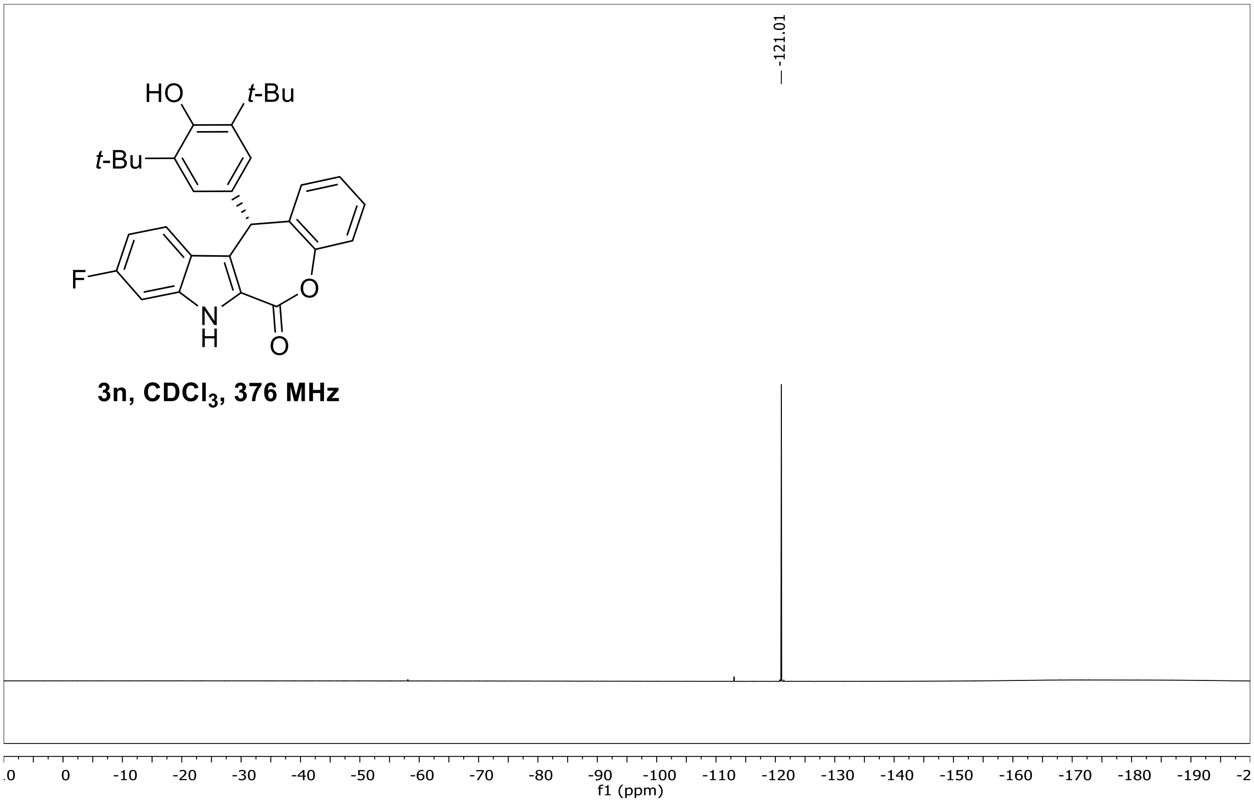

(*S*)-12-(3,5-Di-*tert*-butyl-4-hydroxyphenyl)-9-phenyl-7,12-dihydro-6*H*-benzo[6,7]oxepino[3,4-*b*]indol-6-one (3o)

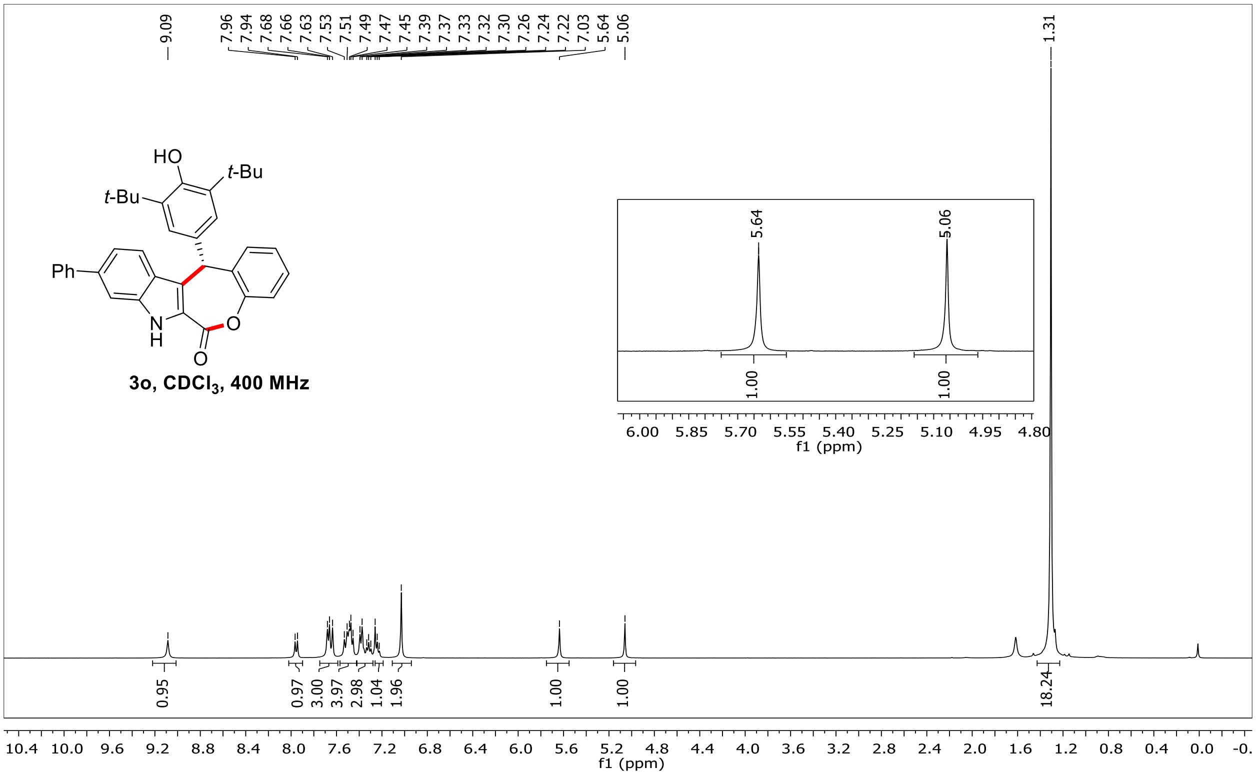

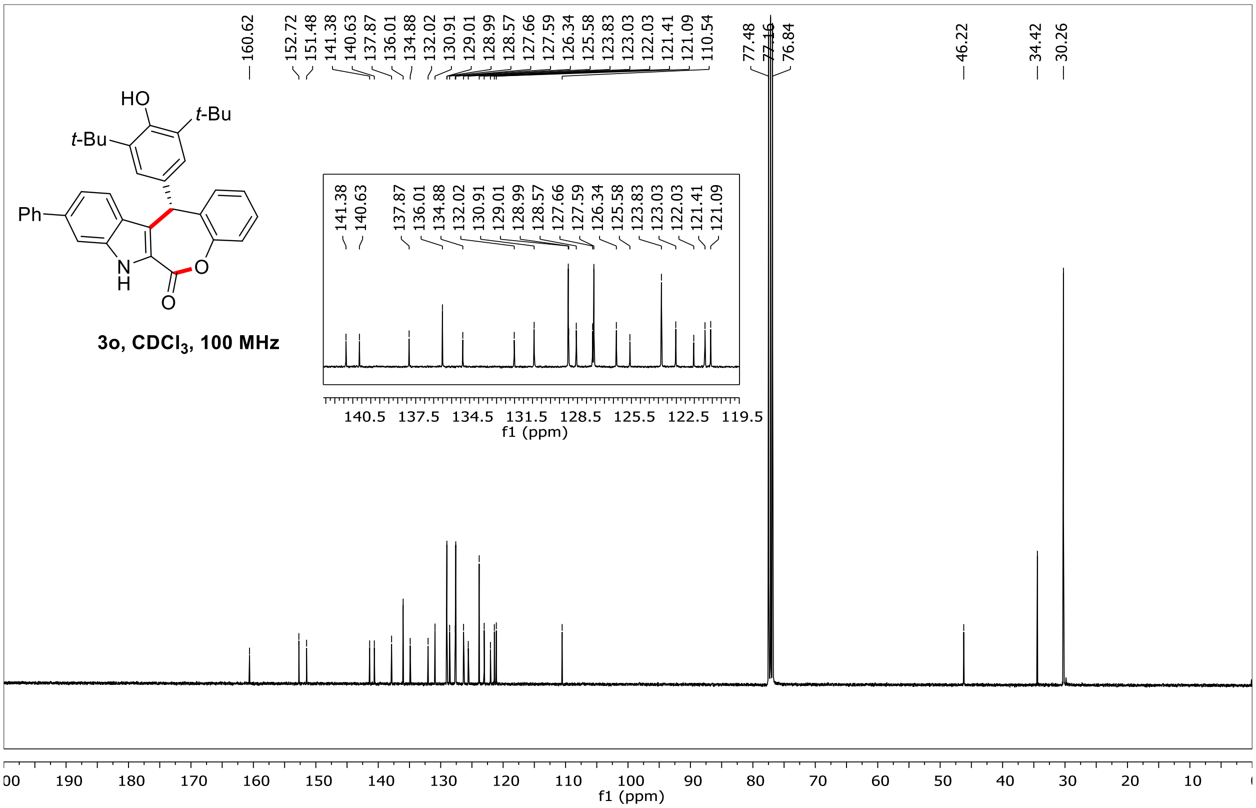

(*S*)-12-(3,5-Di-*tert*-butyl-4-hydroxyphenyl)-9-(2-fluorophenyl)-7,12-dihydro-6*H*-benzo [6,7] oxepino[3,4-*b*] indol-6-one (3p)

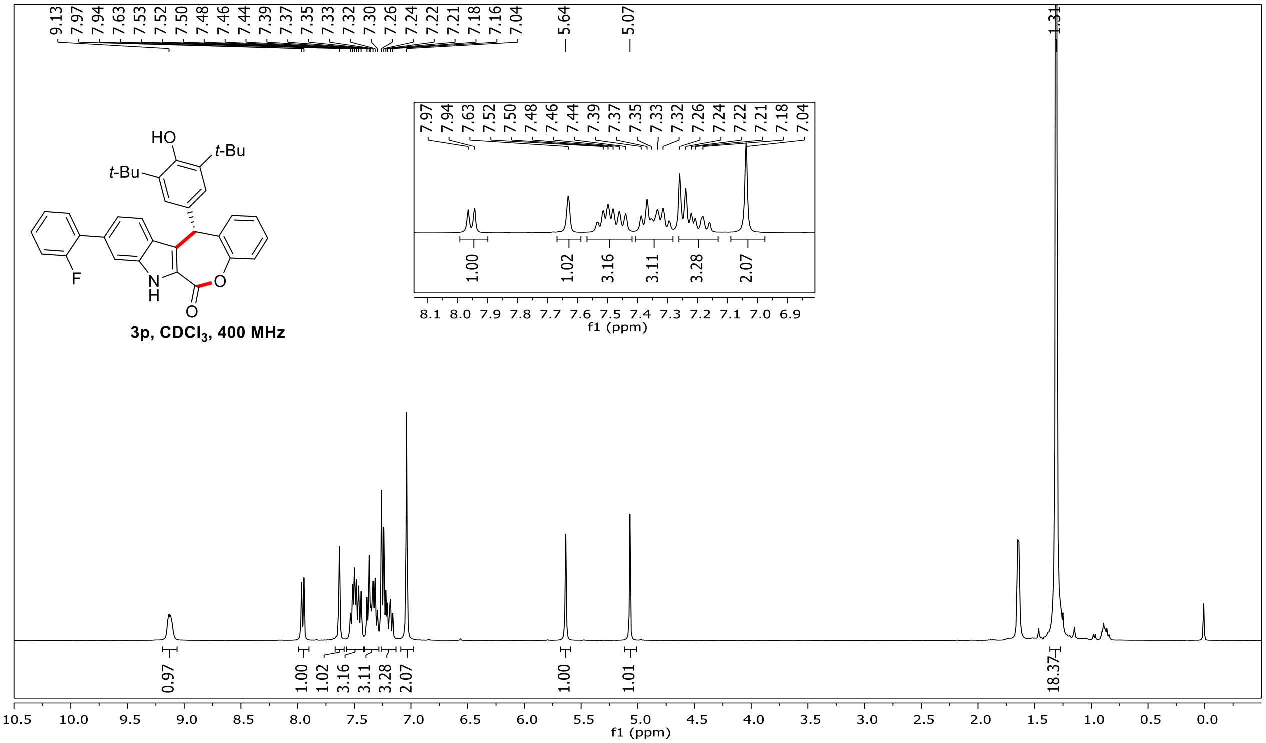

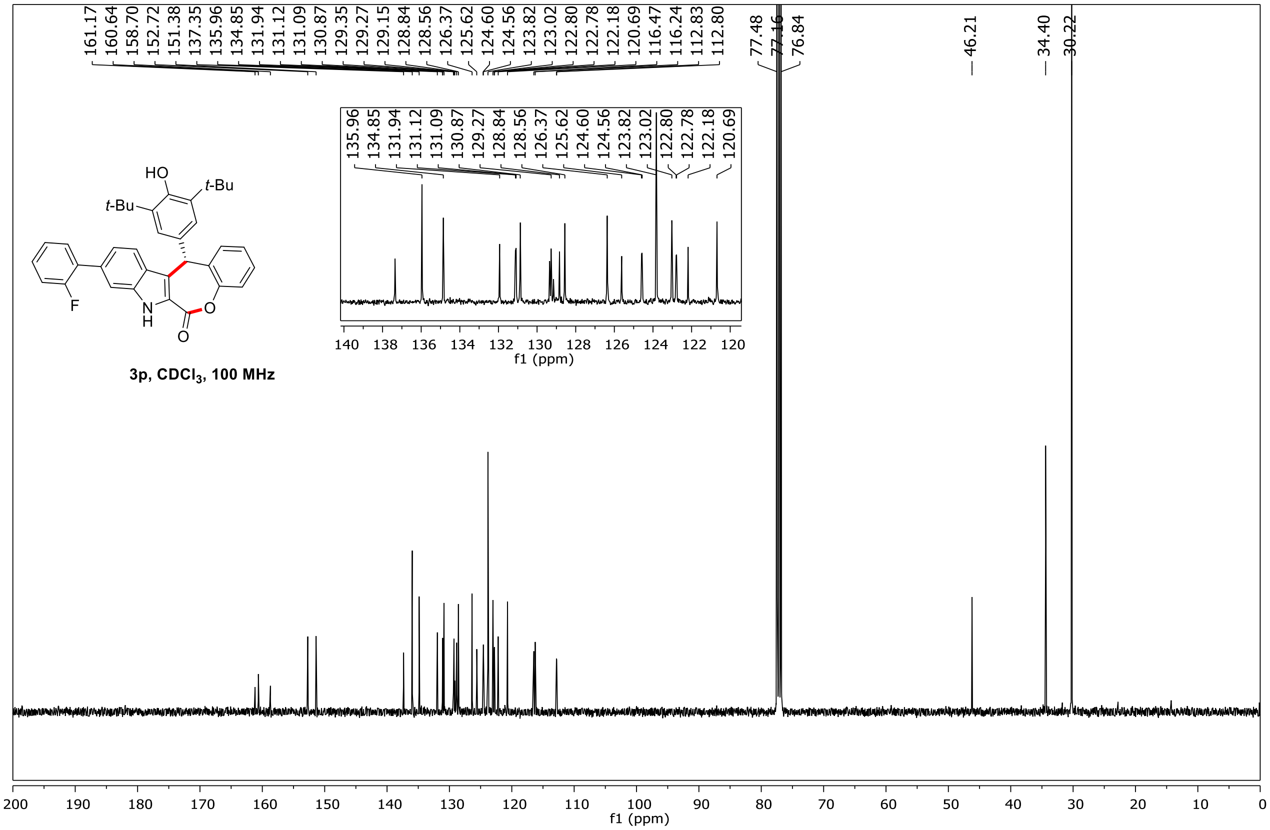

(*S*)-12-(3,5-Di-*tert*-butyl-4-hydroxyphenyl)-9-(2-fluorophenyl)-7,12-dihydro-6*H*-benzo [6,7] oxepino[3,4-*b*] indol-6-one (3p)

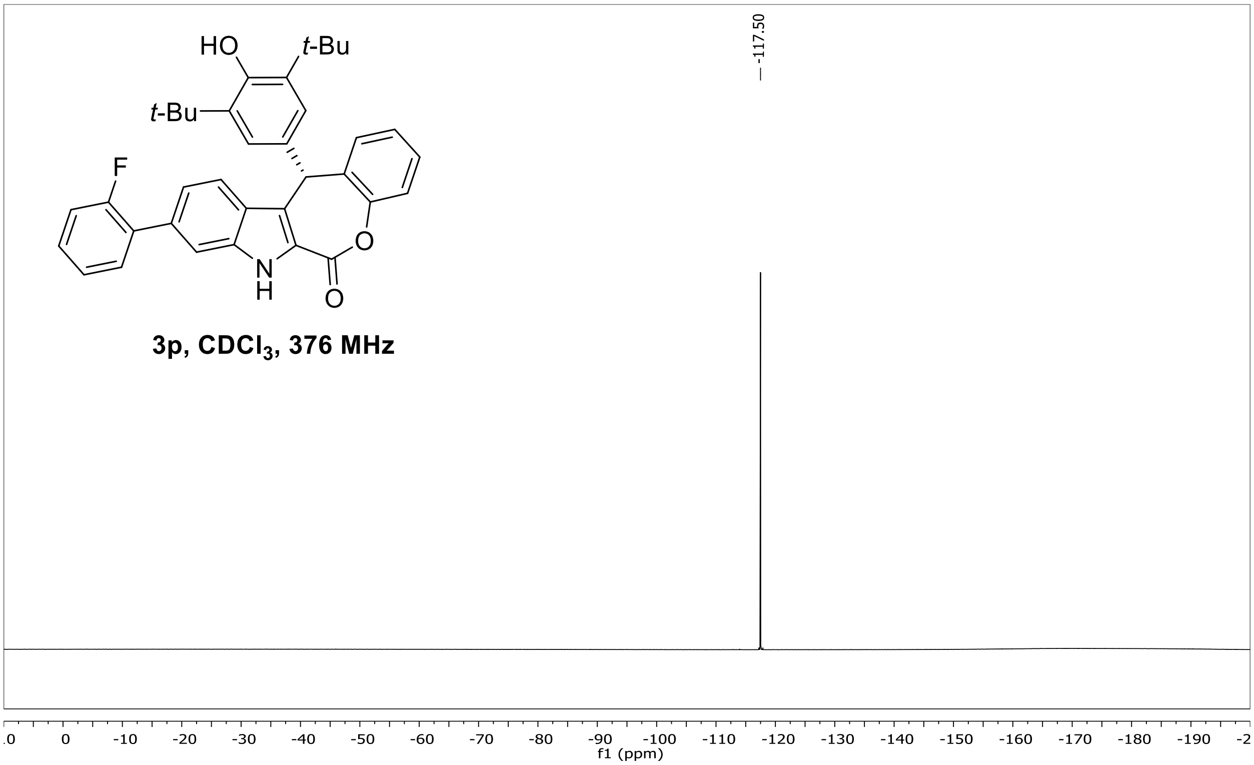

(*S*)-12-(3,5-di-*Tert*-butyl-4-hydroxyphenyl)-9-(2,6-dimethylphenyl)-7,12-dihydro-6*H*-benzo[6,7]oxepino[3,4-*b*]indol-6-one (3q)

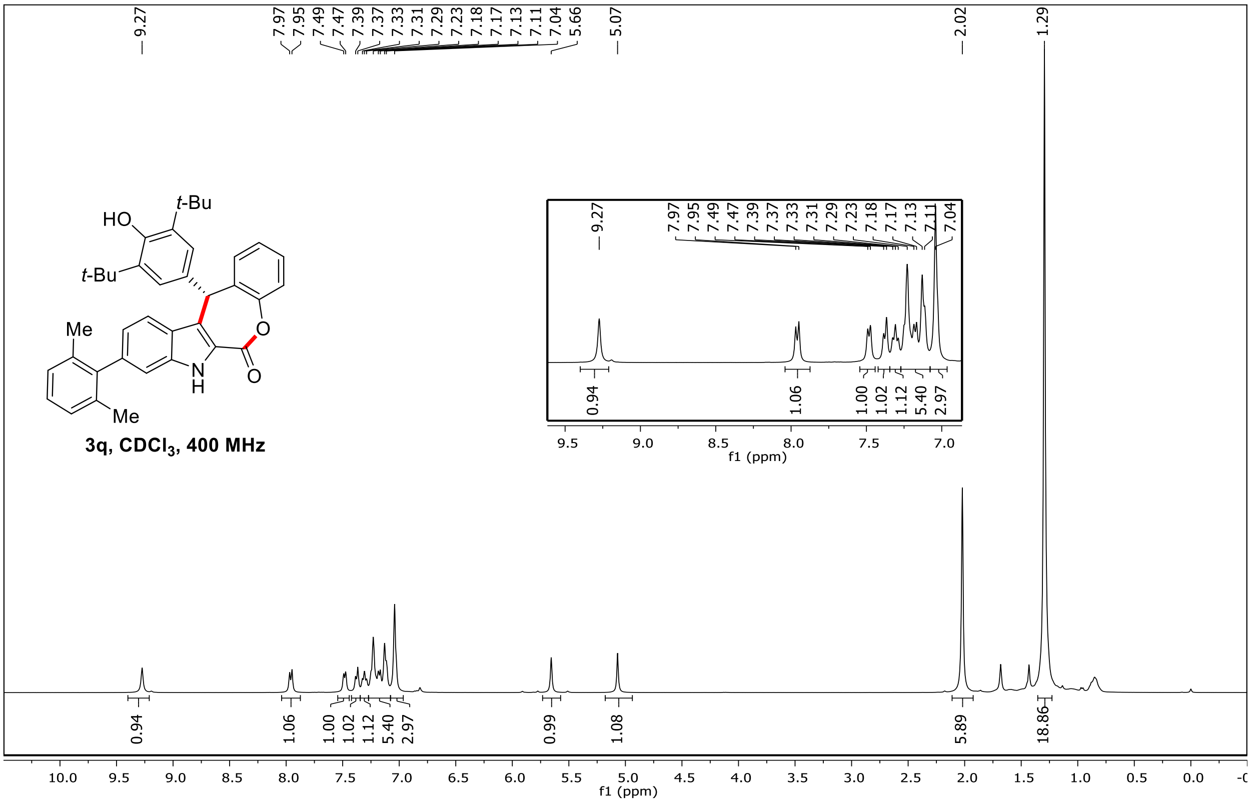

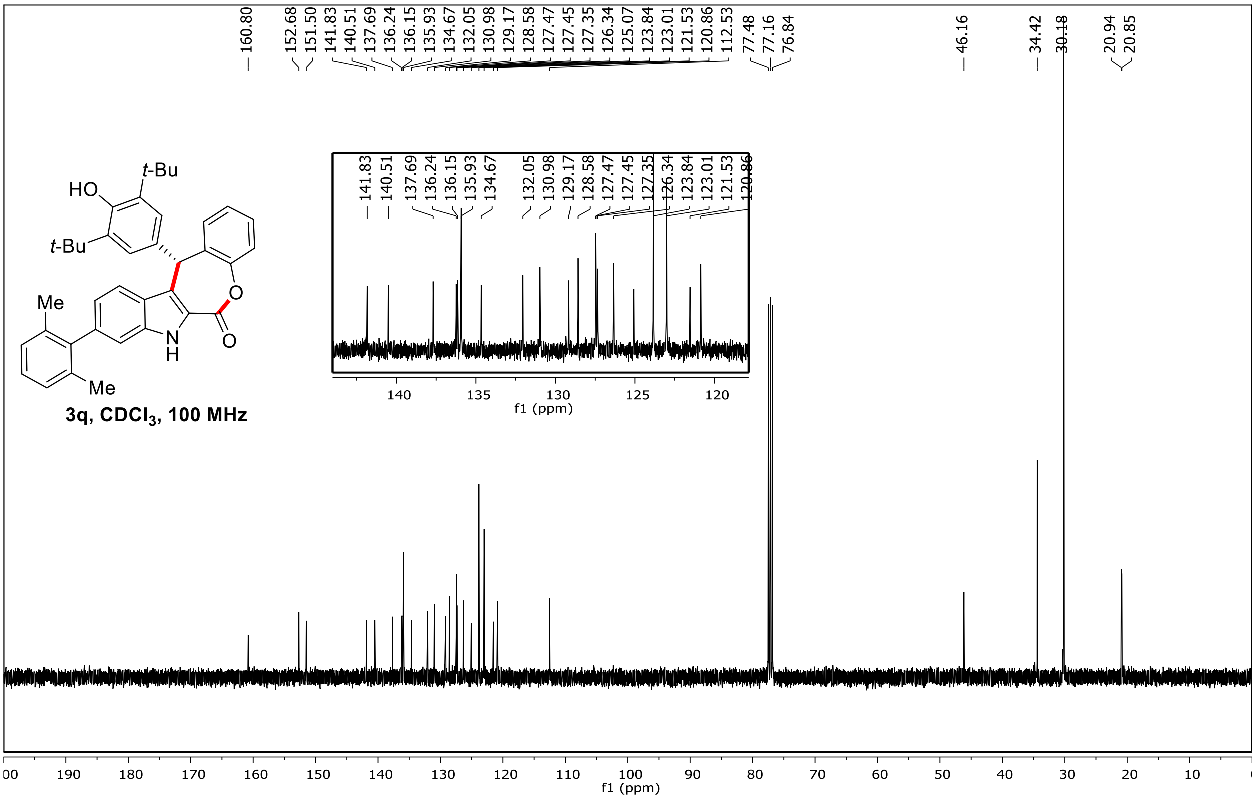

(*S*)-12-(3,5-Di-*tert*-butyl-4-hydroxyphenyl)-9-(2,3-dimethylphenyl)-7,12-dihydro-6*H*-benzo [6,7] oxepino[3,4-*b*] indol-6-one (3r)

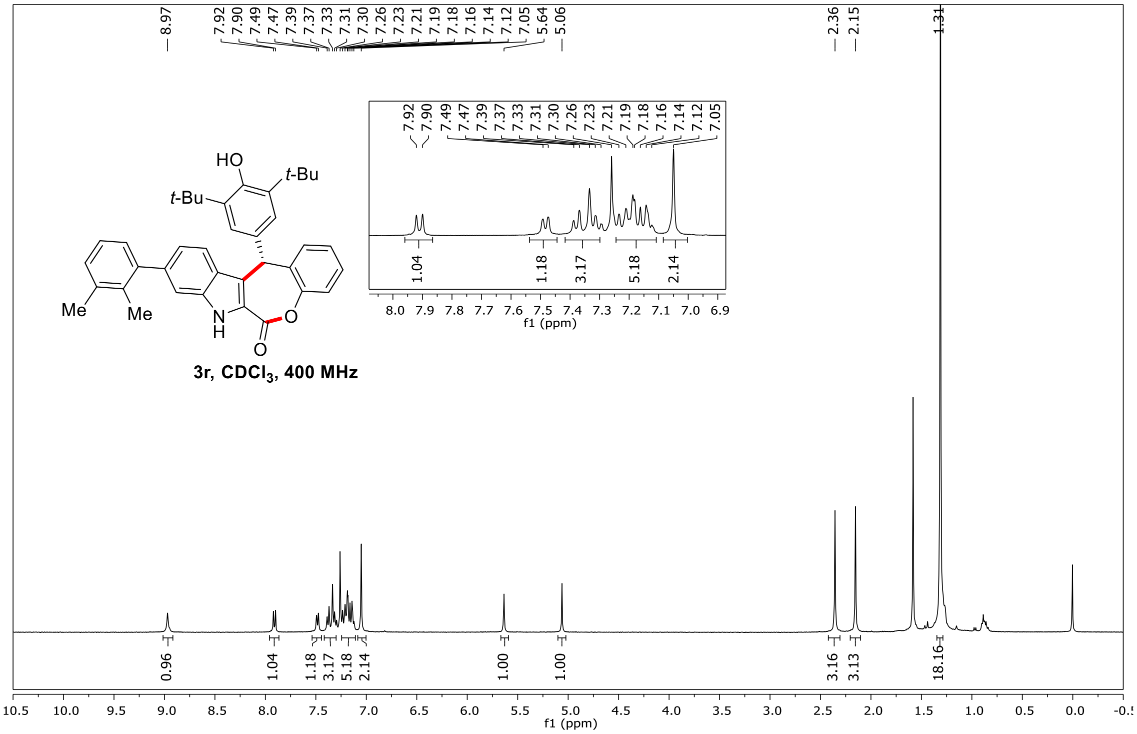

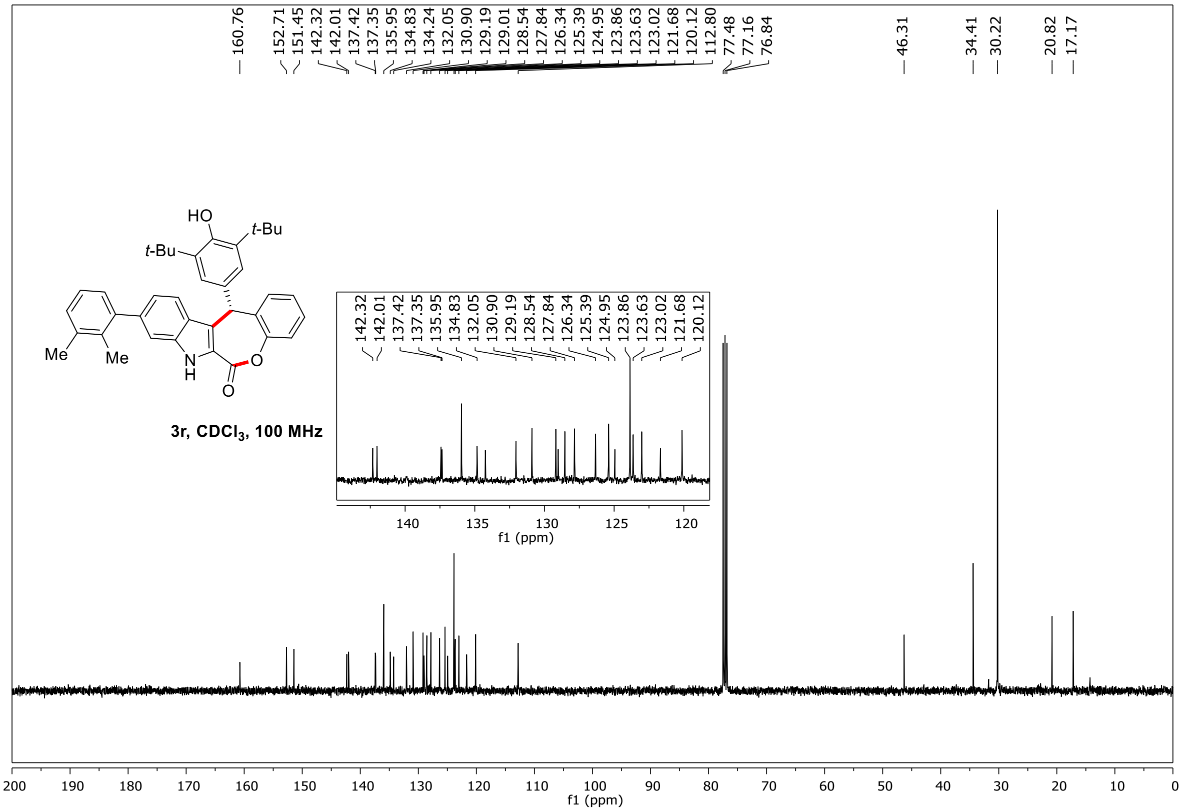

(*S*)-12-(3,5-Di-*tert*-butyl-4-hydroxyphenyl)-8-methoxy-7,12-dihydro-6*H*-benzo[6,7]oxepino[3,4-*b*]indol-6-one (3s)

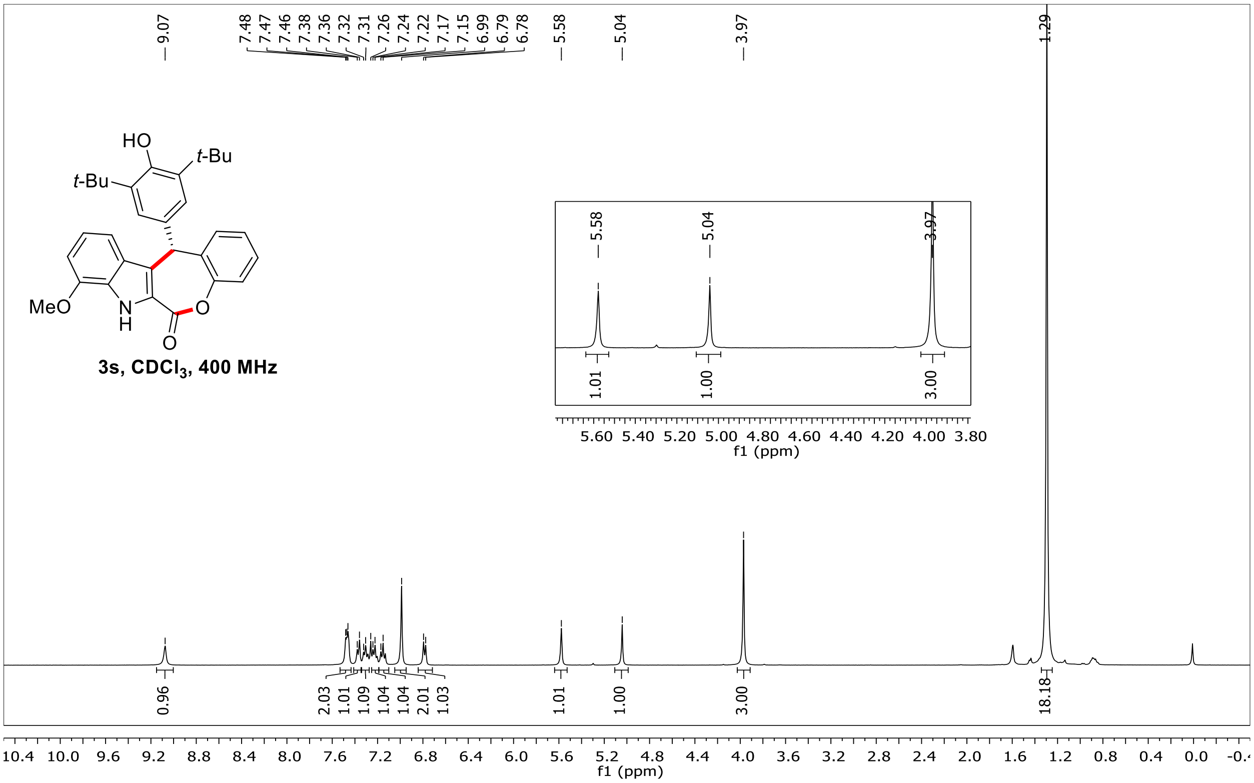

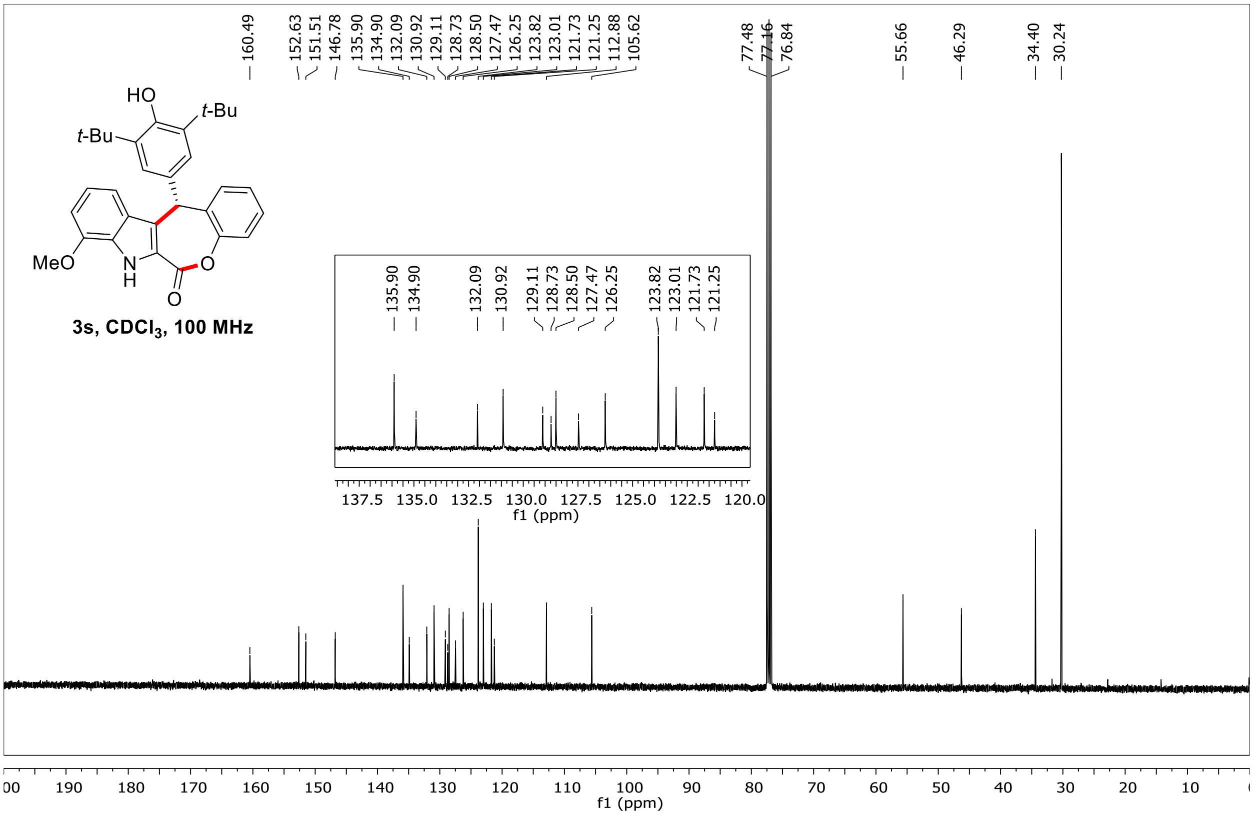

(*S*)-7-(3,5-Di-*tert*-butyl-4-hydroxyphenyl)-7,14-dihydro-13*H*-benzo[*g*]benzo[6,7]oxepino[3,4-*b*]indol-13-one (3t)

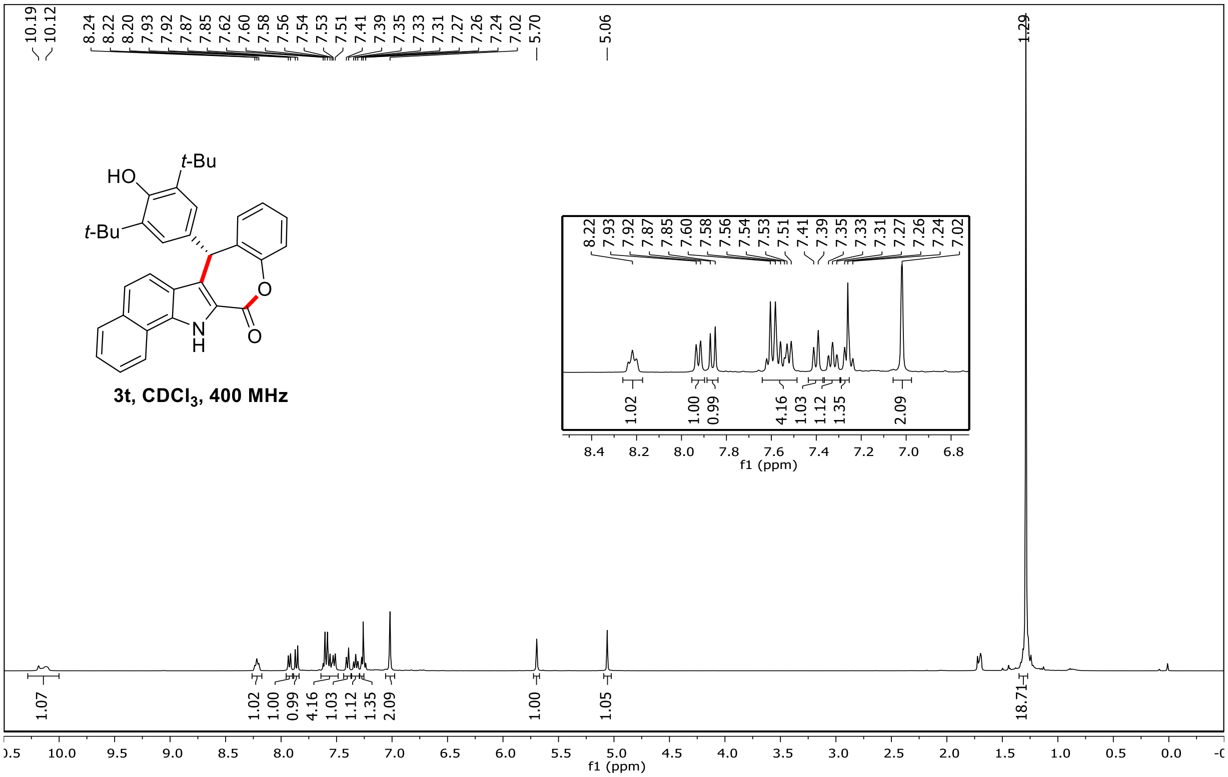

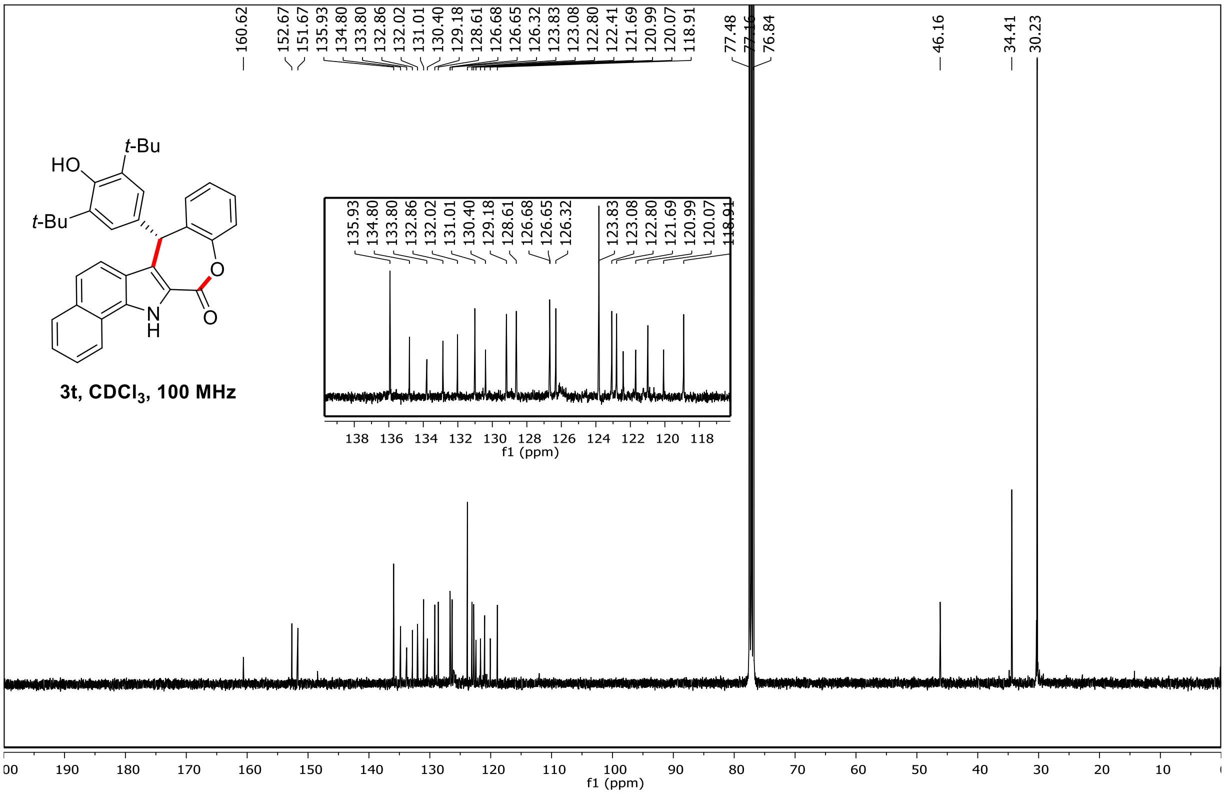

(*S*)-12-(3,5-di-*Tert*-butyl-4-hydroxyphenyl)-9,11-dimethyl-7,12-dihydro-6*H*-benzo[6,7]oxepino[3,4-b]indol-6-one (3u)

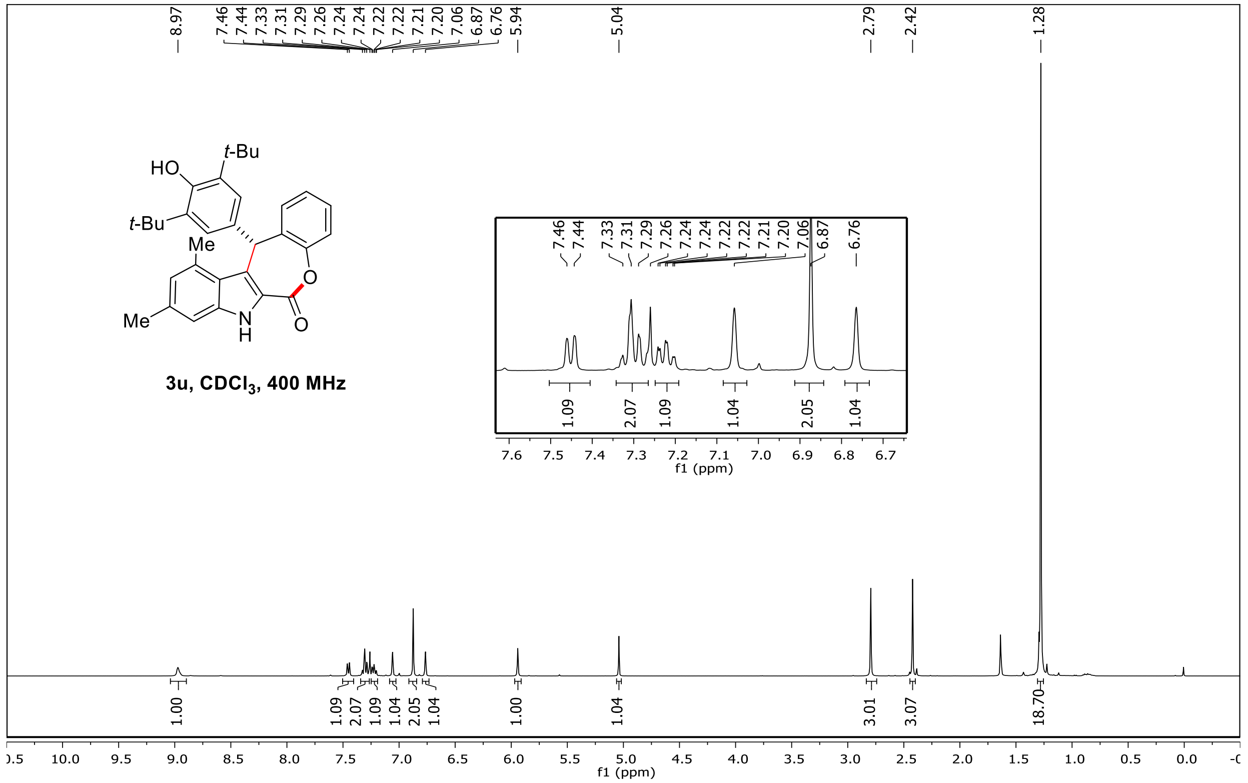

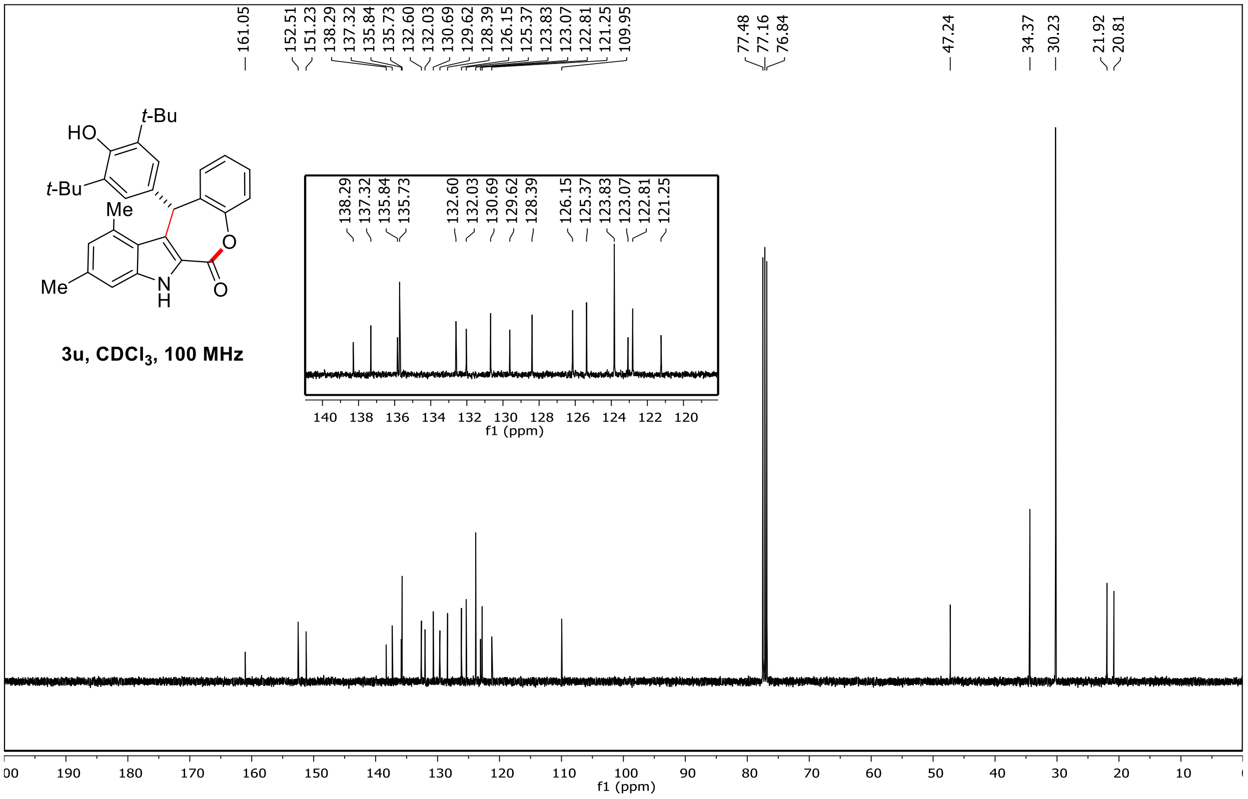

(*S*)-2-Bromo-12-(3,5-di-*tert*-butyl-4-hydroxyphenyl)-7,12-dihydro-6*H*-benzo[6,7]oxepino[3,4-b]indol-6-one (3v)

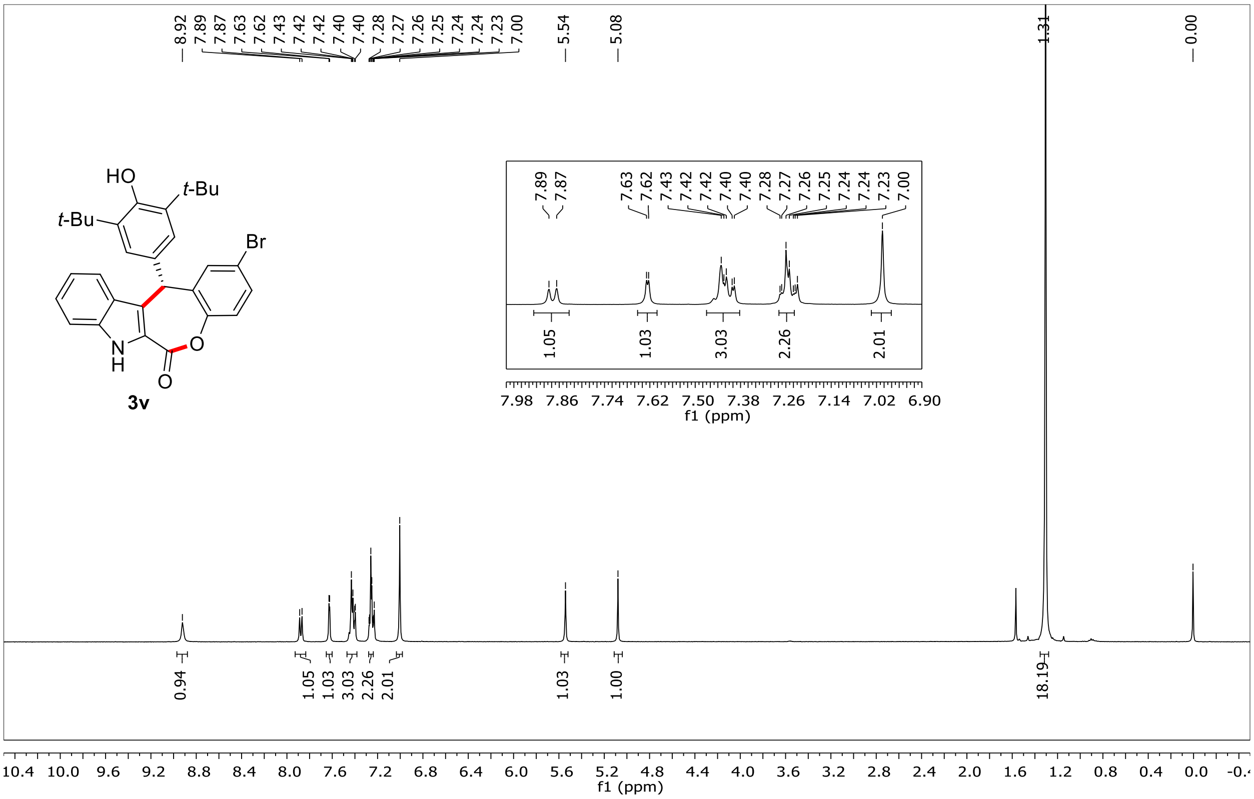

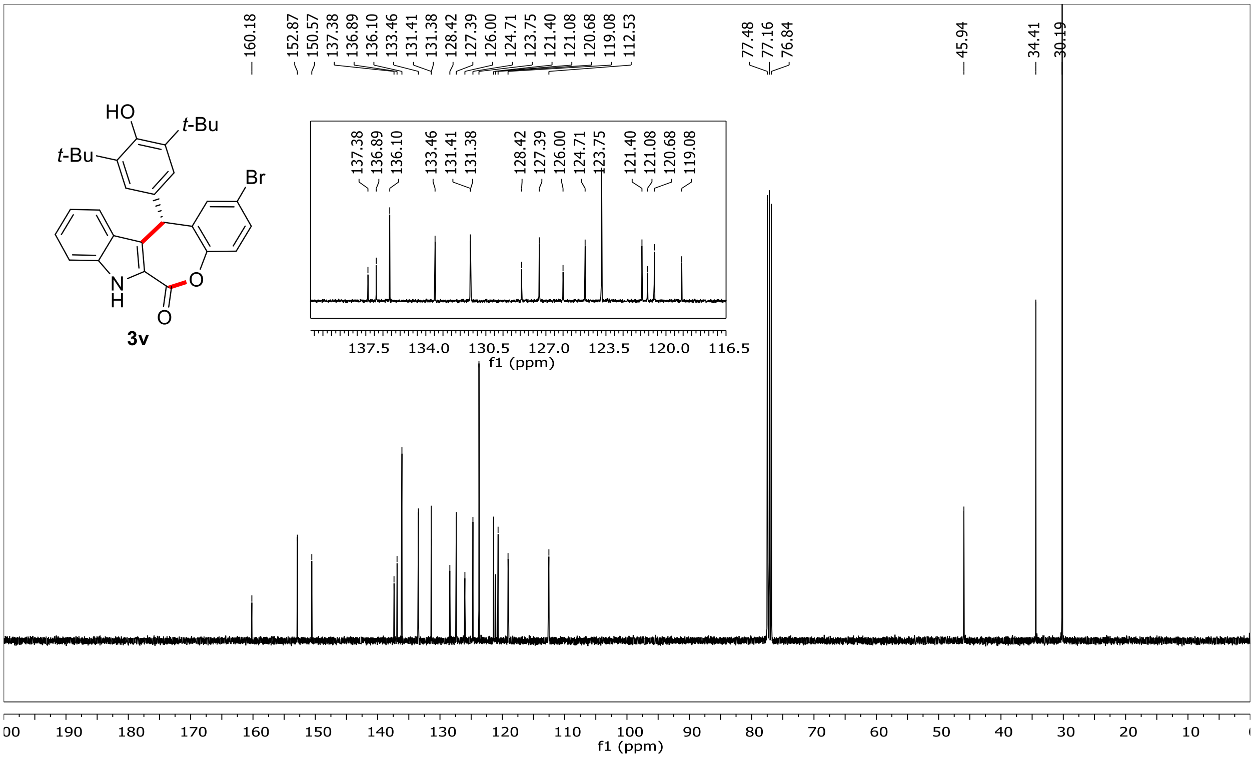

(*S*)-2-Chloro-12-(3,5-di-*tert*-butyl-4-hydroxyphenyl)-7,12-dihydro-6*H*-benzo[6,7]oxepino[3,4-b]indol-6-one (3w)

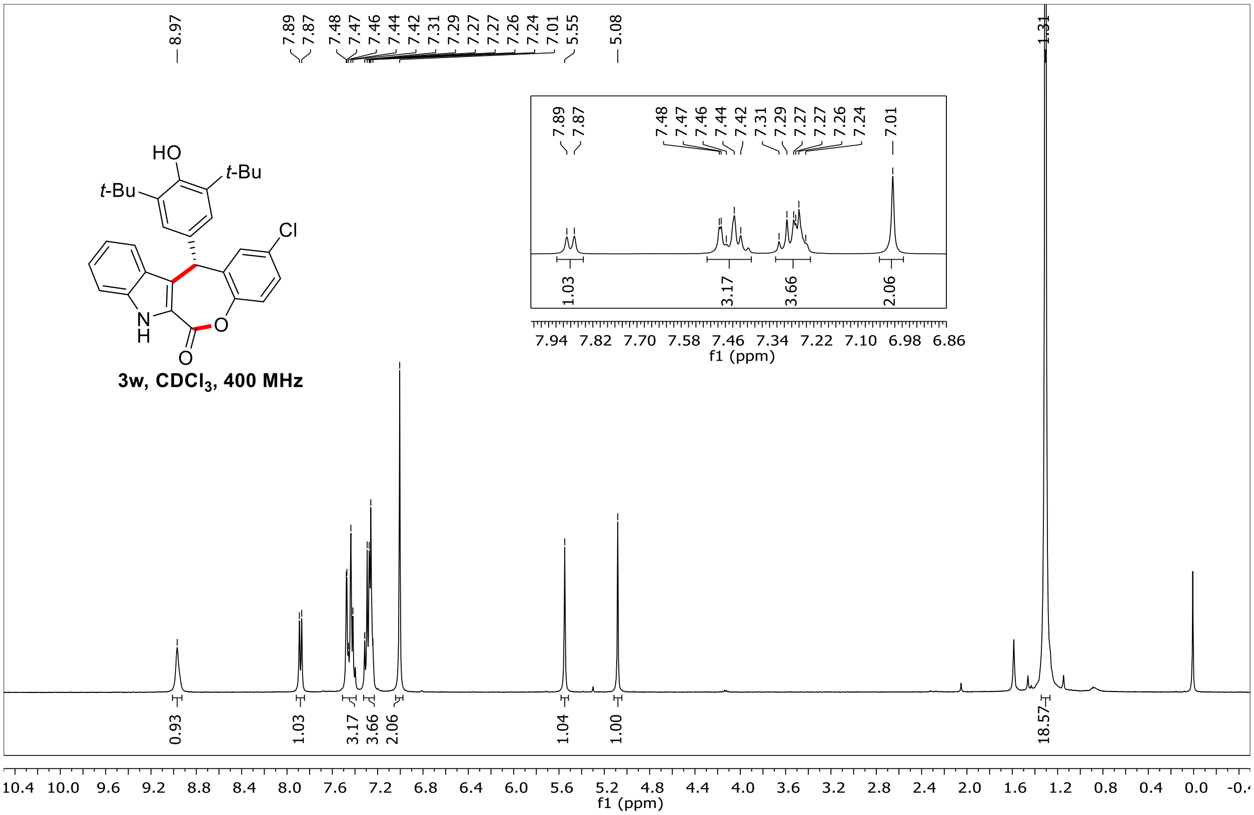

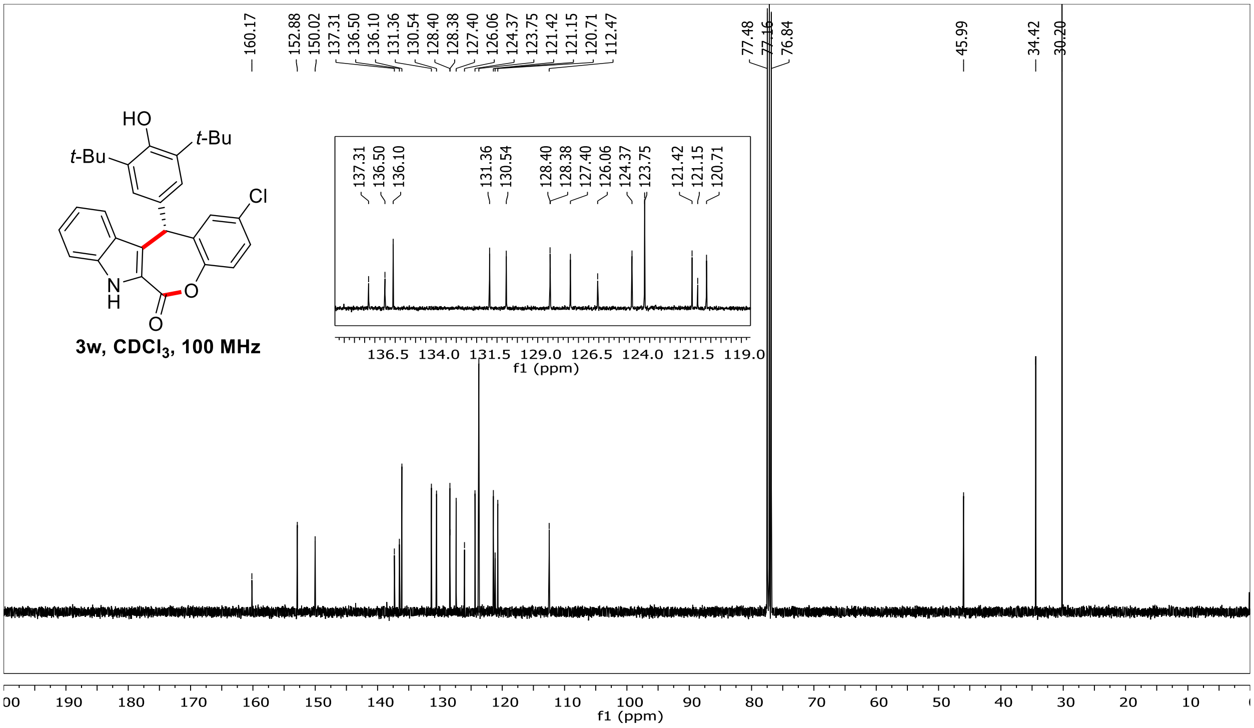

(*S*)-12-(3,5-Di-*tert*-butyl-4-hydroxyphenyl)-2-phenyl-7,12-dihydro-6*H*-benzo [6,7]oxepino[3,4-b]indol-6-one (3x)

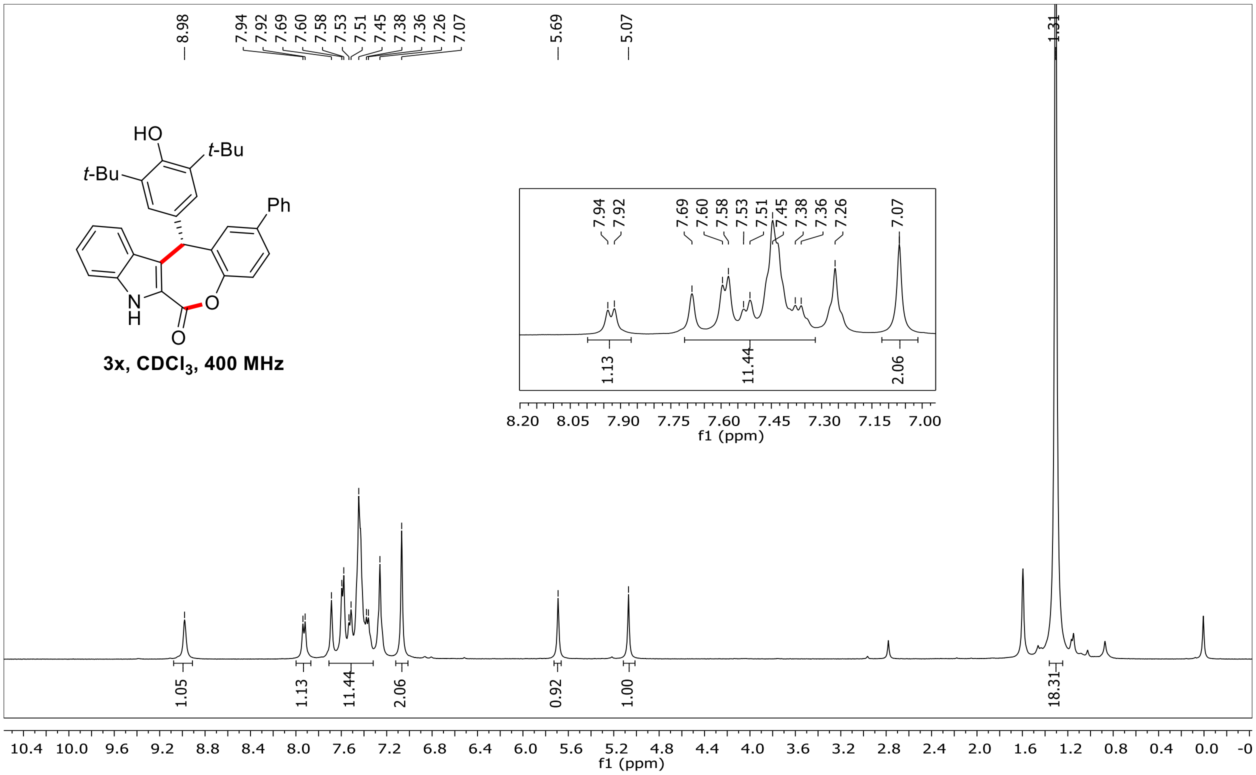

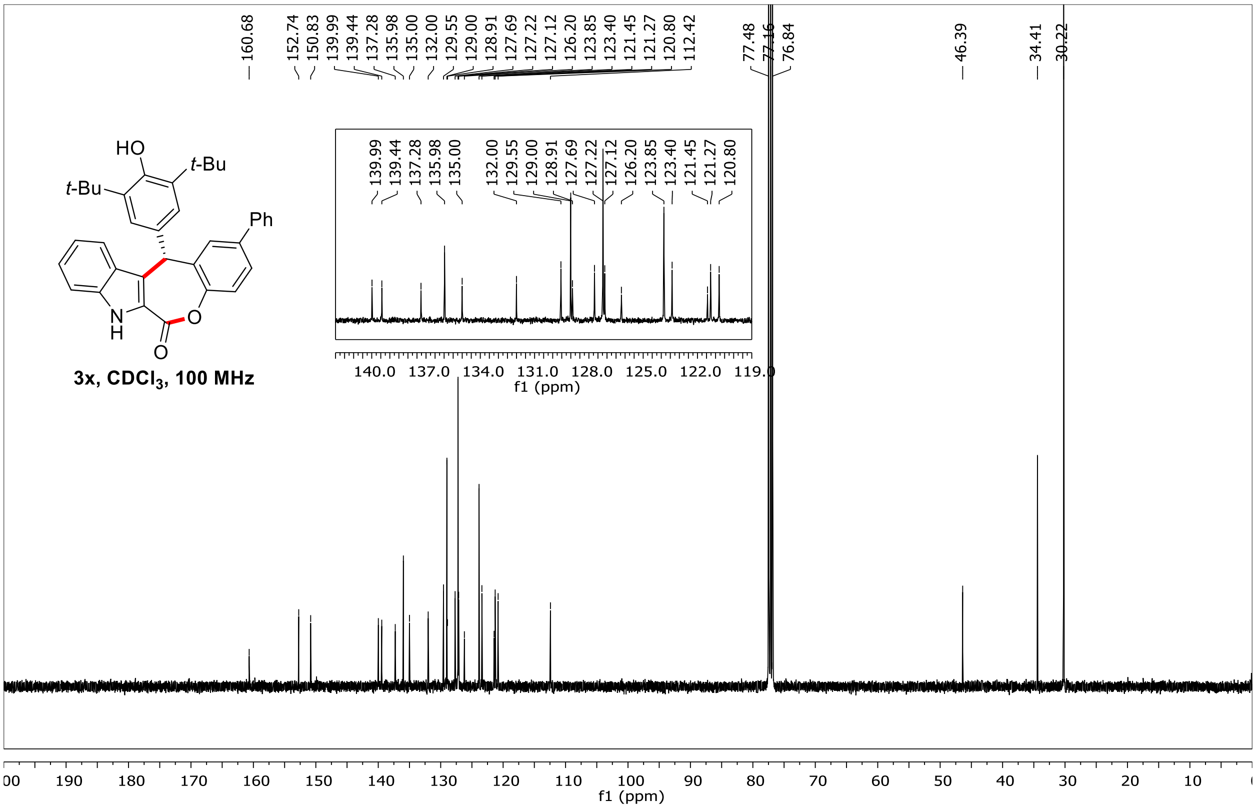

(*S*)-12-(3,5-Di-*tert*-butyl-4-hydroxyphenyl)-2-methoxy-7,12-dihydro-6*H* benzo[6,7]oxepino[3, 4-b]indol-6-one (3y)

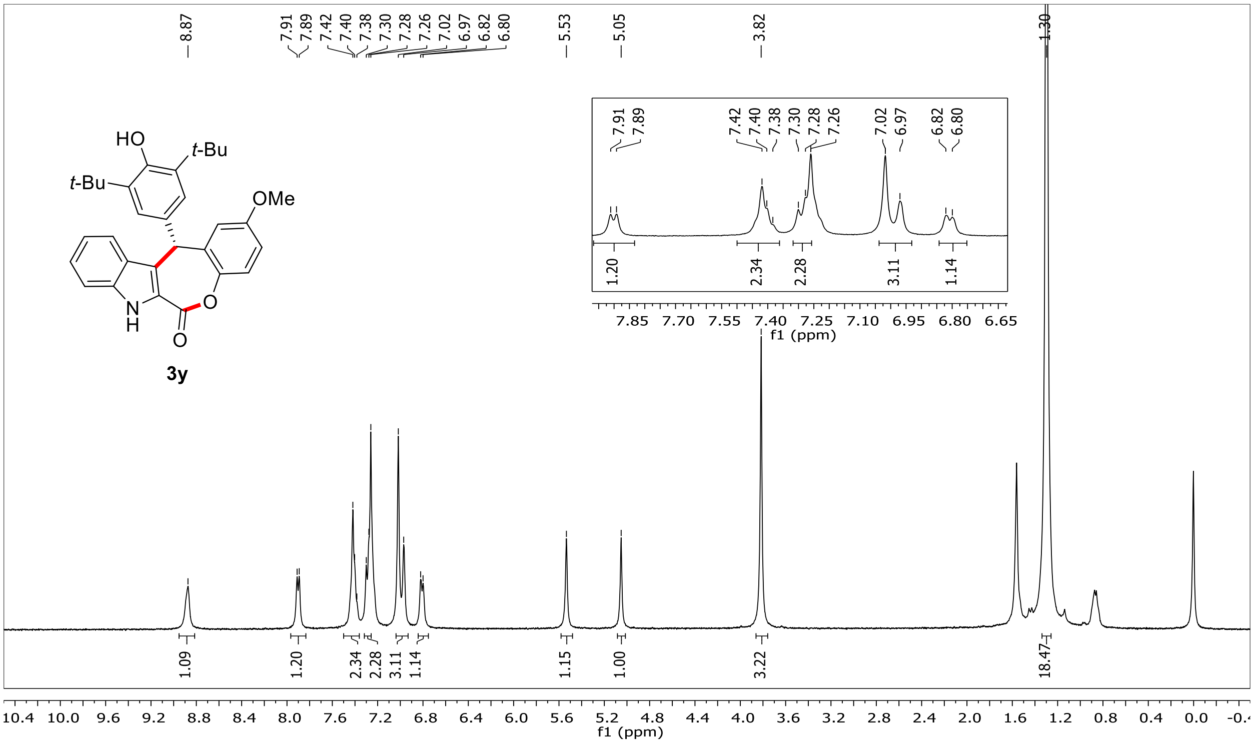

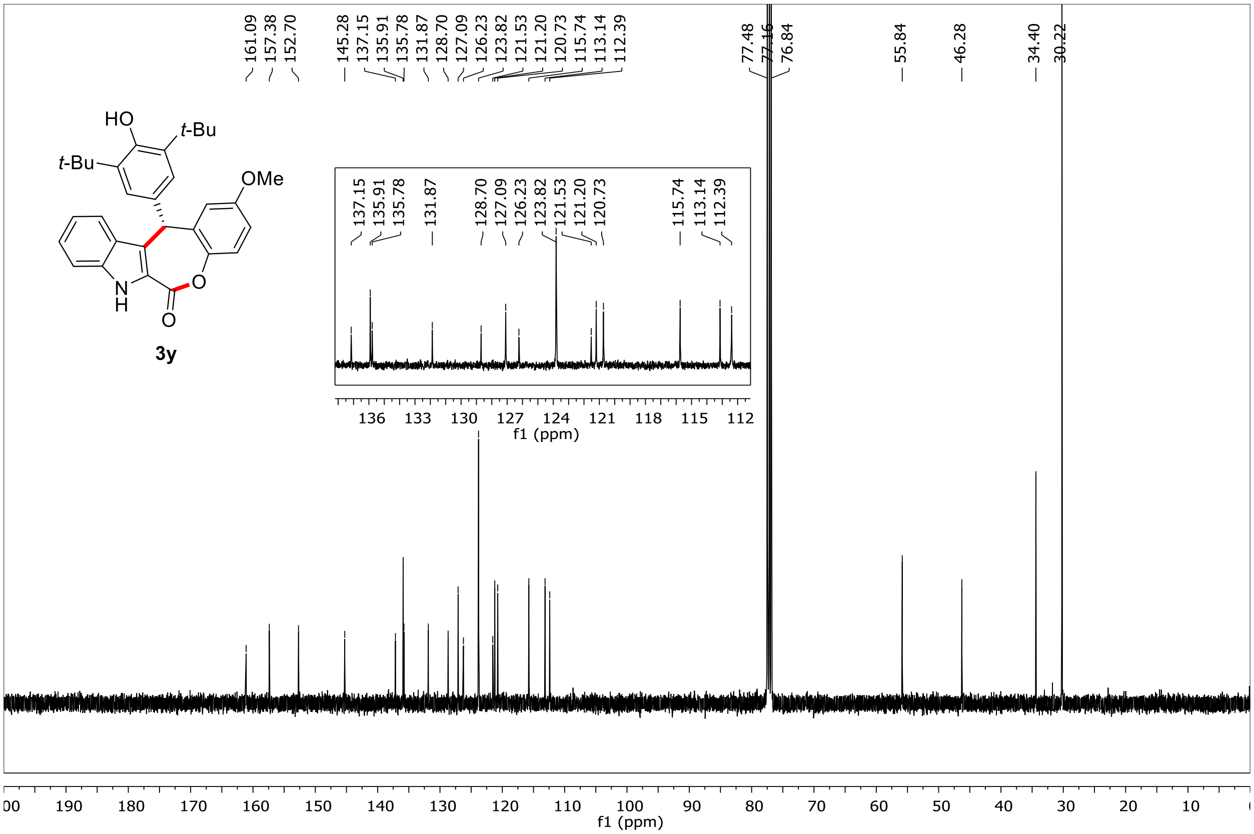

(*S*)-12-(3,5-Di-*tert*-butyl-4-hydroxyphenyl)-3-methyl-7,12-dihydro-6*H*-benzo[6,7]oxepino[3,4-b]indol-6-one (3z)

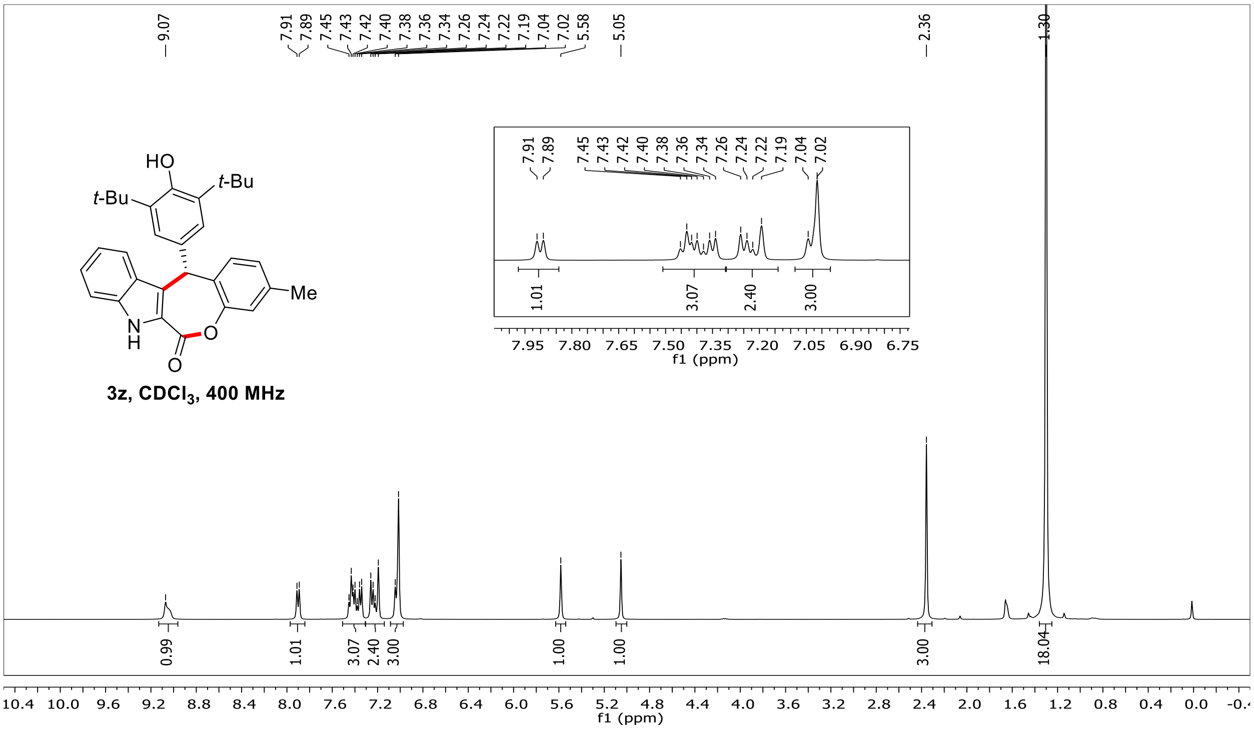

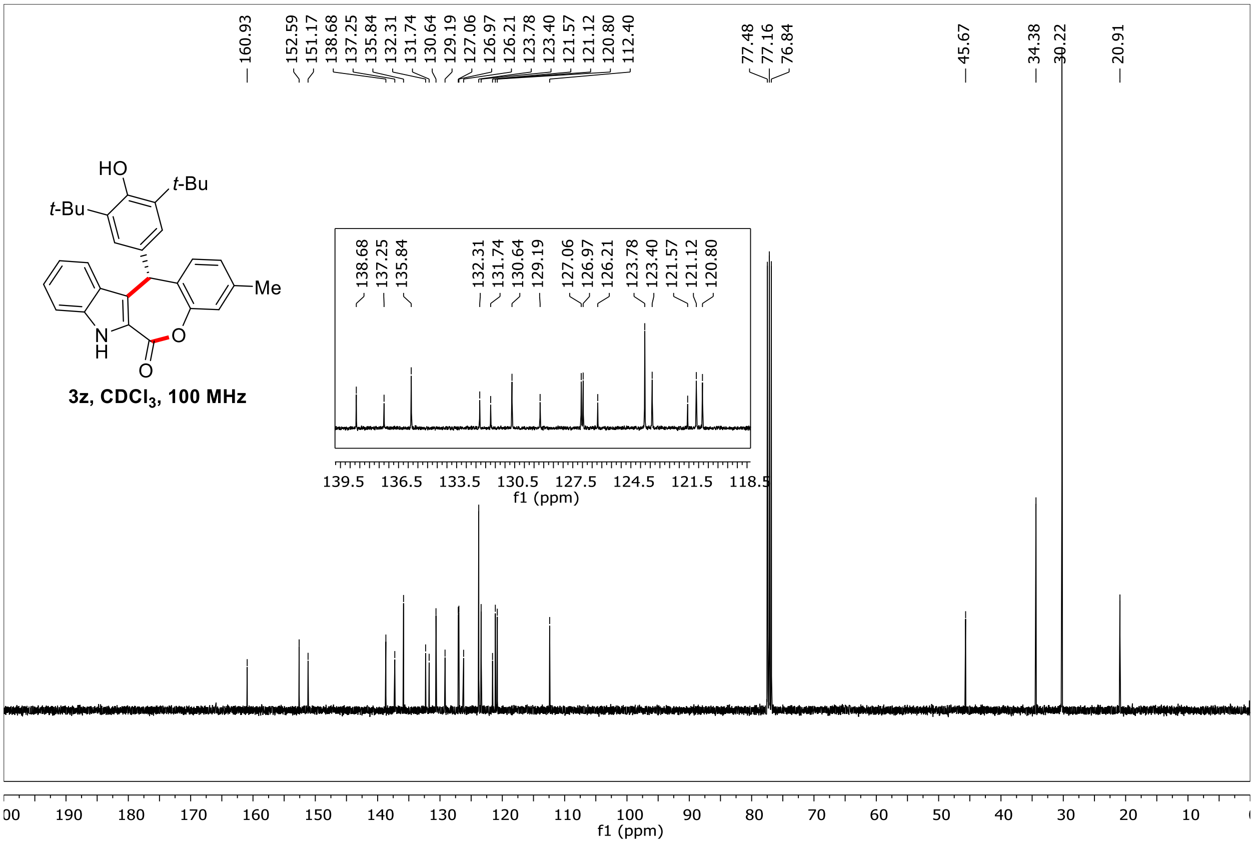

(*S*)-12-(3,5-Di-*tert*-butyl-4-hydroxyphenyl)-4-methoxy-7,12-dihydro-6*H*-benzo[6,7]oxepino[3, 4-b]indol-6-one (3aa)

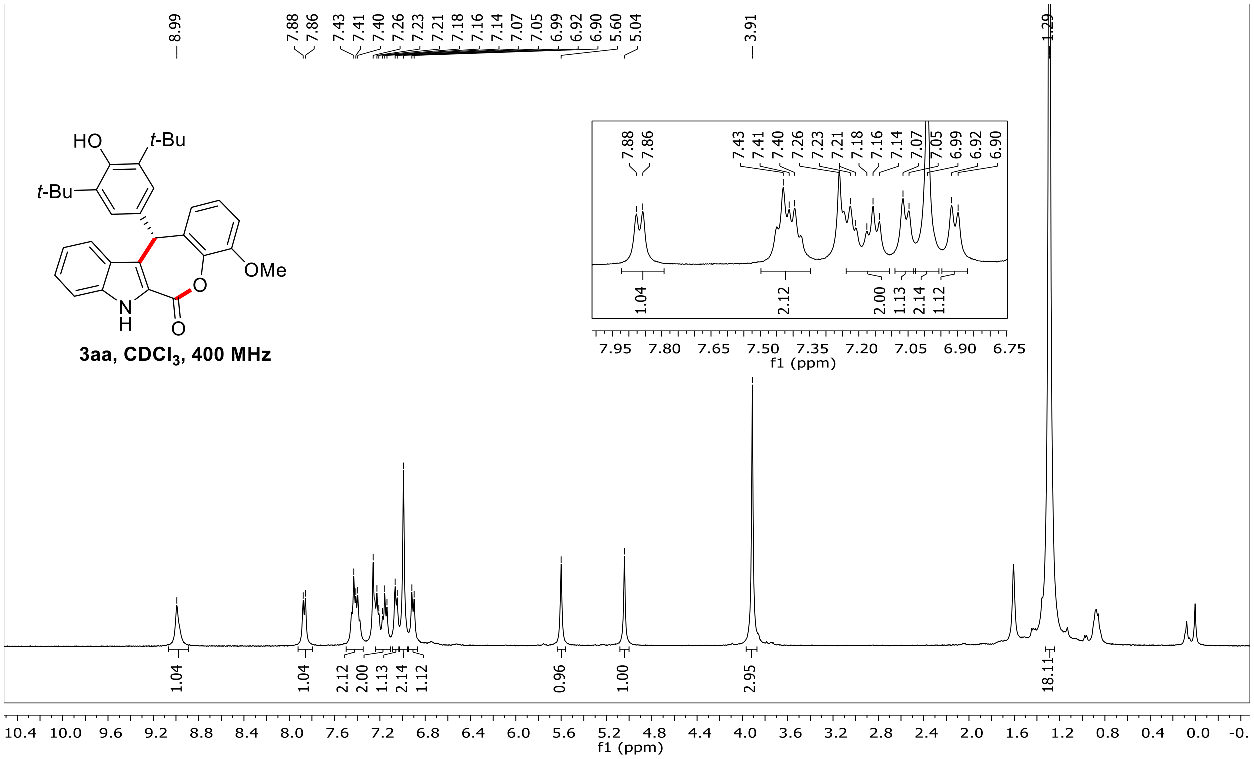

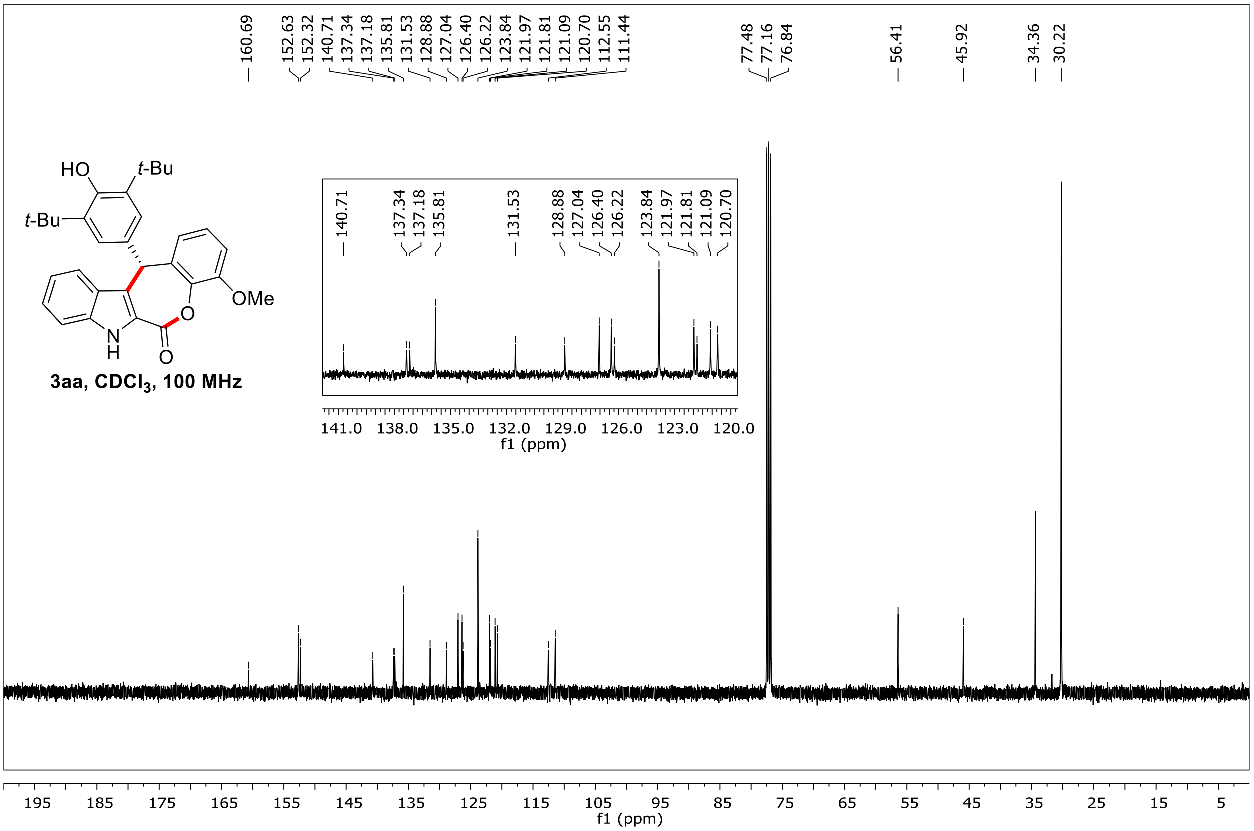

2-((3,5-Di-*tert*-butyl-4-oxocyclohexa-2,5-dien-1-ylidene)methyl)phenyl-1*H*-indole-2-carboxylate (3a')

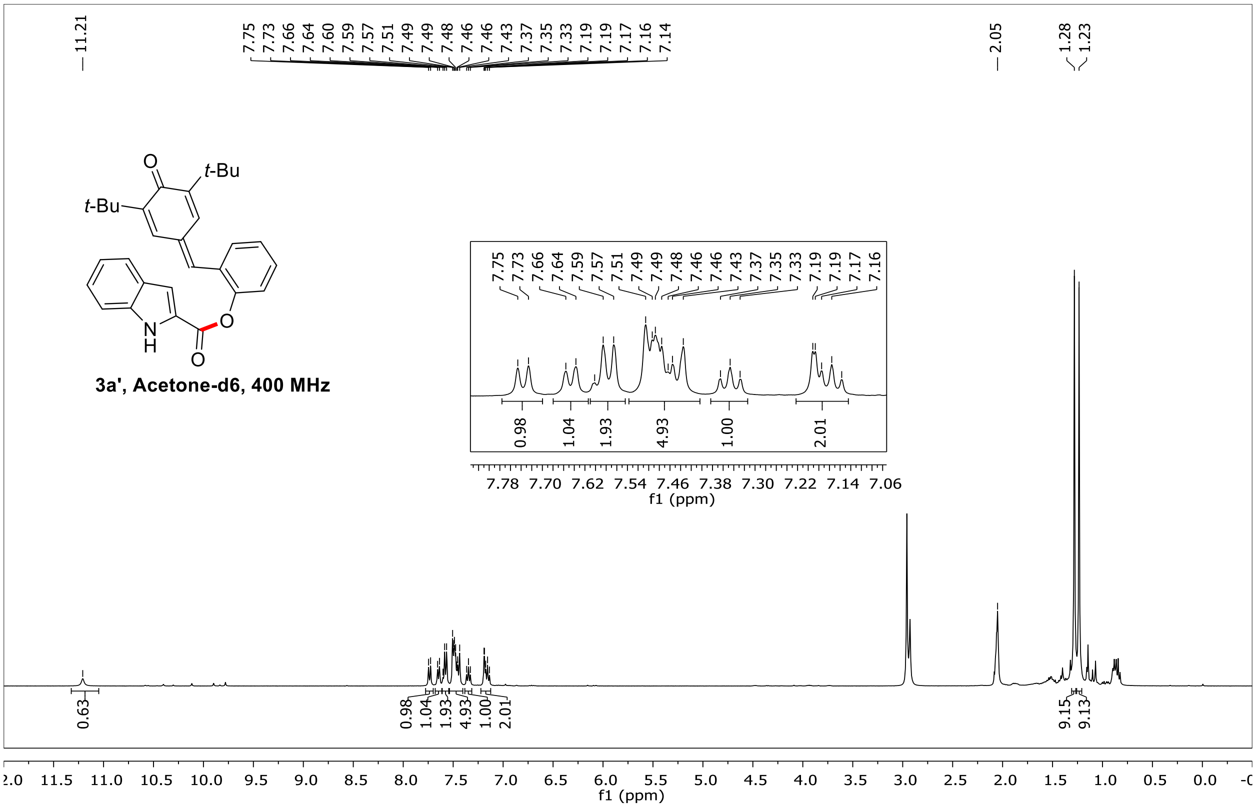

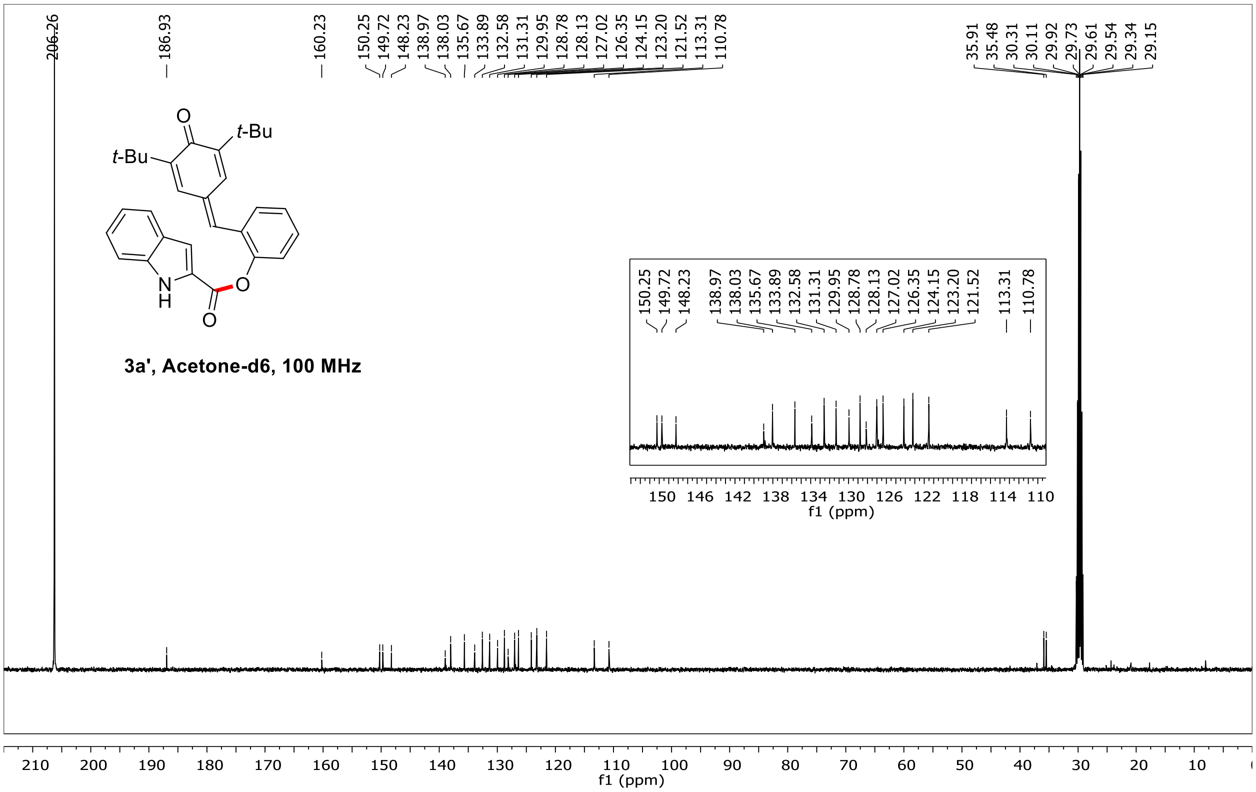

3-((3,5-Di-*tert*-butyl-4-hydroxyphenyl)(2-hydroxyphenyl)methyl)-1*H*-indole-2-carbaldehyde (3a”)

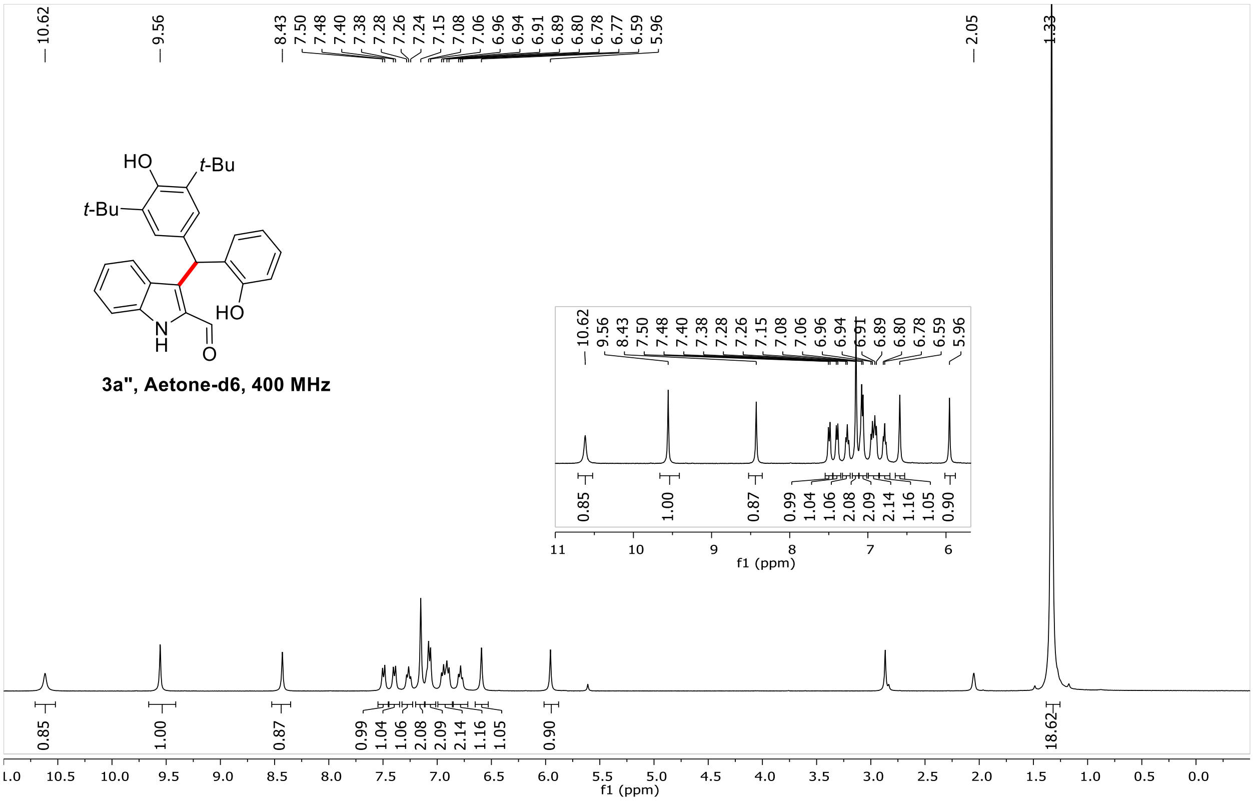

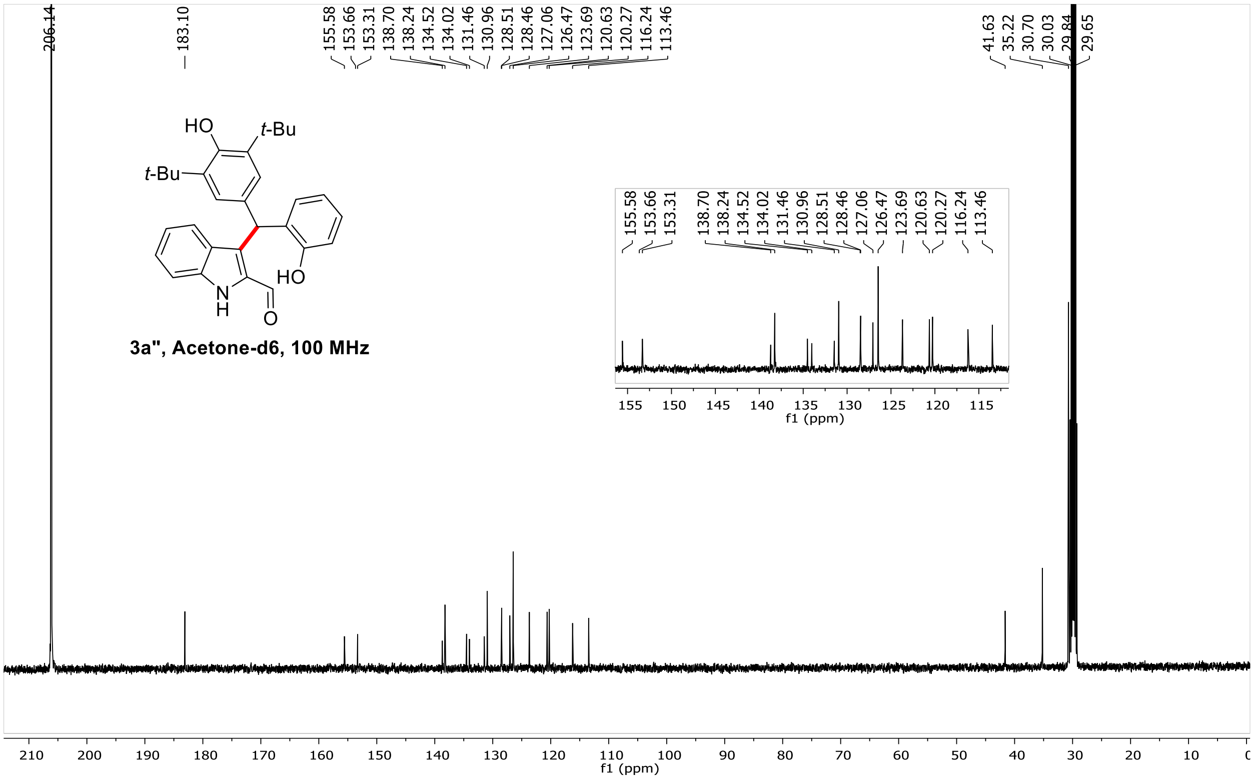

Phenyl-3-((4-bromophenyl)(3,5-di-tert-butyl-4-hydroxyphenyl)methyl)-1*H*-indole-2-carboxylate (10)

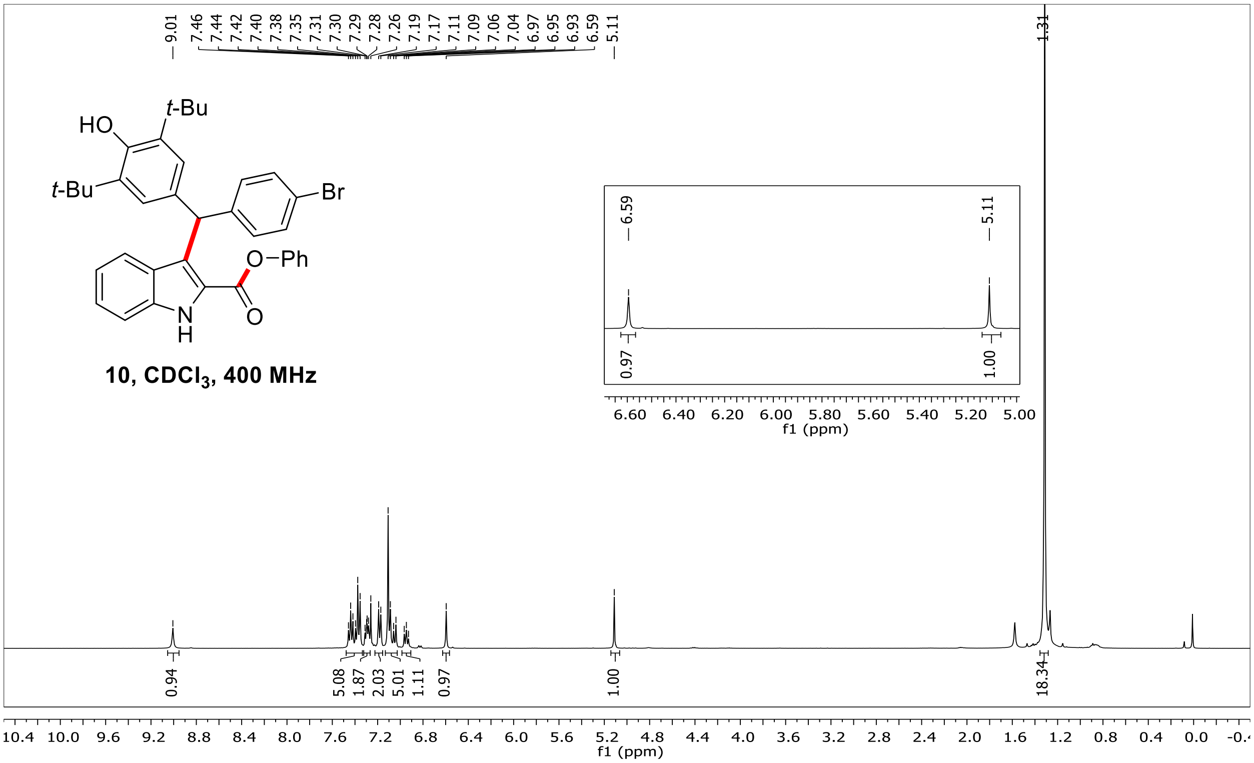

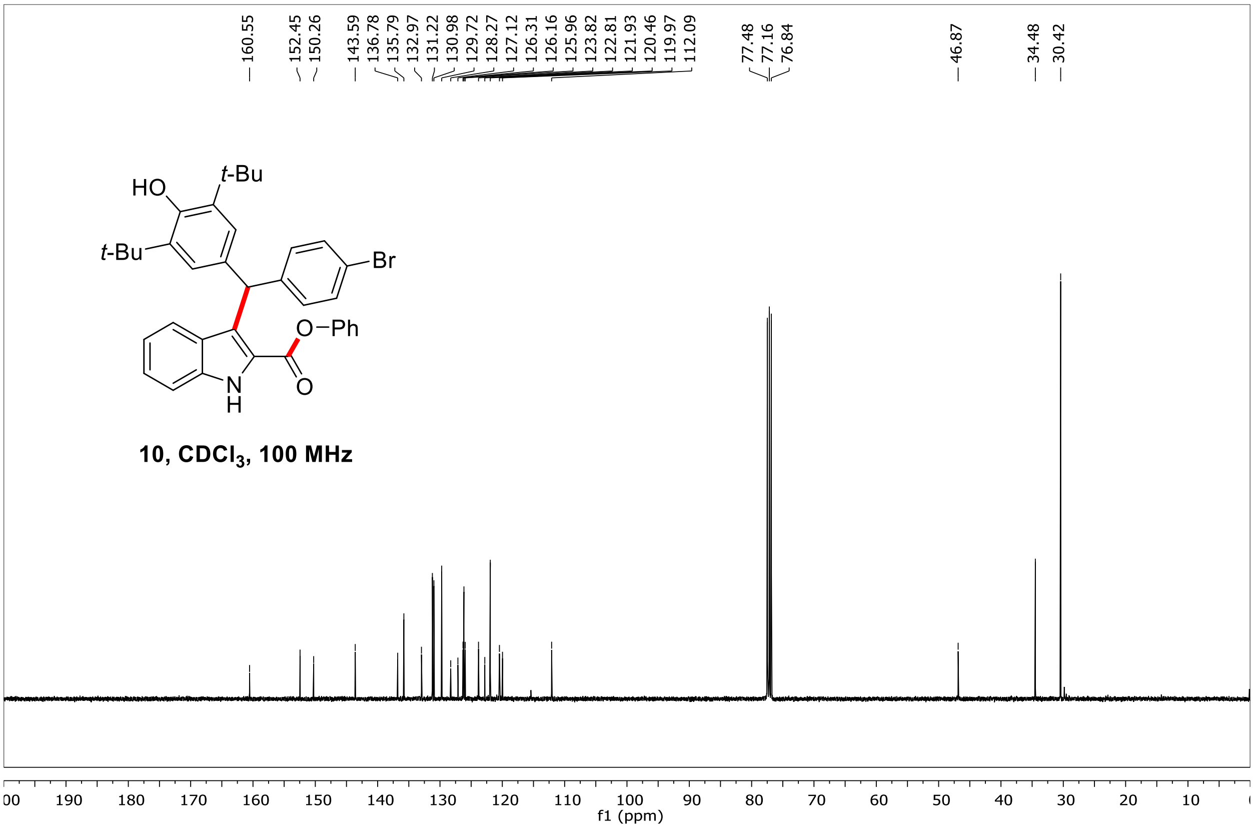

9. HPLC Data of Tetracyclic ε-Lactones

(*S*)-12-(3,5-Di-*tert*-butyl-4-hydroxyphenyl)-7,12-dihydro-6*H*-benzo[6,7]oxepino[3,4-*b*] indol-6-one (*chiral*-3a)

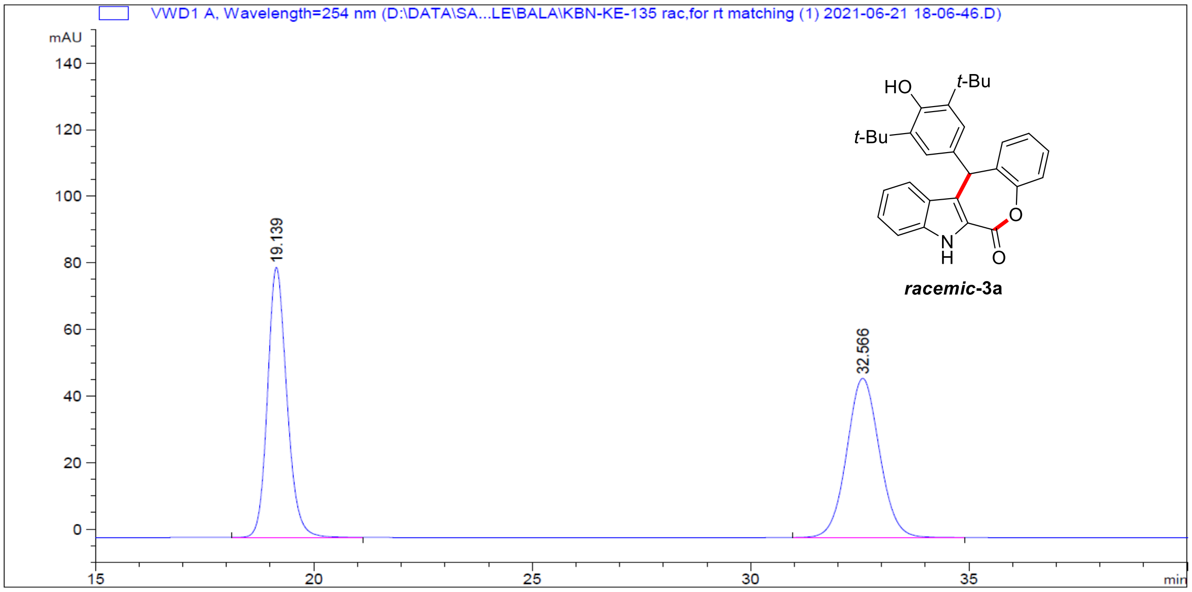

Peak #	RetTime [min]	Type	Width [min]	Area [mAU*s]	Height [mAU]	Area %
1	19.139	BB	0.4718	2500.86304	81.08739	50.0304
2	32.566	BB	0.8066	2497.81885	47.80070	49.9696

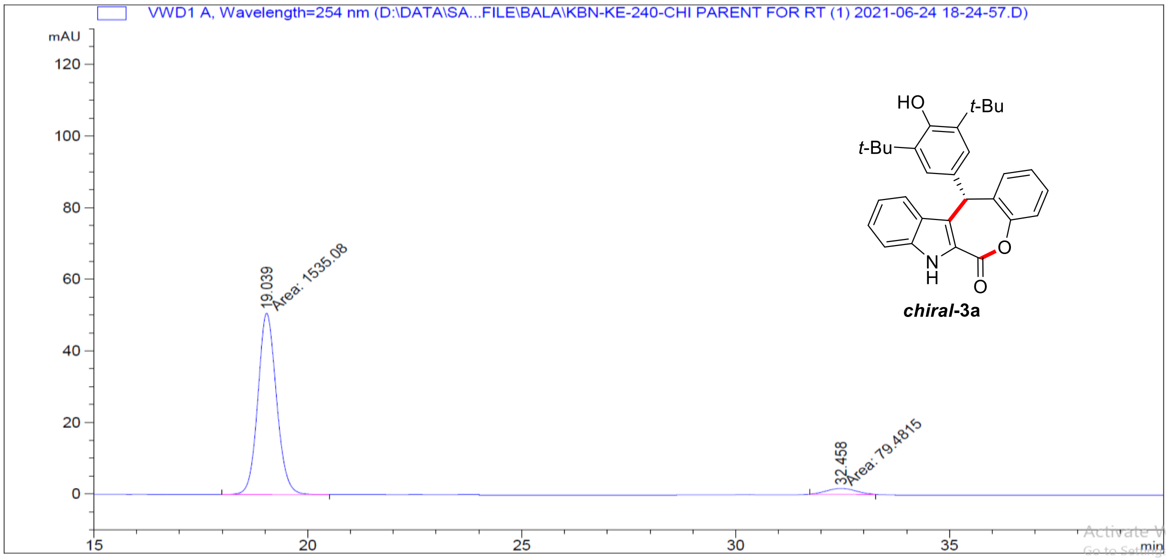

Peak #	RetTime [min]	Type	Width [min]	Area [mAU*s]	Height [mAU]	Area %
1	19.039	MM	0.5049	1535.07922	50.66981	94.6130
2	32.455	MM	0.8341	87.40266	1.74635	5.3870

Sample Info : CHIRALPAK IA, 10% IPA-HEXANE, 1.0 mL/min, 254 nm

(*S*)-12-(3,5-Di-*tert*-butyl-4-hydroxyphenyl)-11-fluoro-7,12-dihydro-6*H*-benzo[6,7]oxepino[3,4-*b*]indol-6-one (3b)

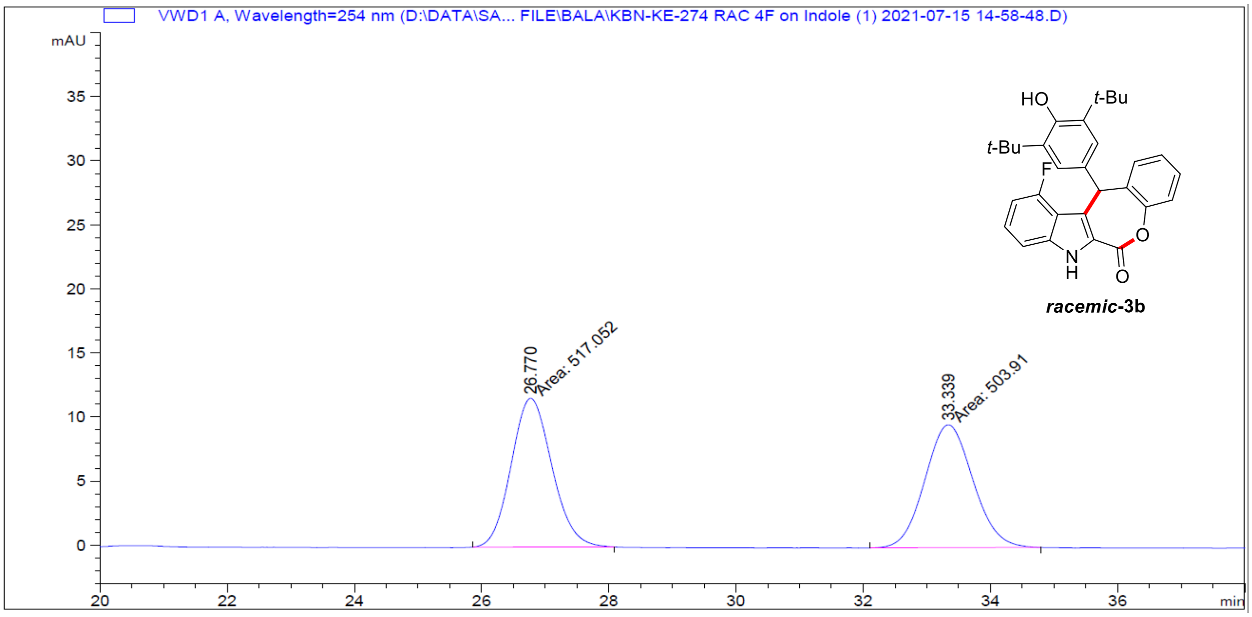

Peak #	RetTime [min]	Type	Width [min]	Area [mAU*s]	Height [mAU]	Area %
1	26.770	MM	0.7427	517.05225	11.60322	50.6436
2	33.339	MM	0.8759	503.91010	9.58895	49.3564

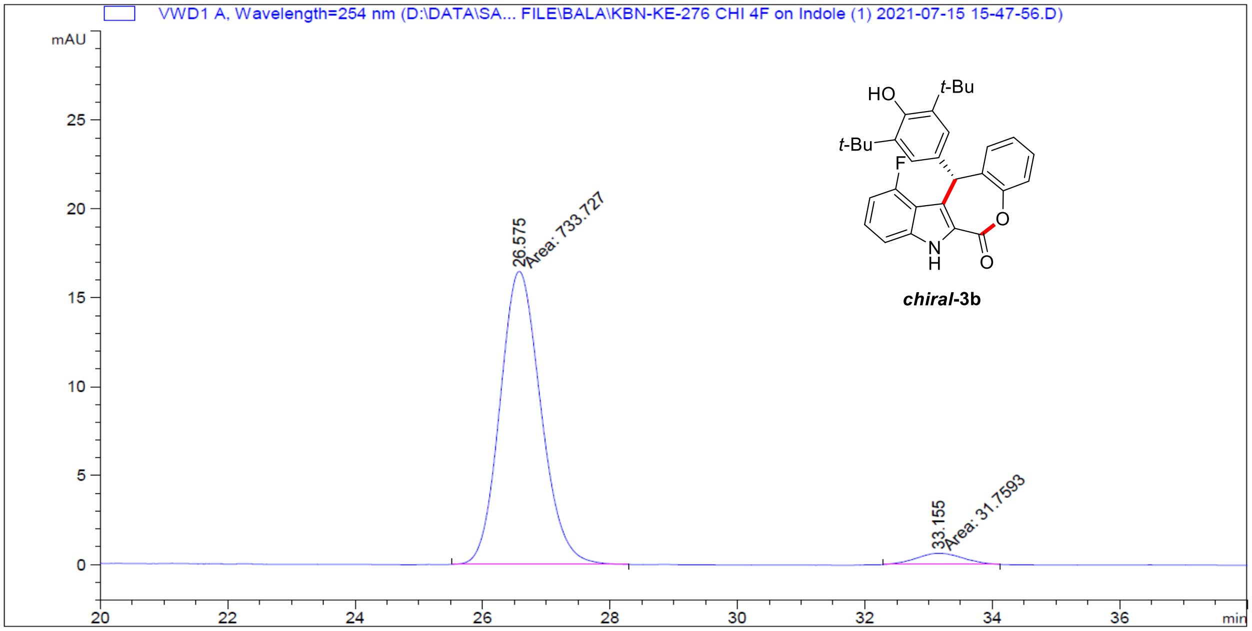

Peak #	RetTime [min]	Type	Width [min]	Area [mAU*s]	Height [mAU]	Area %
1	26.575	MM	0.7419	733.72742	16.48237	95.8511
2	33.155	MM	0.8507	31.75931	6.22197e-1	4.1489

Sample Info : CHIRALPAK IA, 10 % IPA-HEXANE, 1.0 mL/min, 254 nM.M

(*S*)-12-(3,5-Di-*tert*-butyl-4-hydroxyphenyl)-10-methoxy-7,12-dihydro-6*H*-benzo[6,7] oxepino[3, 4-b]indol-6-one (3c)

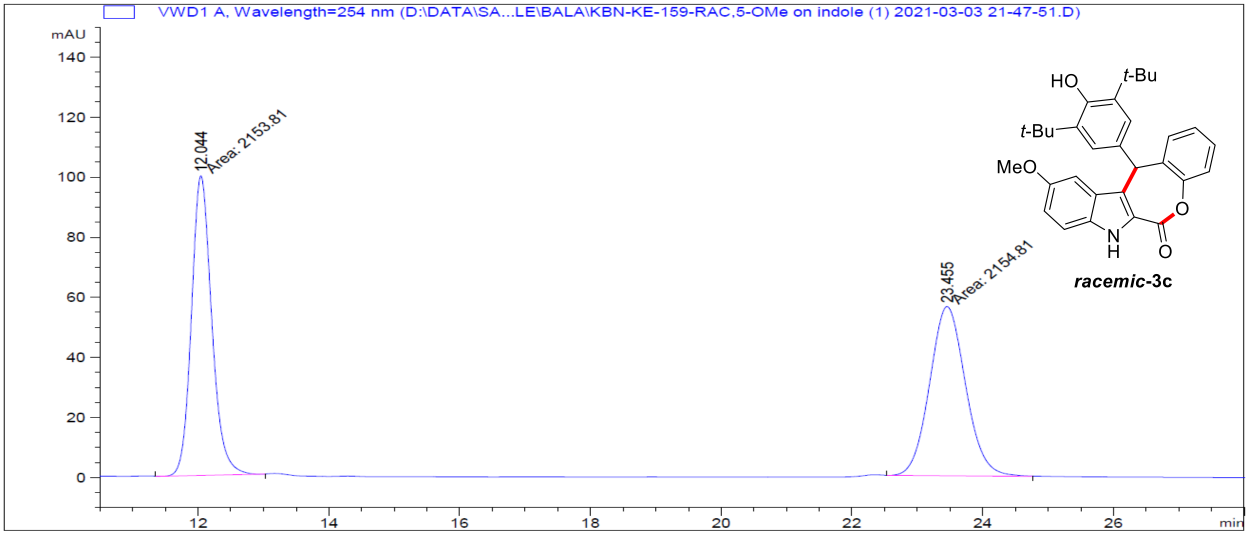

Peak #	RetTime [min]	Type	Width [min]	Area [mAU*s]	Height [mAU]	Area %
1	12.044	MM	0.3599	2153.81470	99.75196	49.9884
2	23.455	MM	0.6375	2154.81421	56.33720	50.0116

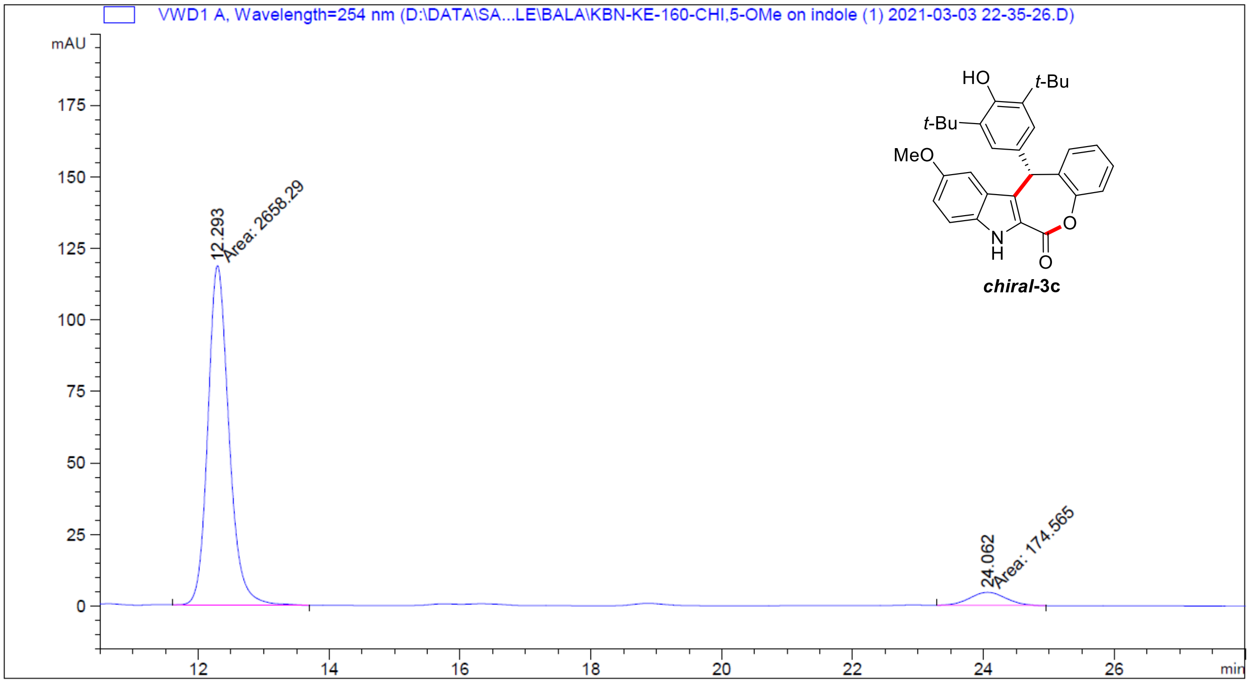

Peak #	RetTime [min]	Type	Width [min]	Area [mAU*s]	Height [mAU]	Area %
1	12.293	MM	0.3736	2658.28613	118.58536	93.8378
2	24.062	MM	0.6359	174.56494	4.57559	6.1622

Sample Info : CHIRALPAK-IA, 10% IPA-HEXANE, 1.0 mL/min, 254 nm

(*S*)-12-(3,5-Di-*tert*-butyl-4-hydroxyphenyl)-10-methyl-7,12-dihydro-6*H*benzo [6,7]oxepino[3,4-b]indol-6-one (3d)

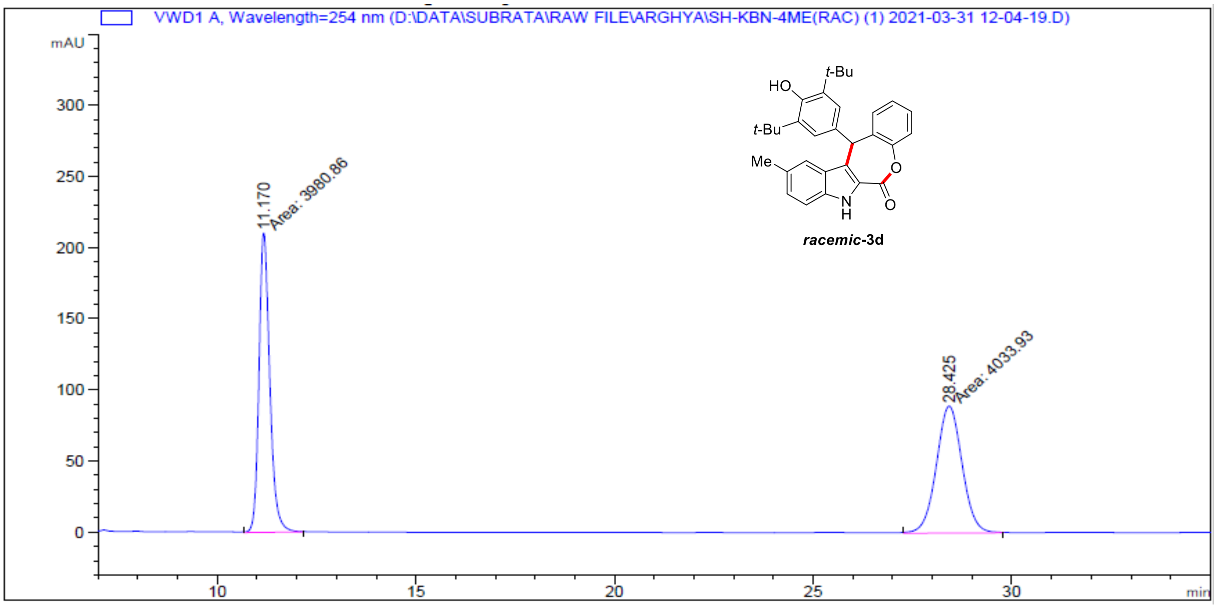

Peak #	RetTime [min]	Type	Width [min]	Area [mAU*s]	Height [mAU]	Area %
1	11.170	MM	0.3154	3980.85620	210.38341	49.6689
2	28.425	MM	0.7531	4033.93091	89.27859	50.3311

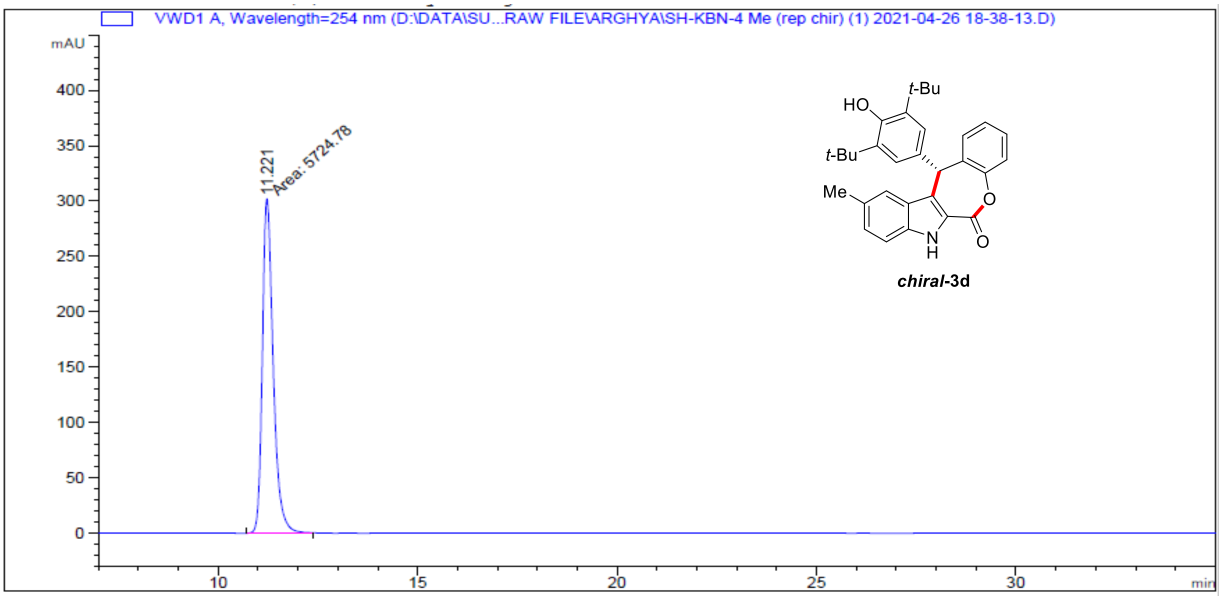

Peak #	RetTime [min]	Type	Width [min]	Area [mAU*s]	Height [mAU]	Area %
1	11.221	MM	0.3157	5724.78125	302.23056	100.0000

Sample Info : CHIRALPAK IA, 10% IPA-HEXANE,1.0 mL/min, 254 nm

S)-12-(3,5-di-*Tert*-butyl-4-hydroxyphenyl)-10-ethyl-7,12-dihydro-6*H*-benzo[6,7]oxepino[3,4-*b*]indol-6-one (3e)

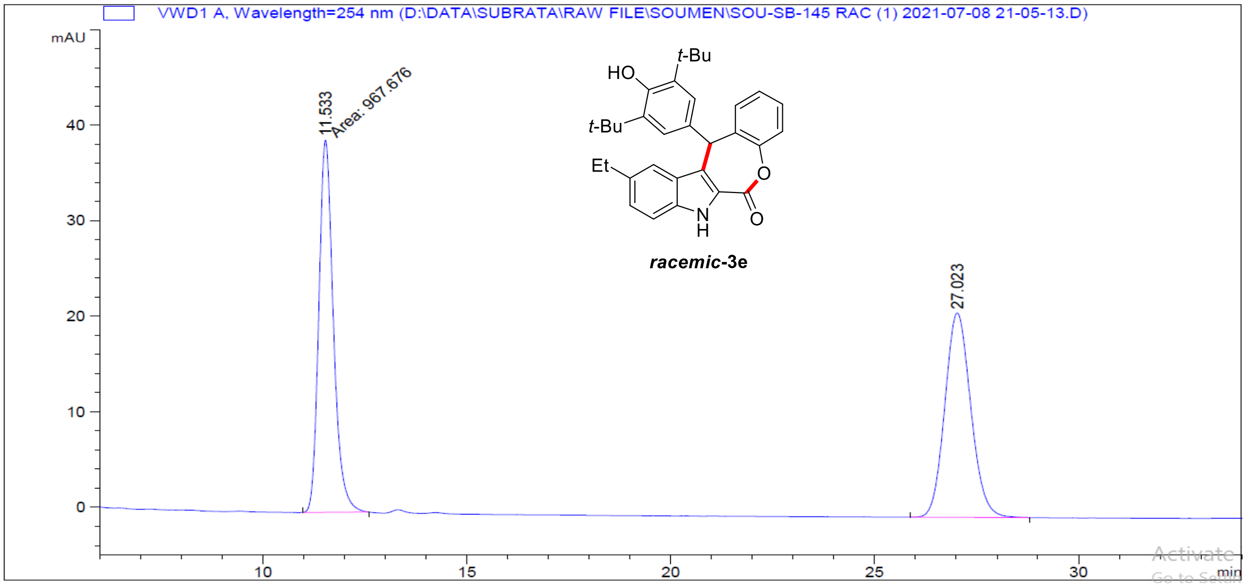

Peak #	RetTime [min]	Type	Width [min]	Area [mAU*s]	Height [mAU]	Area %
1	11.533	MM	0.4143	967.67603	38.92627	50.5375
2	27.023	BB	0.6858	947.09058	21.38240	49.4625

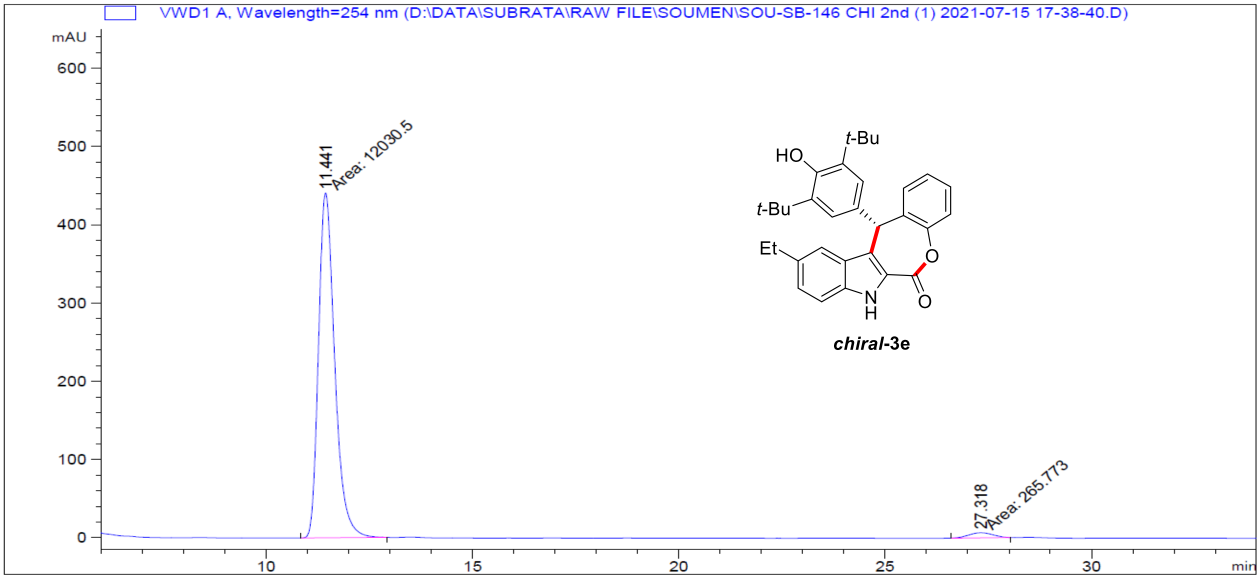

Peak #	RetTime [min]	Type	Width [min]	Area [mAU*s]	Height [mAU]	Area %
1	11.441	MM	0.4551	1.20305e4	440.59888	97.8386
2	27.318	MM	0.6743	265.77267	6.56903	2.1614

Sample Info : CHIRALPAK IA, 10% IPA-HEXANE, 1.0 mL/min, 254 nm

(*S*)-10-(*Tert*-butyl)-12-(3,5-di-*tert*-butyl-4-hydroxyphenyl)-7,12-dihydro-6*H* benzo[6,7]oxepino[3,4-b]indol-6-one (3f)

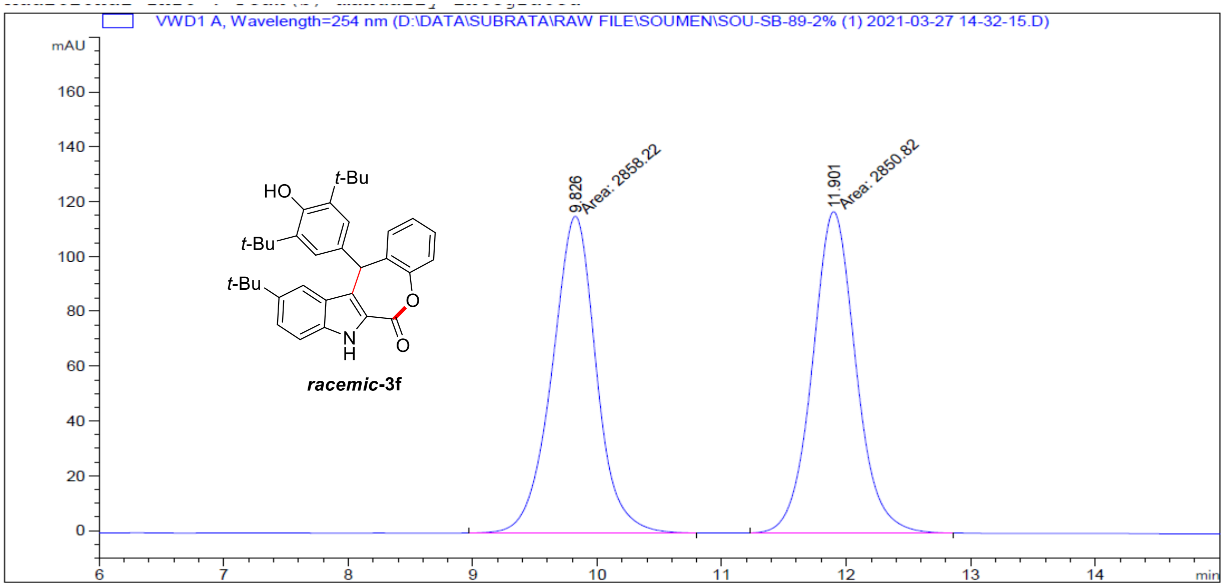

Peak #	RetTime [min]	Type	Width [min]	Area [mAU*s]	Height [mAU]	Area %
1	9.622	MM	0.5192	227.34413	7.29850	4.9055
2	11.633	MM	0.5209	4407.13135	141.01996	95.0945

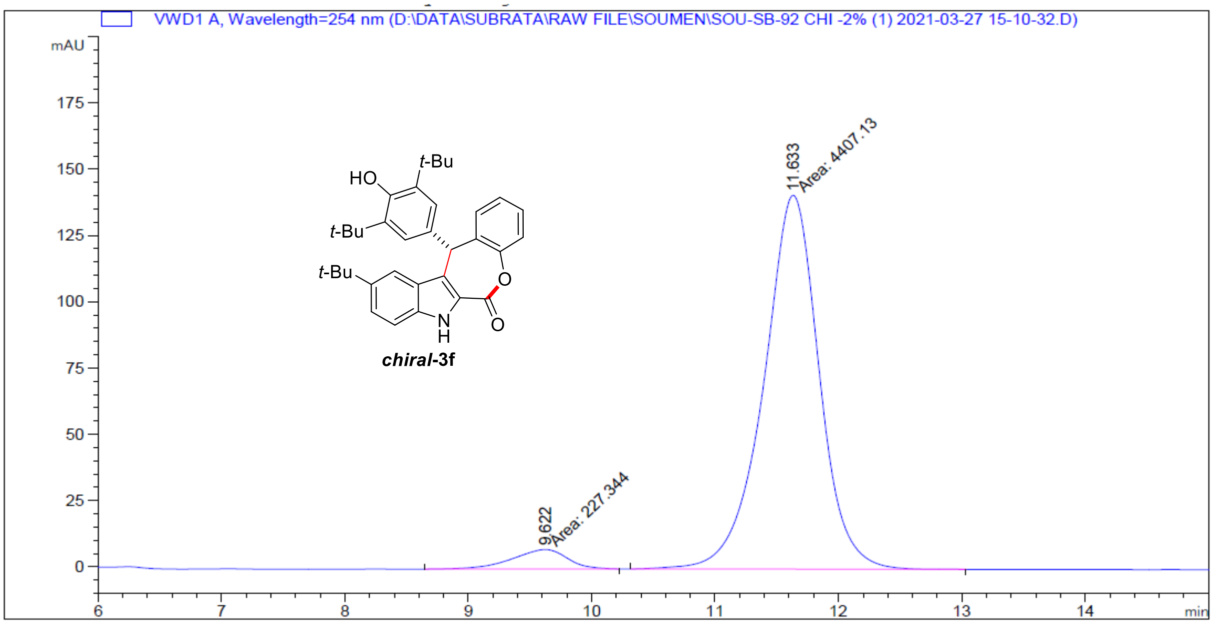

Peak #	RetTime [min]	Type	Width [min]	Area [mAU*s]	Height [mAU]	Area %
1	9.622	MM	0.5192	227.34413	7.29850	4.9055
2	11.633	MM	0.5209	4407.13135	141.01996	95.0945

Sample Info : CHIRALPAK-IA, 2 % IPA-HEXANE, 1.0 mL/min, 254 nm

(*S*)-12-(3,5-Di-*tert*-butyl-4-hydroxyphenyl)-10-(trifluoromethoxy)-7,12-dihydro-6*H*-benzo[6,7]oxepino[3,4-*b*]indol-6-one (3g)

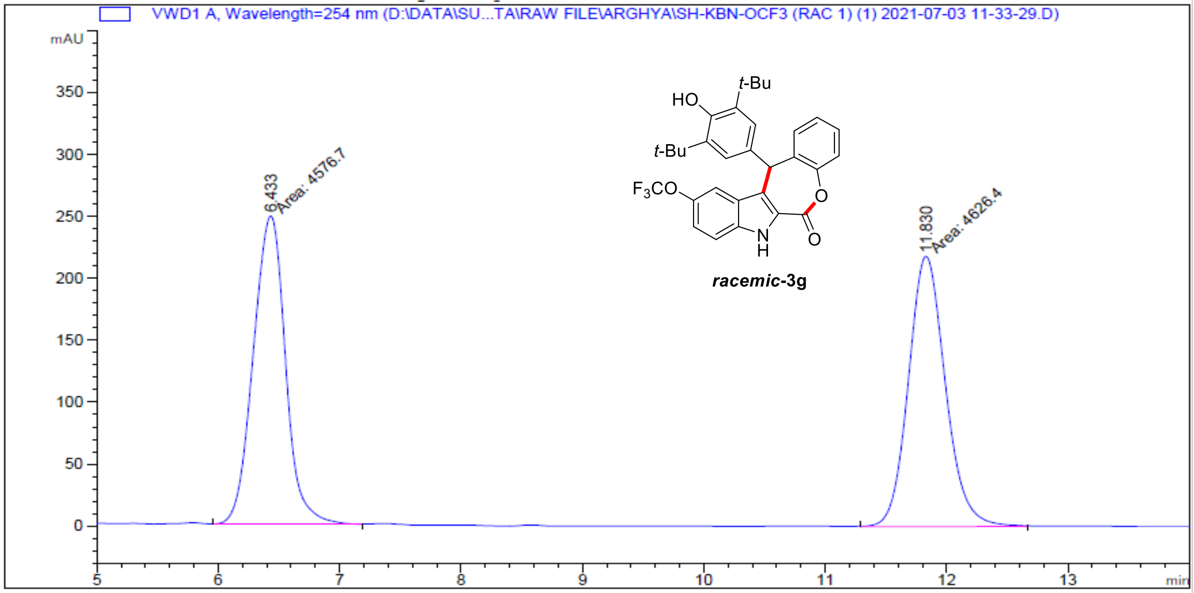

Peak #	RetTime [min]	Type	Width [min]	Area [mAU*s]	Height [mAU]	Area %
1	6.433	MM	0.3069	4576.70166	248.50728	49.7300
2	11.830	MM	0.3534	4626.39746	218.18427	50.2700

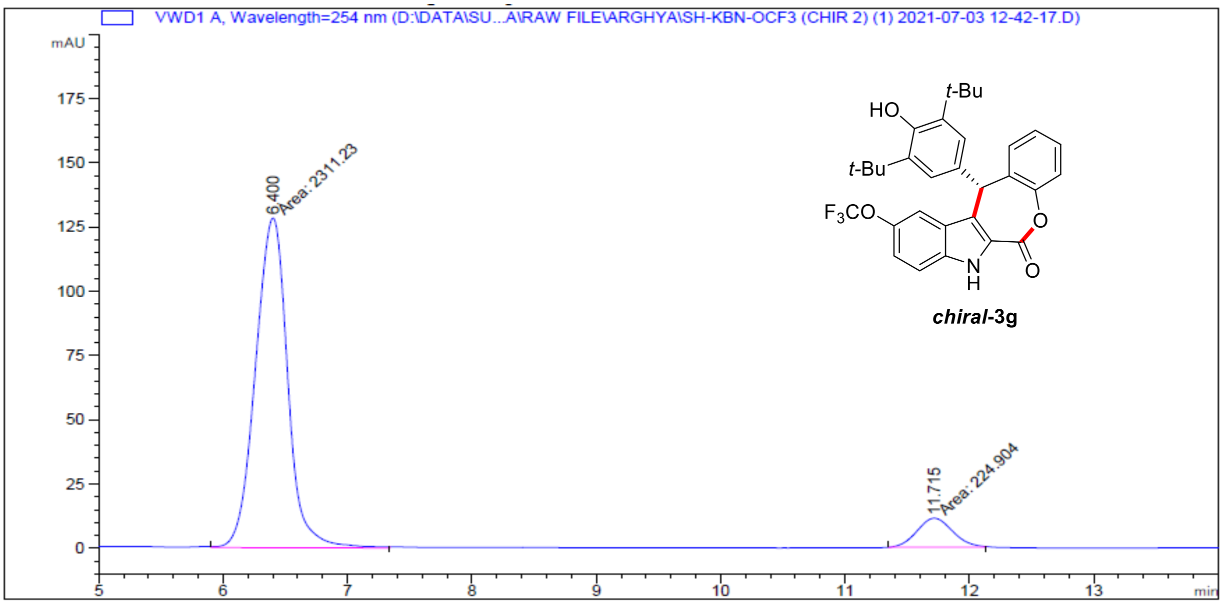

Peak #	RetTime [min]	Type	Width [min]	Area [mAU*s]	Height [mAU]	Area %
1	6.400	MM	0.3002	2311.22803	128.31041	91.1320
2	11.715	MM	0.3318	224.90450	11.29724	8.8680

Sample Info : CHIRALPAK IA, 10% IPA-HEXANE, 1.0 mL/min, 254 nm

(*S*)-10-Bromo-12-(3,5-di-*tert*-butyl-4-hydroxyphenyl)-7,12-dihydro-6*H*-benzo[6,7]oxepino[3,4-*b*]indol-6-one (3h)

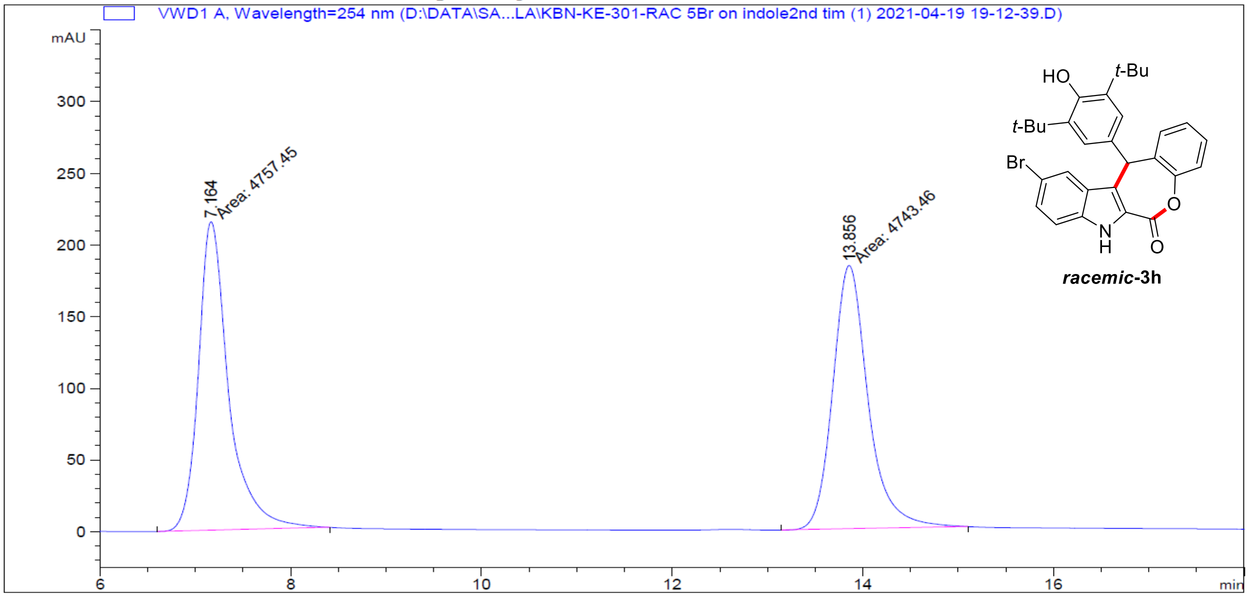

Peak #	RetTime [min]	Type	Width [min]	Area [mAU*s]	Height [mAU]	Area %
1	7.164	MM	0.3685	4757.44629	215.16971	50.0736
2	13.856	MM	0.4304	4743.46094	183.66583	49.9264

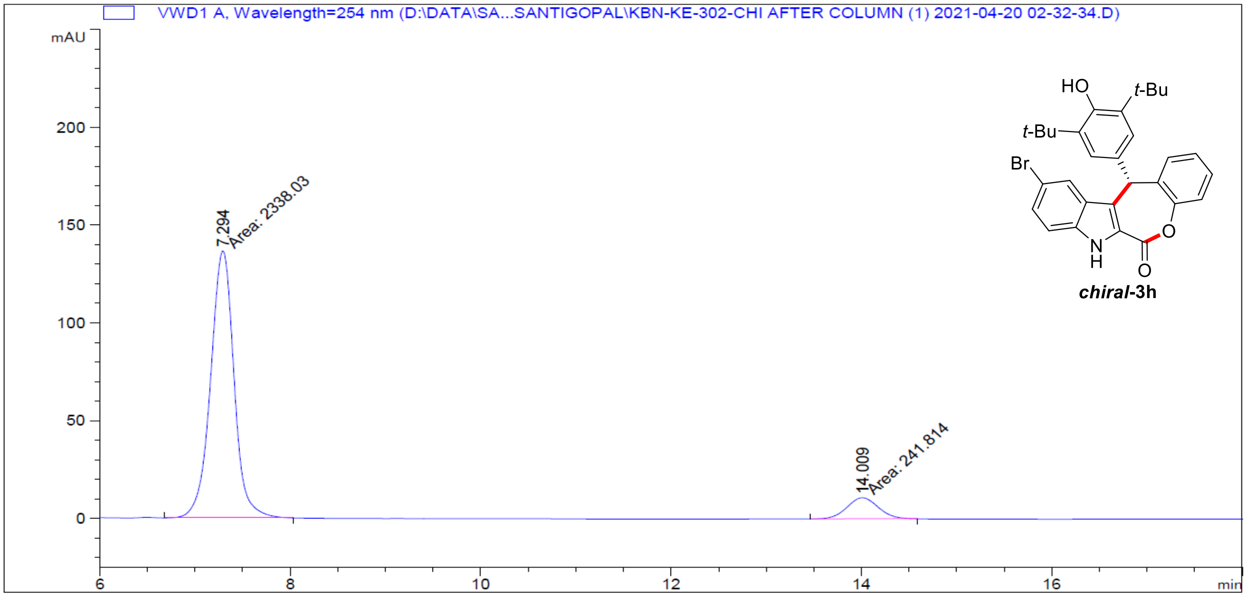

Peak #	RetTime [min]	Type	Width [min]	Area [mAU*s]	Height [mAU]	Area %
1	7.294	MM	0.2855	2338.03467	136.48889	90.6268
2	14.009	MM	0.3761	241.81406	10.71552	9.3732

Sample Info : CHIRALPAK-IA, 10% IPA-HEXANE, 1.0 mL/min, 254 nm

(*S*)-10-Chloro-12-(3,5-di-*tert*-butyl-4-hydroxyphenyl)-7,12-dihydro-6*H*-benzo[6,7]oxepino[3,4-*b*]indol-6-one (3i)

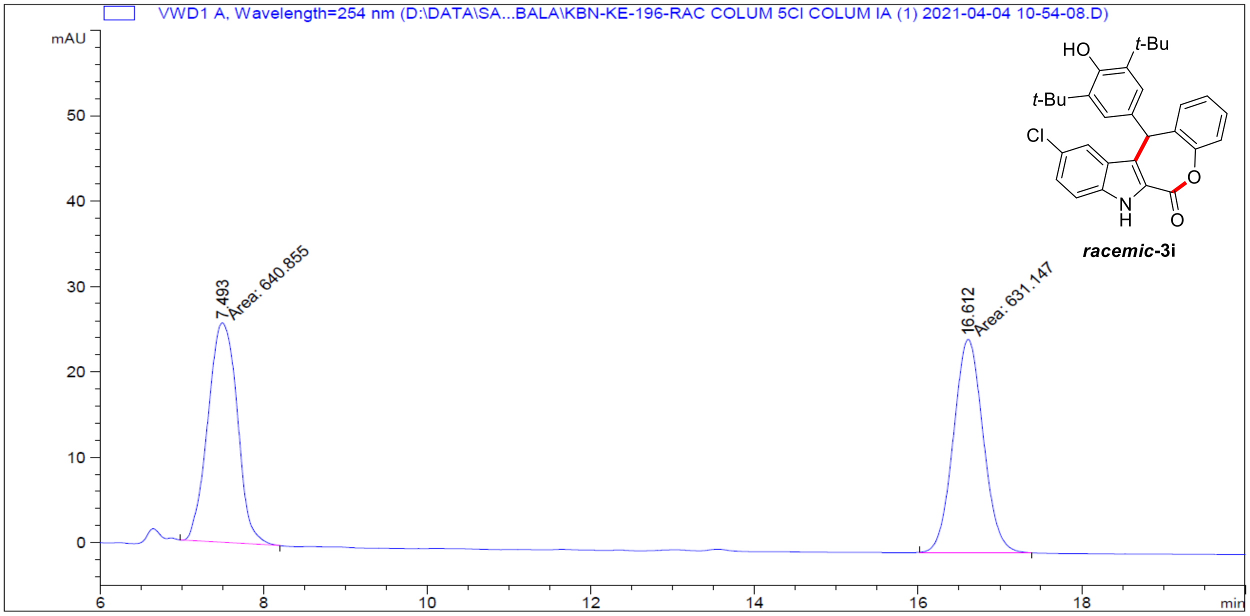

Peak #	RetTime [min]	Type	Width [min]	Area [mAU*s]	Height [mAU]	Area %
1	7.493	MM	0.4157	640.85535	25.69365	50.3816
2	16.612	MM	0.4212	631.14740	24.97251	49.6184

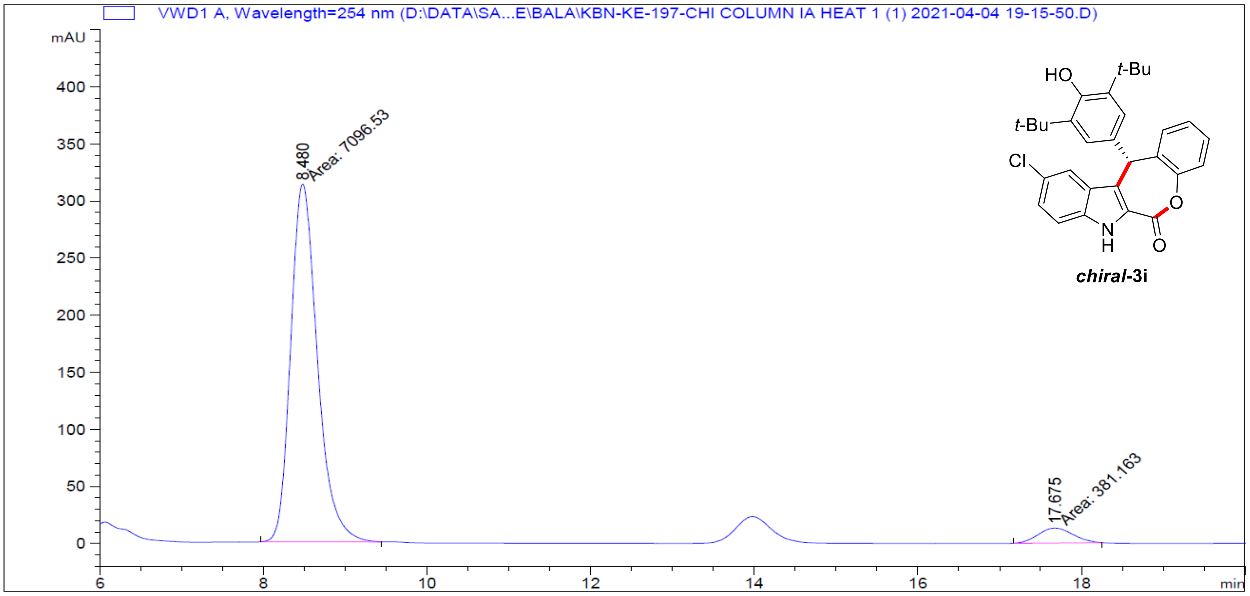

Peak #	RetTime [min]	Type	Width [min]	Area [mAU*s]	Height [mAU]	Area %
1	8.480	MM	0.3779	7096.53027	312.97079	94.9027
2	17.675	MM	0.4874	381.16318	13.03368	5.0973

Sample Info : CHIRALPAK-IA, 10% IPA-HEXANE, 1.0 mL/min, 254 nm

(*S*)-12-(3,5-Di-*tert*-butyl-4-hydroxyphenyl)-10-fluoro-7,12-dihydro-6*H*-benzo[6,7]oxepino[3,4-b]indol-6-one (3j)

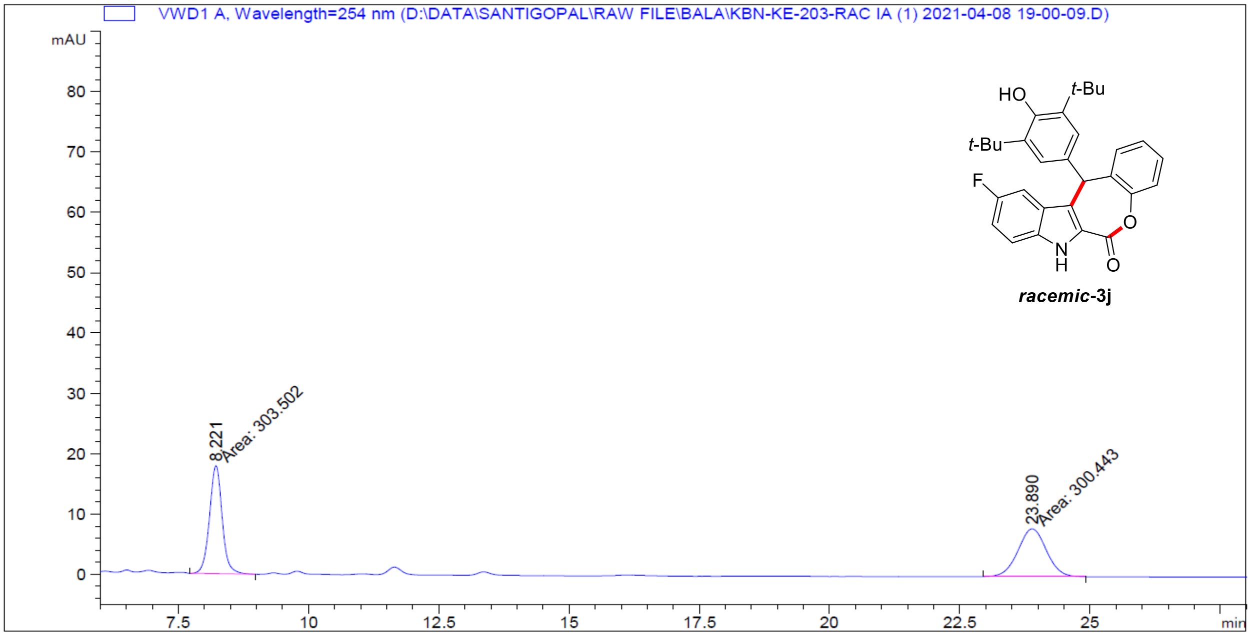

Peak #	RetTime [min]	Type	Width [min]	Area [mAU*s]	Height [mAU]	Area %
1	8.221	MM	0.2826	303.50235	17.89635	50.2533
2	23.890	MM	0.6332	300.44290	7.90804	49.7467

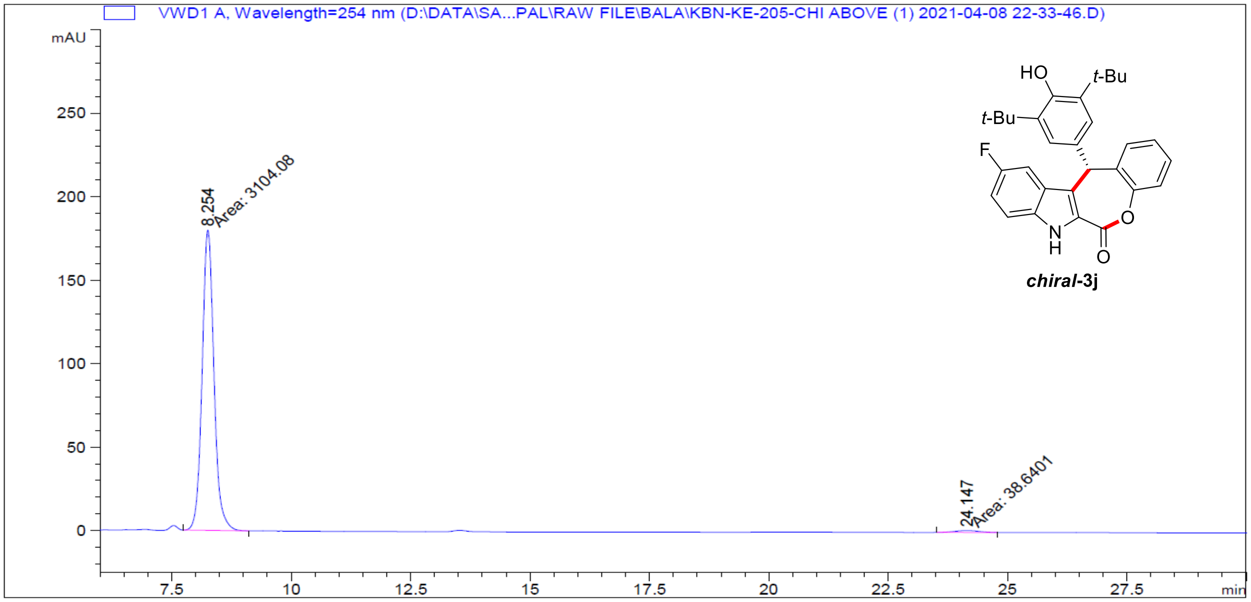

Peak #	RetTime [min]	Type	Width [min]	Area [mAU*s]	Height [mAU]	Area %
1	8.254	MM	0.2876	3104.07861	179.85658	98.7705
2	24.147	MM	0.5979	38.64014	1.07716	1.2295

Sample Info : CHIRALPAK- IA, 10% IPA-HEXANE, 1.0 mL/min, 254 nm

(*S*)-12-(3,5-di-*Tert*-butyl-4-hydroxyphenyl)-9-methoxy-7,12-dihydro-6*H* benzo[6,7]oxepino[3,4-b]indol-6-one(3k)

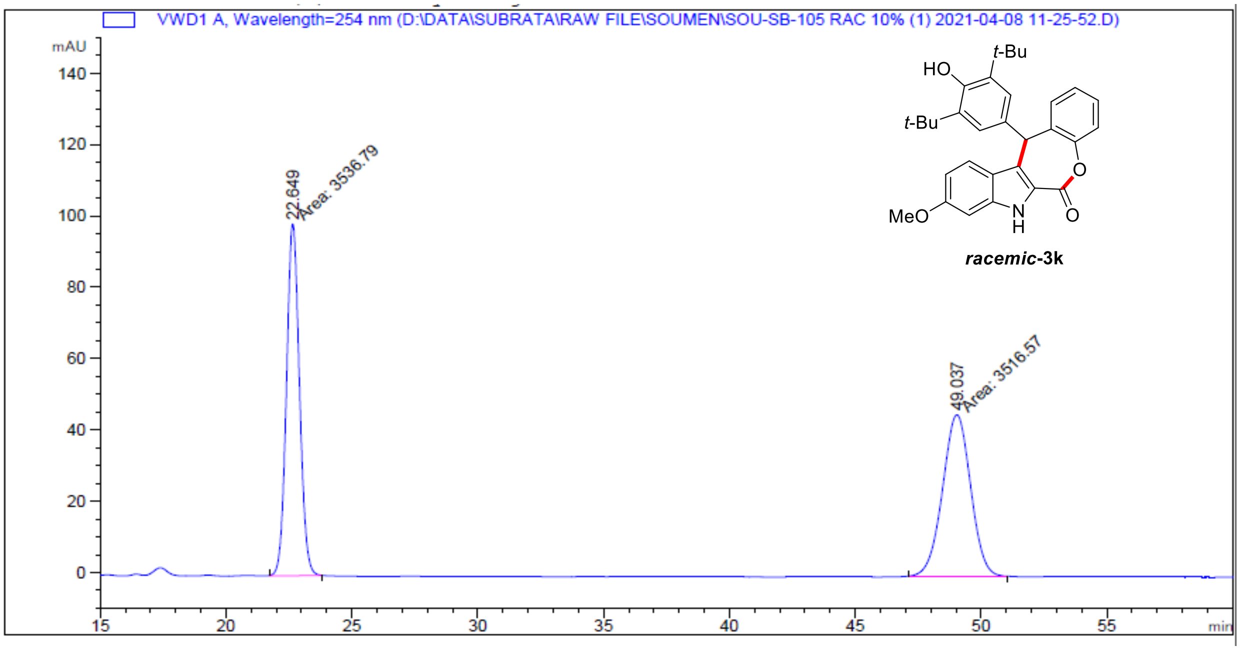

Peak #	RetTime [min]	Type	Width [min]	Area [mAU*s]	Height [mAU]	Area %
1	22.649	MM	0.5979	3536.78931	98.59322	50.1433
2	49.037	MM	1.2941	3516.57104	45.28960	49.8567

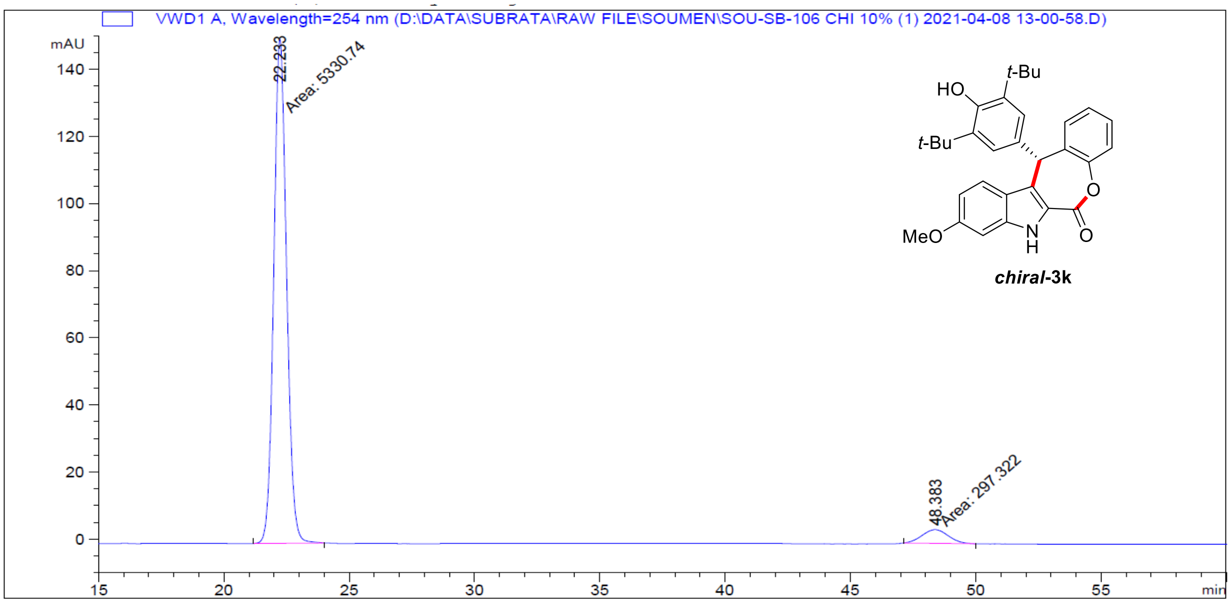

Peak #	RetTime [min]	Type	Width [min]	Area [mAU*s]	Height [mAU]	Area %
1	22.233	MM	0.5918	5330.74023	150.12422	94.7171
2	48.383	MM	1.2107	297.32248	4.09314	5.2829

Sample Info : CHIRALPAK IA, 10% IPA-HEXANE, 1.0 mL/min, 254 nm

(*S*)-12-(3,5-Di-*tert*-butyl-4-hydroxyphenyl)-9-methyl-7,12-dihydro-6*H*-benzo[6,7]oxepino[3,4-*b*]indol-6-one (3l)

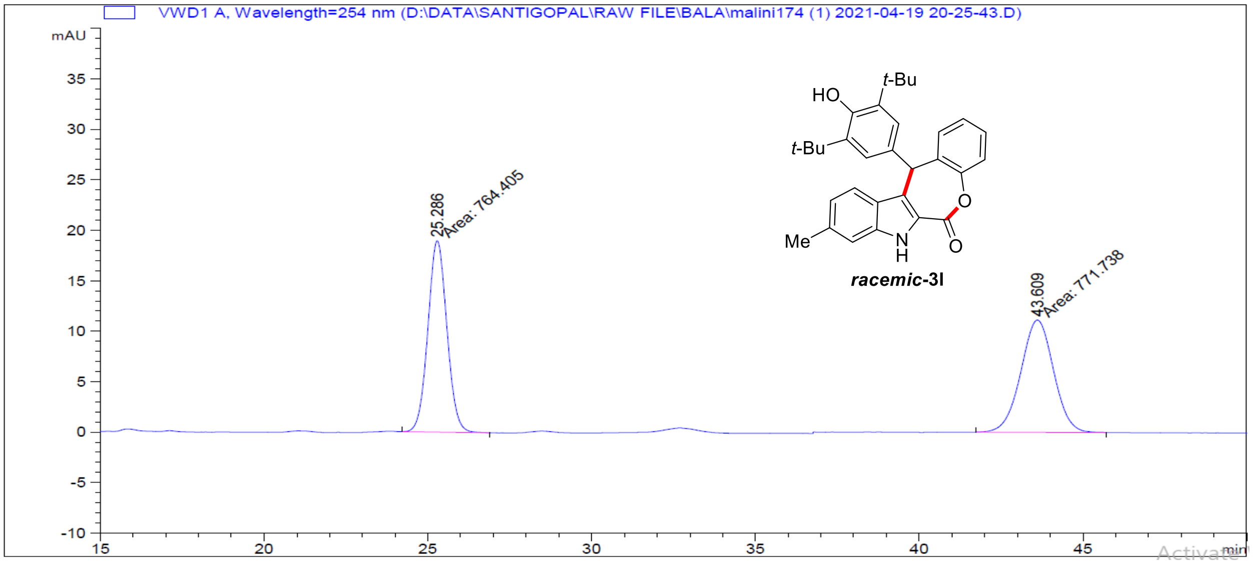

Peak #	RetTime [min]	Type	Width [min]	Area [mAU*s]	Height [mAU]	Area %
1	25.286	MM	0.6721	764.40466	18.95465	49.7613
2	43.609	MM	1.1570	771.73798	11.11731	50.2387

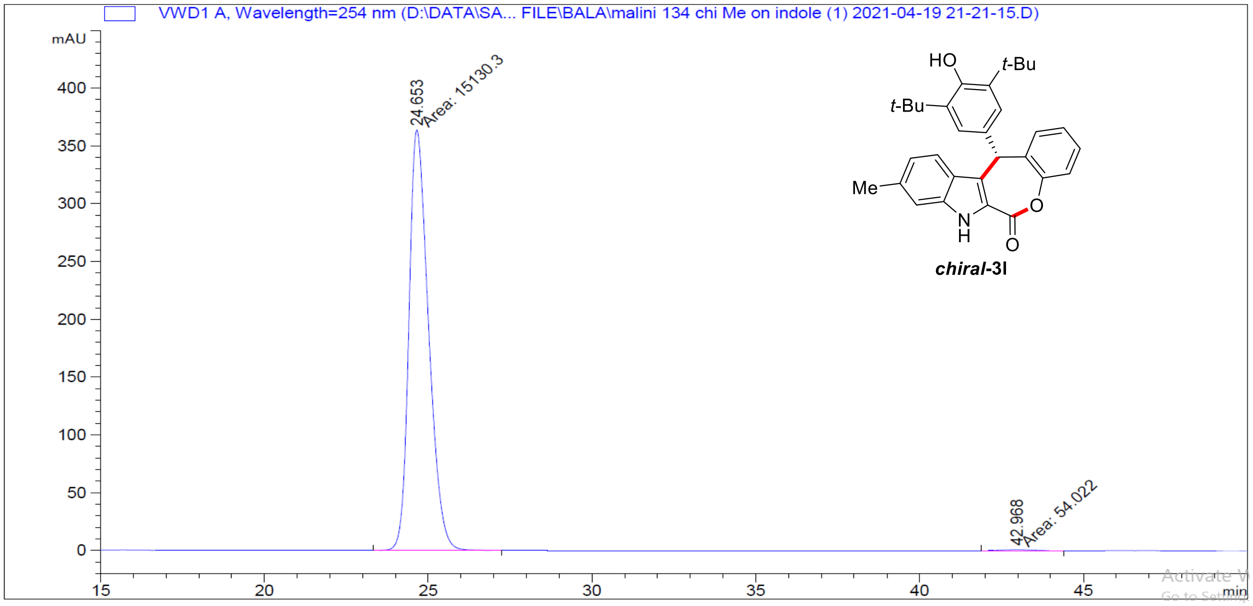

Peak #	RetTime [min]	Type	Width [min]	Area [mAU*s]	Height [mAU]	Area %
1	24.653	MM	0.6935	1.51303e4	363.61896	99.6442
2	42.968	MM	1.0951	54.02200	8.22173e-1	0.3558

Sample Info : CHIRALPAK-IA, 10% IPA-HEXANE, 1.0 mL/min, 254 nm

(*S*)-9-Bromo-12-(3,5-di-*tert*-butyl-4-hydroxyphenyl)-7,12-dihydro-6*H*-benzo[6,7]oxepino[3,4-*b*]indol-6-one (3m)

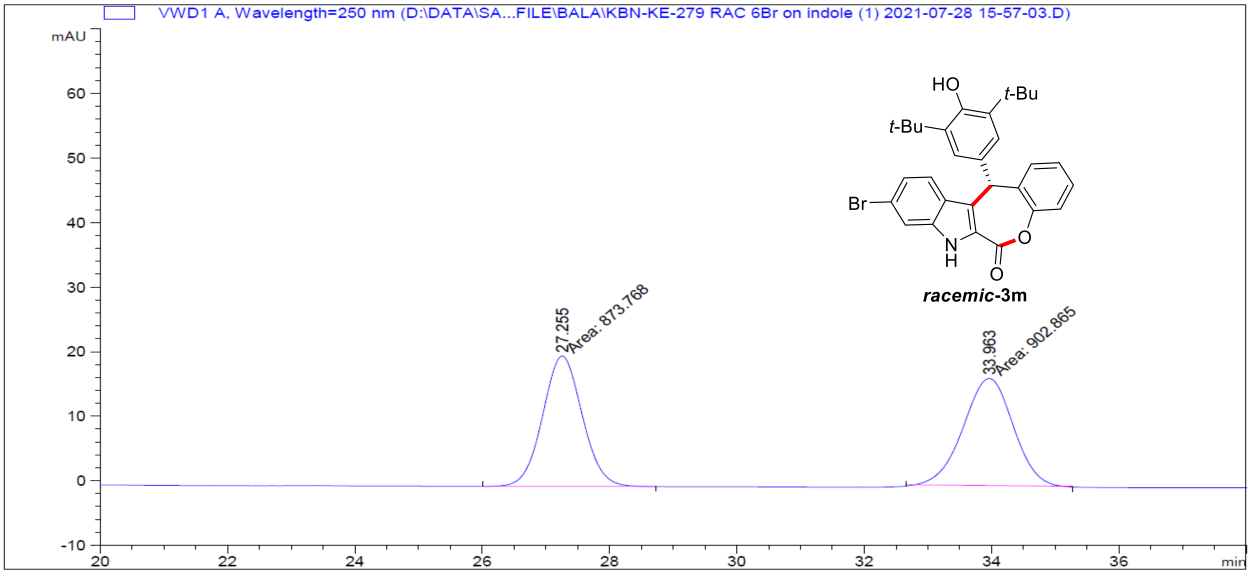

Peak #	RetTime [min]	Type	Width [min]	Area [mAU*s]	Height [mAU]	Area %
1	27.255	MM	0.7209	873.76837	20.20135	49.1811
2	33.963	MM	0.9076	902.86536	16.57979	50.8189

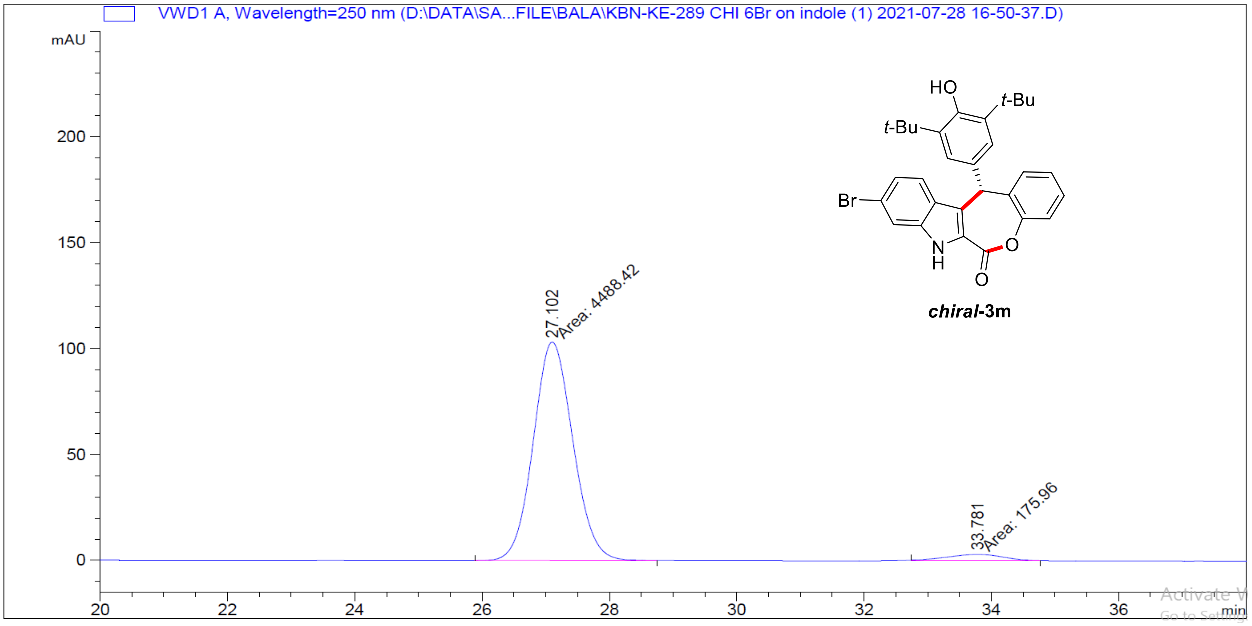

Peak #	RetTime [min]	Type	Width [min]	Area [mAU*s]	Height [mAU]	Area %
1	27.102	MM	0.7243	4488.42285	103.28620	96.2276
2	33.781	MM	0.9994	175.95998	2.93451	3.7724

Sample Info : CHIRALPAK IA, 10 % IPA-HEXANE, 1.0 mL/min, 254 nM.M

(*S*)-12-(3,5-Di-*tert*-butyl-4-hydroxyphenyl)-9-fluoro-7,12-dihydro-6*H*-benzo[6,7]oxepino[3,4-*b*]indol-6-one (3n)

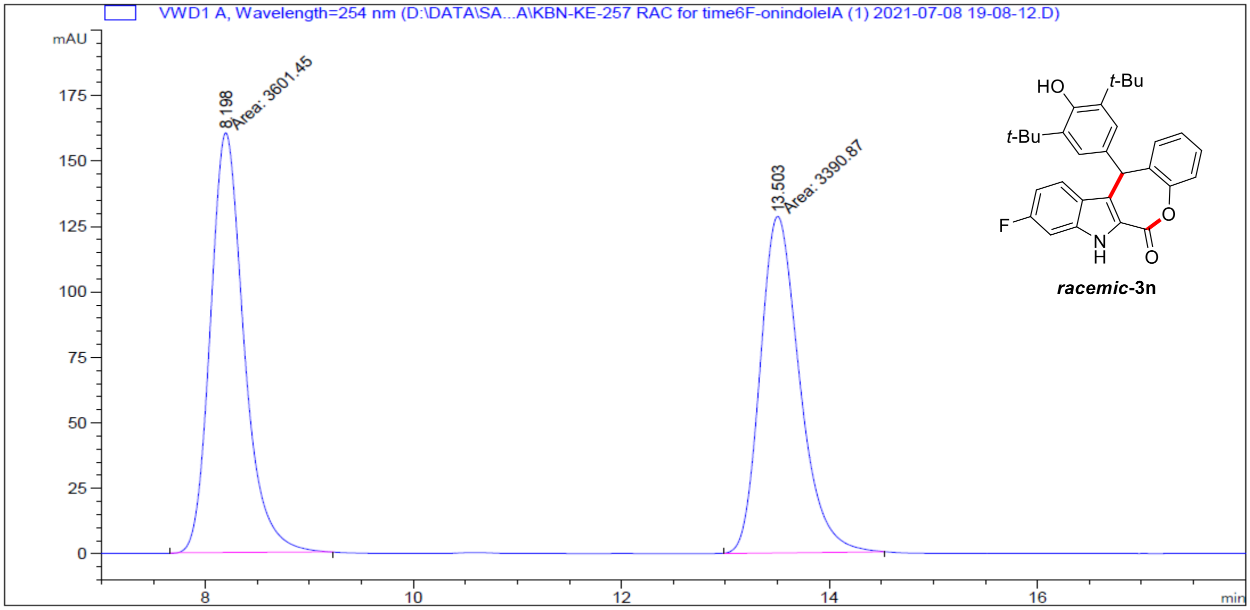

Peak #	RetTime [min]	Type	Width [min]	Area [mAU*s]	Height [mAU]	Area %
1	8.198	MM	0.3744	3601.45190	160.31020	51.5058
2	13.503	MM	0.4395	3390.87061	128.58704	48.4942

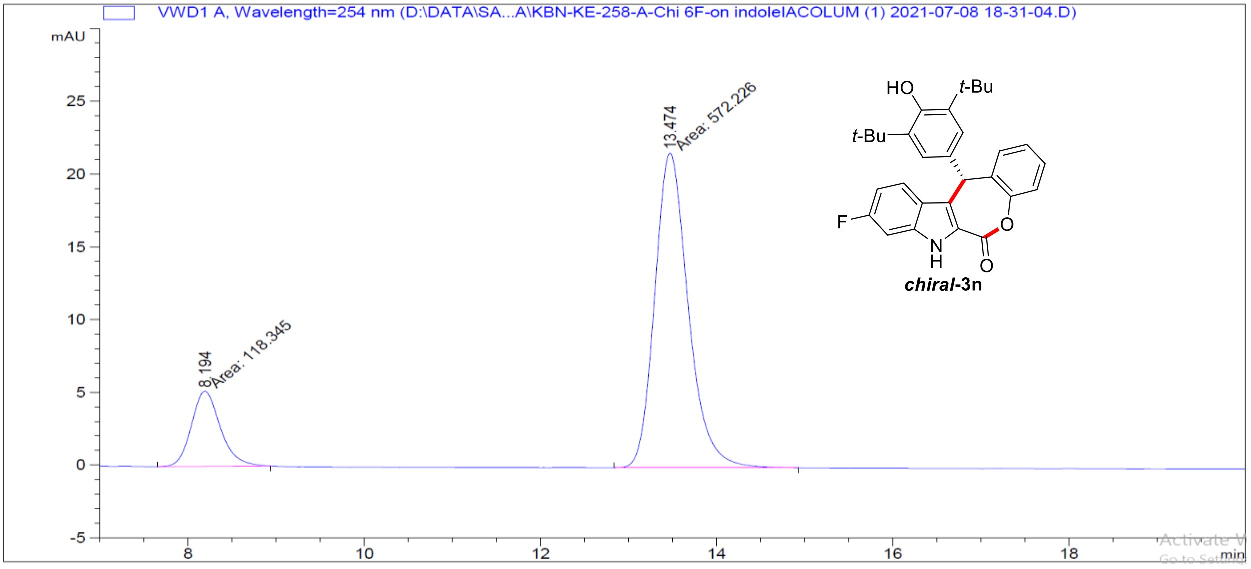

Peak #	RetTime [min]	Type	Width [min]	Area [mAU*s]	Height [mAU]	Area %
1	8.194	MM	0.3819	118.34513	5.16443	17.1373
2	13.474	MM	0.4413	572.22565	21.61150	82.8627

Sample Info : CHIRALPAK IA, 10 % IPA-HEXANE, 1.0 mL/min, 254 nM.M

(*S*)-12-(3,5-Di-*tert*-butyl-4-hydroxyphenyl)-9-phenyl-7,12-dihydro-6*H*-benzo[6,7]oxepino[3,4-*b*]indol-6-one (3o)

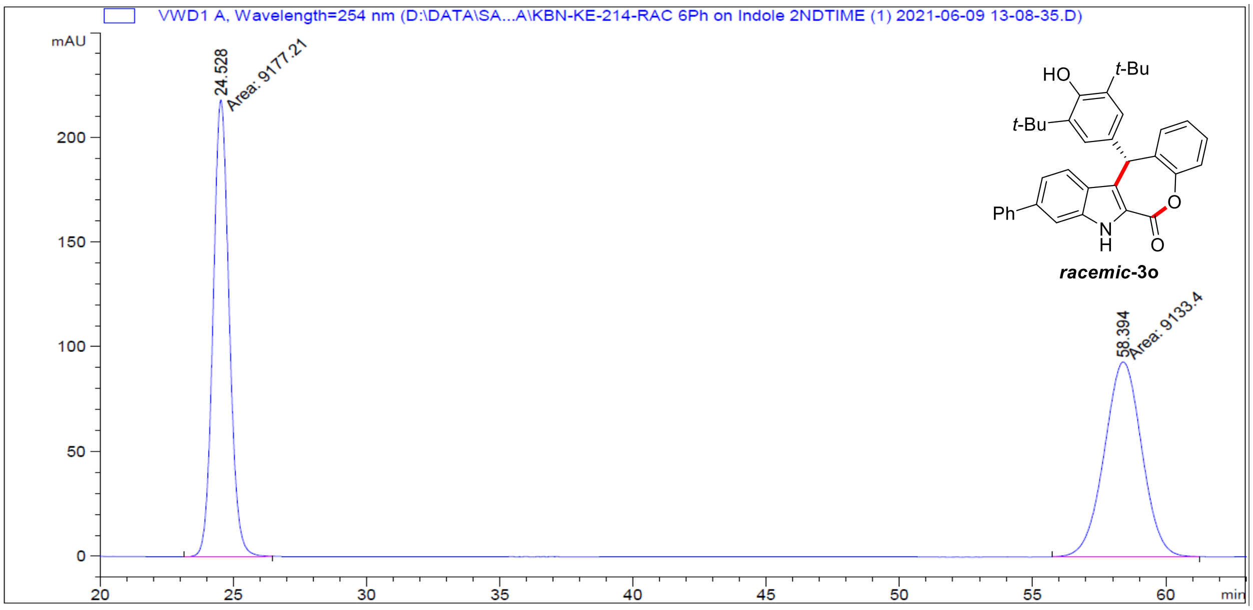

Peak #	RetTime [min]	Type	Width [min]	Area [mAU*s]	Height [mAU]	Area %
1	24.528	MM	0.7014	9177.21191	218.07961	50.1196
2	58.394	MM	1.6354	9133.39941	93.07738	49.8804

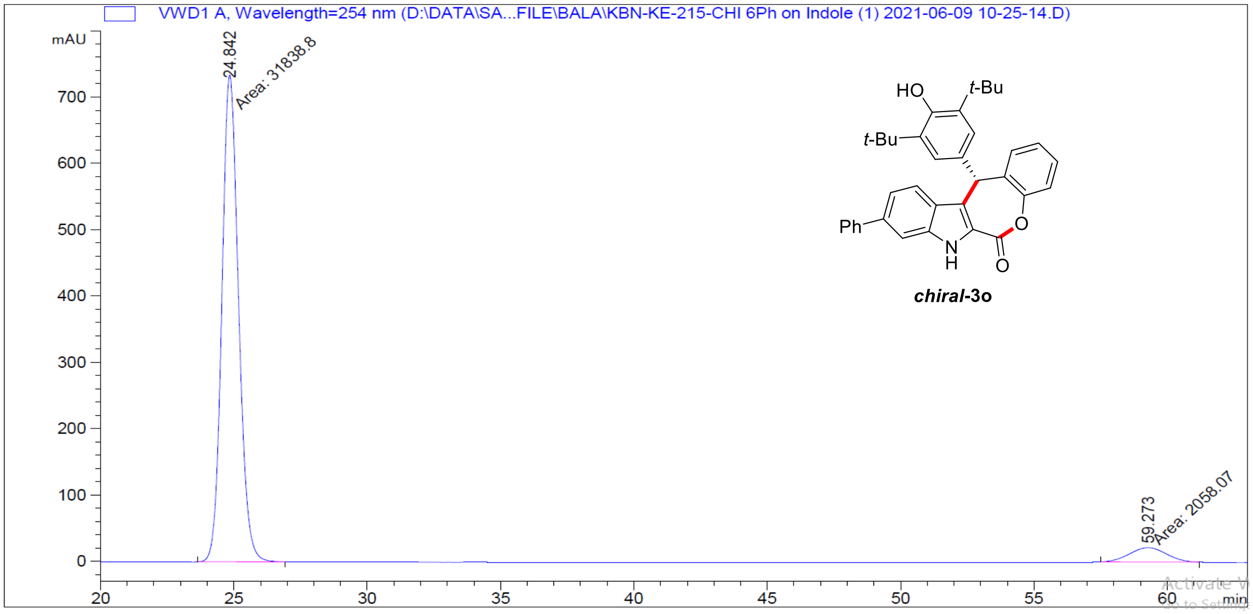

Peak #	RetTime [min]	Type	Width [min]	Area [mAU*s]	Height [mAU]	Area %
1	24.842	MM	0.7232	3.18388e4	733.76428	93.9284
2	59.273	MM	1.5853	2058.07251	21.63685	6.0716

Sample Info : CHIRALPAK IA, 10% IPA-HEXANE, 1.0 mL/min, 254 nm

(*S*)-12-(3,5-Di-*tert*-butyl-4-hydroxyphenyl)-9-(2-fluorophenyl)-7,12-dihydro-6*H*-benzo [6,7] oxepino[3,4-*b*] indol-6-one (3p)

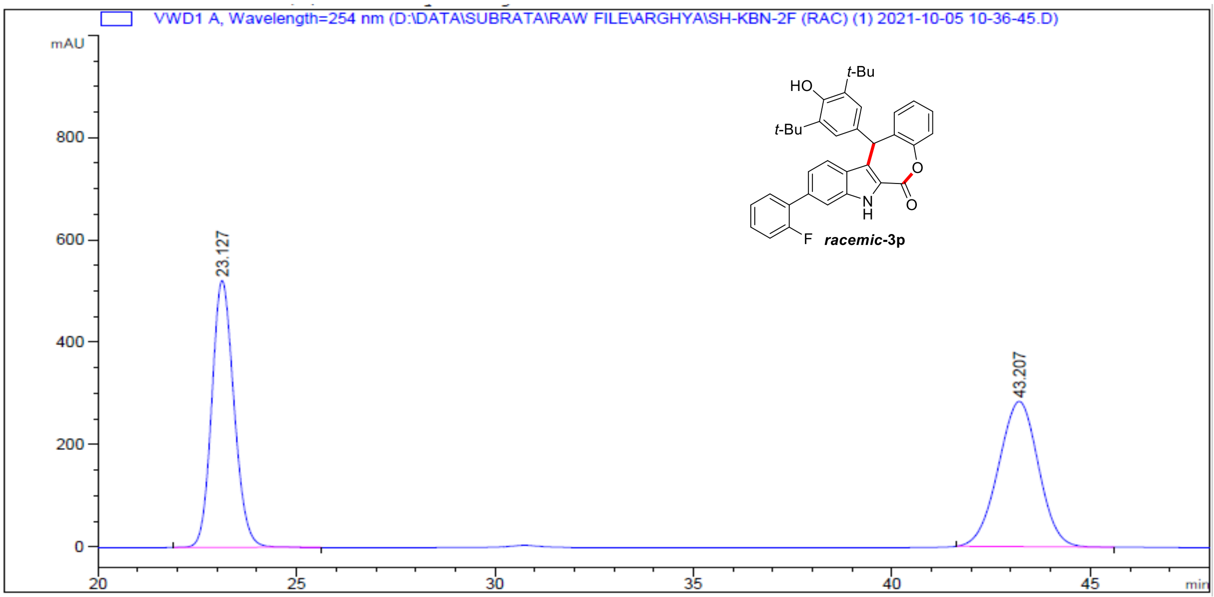

Peak #	RetTime [min]	Type	Width [min]	Area [mAU*s]	Height [mAU]	Area %
1	23.127	MM	0.6492	2.03000e4	521.15692	49.7931
2	43.207	MM	1.1927	2.04686e4	286.02219	50.2069

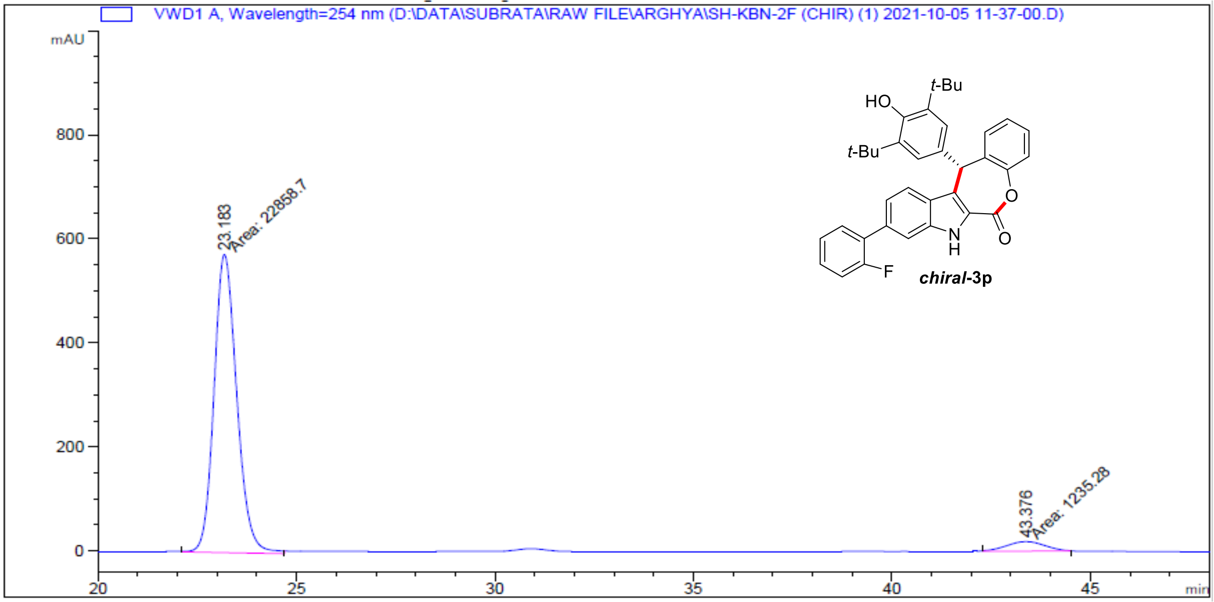

Peak #	RetTime [min]	Type	Width [min]	Area [mAU*s]	Height [mAU]	Area %
1	23.183	MM	0.6647	2.28587e4	573.17816	94.8731
2	43.376	MM	1.1191	1235.28381	18.39662	5.1269

Sample Info : CHIRALPAK IA, 10% IPA-HEXANE,1.0 mL/min, 254 nm

(*S*)-12-(3,5-di-*Tert*-butyl-4-hydroxyphenyl)-9-(2,6-dimethylphenyl)-7,12-dihydro-6*H*-benzo[6,7]oxepino[3,4-*b*]indol-6-one (3q)

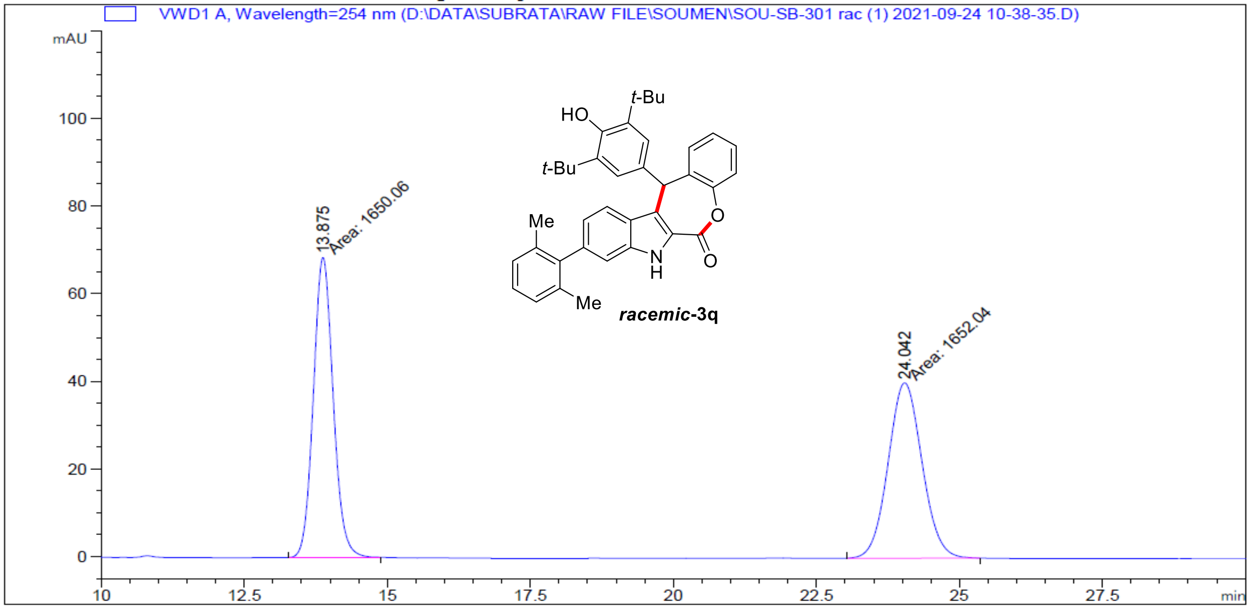

Peak #	RetTime [min]	Type	Width [min]	Area [mAU*s]	Height [mAU]	Area %
1	13.875	MM	0.4017	1650.05579	68.45570	49.9699
2	24.042	MM	0.6879	1652.04370	40.02373	50.0301

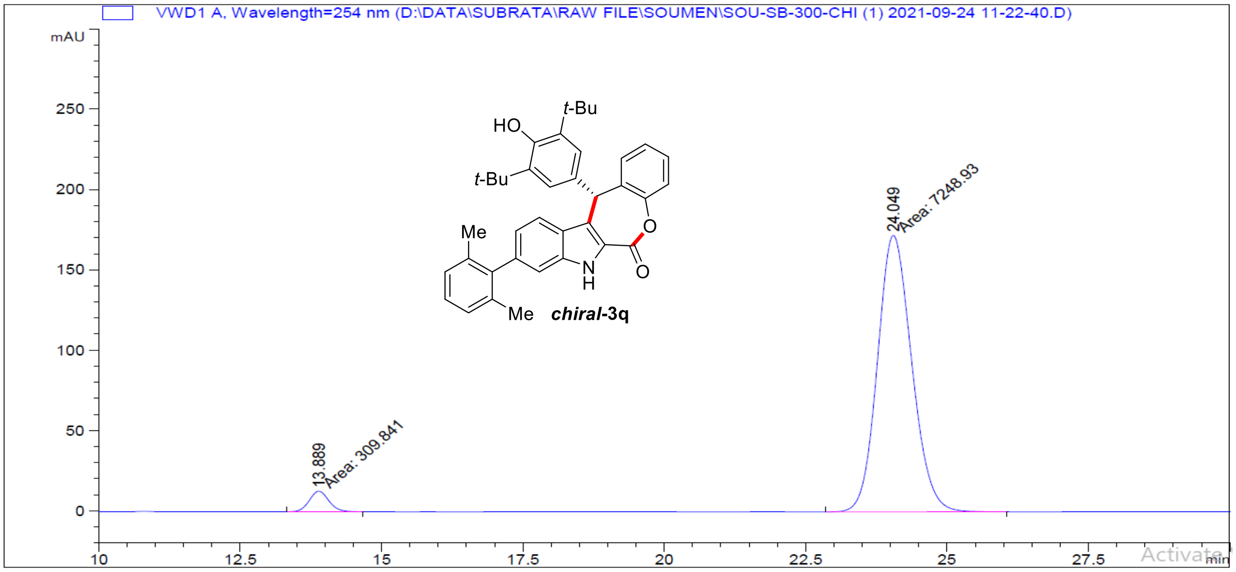

Peak #	RetTime [min]	Type	Width [min]	Area [mAU*s]	Height [mAU]	Area %
1	13.889	MM	0.4039	309.84128	12.78569	4.0991
2	24.049	MM	0.7022	7248.92920	172.06261	95.9009

Sample Info : CHIRALPAK IA, 10% IPA-HEXANE, 1.0 mL/min, 254 nm

(*S*)-12-(3,5-Di-*tert*-butyl-4-hydroxyphenyl)-9-(2,3-dimethylphenyl)-7,12-dihydro-6*H*-benzo [6,7] oxepino[3,4-*b*] indol-6-one (3r)

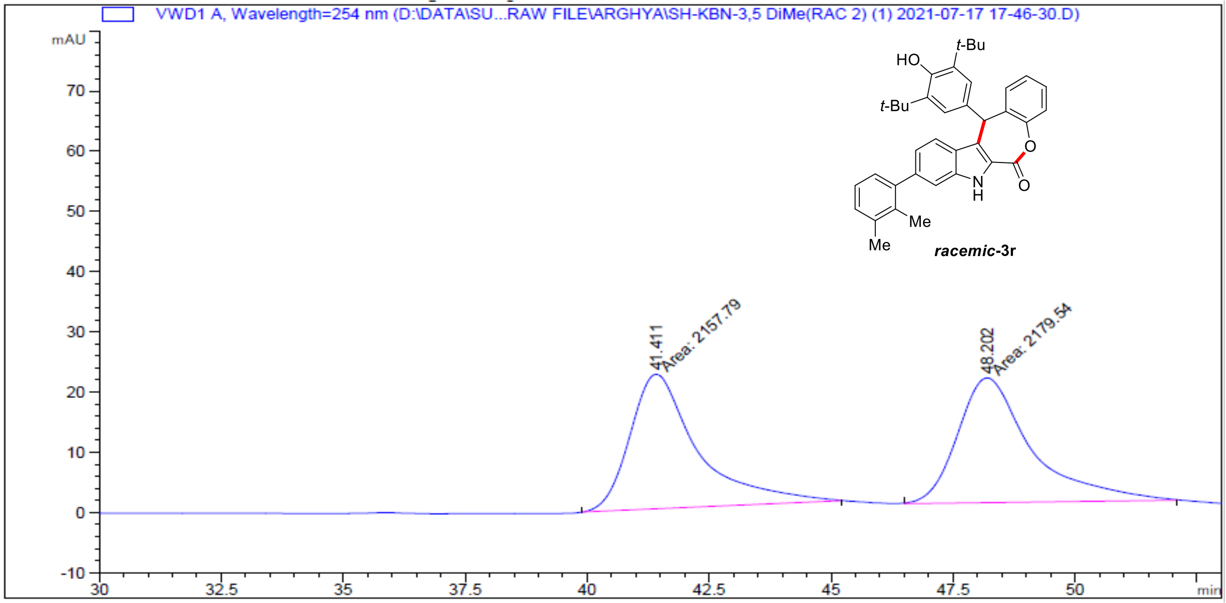

Peak #	RetTime [min]	Type	Width [min]	Area [mAU*s]	Height [mAU]	Area %
1	41.411	MM	1.6103	2157.79395	22.33299	49.7493
2	48.202	MM	1.7548	2179.54419	20.70073	50.2507

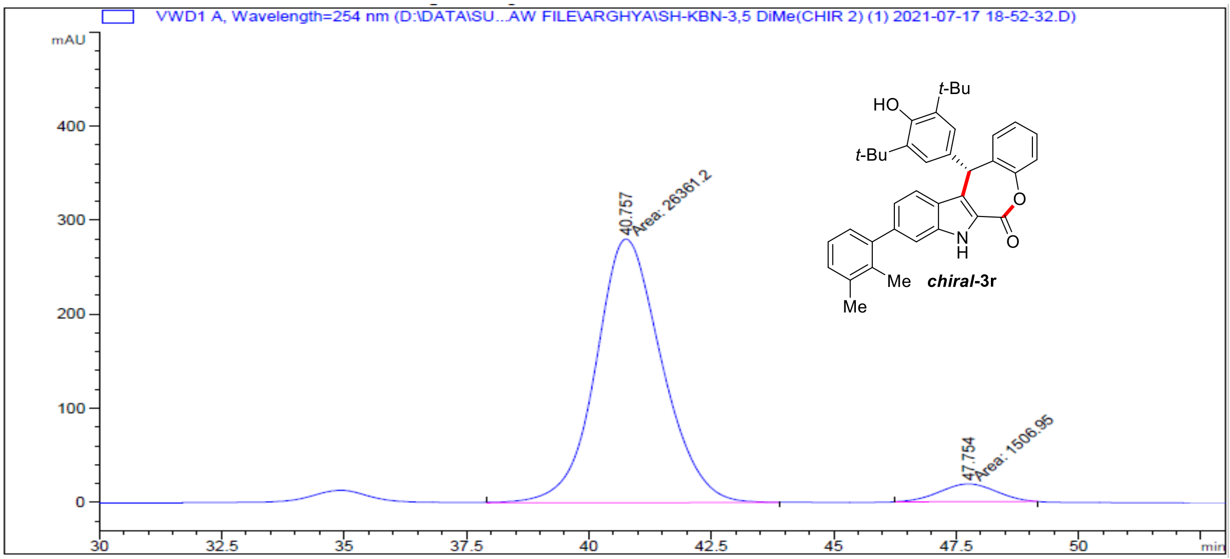

Peak #	RetTime [min]	Type	Width [min]	Area [mAU*s]	Height [mAU]	Area %
1	40.757	MM	1.5673	2.63612e4	280.31915	94.5926
2	47.754	MM	1.3370	1506.94922	18.78528	5.4074

Sample Info : CHIRALPAK IA, 5 % IPA-HEXANE, 1.0 mL/min, 254 nM.M

(*S*)-12-(3,5-Di-*tert*-butyl-4-hydroxyphenyl)-8-methoxy-7,12-dihydro-6*H*-benzo[6,7]oxepino[3,4-*b*]indol-6-one (3s)

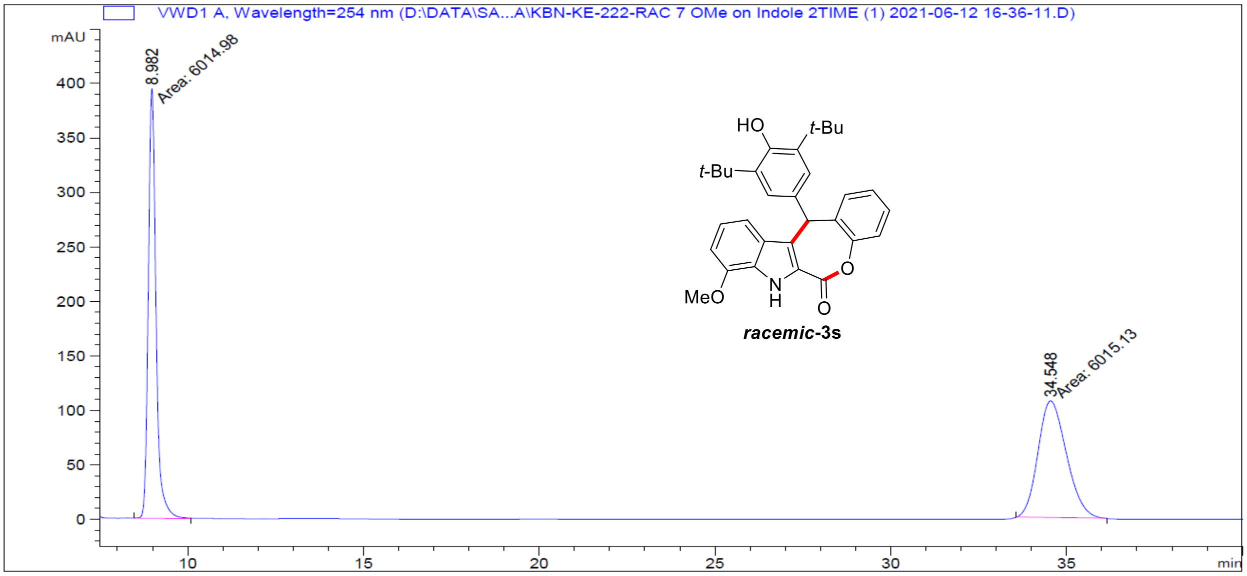

Peak #	RetTime [min]	Type	Width [min]	Area [mAU*s]	Height [mAU]	Area %
1	8.982	MM	0.2543	6014.97803	394.17563	49.9994
2	34.548	MM	0.9372	6015.12549	106.97084	50.0006

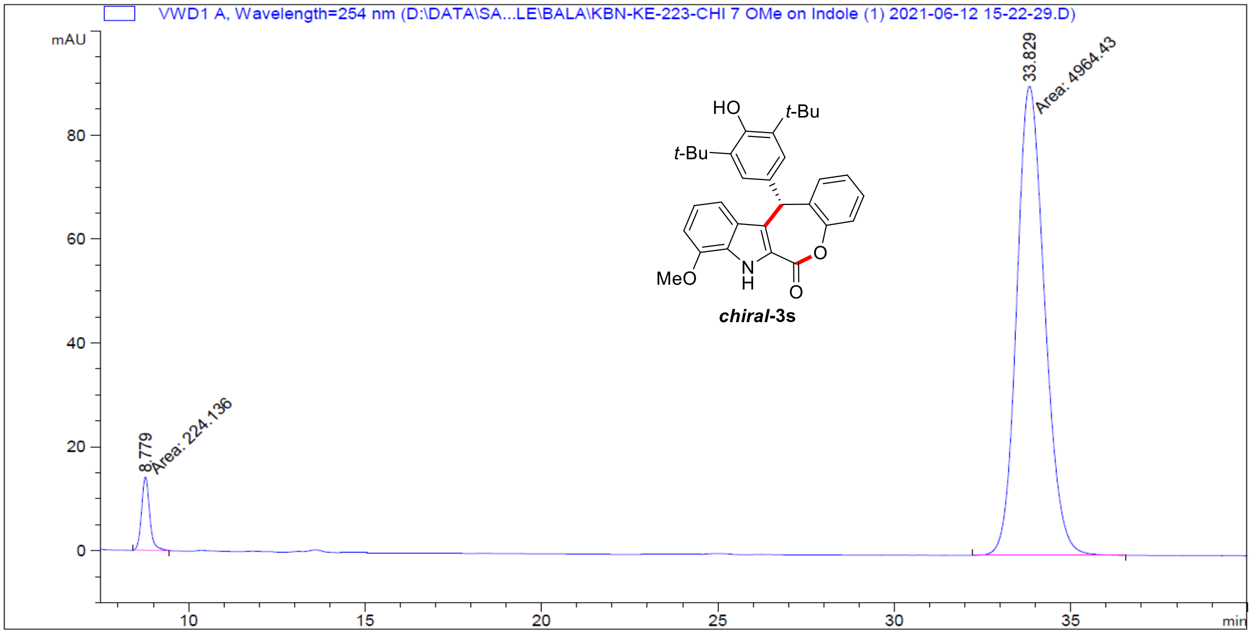

Peak #	RetTime [min]	Type	Width [min]	Area [mAU*s]	Height [mAU]	Area %
1	8.779	MM	0.2650	224.13635	14.09578	4.3198
2	33.829	MM	0.9163	4964.43311	90.29681	95.6802

Sample Info : CHIRALPAK IA, 10% IPA-HEXANE, 1.0 mL/min, 254 nm

(*S*)-7-(3,5-Di-*tert*-butyl-4-hydroxyphenyl)-7,14-dihydro-13*H*-benzo[*g*]benzo[6,7]oxepino[3,4-*b*]indol-13-one (3t)

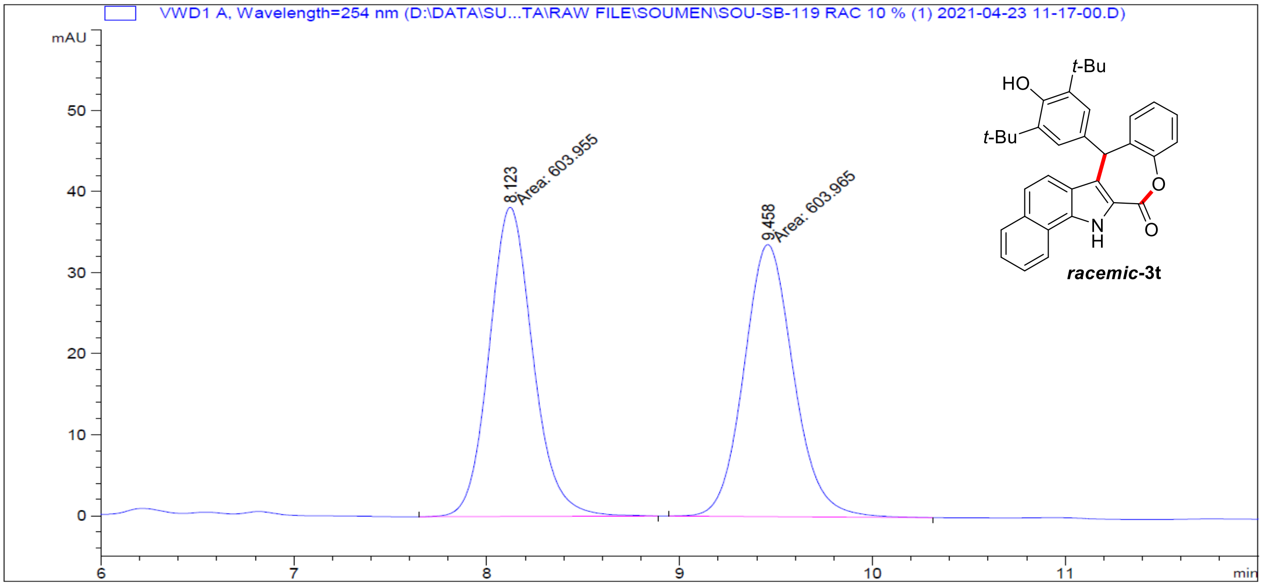

Peak #	RetTime [min]	Type	Width [min]	Area [mAU*s]	Height [mAU]	Area %
1	8.123	MM	0.2639	603.95520	38.14765	49.9996
2	9.458	MM	0.2997	603.96454	33.58168	50.0004

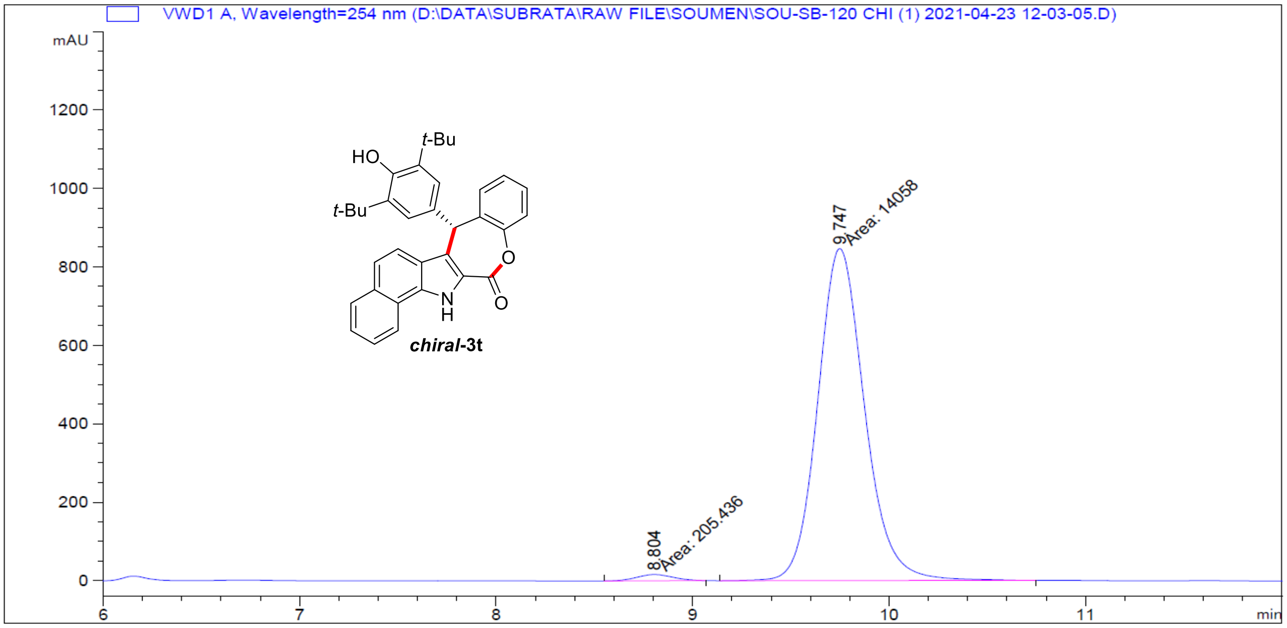

Peak #	RetTime [min]	Type	Width [min]	Area [mAU*s]	Height [mAU]	Area %
1	8.804	MM	0.2167	205.43631	15.79901	1.4403
2	9.747	MM	0.2768	1.40580e4	846.43469	98.5597

Sample Info : CHIRALPAK IA, 10% IPA-HEXANE, 1.0 mL/min, 254 nm

(*S*)-12-(3,5-di-*Tert*-butyl-4-hydroxyphenyl)-9,11-dimethyl-7,12-dihydro-6*H*-benzo[6,7]oxepino[3,4-b]indol-6-one (3u)

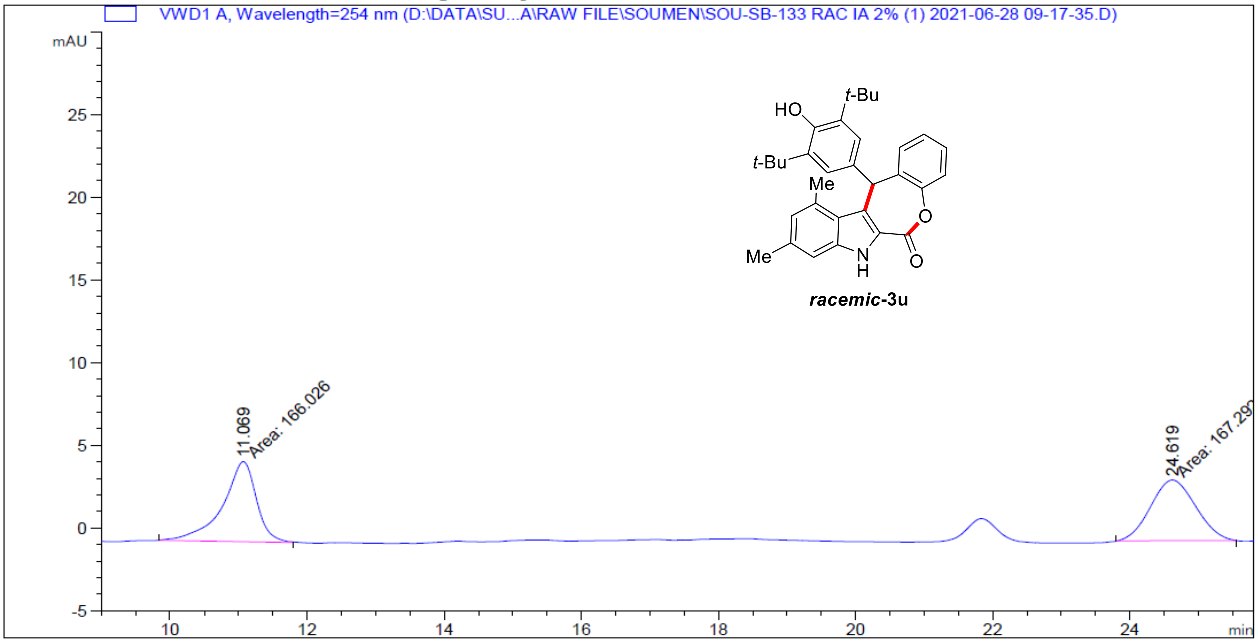

Peak #	RetTime [min]	Type	Width [min]	Area [mAU*s]	Height [mAU]	Area %
1	11.069	MM	0.5708	166.02626	4.84737	49.8101
2	24.619	MM	0.7601	167.29187	3.66826	50.1899

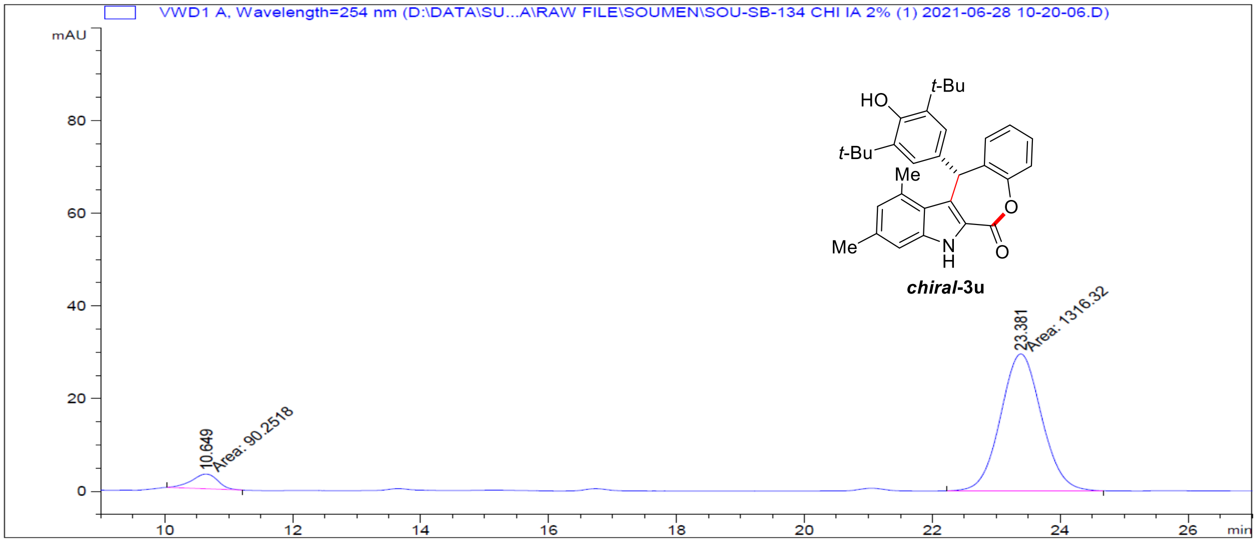

Peak #	RetTime [min]	Type	Width [min]	Area [mAU*s]	Height [mAU]	Area %
1	10.649	MM	0.4747	90.25180	3.16868	6.4164
2	23.381	MM	0.7430	1316.31995	29.52674	93.5836

Sample Info : CHIRALPAK IA, 2 % IPA-HEXANE, 1.0 mL/min, 254 nM.M

(S)-2-Bromo-12-(3,5-di-*tert*-butyl-4-hydroxyphenyl)-7,12-dihydro-6*H*-benzo[6,7]oxepino[3,4-b]indol-6-one (3v)

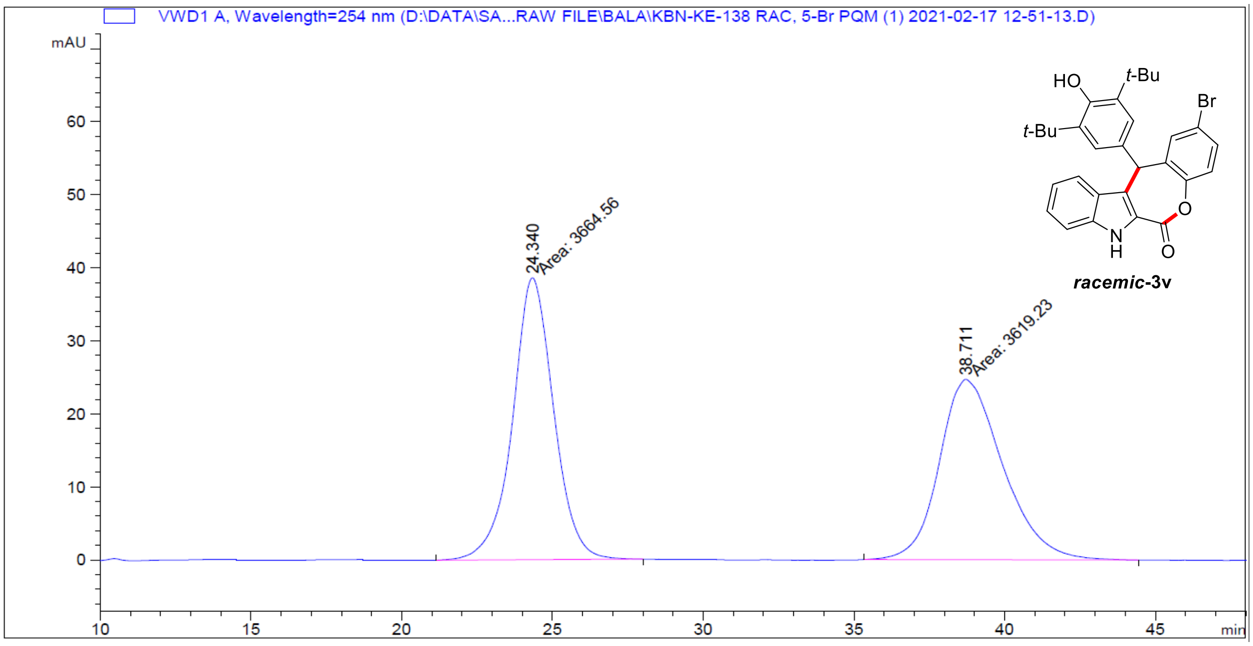

Peak #	RetTime [min]	Type	Width [min]	Area [mAU*s]	Height [mAU]	Area %
1	24.340	MM	1.5823	3664.55737	38.59905	50.3111
2	38.711	MM	2.4427	3619.23145	24.69383	49.6889

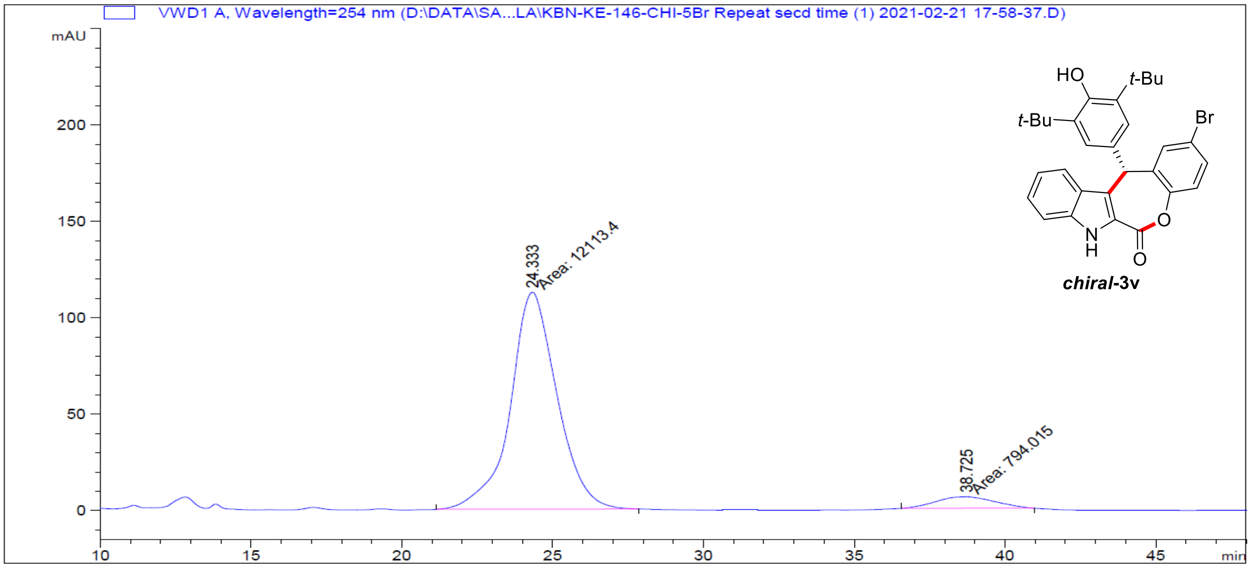

Peak #	RetTime [min]	Type	Width [min]	Area [mAU*s]	Height [mAU]	Area %
1	24.333	MM	1.7938	1.21134e4	112.54713	93.8484
2	38.725	MM	2.2220	794.01489	5.95578	6.1516

Sample Info : CHIRALCELL OD-H , 1% IPA-HEXANE, 1.0 mL/min, 254 nm

(*S*)-2-Chloro-12-(3,5-di-*tert*-butyl-4-hydroxyphenyl)-7,12-dihydro-6*H*-benzo[6,7]oxepino[3,4-b]indol-6-one (3w)

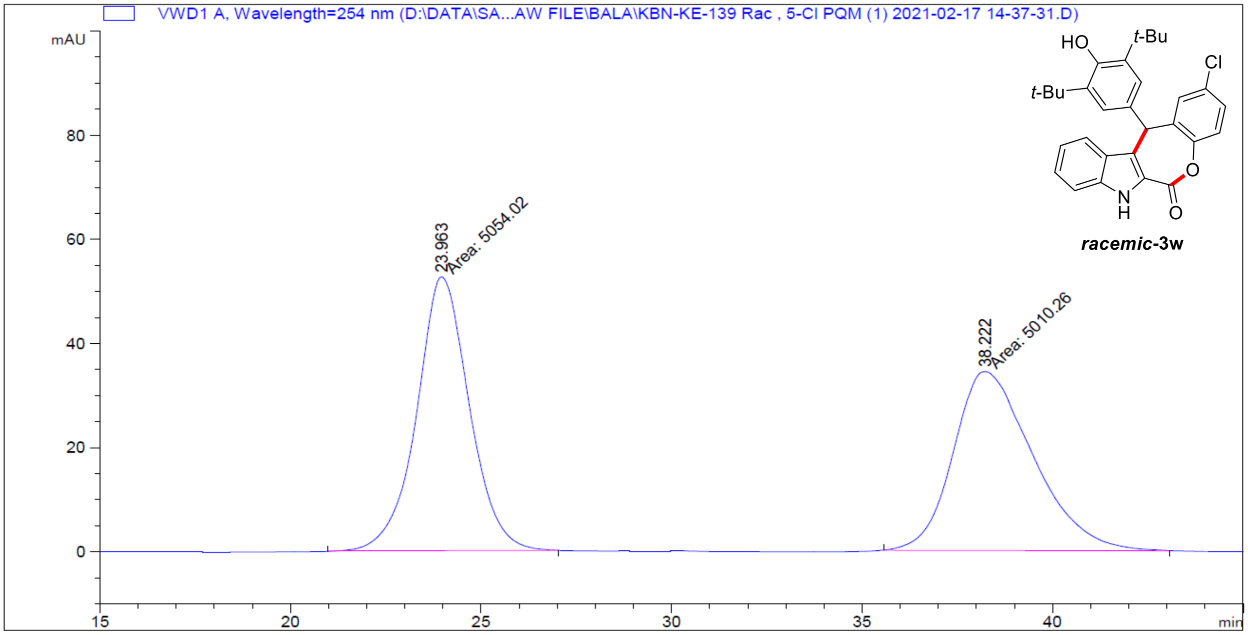

Peak #	RetTime [min]	Type	Width [min]	Area [mAU*s]	Height [mAU]	Area %
1	23.963	MM	1.6010	5054.01709	52.61432	50.2174
2	38.222	MM	2.4320	5010.26367	34.33619	49.7826

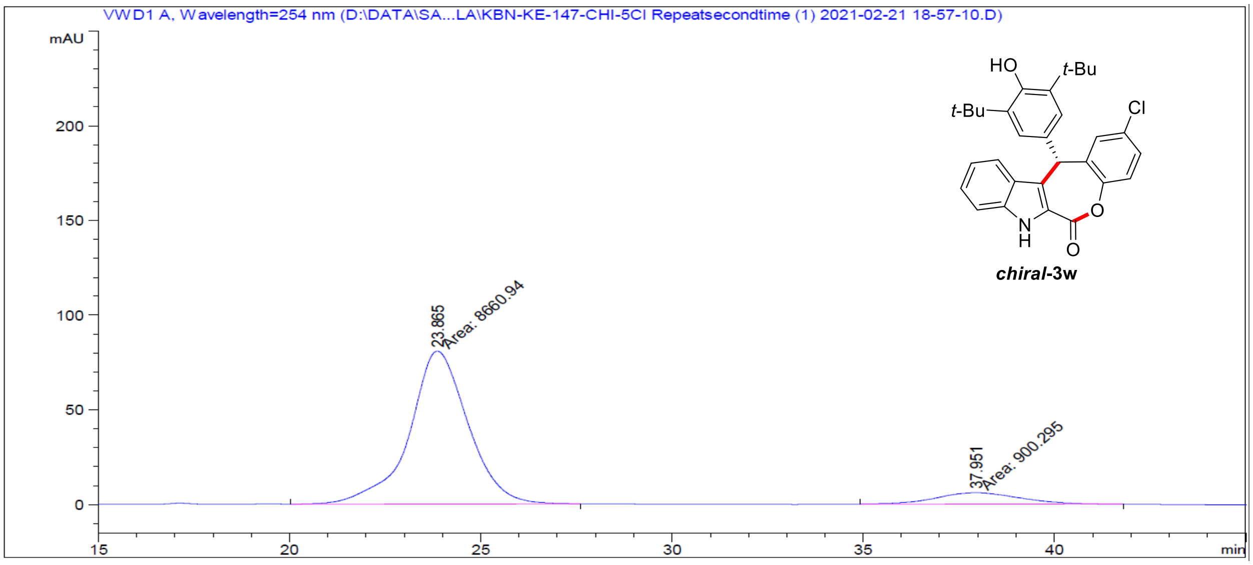

Peak #	RetTime [min]	Type	Width [min]	Area [mAU*s]	Height [mAU]	Area %
1	23.865	MM	1.7883	8660.93750	80.71883	90.5839
2	37.951	MM	2.5025	900.29535	5.99599	9.4161

Sample Info : CHIRALCELL OD-H , 1% IPA-HEXANE, 1.0 mL/min, 254 nm

(*S*)-12-(3,5-Di-*tert*-butyl-4-hydroxyphenyl)-2-phenyl-7,12-dihydro-6*H*-benzo[6,7]oxepino[3,4-b]indol-6-one (3x)

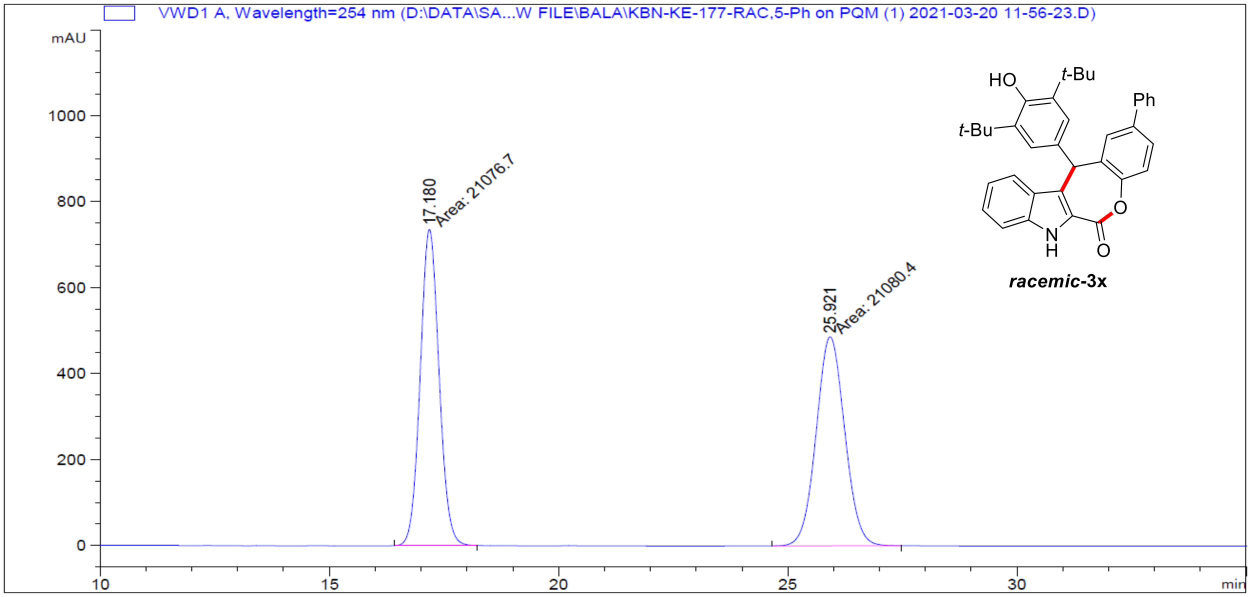

Peak #	RetTime [min]	Type	Width [min]	Area [mAU*s]	Height [mAU]	Area %
1	17.180	MM	0.4774	2.10767e4	735.84338	49.9957
2	25.921	MM	0.7225	2.10804e4	486.27020	50.0043

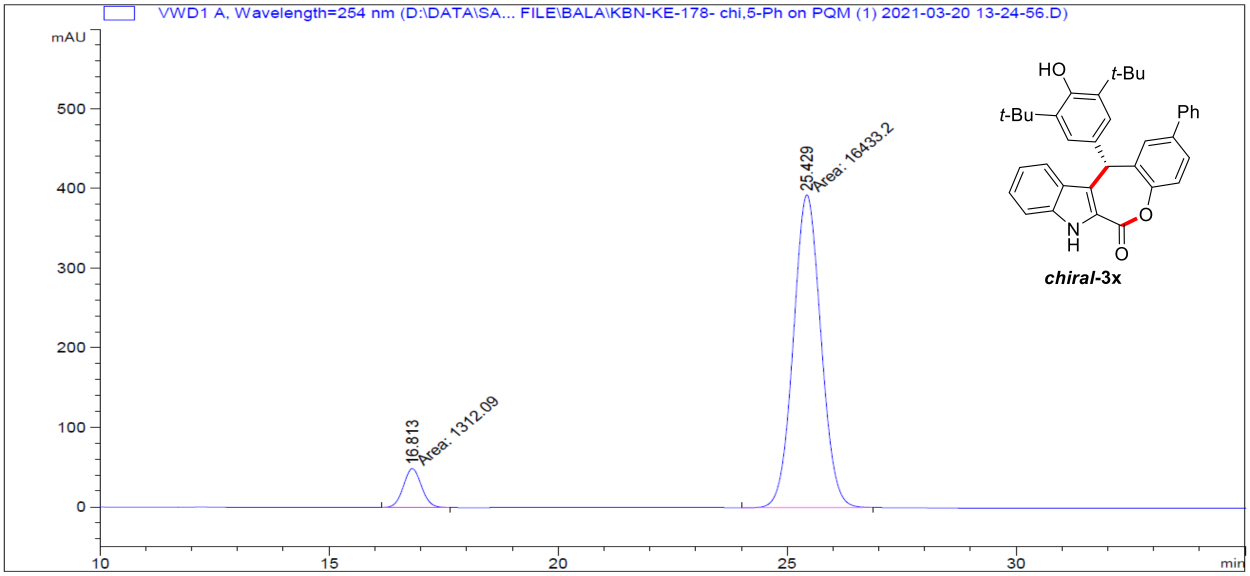

Peak #	RetTime [min]	Type	Width [min]	Area [mAU*s]	Height [mAU]	Area %
1	16.813	MM	0.4517	1312.08813	48.41216	7.3940
2	25.429	MM	0.6974	1.64332e4	392.72919	92.6060

Sample Info : CHIRALPAK IA, 10 % IPA-HEXANE, 1.0 mL/min, 254 nm

(*S*)-12-(3,5-Di-*tert*-butyl-4-hydroxyphenyl)-2-methoxy-7,12-dihydro-6*H*-benzo[6,7]oxepino[3,4-b]indol-6-one (3y)

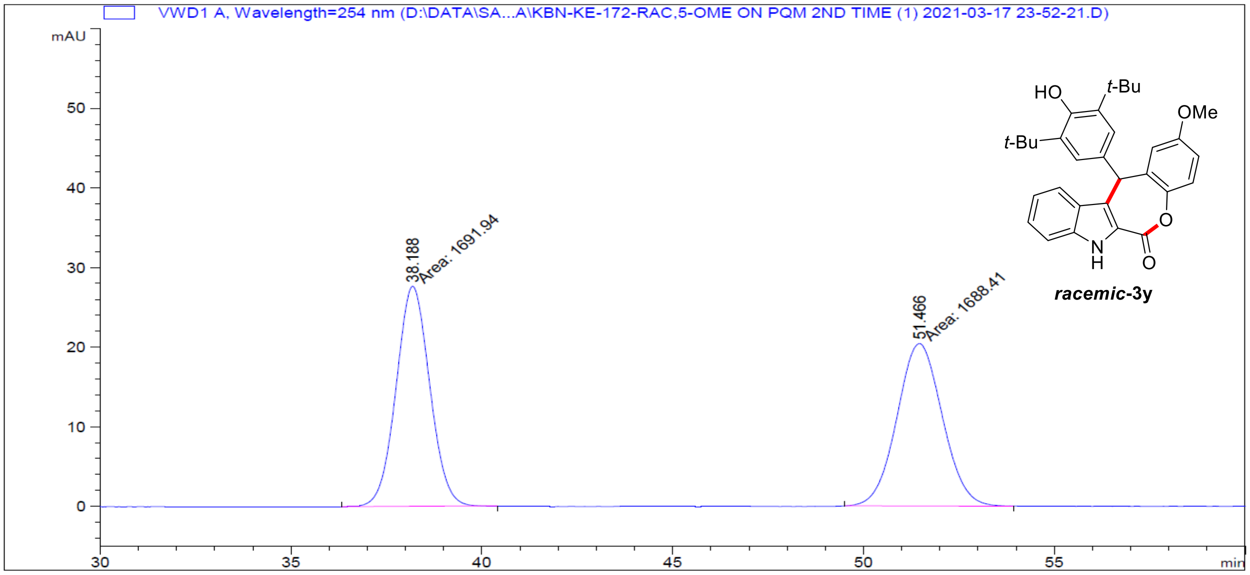

Peak #	RetTime [min]	Type	Width [min]	Area [mAU*s]	Height [mAU]	Area %
1	38.188	MM	1.0206	1691.94189	27.62939	50.0522
2	51.466	MM	1.3792	1688.41443	20.40334	49.9478

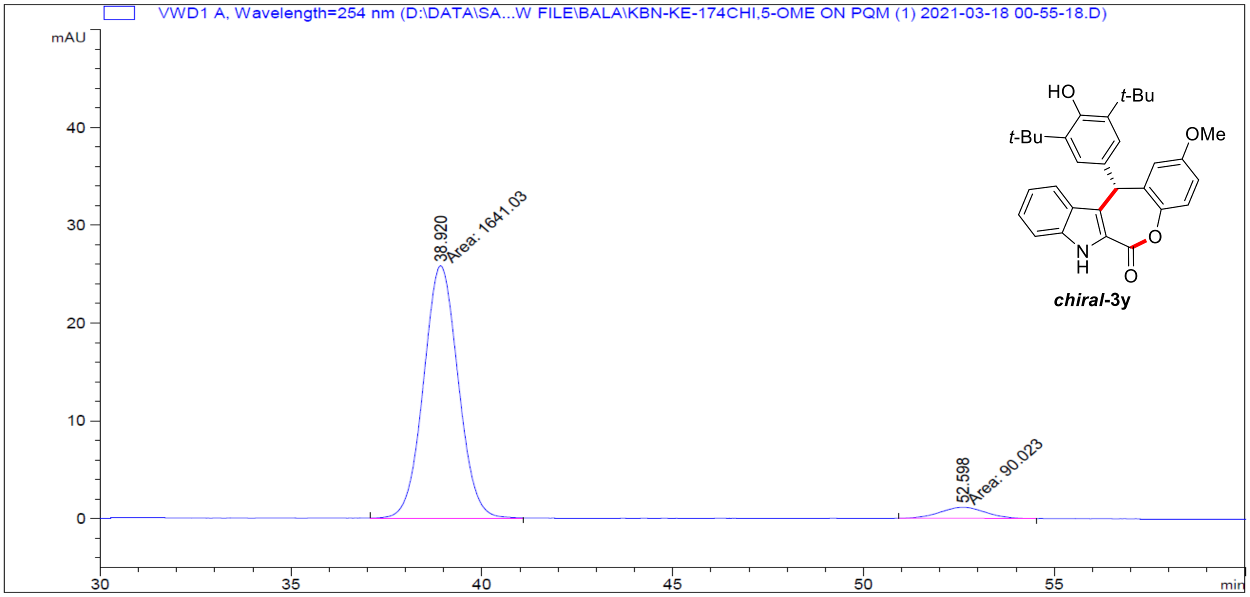

Peak #	RetTime [min]	Type	Width [min]	Area [mAU*s]	Height [mAU]	Area %
1	38.920	MM	1.0574	1641.02502	25.86586	94.7995
2	52.598	MM	1.3496	90.02300	1.11170	5.2005

Sample Info : CHIRALPAK IA, 10 % IPA-HEXANE, 1mL/min, 254 nm

(*S*)-12-(3,5-Di-*tert*-butyl-4-hydroxyphenyl)-3-methyl-7,12-dihydro-6*H*-benzo[6,7]oxepino[3,4-b]indol-6-one (3z)

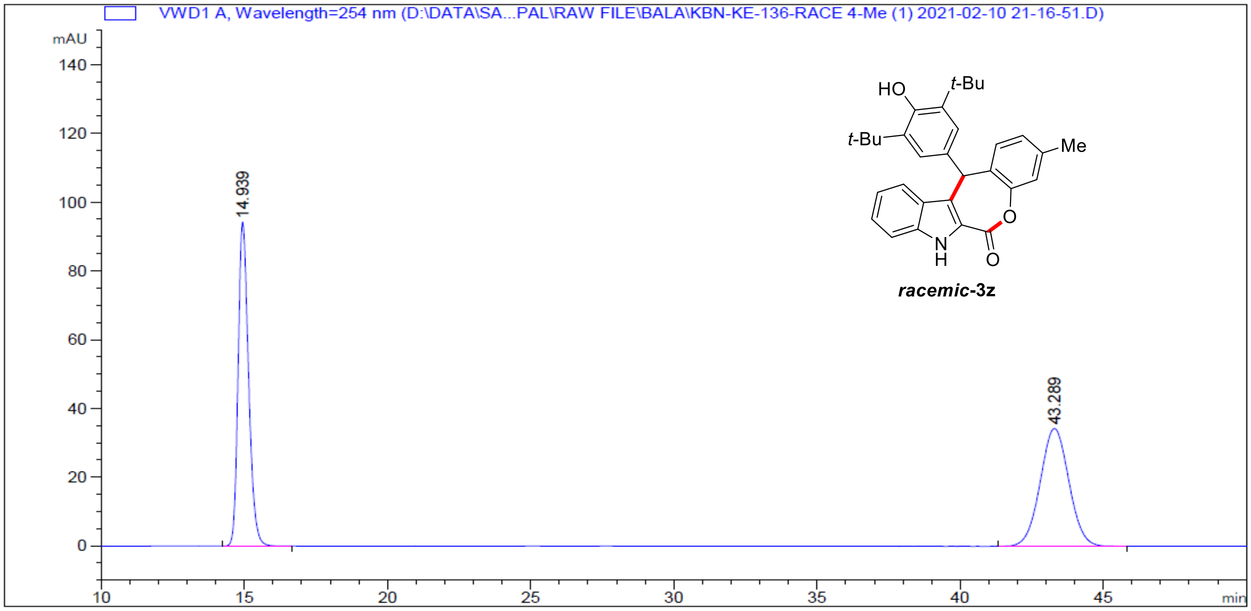

Peak #	RetTime [min]	Type	Width [min]	Area [mAU*s]	Height [mAU]	Area %
1	14.939	BB	0.3893	2386.49146	94.28596	49.8928
2	43.289	BB	1.0722	2396.75098	34.29923	50.1072

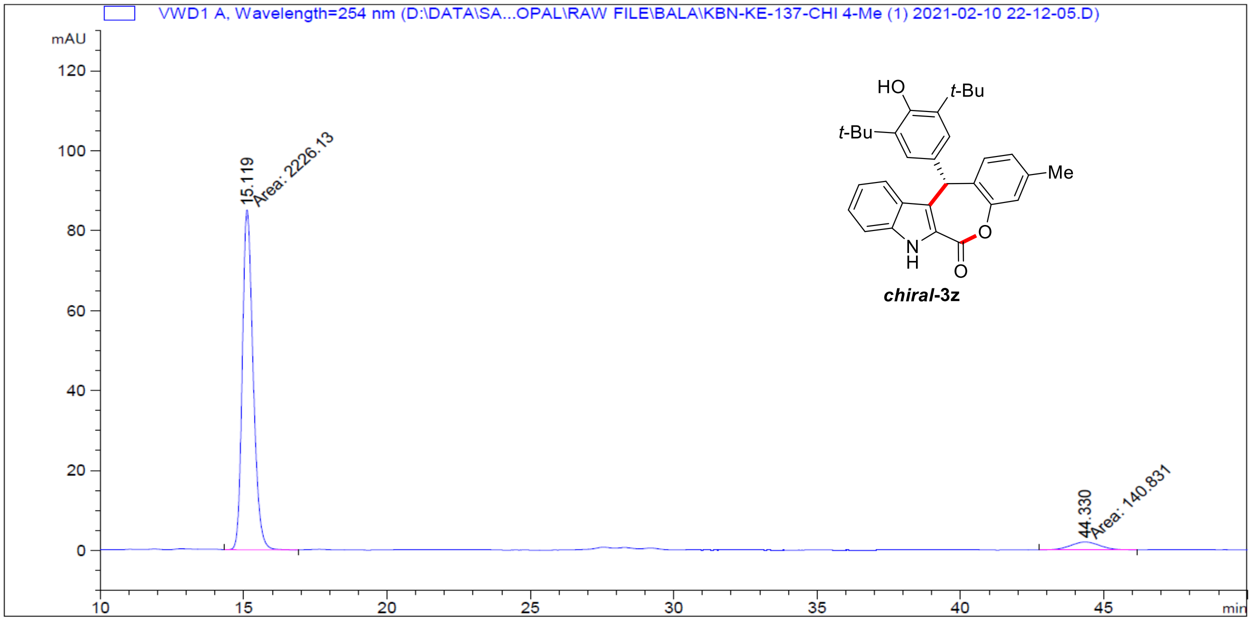

Peak #	RetTime [min]	Type	Width [min]	Area [mAU*s]	Height [mAU]	Area %
1	15.119	MM	0.4364	2226.13379	85.02112	94.0501
2	44.330	MM	1.1957	140.83144	1.96308	5.9499

Sample Info : CHIRALPAK IA, 10% IPA-HEXANE, 1.0 mL/min, 254 nm

(*S*)-12-(3,5-Di-*tert*-butyl-4-hydroxyphenyl)-4-methoxy-7,12-dihydro-6*H*-benzo[6,7]oxepino[3, 4-b]indol-6-one (3aa)

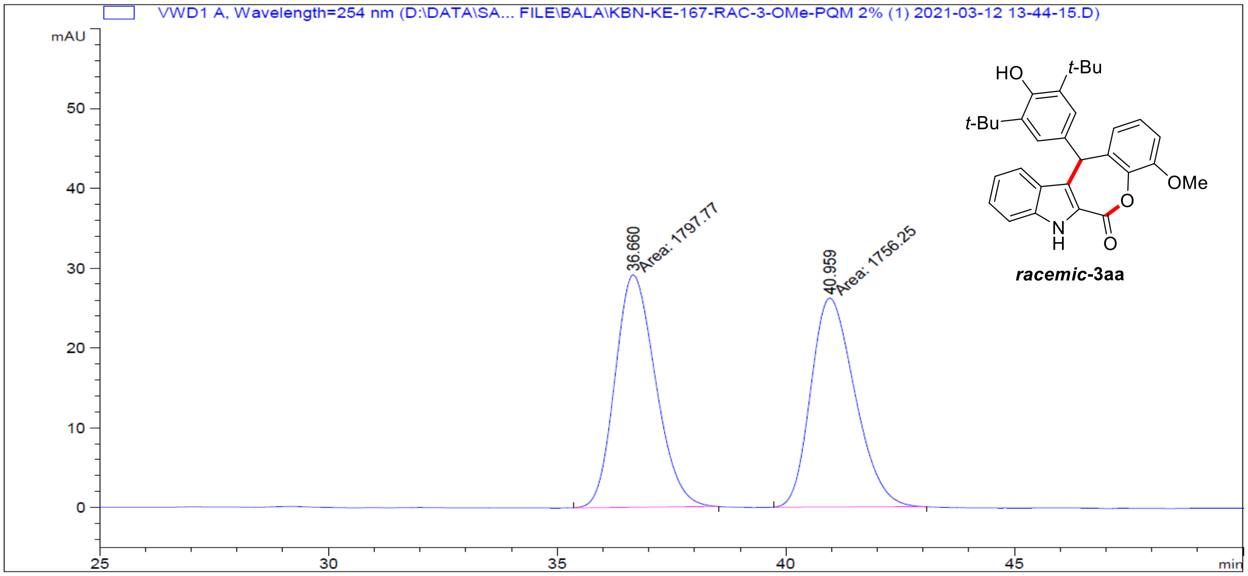

Peak #	RetTime [min]	Type	Width [min]	Area [mAU*s]	Height [mAU]	Area %
1	36.660	MM	1.0288	1797.76794	29.12329	50.5841
2	40.959	MM	1.1185	1756.24951	26.17004	49.4159

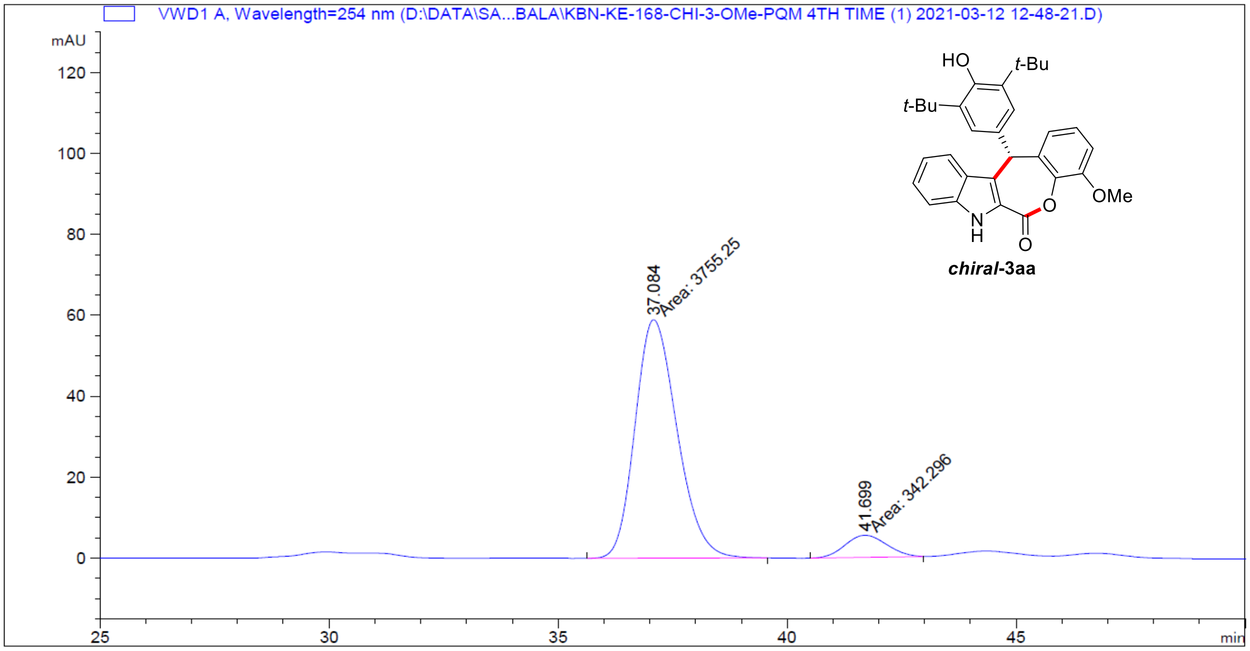

Peak #	RetTime [min]	Type	Width [min]	Area [mAU*s]	Height [mAU]	Area %
1	37.084	MM	1.0625	3755.25269	58.90541	91.6463
2	41.699	MM	1.0507	342.29568	5.42984	8.3537

Sample Info : CHIRAl PAK IA,2% IPA-HEXANE, 1.0 mL/min, 254 nm

3-((3,5-Di-*tert*-butyl-4-hydroxyphenyl)(2-hydroxyphenyl)methyl)-1*H*-indole-2-carbaldehyde (3a")

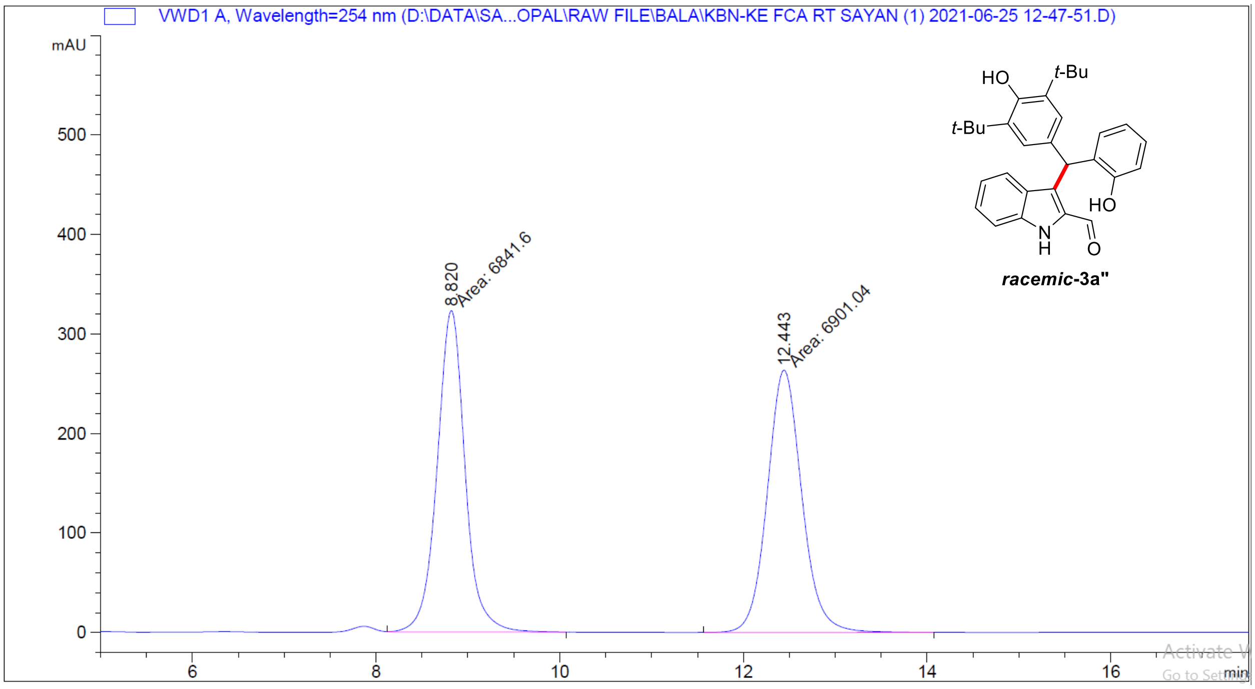

Peak #	RetTime [min]	Type	Width [min]	Area [mAU*s]	Height [mAU]	Area %
1	8.820	MM	0.3533	6841.59912	322.76346	49.7837
2	12.443	MM	0.4365	6901.03857	263.52148	50.2163

Sample Info : CHIRALPAK IA, 15 % IPA-HEXANE, 1.0 mL/min, 254 nM.M

Phenyl 3-((4-bromophenyl)(3,5-di-*tert*-butyl-4-hydroxyphenyl)methyl)-1*H*-indole-2-carboxylate (10)

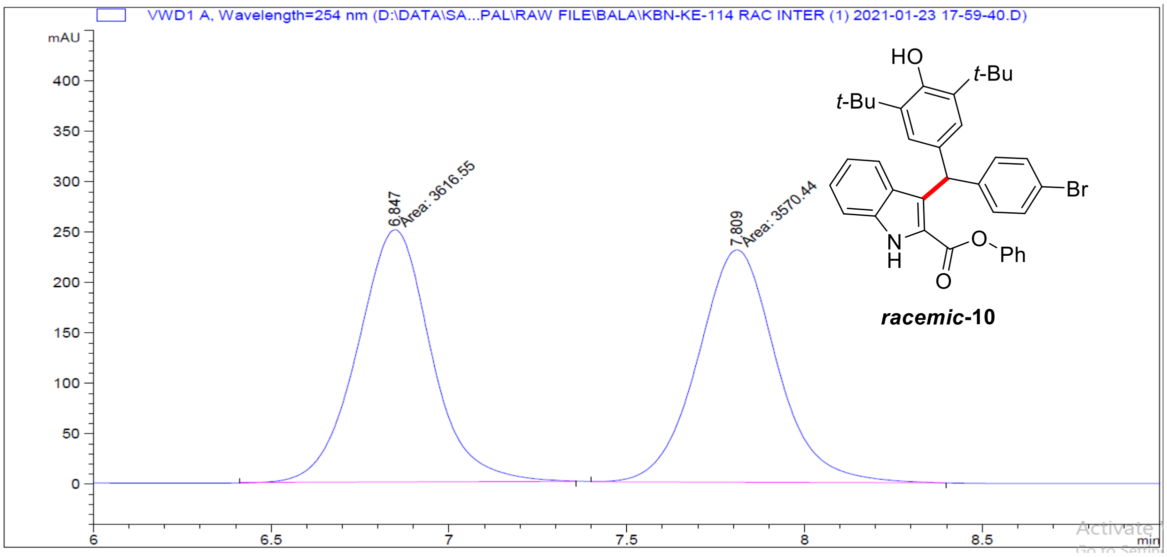

Peak #	RetTime [min]	Type	Width [min]	Area [mAU*s]	Height [mAU]	Area %
1	6.847	MM	0.2409	3616.55298	250.24260	50.3208
2	7.809	MM	0.2579	3570.44287	230.69826	49.6792

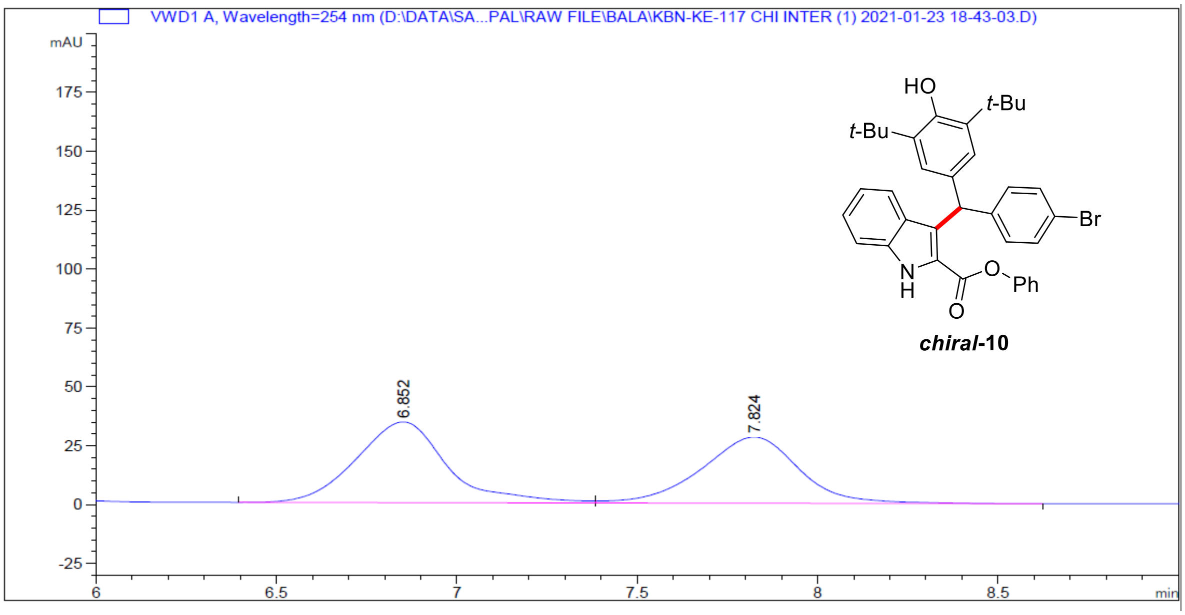

Peak #	RetTime [min]	Type	Width [min]	Area [mAU*s]	Height [mAU]	Area %
1	6.852	BV	0.2652	603.20142	34.30189	53.2210
2	7.824	VB	0.2830	530.18756	28.11667	46.7790

Sample Info : CHIRAL PAK IA,10% IPA-HEXANE, 1.0 mL/min.,254 nm
